# Simplified, interpretable graph convolutional neural networks for small molecule activity prediction

**DOI:** 10.1007/s10822-021-00421-6

**Published:** 2021-11-24

**Authors:** Jeffrey K. Weber, Joseph A. Morrone, Sugato Bagchi, Jan D. Estrada Pabon, Seung-gu Kang, Leili Zhang, Wendy D. Cornell

**Affiliations:** IBM Thomas J Watson Research Center, Yorktown Heights, NY USA

**Keywords:** Saliency, Interpretability, Explainability, QSAR, gCNN

## Abstract

We here present a streamlined, explainable graph convolutional neural network (gCNN) architecture for small molecule activity prediction. We first conduct a hyperparameter optimization across nearly 800 protein targets that produces a simplified gCNN QSAR architecture, and we observe that such a model can yield performance improvements over both standard gCNN and RF methods on difficult-to-classify test sets. Additionally, we discuss how reductions in convolutional layer dimensions potentially speak to the “anatomical” needs of gCNNs with respect to radial coarse graining of molecular substructure. We augment this simplified architecture with saliency map technology that highlights molecular substructures relevant to activity, and we perform saliency analysis on nearly 100 data-rich protein targets. We show that resultant substructural clusters are useful visualization tools for understanding substructure-activity relationships. We go on to highlight connections between our models’ saliency predictions and observations made in the medicinal chemistry literature, focusing on four case studies of past lead finding and lead optimization campaigns.

**Supplementary Information:**

The online version contains supplementary material available at 10.1007/s10822-021-00421-6.

## Introduction

Machine-learning models of quantitative structure–activity relationships (QSAR models) are staples of drug discovery research and represent some of the longest established applications of artificial intelligence in any industrial field [1–6]. Generally speaking, QSAR models apply some parametric function to relate a representation of a small molecule’s structure to an experimental measurement of a physical property, activity against a particular biomolecular target, or other observable [7].

The form of this function, which has free parameters fit to minimize deviations from experimental activity labels/values, can range from the simple straight lines to logistic curves to “random forests” of decision trees [8] to complex arrangements of neurons distributed across several or even dozens of hidden functional layers [9] Molecular representations can be simple predefined atom and substructure count-based fingerprints (e.g., PubChem fingerprints) [10] or more general hashed radial fingerprints (e.g., ECFP4/ECFP6 fingerprints) [11] or even vector-based molecular representations that are fully learned through some artificial intelligence approach [12]. Over the past several decades, multitudes of QSAR function and molecular representation combinations have been tested in the context of small molecule activity prediction, often with good results [13–16].

Graph convolutional neural networks (gCNNs) have emerged in the last ten years as attractive QSAR architectures, particularly because gCNNs combine automated molecular representation learning with molecular classification in one integrated framework [12, 17–20]. A graph convolutional encoder, for example, first converts a molecular topology into a graph (with atoms as nodes and bonds as edges) and then generates hierarchical representations of that molecule based on increasing bond separation radii around atomic centers. The “neural fingerprints” that result from encoding provide rich, multiscale vectorial representations of molecules that can, in turn, be fed into additional neural network layers that facilitate activity classification.

Given the long-established success of methods like random forest (RF) and logistic regression, activity prediction improvements from gCNNs are not ubiquitous, especially in systems for which experimental data are sparse or structure–activity relationships are evident from relatively simple features. However, the flexibility that graph-based neural architectures afford in adding additional features (e.g., protein–ligand contact maps [21]) and the broad adoption of deep neural networks (DNNs) across most scientific fields perhaps justify their use in QSAR applications [4]. An understanding of the performance of gCNNs in simpler tasks like ligand-only classification (QSAR) provides a foundation for applying them in more complex tasks like 3D target-ligand molecular classification (docking or virtual screening) or ligand-only or 3D target-ligand molecular generation.

Another possible advantage of gCNNs concerns how activity predictions can be interpreted: in theory, richer molecular representations allow for more complex or non-obvious correlations to be inferred between molecular properties/substructures and activities. While one can map certain components of fixed fingerprints to specific activity trends with methods like RF [22], the representation weights obtained through training a DNN further help highlight the features that contribute most to activity predictions.

The addition of attention weights to deep neural networks likely represents the most popular mode of adding interpretability to complicated classifiers. Indeed, numerous examples of using attention mechanisms for interpretability exist within the small molecule property prediction space and more broadly in physical modeling [23–25]. Attentive frameworks provide mechanistic insight by adding auxiliary “attention” layers to a given DNN architecture that connect input, output, and hidden layers, highlighting features that contribute to a model’s predictions. However, situations might exist in which methods other than attentive explainability are beneficial. In particular, the addition of an attention mechanism, by definition, forces the modification of the DNN architecture itself; by attaching trainable weights to every hidden layer of interest to input and output neurons, one potentially risks bypassing the development of more complex molecular features that might appear in a neural fingerprint during training.

**Fig. 1:**
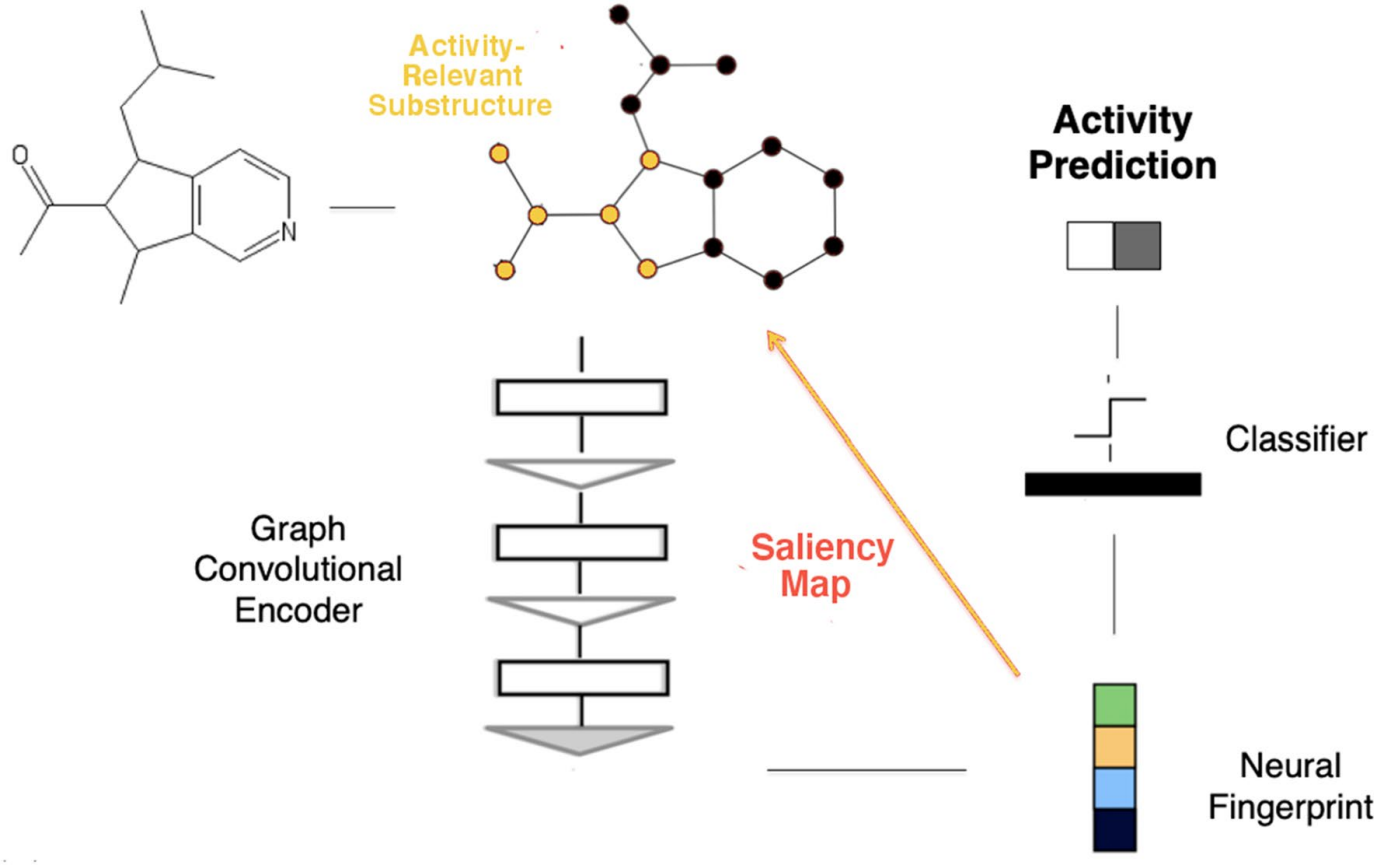
Illustration of simple saliency procedure for interpreting activity predictions taken from graph convolutional neural network QSAR models

Saliency maps offer a less intrusive mechanism for adding explainablility to QSAR models. First developed in the image recognition field [26, 27], saliency maps simply draw connections between trained weights or dependent amplitudes and the input features that give rise to them. In the context of QSAR, one might use saliency maps to establish direct relationships between components of a neural fingerprint and specific substructures within an input molecule that might relate to activity (Fig. 1). Saliency maps have already been successfully applied to identifying various protein conformations and to QSAR substructural analysis of early gCNNs [27–29].

In this work, we first simplify our past gCNN architecture [21] based on classification results averaged over a QSAR meta-dataset comprised of 788 protein targets. We then add means by which saliency maps can be calculated between input test molecules and their neural fingerprints, and we apply substructural clustering techniques to expose molecular components that might consistently result in activity against protein targets. Overall, we show that our interpretable gCNN technology yields results consistent with those of medicinal chemistry experts with respect to inferring structure–activity relationships for drug-like small molecules.

## Methods

### Curation of QSAR data for ~ 800 protein targets

To exhaustively optimize our graph convolutional neural network architecture and test our saliency procedure at scale, we needed a large and diverse set of experimental small molecule activity labels spanning a wide range of protein targets. We here leveraged a previous curation effort that extracted assay data from the ChEMBL database and converted continuous activity measures (e.g., pIC50, pKi, etc.) into binary activity labels [30]. After applying filters for redundancy and minimum dataset size, we were left with a varied set of small molecule labels distributed across 788 targets, of which approximately 500 were human. A complete target list is included in the Supplementary Information.

The mean number of activity labels in our QSAR dataset was 661 per target, with a standard [31] deviation of 834 and a range of 71 to 7839. This broad range of target-specific dataset sizes allowed us to mutually optimize our networks over data-poor and data-rich scenarios and across a half million data labels in total. Since the largest individual dataset still contained fewer than 10,000 examples, we do not here examine the high-data regime wherein graph convolutional deep learning models might be particularly expected to outpace simpler approaches.

### GCNN training and hyperparameter optimization

Our default graph convolutional neural network architecture was taken directly from our previous work comparing activity prediction and binding mode prediction use cases; that past architecture was in turn adapted from the well-studied DeepChem GraphConv network [18]. Our code is written in python using TensorFlow [32] and converts SMILES representations of ligands to graph form using the RDKit cheminformatics library [33]. In cases in which this standard architecture was trained, default hyperparameters from our previous work were used [21].

In order to minimize dataset bias and trivial correlations between our training and test sets, we applied “chronological” train/test splits [31] based on the data deposition dates made available by the ChEMBL database. Specifically, the earliest 80% of datapoints for each target were allocated to that target’s training set, while the latest 20% of datapoints for each target were assigned to that target’s test set. This chronological procedure allows one to simulate a situation of training on all past (or even present) data on a given target with the intent of predicting activities for future molecules generated as a lead series evolves or new lead series are adopted.

Default gCNNs were trained over the course of 50 epochs to generate baseline results for hyperparameter optimization. Hyperparameter optimization was then conducted using a simple, greedy Gaussian process in four dimensions: one corresponding to the L2 regularization parameter and three more corresponding to each convolutional layer size. Furthermore, the final 20% of data representing the test set was excluded from the hyperparameter optimization process. Validation sets were instead drawn from the earlier 80% data, specifically from the 70%-80% time slice for each target. During hyperparameter optimization, models were thus trained on the first 70% of chronological data and validated on the 10% range between 70%-80%, allowing for final results to be truly and fairly tested on the latest 20% of data. Models were retrained on the full 80% training set prior to testing on the reserved test set.

In this case, 80 Gaussian-distributed samples of this four-vector were tested in each iteration, and the top performing result after 50 epochs (measured by mean test AUROC, hereafter simply referred to as “AUC,” across all 788 targets) was selected in greedy fashion and reinserted as the seed for the next iteration. Results were finalized after a total of ten iterations of the Gaussian process, yielding an “optimized” gCNN QSAR architecture.

For all 788 targets, standard RF models with 100 trees were also trained on the ECFP4 fingerprint for use as a baseline.

### Saliency map procedure

As noted above, saliency maps connect internal neural network weights (or dependent amplitudes) to input features to facilitate interpretations of model predictions. In classification applications based on molecular graphs, the input features are the graph nodes and edges corresponding to the molecule being investigated. The hidden weights/amplitudes subjected to saliency analysis can be chosen arbitrarily. Our graph convolutional encoder consists of three convolutional units (each combining a graph convolutional operation and an edge-based pooling operation), a single fully-connected layer, and a gather operation that yields a 1 × 128 neural fingerprint vector. In this case, we chose the “endpoint” for our saliency analysis to be the n_atom × 128 tensor (hereafter referred to as the “saliency tensor”) output by the fully connected layer just prior to the gather operation that yields a decimated neural fingerprint. Roughly speaking, values within the saliency tensor represent convolutional amplitudes that arise for radius-3 bonded neighborhoods around each atom in the molecule. Values in the saliency tensor thus approximate the relative “importance” of various atomic neighborhoods (defined 3 bonds out from the central atom) for the network’s activity prediction.

More than one convolutional component corresponding to a given atom can contribute to the model’s output. The saliency tensor was thus sorted column-wise, with atomic indices added to and conserved for each tensor component; individual atomic neighborhoods (defined by common atomic indices) within the saliency tensor were then ranked according to a frequency-weighted amplitude modulated by a standard softmax function. The top five atomic neighborhoods were designated as salient neighborhoods for each molecule, if at least five were available upon sorting the saliency tensor (otherwise, the top k < 5 neighborhoods were chosen). A sample of pseudocode describing the TensorFlow operations that produce to our salient maps is included in the Supplementary Information alongside a detailed network diagram (Fig. S1).

For visualization purposes, these salient neighborhoods were drawn together on each molecule, with the intensity of the (orange) color decaying linearly (with a factor of 0.8/bond) as one moves away from the atomic center of a particular neighborhood. When atomic neighborhoods overlapped on given atoms, these decaying color amplitudes were simply summed to yield more intense atomic highlights.

### Substructural clustering and analysis

Once activity-salient substructures (i.e., the highest-ranked 3-bond neighborhoods) for individual molecules were defined, those groups were clustered to provide insight into substructures’ relevance across a target’s entire labeled small molecule dataset. Salient neighborhoods were first translated into SMILES strings and next converted to ECFP4 fingerprints.

At this point, molecules within a given target’s test set were subdivided into four confusion matrix categories based on both reference labels (l) and QSAR model softmax outputs (o): true positives (l = 1; o > 0.5), false positives (l = 0; o > 0.5), true negatives (l = 0; o < 0.5), and false negatives (l = 1; o < 0.5). The substructural fingerprints within each category were then subjected to a clustering procedure involving one of two clustering algorithms: density peak clustering (DPC) or k-means clustering. DPC results were given priority and used in most cases. However, if data were too sparse for a density to be computed under default DPC settings, the clustering procedure reverted to a minimal k-means clustering algorithm (k = 2). All saliency results presented in the main text of this paper were clustered with the DPC approach.

Substructural clusters across the four confusion matrix categories were next ranked according to population, from high to low, and the molecules from each cluster were plotted for visual inspection. Since the true positive quadrant is perhaps the simplest to interpret (considering all molecules are both predicted and experimentally determined to be active against the target), we here focused our qualitative analysis and comparisons with the literature on true positives only.

## Results

### Simplified graph convolutional architecture

**Fig. 2:**
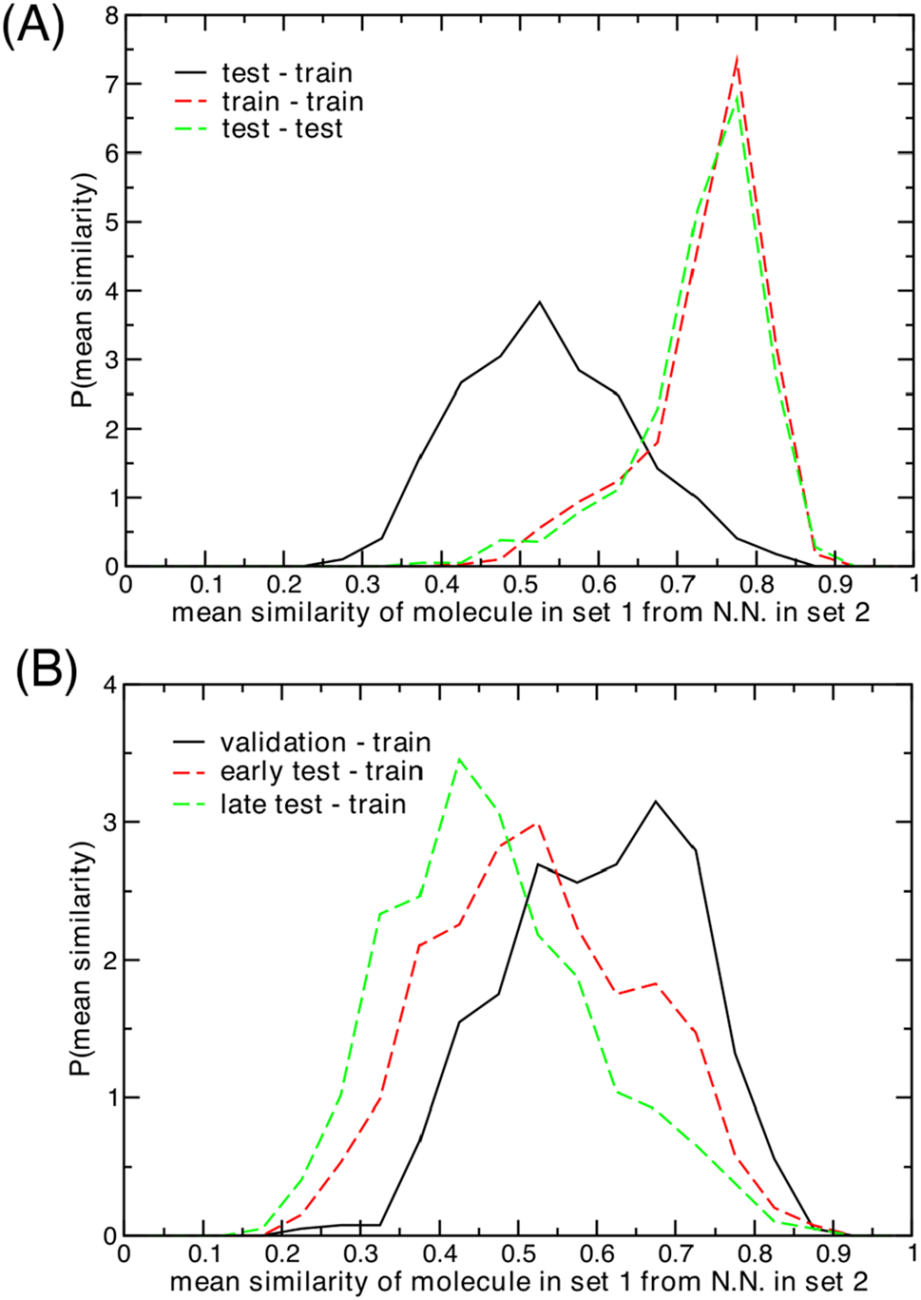
Analysis of chronological split molecular similarity statistics across 788 protein targets. Mean values over each distribution are written in parentheses. (**a**) Comparison between mean test-test (0.73), train-train (0.74), and test-train (0.53) molecular similarities. (**b**) Comparison between training set and test set time slice (validation—0.60, test-early—0.53, and test-late—0.47) molecular similarities

We first provide basic analysis of our chronological data set splitting procedure for the 788 QSAR targets studied in this work (Fig. 2). Similarity between test set and training set molecules is one the key drivers of performance in QSAR models [34], and reducing this similarity yields a more robust and realistic evaluation of a given model. Chronological splits between training and test sets have been used in the past to minimize trivial classification outcomes often observed with random splits. With random splits, highly similar molecules are likely be placed on both sides of the train-test divide. By invoking a chronological split, one assumes that molecules separated in time are, on average, less similar than molecules synthesized/characterized/deposited around the same date.

Analysis of our data confirms this relationship between time and similarity. The plots in Fig. 2 show mean nearest-neighbor Tanimoto similarities computed from ECFP4 fingerprints averaged over each individual target’s activity data set and distributed over the full set of targets. With a random train-test split, one would expect these distributions to be approximately equivalent when comparing training molecules to training molecules, test molecules to test molecules, and training molecules to test molecules.

With a chronological split (here a 70%/30% division), the “self” comparisons—train-train and test-test—produce nearly identical distributions, as one would still expect (Fig. 2a); however, the mean nearest neighbor similarities between the test and training molecules are far lower, on average. Splitting the test set into three time slices based on deposition date—70–80% (validation), 80–90% (“early” test), and 90–100% (“late” test)—one sees that molecules get progressively more dissimilar from the training set as deposition dates become later (Fig. 2b). These data indicate that our chronological splits are performing exactly as intended: time-based splits produce a gulf in molecular similarity between the training and test sets, reducing trivial correlations that machine learning models could readily identify.

**Fig. 3:**
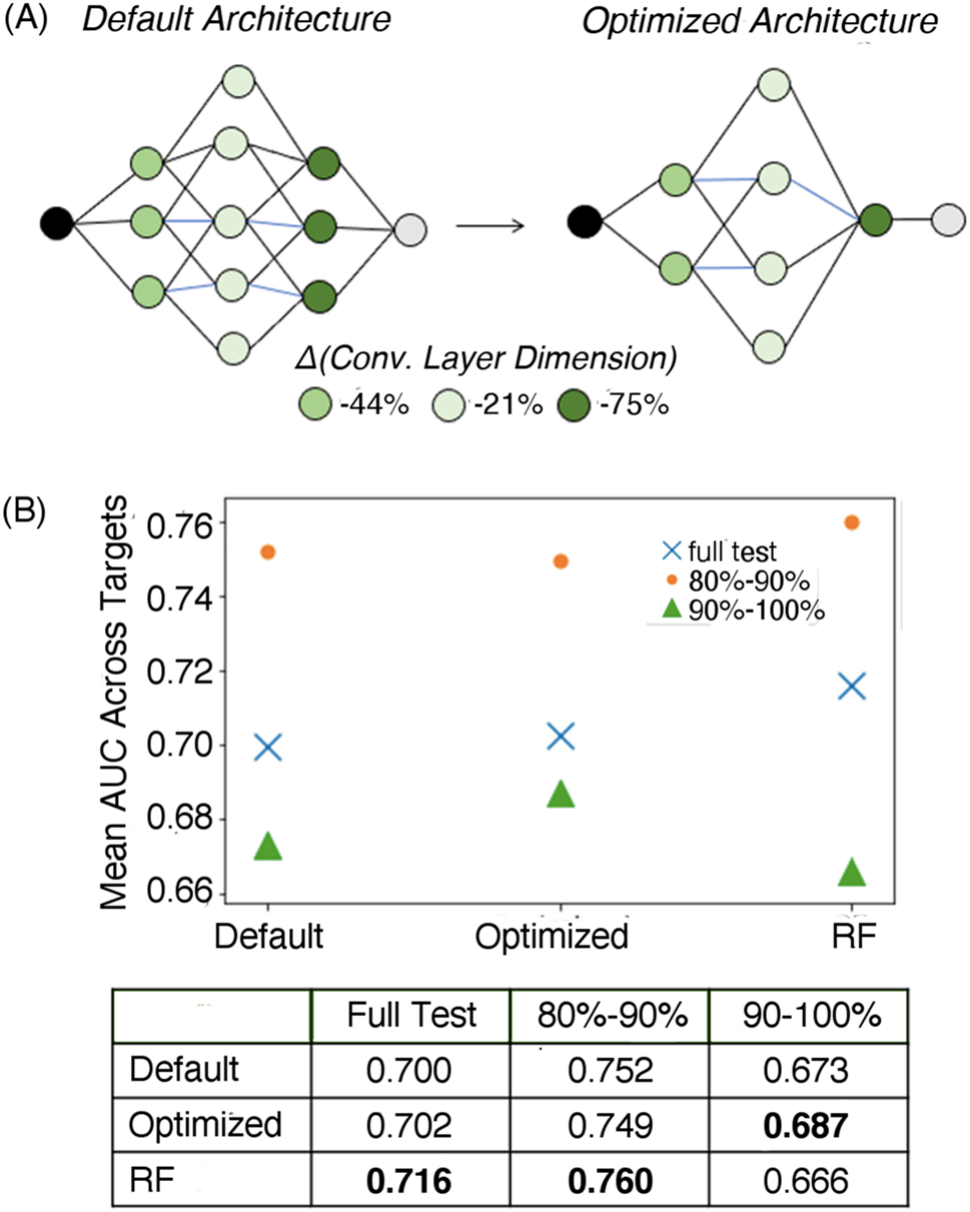
Optimization and analysis of graph convolutional neural networks employed in this work across 788 protein targets. **a** Network contraction observed as a function of hyperparameter optimization. **b** Mean AUC scores for default and optimized gCNN methods and a random forest (RF) benchmark for chronological test sets comprised of the latest 20% of data labels for each target (“full test”) and further divided into “early” (80–90%) and “late” (90–100%) test subsets. Average deviations (analogous to 95% confidence intervals) for all nine table values are ± 0.005 when assessed across the 788 protein targets

The anatomical changes to our default gCNN architecture that occurred over the course of hyperparameter optimization are shown in Fig. 3a. All three convolutional layers were reduced in size compared to the default architecture. While the middle convolutional layer was reduced by 21% in number of neurons, the first and third layers were reduced by more than twice (44%) and three times (75%) that percentage, respectively. The L2 regularization parameter increased only slightly from 0.0005 to 0.0006 during optimization.

The performance of our gCNN architecture “optimized” across the 788 targets compared with default gCNN and RF benchmarks is shown in Fig. 3b. Since our primary strategy was to optimize performance across a heterogeneous set of protein targets (thereby avoiding overfitting to individual targets), we chose to apply a confidence measure analogous to 1.96 times the standard error, but with means and “standard” deviations computed over validation/test AUC values for all 788 targets. While this metric does not yield a true 95% confidence interval (since statistics across heterogeneous systems don’t provide true standard errors), we feel this calculation does reasonably represent the range of fluctuations in AUC across the large set of targets considered in this work. For each of the three featured methods, our heterogeneous AUC confidence measure happens to converge to ± 0.005.

While the mean AUC over the 70%-80% validation time slice did improve by approximately two points (from ~ 0.76 to ~ 0.78) over the course of 10 iterations of the Gaussian process, that improvement did not translate to the independent (final 20%) test set. The optimized results only bested the default by two-tenths of a point in mean AUC (0.702 vs. 0.700), which is insignificant at 95% confidence (Fig. 3b). The RF model produces the highest mean AUC in this case (0.716), though even then only at the border of significance using our heterogeneous confidence measure. A scatter plot comparing RF and optimized gCNN results distributed across individual targets is included in the Supplementary Information (Fig. S2).

These results show that standard RF models, historically favored by pharmaceutical modeling teams, measure up quite well against DNNs over this large and diverse QSAR set. However, as we previously noted, the added flexibility and interpretability of gCNN architectures likely justify their use in cases where RF and DNN results are approximately equivalent, as they are here.

With respect to gCNN performance across the full test sets, one should note that the original DeepChem architecture had already been subjected to some manner of hyperparameter optimization. Still, our results suggest that one can significantly reduce the size of convolutional layers (particularly layers 1 and 3) and maintain or even slightly improve model performance. Based on an “Occam’s Razor” philosophy, we would argue our optimized, simpler network that contains significantly fewer parameters is the preferred model for QSAR applications like those presented in this work.

Breaking down the test set into early and late subsets (80%-90% and 90%-100% time slices) illustrates possible situations in which gCNNs might be advantageous over RFs and in which our optimized gCNN architecture might significantly improve on the default. As one can see from Fig. 3b, RF models excel on the early time slice, which has more overlap with the training set in terms of molecular structure. However, the RF models’ performance plummets by nearly 10 full points in average AUC on the late time slice. The gCNN results also decline on the late time slice, but not to the same extent. While the default gCNN results are on the border of significance in terms of improvement over RF, our optimized gCNN clearly performs better than both RF and the default gCNN at 95% confidence on that late subset. These results suggest that, even for relatively small labeled QSAR datasets, gCNNs should perhaps be favored over more classical QSAR methods like RF when training and test sets are distinctly separated in time and/or molecular similarity.

Our hyperparameter optimization of the gCNN architecture also provides some insight into the “anatomical” needs of networks of this type. In analogy to circular fingerprints like ECFP4 (which captures two-bond radii around atomic centers) and ECFP6 (three-bond radii), the second and third graph convolutional layers in our gCNN roughly capture substructures with two-bond and three-bond radii, respectively. Our optimization procedure showed that the second layer needs to be largely preserved to maintain performance, whereas the third layer can be significantly reduced in size without hurting results. However, completely decimating the third layer did harm overall performance across the validation sets, suggesting a small third convolutional is helpful. Invoking the ECFP analogy, our results suggest that our gCNN primarily leverages information like that captured by ECFP4, but still benefits from a “perturbative” or small, relatively inflexible layer at the ECFP6 level. Notably, the third convolutional layer yields the coarsest representation of molecular structure available in network, so the notion that a smaller/less flexible layer performs best with larger atomic neighborhoods perhaps makes intuitive sense.

### Qualitative saliency analysis across ~ 100 targets

We now move on to interpreting the activity predictions produced by gCNN QSAR models trained in this study. The usefulness of saliency analysis for understanding relationships between chemical substructures and molecular activity depends on the quality of the underlying QSAR model: if a model incorrectly classifies the activity of a molecule, the substructural contributors to that prediction will likely also be flawed. Furthermore, the richness of salient substructural clusters is also likely to be correlated with the amount of data available for training the underlying QSAR model, as smaller training sets will naturally be less diverse, on average.

**Fig. 4:**
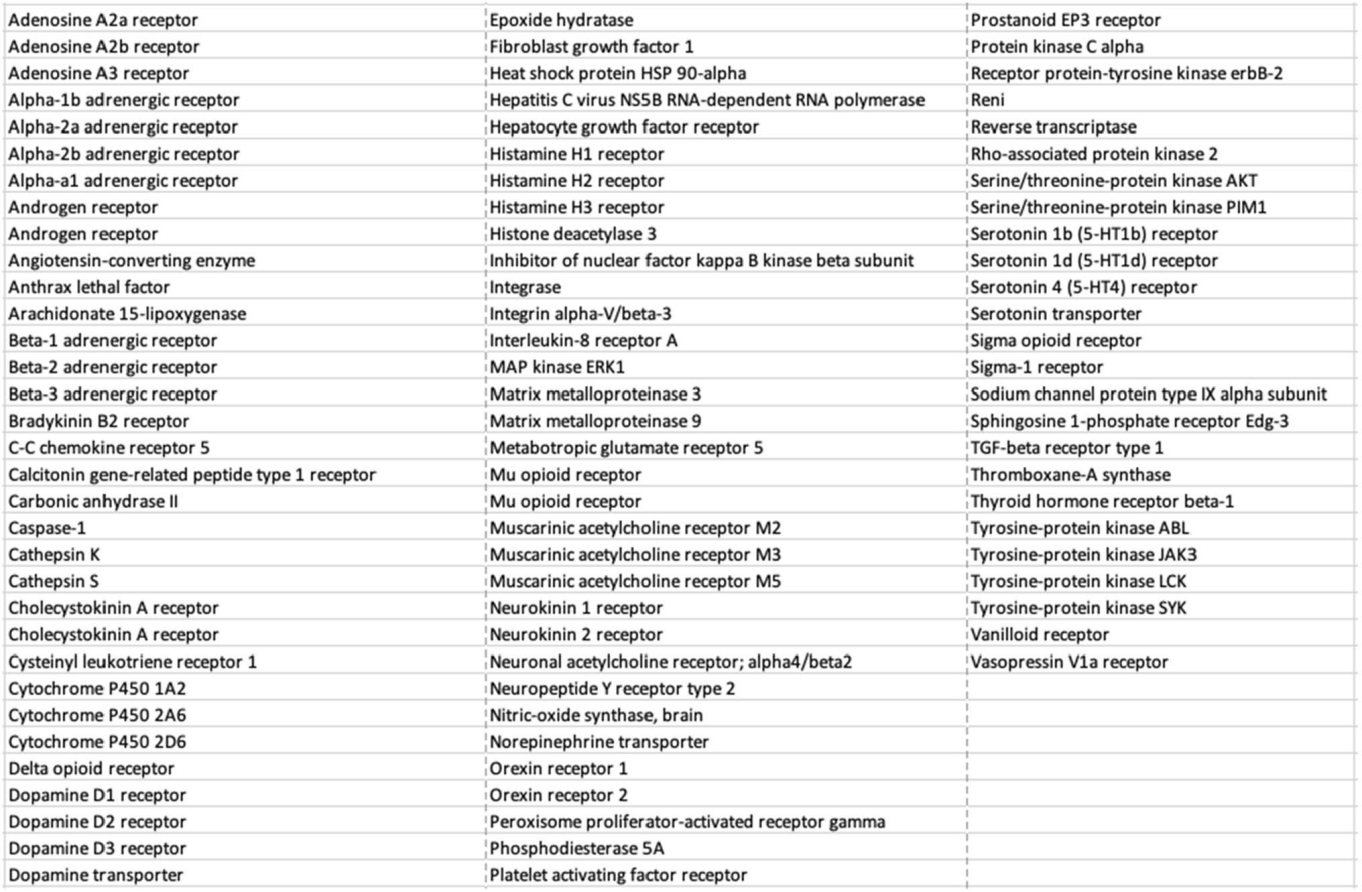
List of high-performing and relatively high population targets subjected to saliency analysis in this work

We therefore identified our top candidate protein targets for saliency analysis based on two selection criteria: the gCNN AUC on the chronological test set (latest 20% of data) and the total number of labeled molecules available for training and testing. Approximately 100 targets fit had AUCs greater than 0.80 and total dataset sizes greater than 500 molecules. The identities of these targets are shown in Fig. 4; saliency maps for true positive clusters across each of these targets are included in the Supplementary Information. We present saliency results for test sets of models trained using the optimized gCNN architecture below.

**Fig. 5:**
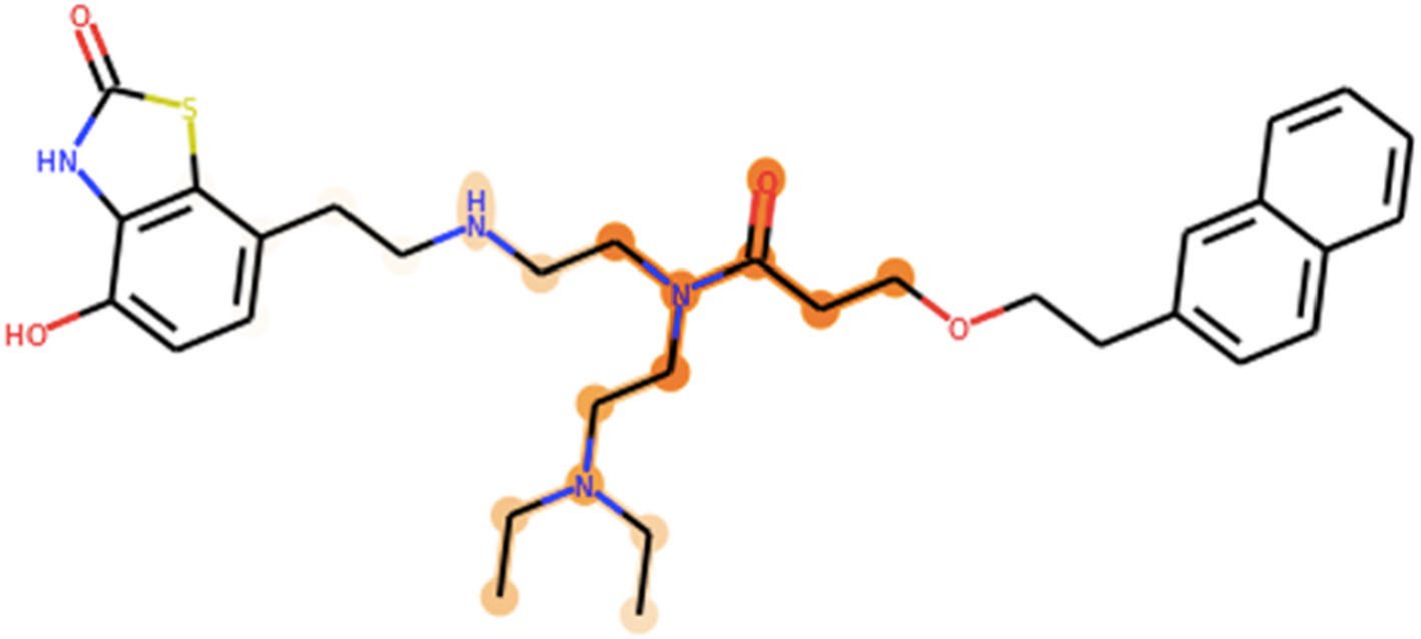
Example of an activity saliency map (visualized through orange highlights of varying intensity) projected onto a drug-like small molecule

An example of an activity saliency map visualized on a small molecule (active against the β_2_-adrenergic receptor) is shown in Fig. 5. As described in our methods section, orange highlights correspond to saliency weights on individual atoms; since these weights are derived from neighborhoods extracted from three successive graph convolutions, highlighted atoms are generally connected into substructures with bond radii of three or more. The intensity of an atom’s orange highlight is positively correlated with the importance the saliency map assigns to that atom across one or more neighborhoods. For example, the saliency map in Fig. 5 assigns the highest weight to the carbonyl oxygen and amide bond in the center of the molecule, and lower but still significant weights to the tertiary amine at bottom. The potential significance of this highlighted substructure in chemical/lead optimization terms is discussed below.

**Fig. 6:**
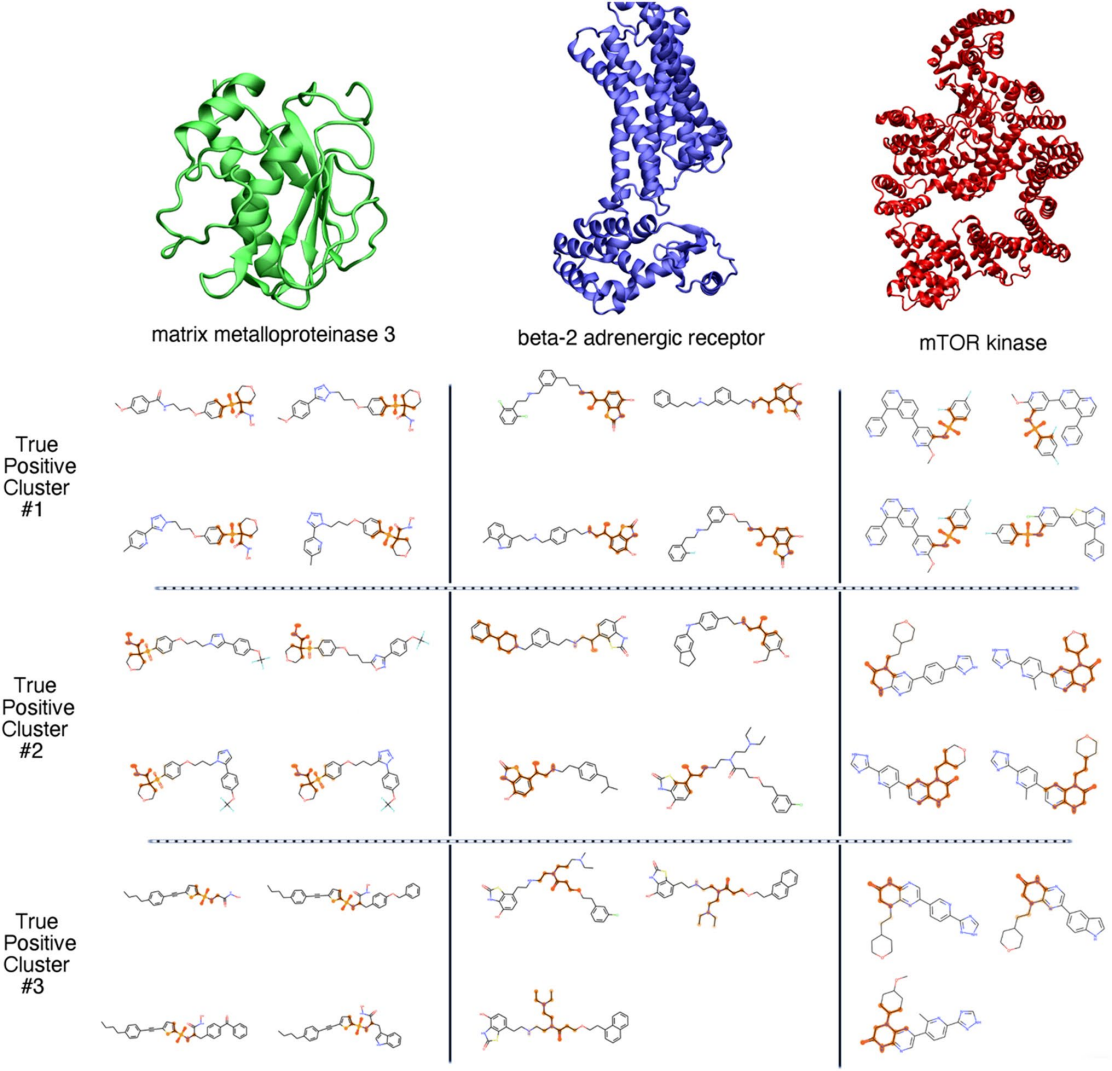
Representative molecules and highlighted activity-salienct substructures for three true positive clusters across three protein targets

The saliency map plots shown in Fig. 6 provide a qualitative sense of how well our substructural clustering algorithm performs in generating unified pictures of substructure-activity relationships. Here, we chose to highlight results on three protein targets of varying size and function: the smaller matrix metalloproteinase-3 (MMP-3), the midsized, membrane-bound β_2_-adrenergic receptor (β_2_-AR), and the larger human mTOR kinase (mTOR). In each case, the underlying QSAR model performed exceptionally well (with an AUC > 0.90 on the test set) on relatively large, labeled datasets (> 1000 labeled molecules in total for each target).

Generally speaking, one can observe common trends in the substructures highlighted in most of the clusters. For example, highlighted sulfonyl groups dominate all three clusters for MMP-3, the same bicyclic motif is highlighted across all four molecules in cluster #1 for β_2_-AR, and similar ring systems are highlighted in clusters #2 and #3 for mTOR. While our clustering scheme is certainly imperfect (the highlighted substructures in cluster #2 for β_2_-AR, for example, are quite disperse), these clusters do seem to serve their intended purpose of grouping activity-salient substructures in most cases. It is important to note that the quality of clusters (which serve as simple visualization tools) does not reflect on the quality of the underlying saliency analysis.

### Saliency case studies: comparisons with the medicinal chemistry literature

While our qualitative saliency results seem to be consistent with expectations (e.g., our saliency map highlights localized substructures, and those substructures are generally clustered together for a given target), a true test of an interpretable model needs to involve comparisons with practical data and analysis generated by domain experts. In this case, the most relevant domain experts are medicinal chemists carrying out small molecule lead finding and lead optimization studies for specific protein targets. Accordingly, we now present several case studies that relate our saliency map results to specific medicinal chemistry campaigns published in the literature.

#### Case study #1: β_2_ adrenergic receptor

**Fig. 7:**
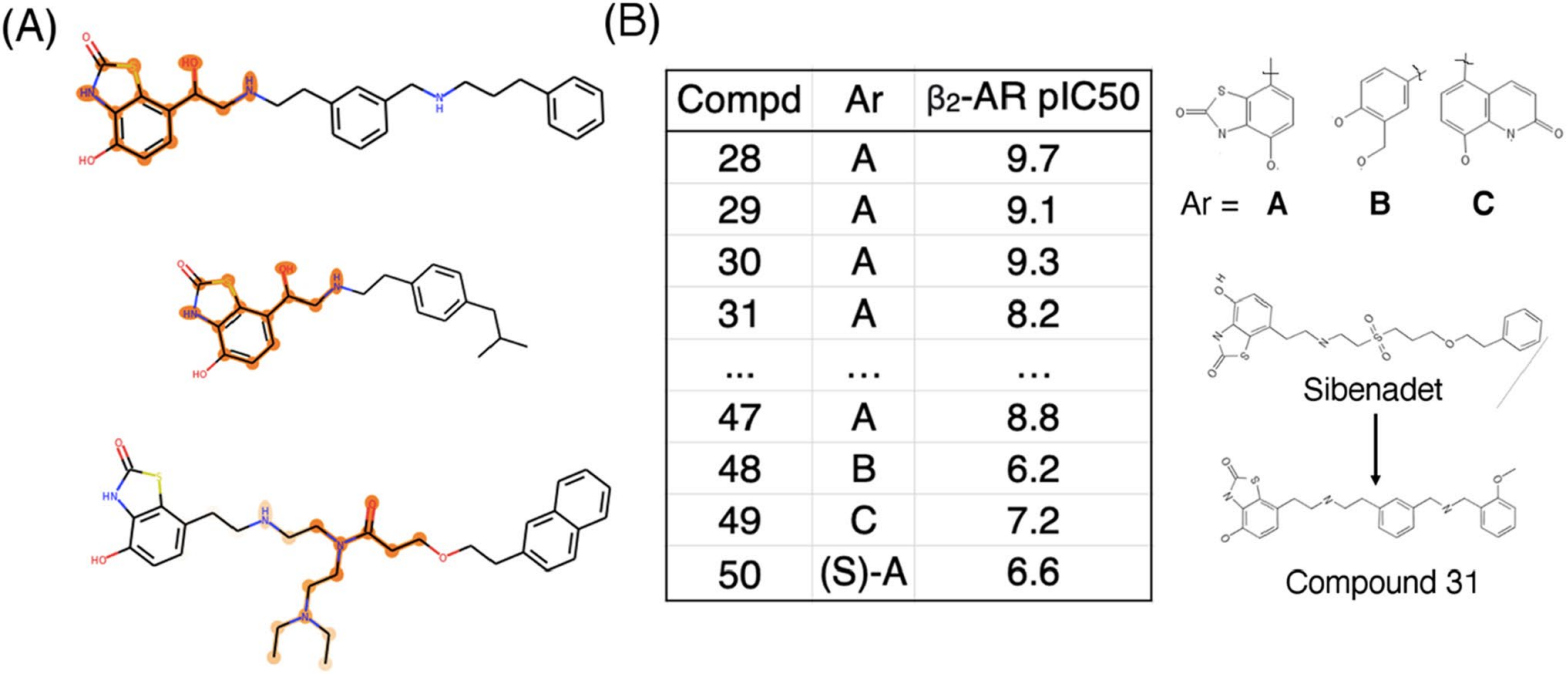
Case study for lead finding and optimization based on known β_2_-AR agonists. **a** High-level summary of substructural classes highlighted by saliency maps in this work. **b** Previously published optimization of targeted library of molecules related to past candidate β_2_-AR agonist sibenadet

Three saliency map clusters corresponding to our gCNN QSAR model for the β_2_ adrenergic receptor are presented in Fig. 6. For the most part, these three clusters favor two different substructural motifs with respect to saliency predictions: a benzothiazolone head group in the top two clusters and a”linker” between the benzothiazolone group and a generally lipophilic group on the opposite end of the molecule (Fig. 7a). Do these substructural classes make intuitive sense from the perspective of medicinal chemistry?

The source publication for the molecules featured in our saliency maps represents an effort to identify long-acting dibasic agonists of the β_2_ adrenergic receptor [35]. In particular, the authors generate a focused compound library surrounding the nearly approved β_2_-agonist sibenadet (Viozan), with the aim of identifying molecules with further improved potency, specificity, and other properties.

As illustrated in Fig. 7b, sibenadet follows the benzothiazolone head group/lipophilic tail group motif observed in most molecules shown in the central column of Fig. 6. Within the focused library, the authors make several modifications to the overall sibenadet scaffold: replacement of the benzothiazolone with alternative headgroups, replacement of the here-dubbed “linker” portion of the molecule, and the modification of the terminal lipophilic group. The authors show that, in agreement with a past research program, the benzothiazolone warhead is essential for maintaining potent activity, with Compounds 48–50 losing much of their activity upon headgroup replacement. Our saliency procedure was thus likely correct in highlighting that headgroup as an activity-relevant substructure. Lead finding efforts focused first on modifying the scaffold of the central (“linker”) portion of the molecule; the mono-phenyl lead series evident in the top two β_2_ clusters in Fig. 6 was selected based on β_2_ binding properties. Accordingly, we would argue that our saliency procedure identified both the warhead that needed to be retained and (albeit less clearly) the linker that needed to be modified to improve the potency and properties of sibenadet. The compound series that progressed to further optimization in the paper held this warhead and a chosen linker motif constant.

Admittedly, the activity-relevant substuctural predictions presented here relate to motifs that would likely be somewhat obvious to the medicinal chemists involved in the published work. One should note that our gCNN-based saliency observations were made with no knowledge or input from that work and were based purely on publicly available activity data. The more subtle lead optimization that occurred at the R3 position in the paper and not captured in our featured saliency clusters was partially focused on compound specificity (with the particular avoidance of related GPCR targets). One could certainly build off-target gCNN QSAR models to incorporate more specificity-relevant information into our saliency predictions.

#### Case study #2: Matrix metalloproteinase-3 (MMP-3 or Stromelysin)

MMP-3 (Stromelysin), featured on the left in Fig. 6, is a member of the broader MMP family. In general, MMPs are characterized by a catalytic Zn (which most inhibitors target), an adjacent beta-strand (which binds the substrate backbone), and an S1’ pocket (where the substrate P1’ sidechain binds and which is used to tune for selectivity). Hydroxamic acids, carboxylic acids, and thiols are commonly employed as Zn-binding groups. The landmark Ciba-Geigy compound CGS27023 [36] introduced a sulfonamide group to interact with the beta-strand amide group in the binding pocket and many subsequent series possessed either a sulfonyl or sulfonamide group.

**Fig. 8:**
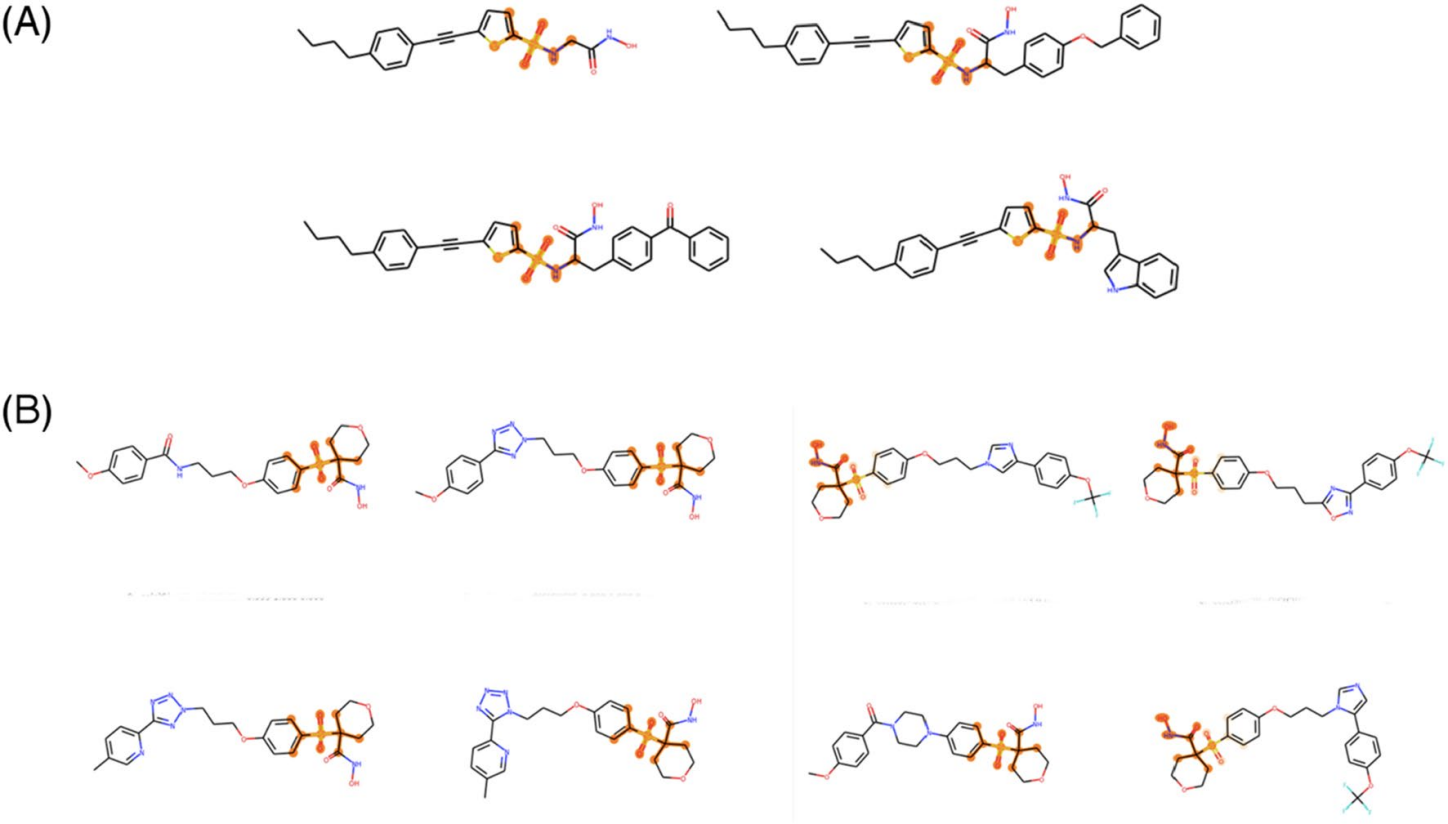
Saliency maps corresponding to MMP-3 case study. **a** Lead series from Nuti, et al. [38]. **b** Lead series from Barta, et al. [37]

Turning to our saliency maps for MMP-3, we see the sulfonamide/sulfone and hydroxamic acid groups were the most common groups identified. The saliency maps for an (ethynylthiophene) sulfonamido-based hydroxamic acid series consistently recognized the sulfonamide group but not the hydroxamic acid (Fig. 8a). The maps corresponding to a hydroxamic acid series 4-(benzenesulfonyl)-N-hydroxyoxane-4-carboxamide represented in the Pfizer publication “MMP-13 selective a-sulfone hydroxamates: A survey of P1’ heterocyclic amide isosteres” always identified the sulfonyl as key to activity and sometimes recognized the hydroxamic group as well (Fig. 8b) [37].

Together, these results suggest that sulfonamide/sulfone and hydroxamic acid signals are both present and relatively strong in our gCNN QSAR model for MMP-3. The sulfur-containing group is the dominant structure identified by the network, but hydroxamic acid groups do appear in the top five atomic neighborhoods on occasion.

#### Case study #3: Hepatitis C virus (HCV) NS5B RNA-dependent RNA polymerase (RdRp)

**Fig. 9:**
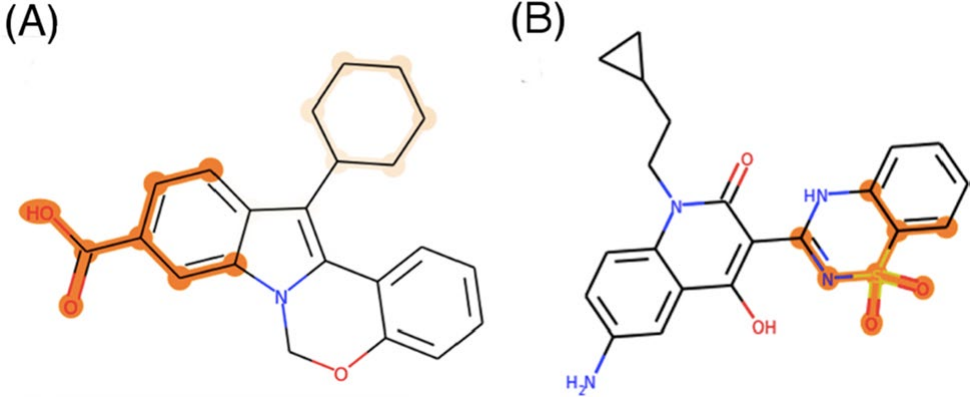
**a** Aromatic acid group and **b** benzothiadiazine group highlighted by our saliency maps in molecules that targeted HCV NS5B RdRp (Hepatitis C virus NS5B RNA-dependent RNA polymerase)

Within the ~ 150 compounds in our HCV NS5B RdRp test set, true positive saliency maps tended to highlight either an aromatic carboxylic acid group and/or a benzothiadiazine group (Fig. 9a, b). Intriguingly, two independent research groups have conducted lead optimization studies on derivatives of the structure shown in Fig. 9a. Both teams concluded that replacing the highlighted carboxylic acid group would significantly decrease the activity of the molecule [39]. Beaulieu et al. [40] further noted that such aromatic carboxylic acids could be used as a minimal core for bioactivity against HCV NS5B RdRp [39].

The benzothiadiazine motif identified in our saliency map was first proposed by GlaxoSmithKline [41] and was extensively explored by Das et al. [42] with respect to various derivatives targeting HCV NS5B RdRp. The sulfonamide subgroup, specifically highlighted by saliency map, was shown by Tedesco et al. [43] to be essential, as evidenced by a loss of activity when the sulfonamide group was replaced by a carboxamide group. It is worth mentioning that neither of these two submolecular motifs target the enzyme active site of HCV NS5B RdRp (which is typically targeted by a nucleoside analogue). Instead, these motifs inhibit the broader activity of HCV NS5B RdRp by interacting with the allosteric binding sites NNI I (targeted by aromatic acids) and NNI IV (targeted by benzothiadiazine), respectively [44]. Overall, our saliency maps were able to identify two types of molecular substructures that were deemed critical to activity against this RdRp in past experiments.

#### Case study #4: Dopamine D2 and D3 receptors

We last focus on a use case that allows us to take small molecule selectivity into account: we compare the salient features for activity against the dopamine D3 receptor (D3R) to the features associated with its close homolog, the dopamine D2 receptor. In particular, we aim to understand attempts to enhance selectivity for D3R over D2R.

**Fig. 10:**
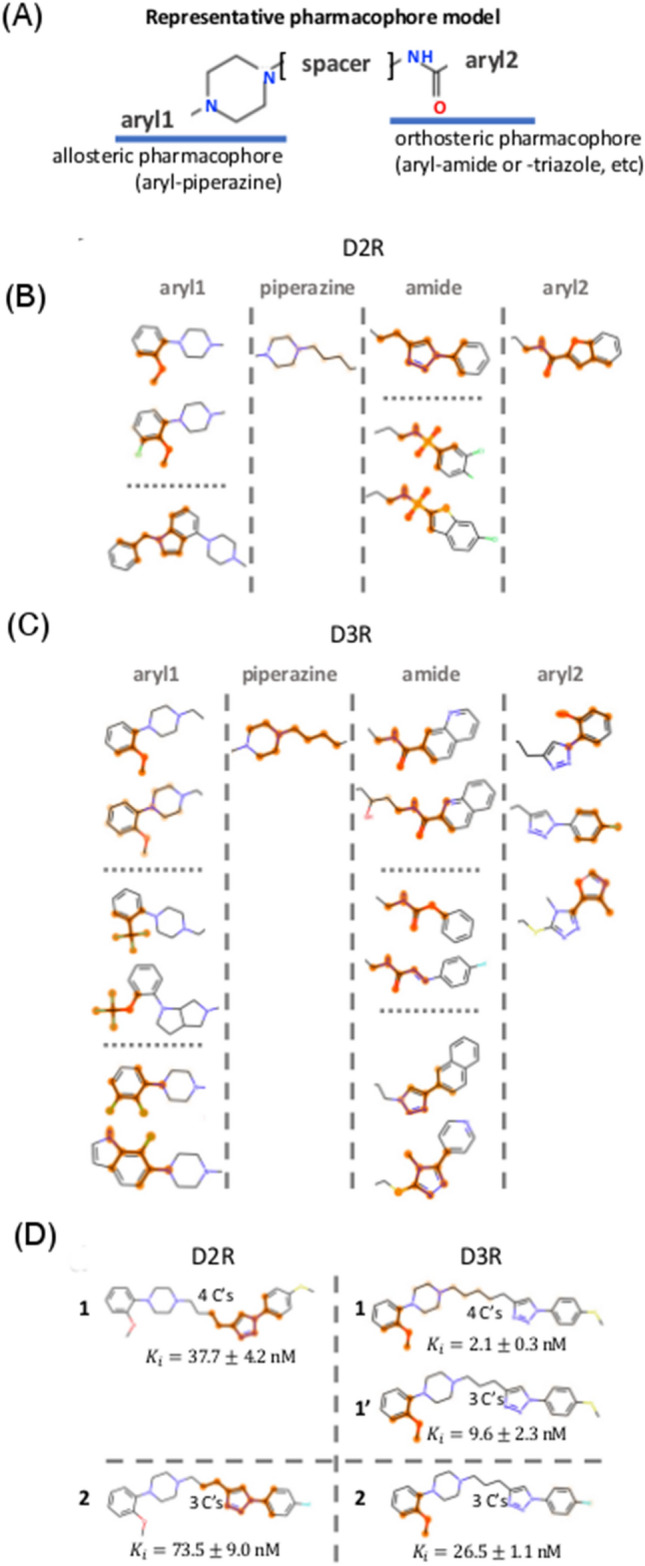
Case study on ligand selectivity with dopamine D2 and D3 receptors. **a** Representative pharmacophore models for D2R and D3R. **b**, **c** Representative substructures obtained for D2R and D3R via saliency analysis, sorted by pharmacophore identity. **d** Representative molecules showing saliency prediction differences with respect to the dopamine receptor types

Figure 10a–c illustrate representative structural motifs identified from saliency analysis on D2R and D3R. The features highlighted in our saliency clusters are further divided into two groups depending on their relative role in molecular composition, i.e., whether a substructure belongs to the core drug scaffold/skeleton (e.g., as part of a piperazine and amide (or triazole) backbone) or is a functional side-group (e.g., aryl1 and aryl2). In the course of D3R/D2R antagonist development, several skeletal elements were proposed in the construction of a consensus pharmacophore model [45], which was ultimately comprised of (1) an arylamide or aryltriazole interacting with the orthosteric binding pocket (OBP), (2) an arylpiperazine interacting with a second allosteric binding pocket (ABP), and (3) an alkyl linker (see Fig. 10a). Intriguingly, these common elements were successfully isolated by our saliency maps in many D3R and D2R ligands. Apart from these shared motifs (i.e., amide and triazole) that facilitate the OBP interaction, D2R-focused antagonists were sometimes further decorated with a sulfonamide moiety (as published in [46]), while D3R-focused antagonists were modified with a variety of motifs including triazole derivatives, carbamates, and diazenecarboxamides [47–49].

In contrast to a skeletal divergence at the orthosteric pharmacophore, the ABP appears to strongly favor an aryl-piperazine group (frequently accompanied by a butyl linker) in both D3R and D2R antagonists. Interestingly, linker length is a factor often used to control inhibitory potency. For instance, two molecules in Fig. 10d are identical apart from linker length (see 1 & 1’, with butyl and propyl linkers). In our saliency maps, the 4-carbon linker was highlighted alongside a piperazine moiety, but the 3-carbon linker was not identified as a salient feature. This result suggests the importance of linker-length for optimizing potency (experiments confirmed that the former molecule was indeed more potent than the latter: ${K}_{i}=2.1\pm 0.3 \mathrm{nM}$ vs. $9.6\pm 2.3 \mathrm{nM}$ [48].

The importance of the piperazine-based allosteric pharmacophore is implied by the range of aryl moieties that appear in our saliency clusters, especially for D3R-specific antagonists. In practice, our D2R/D3R saliency maps highlighted different substructures for identical molecules as a function of receptor type. For example, the orthosteric pharmacophore centered on the triazole moiety appeared to be important D2R antagonism (see 1 and 2 in Fig. 10d), but the allosteric end centered on a phenyl hydroxyl group was highlighted in our D3R saliency model. In this case, both molecules were more potent inhibitors of D3R than D2R: **1**, ${K}_{i}=2.1\pm 0.3 \mathrm{nM}$ for D3R vs. $37.7\pm 4.2 \mathrm{nM}$ for D2R and **2**, ${K}_{i}=26.5\pm 1.1 \mathrm{nM}$ for D3R vs. $73.5\pm 9.0 \mathrm{nM}$ for D2R [48]. This saliency distinction is interesting considering that the orthosteric binding pocket, to which dopamine binds, remains conserved for both receptors, thus making it difficult to modulate ligand selectivity. However, the allosteric pockets are found to be distinct enough to allow for selective ligand design. Though additional analysis on common antogonists is warranted, the relatively high saliency weights on aryl derivatives of the allosteric pharmacophore for D3R seem to give insights into both ligand activity and specificity in this system.

## Conclusion

We have shown that our simplified, interpretable gCNN architecture improves average activity prediction on test sets most separated from our training data and provides insights into substructure-activity relationships that might assist in small molecular design for specific protein targets. When classifying “less difficult” test sets (i.e., test sets with higher mean similarity to the training set), our architecture achieves results roughly equivalent to standard RF and gCNN benchmarks. We integrate this simplified model with a saliency map technique that highlights molecular substructures relevant to activity. While our current cluster-based visualization scheme for saliency maps is imperfect, we do see that, in general, our method groups similar highlighted substructures to an extent that should help end users see qualitative activity trends across a target’s labeled molecules.

A limitation of the present analysis concerns a restriction we made for the conciseness of this paper: we focus our saliency discussion exclusively on test molecules that were true positives within their respective QSAR models. Similar substructural clusters can be trivially generated for true negatives, false negatives, and false positives. The subsequent analysis of those subclasses would be more complex, as one would likely want to cross-reference substructures between categories to determine which subgroups contribute to activity, detract from activity, need to be added to impart activity, or even confound our gCNN architecture as it currently stands. Interesting work has recently been published on identifying specific features whose absence is associated with molecular activity [50], and similar ideas certainly apply to the explainability of our gCNN architecture. A richer analysis of our gCNN confusion matrix categories that allows one to access negative and confounding information will likely be the subject of future work on a less expansive target set.

The saliency analysis presented in this work has potential utility for in silico molecular modeling teams. The usefulness of adding substructure-based interpretability to virtual screening applications is perhaps self-evident, as highlighted substructures could help identify connections between specific functional groups and activity and guide further design. One could also consider integrating a saliency map into a generative small molecule workflow, either through curating training data according to desired highlighted subgroups or even conditioning the training process on a particular set of molecular substructures.

Combining saliency maps with other modern machine learning interpretability tools (e.g. attention mechanisms [23–25], attribution mechanisms [51], Monte Carlo tree search [52, 53], etc.) could be advantageous in a number of ways. As previously discussed, implementation of attention mechanisms [23–25] requires specific modifications to a gCNN architecture of interest; particularly if one can demonstrate a predictive performance improvement via the addition of attention weights, one might benefit from comparing results from saliency maps and attention mechanisms. Secondly, while we have shown saliency maps can identify molecular substructures that are correlated with activity prediction, the ultimate interpretation of “why” those substructures are selected still falls to human experts. Though some substructures identified via saliency indeed correspond to important intermolecular interactions within the protein binding site, others undoubtedly relate to general small molecule properties like solubility or even correlate with spurious connections between data points. One could potentially leverage mechanisms for substructural attribution, which attempt to identify the specific sources of correlations [51], to help automate more quantitative interpretations of saliency maps. Furthermore, details of substructural properties obtained through saliency analysis can again possibly aid molecule generation techniques, including those based on gCNN or Monte Carlo tree search methodologies [52, 53].

As previously noted, saliency predictions focused on subgroups responsible for activity within a given lead series might tend to be obvious to medicinal chemists working closely on that lead series. However, we would expect our method to be more useful to end users when applied to understanding less familiar lead series, broader lead finding applications with diverse screening data, or off-target activity trends. Furthermore, saliency maps are certainly not restricted to activity labels, and instead could be trained on selectivity data, generic toxicity data, metabolism data, downstream biological activity data, etc. One could imagine working closely with medicinal chemists to identify which properties and predictions would be most helpful in the development of lead series. Regardless, capturing the substructural motifs most relevant to activity represents an important first step for automated, deep learning-driven saliency analysis, and we feel we have reached that milestone in this work.

## Supplementary Information

Below is the link to the electronic supplementary material.

Supplementary file1 (XLTX 44 KB)

Supplementary file2 (DOCX 497 KB)

Supplementary file3 (DOCX 497 KB)

Supplementary file4 (ZIP 111545 KB)

## Data Availability

The saliency map clusters generated in the current study for the true positive clusters corresponding to our data-rich/high-performing QSAR models have been provided in structured directories with the top level directory names corresponding to Chembl target numbers (see translation key in the Supplementary Information). True positive molecules are printed four per page for each cluster in each numbered directory.
