# Supplementary material for: Simplified, interpretable graph convolutional neural networks for small molecule activity prediction
Source: J Comput Aided Mol Des. 2021 Nov 24;36(5):391–404. doi: 10.1007/s10822-021-00421-6 (PMC9325818; doi:10.1007/s10822-021-00421-6)

Supplementary Information: Simplified, Interpretable Graph Convolutional Neural Networks for Small Molecule Activity Prediction

*Guide to Saliency Results on Nearly 100 Targets*

Saliency map clusters for the true positive clusters corresponding to our data-rich/high-performing QSAR models have been provided in structured directories with the top level directory names corresponding to Chembl target numbers (see translation key below).

True positive molecules are printed four per page for each cluster in each numbered directory. These results files are offered as supplementary information and for review purposes are currently accessible via the BOX link

<https://ibm.ent.box.com/folder/140782081369>


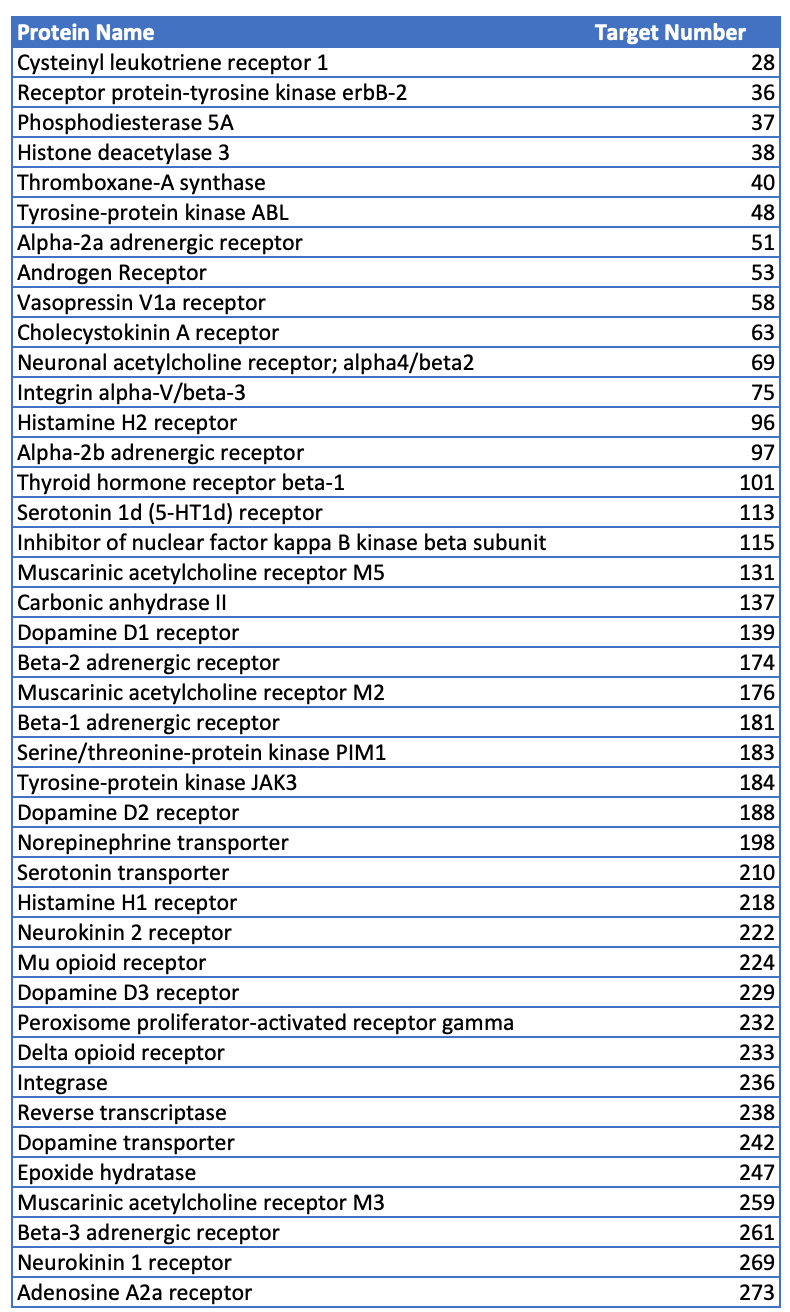

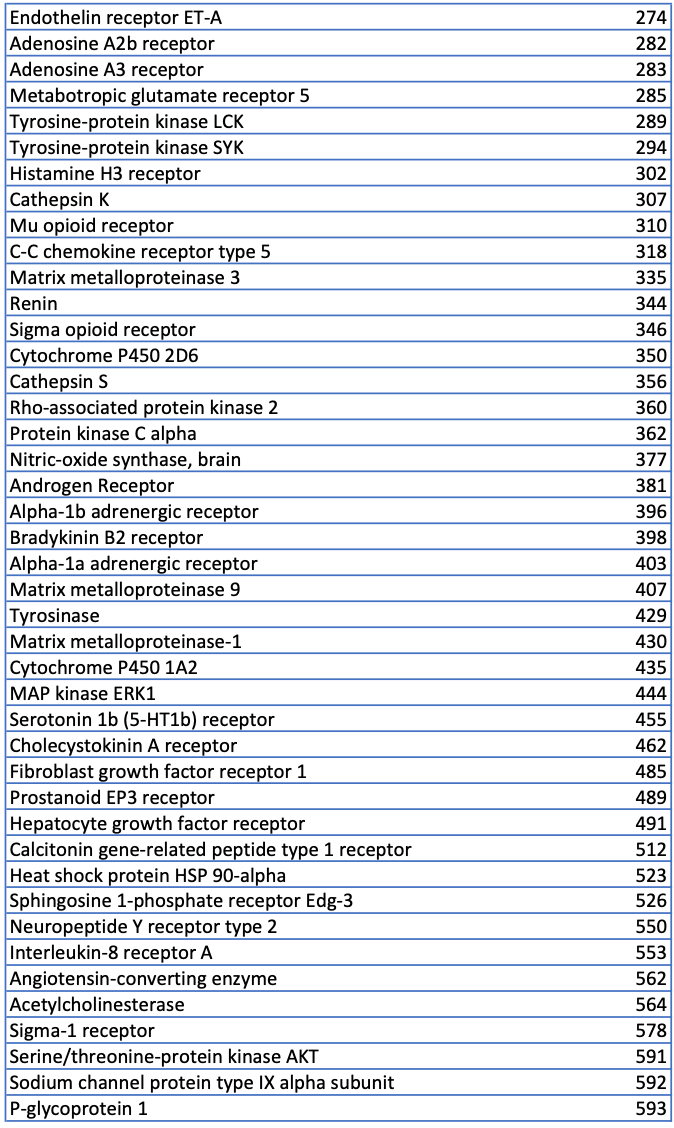


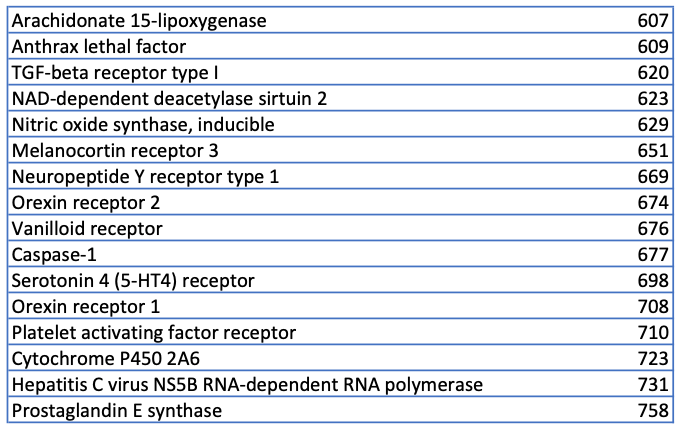

Supplement: Supplementary file 2 — Supplementary file2 (DOCX 497 KB) [file 10822_2021_421_MOESM2_ESM.docx]
