# Supplementary figures and images for: Simplified, interpretable graph convolutional neural networks for small molecule activity prediction (part 2 of 2)
Source: J Comput Aided Mol Des. 2021 Nov 24;36(5):391–404. doi: 10.1007/s10822-021-00421-6 (PMC9325818; doi:10.1007/s10822-021-00421-6)

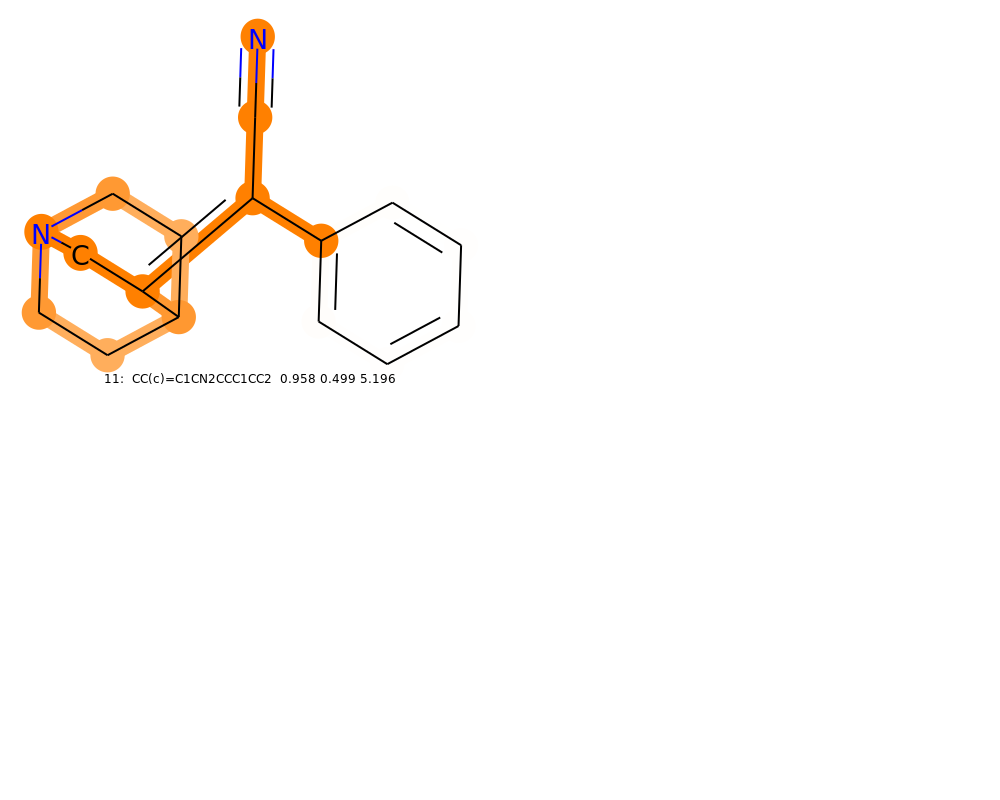

Supplement: Supplementary file 4 — Supplementary file4 (ZIP 111545 KB) [file 10822_2021_421_MOESM4_ESM.zip › 069/tp_cluster_11/tp11_mols_4.png]

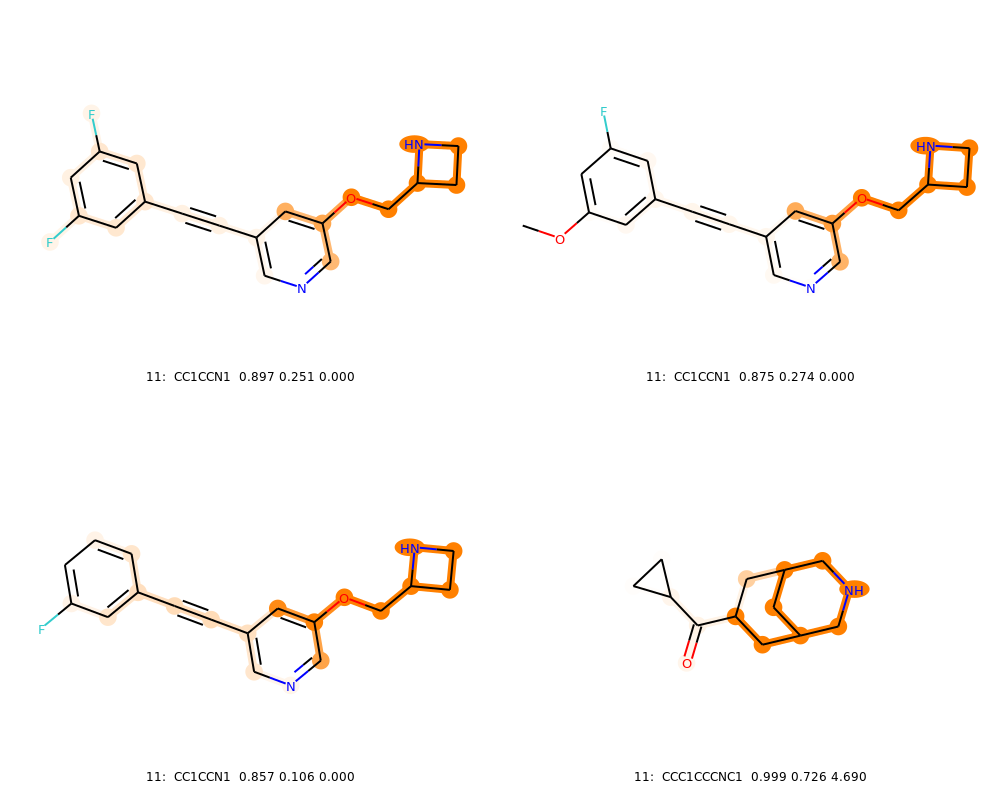

Supplement: Supplementary file 4 — Supplementary file4 (ZIP 111545 KB) [file 10822_2021_421_MOESM4_ESM.zip › 069/tp_cluster_11/tp11_mols_0.png]

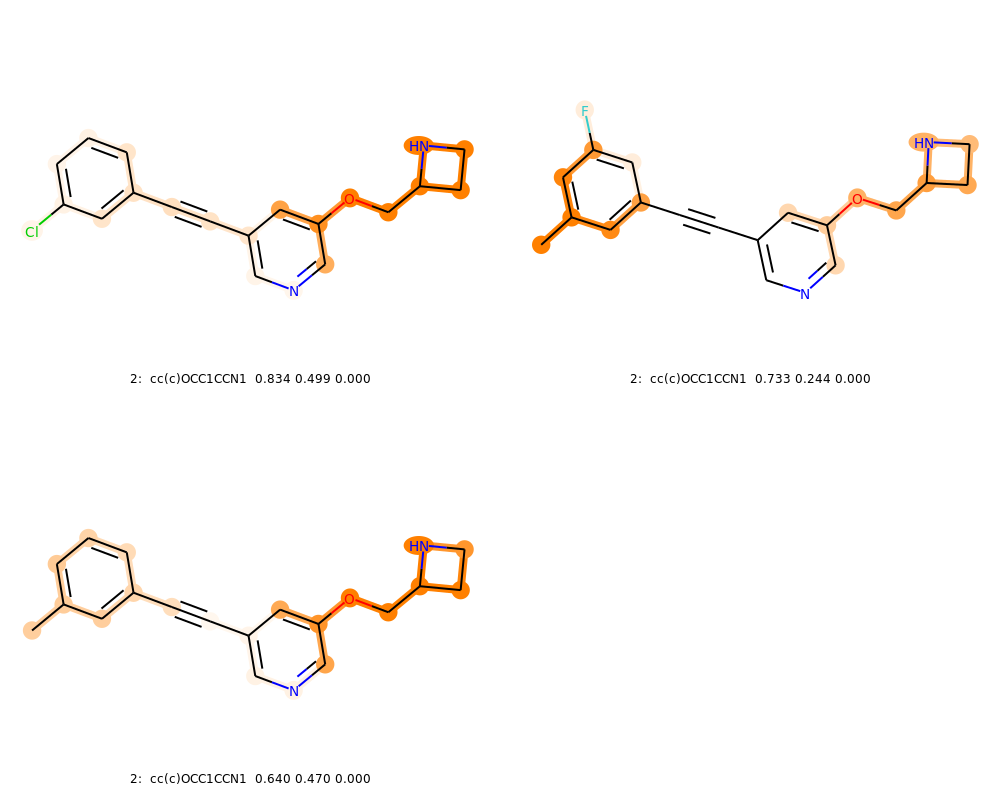

Supplement: Supplementary file 4 — Supplementary file4 (ZIP 111545 KB) [file 10822_2021_421_MOESM4_ESM.zip › 069/tp_cluster_2/tp2_mols_4.png]

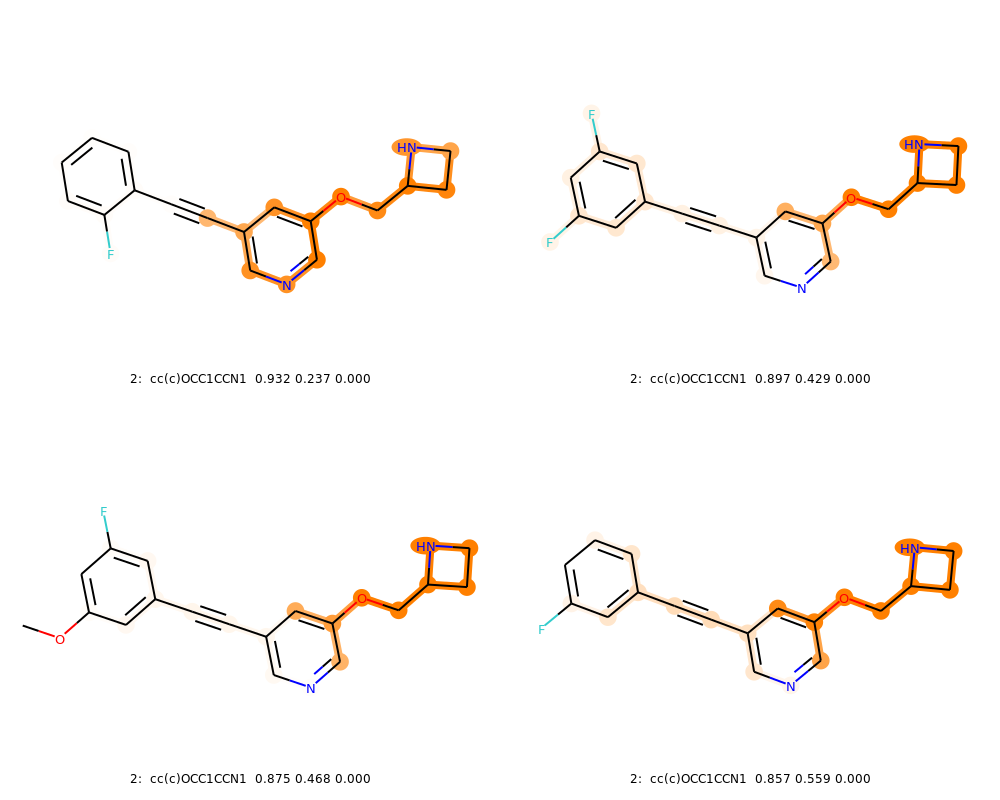

Supplement: Supplementary file 4 — Supplementary file4 (ZIP 111545 KB) [file 10822_2021_421_MOESM4_ESM.zip › 069/tp_cluster_2/tp2_mols_0.png]

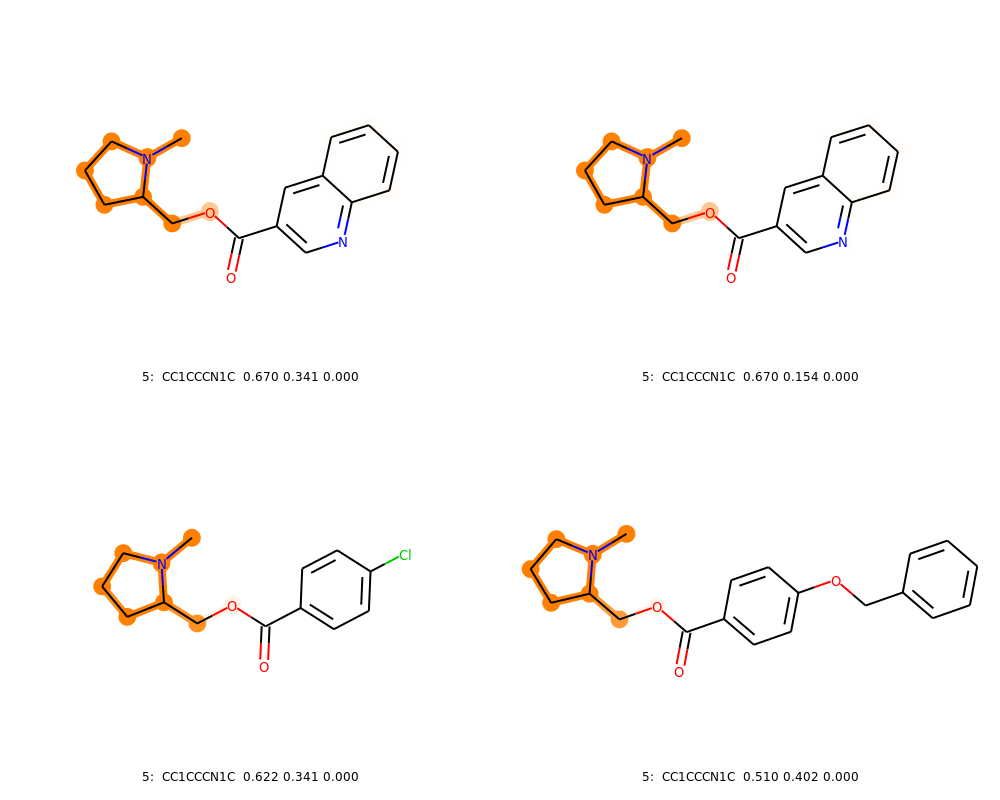

Supplement: Supplementary file 4 — Supplementary file4 (ZIP 111545 KB) [file 10822_2021_421_MOESM4_ESM.zip › 069/tp_cluster_5/tp5_mols_0.png]

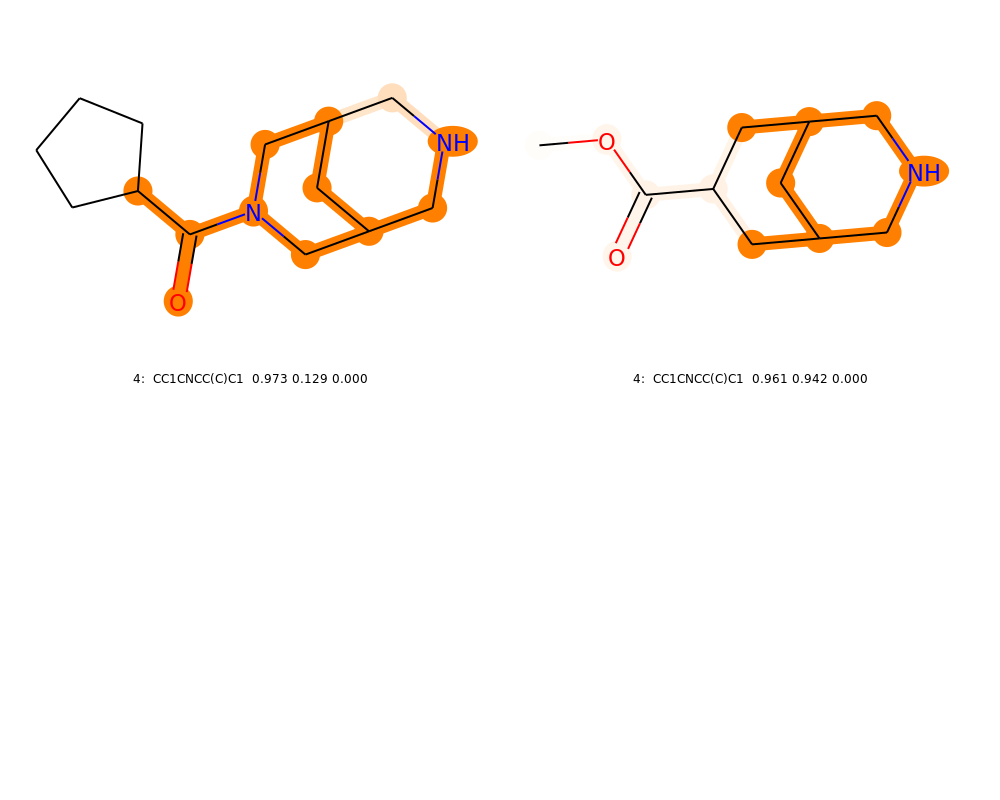

Supplement: Supplementary file 4 — Supplementary file4 (ZIP 111545 KB) [file 10822_2021_421_MOESM4_ESM.zip › 069/tp_cluster_4/tp4_mols_4.png]

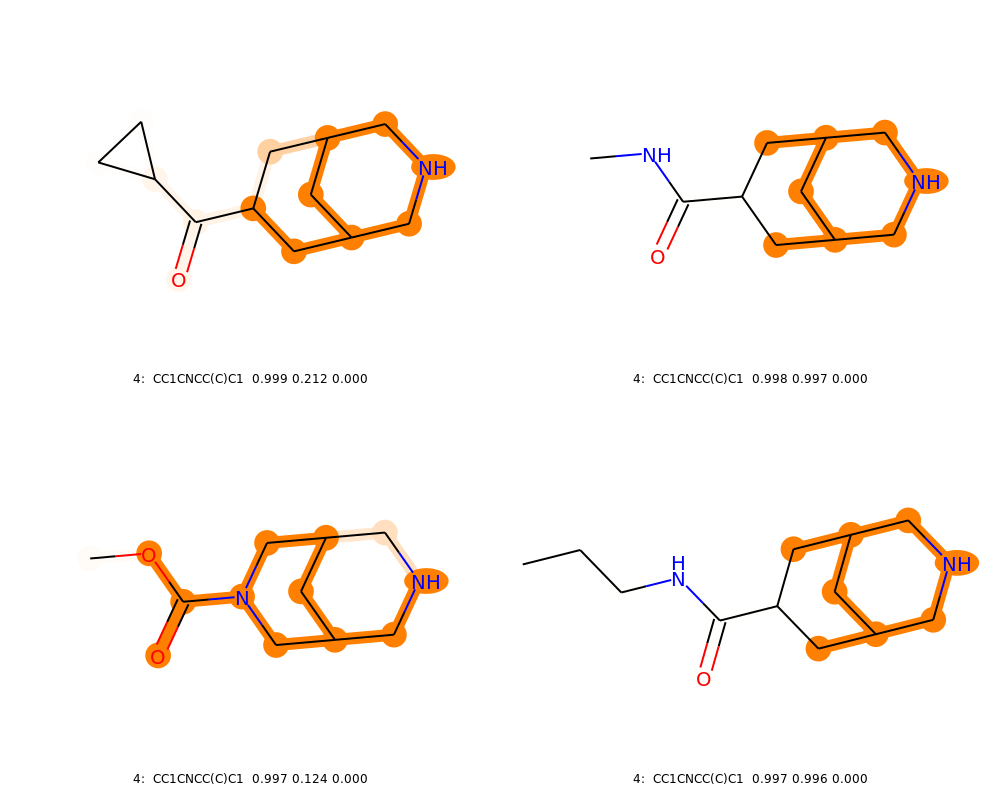

Supplement: Supplementary file 4 — Supplementary file4 (ZIP 111545 KB) [file 10822_2021_421_MOESM4_ESM.zip › 069/tp_cluster_4/tp4_mols_0.png]

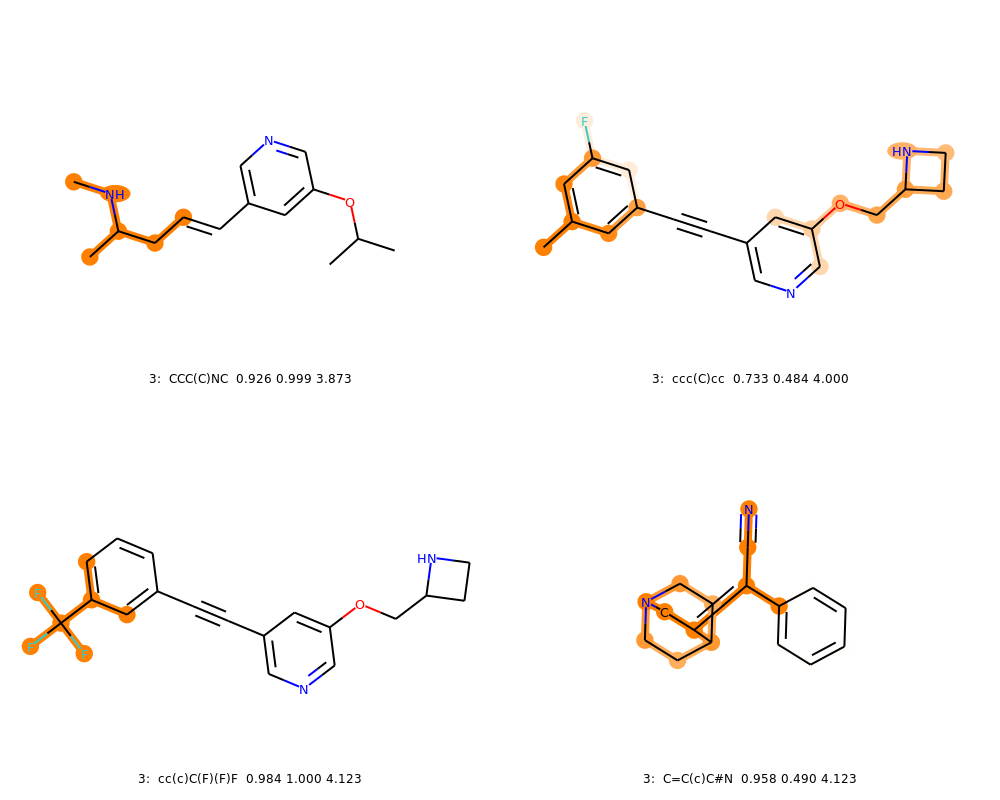

Supplement: Supplementary file 4 — Supplementary file4 (ZIP 111545 KB) [file 10822_2021_421_MOESM4_ESM.zip › 069/tp_cluster_3/tp3_mols_8.png]

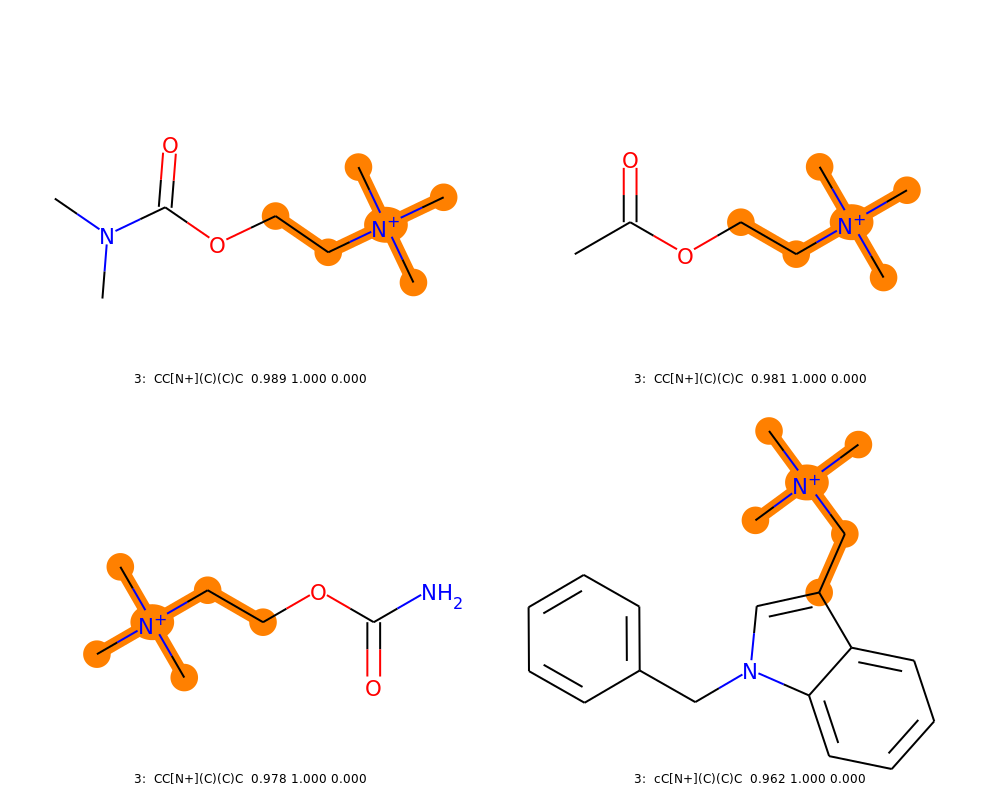

Supplement: Supplementary file 4 — Supplementary file4 (ZIP 111545 KB) [file 10822_2021_421_MOESM4_ESM.zip › 069/tp_cluster_3/tp3_mols_0.png]

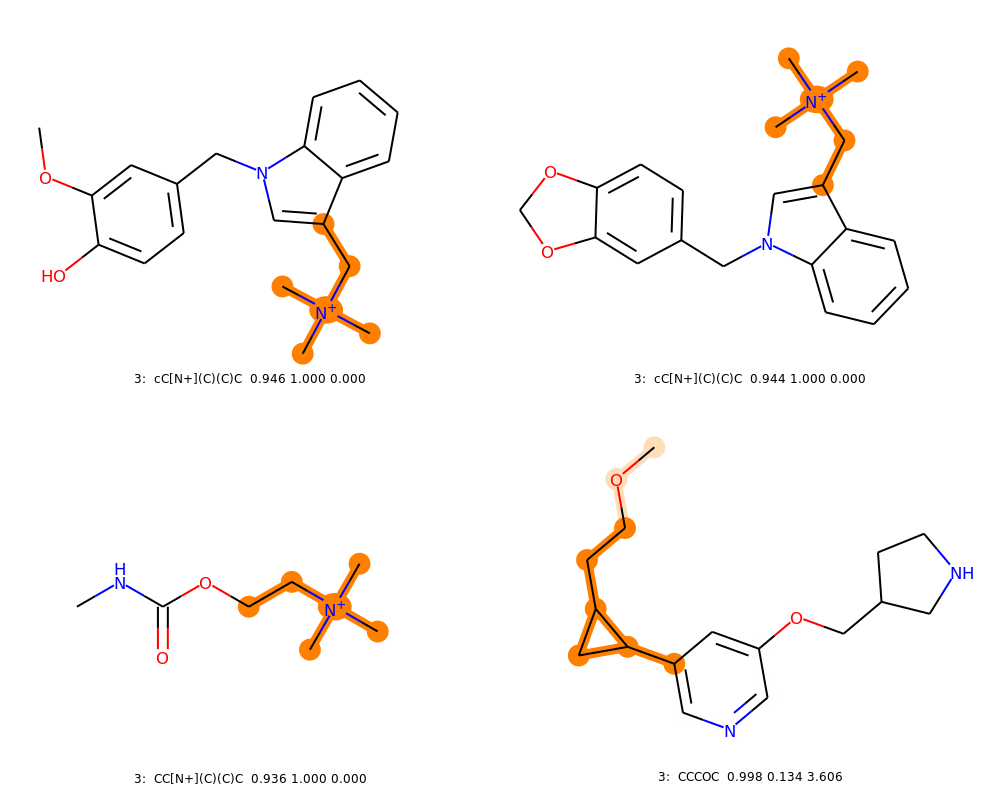

Supplement: Supplementary file 4 — Supplementary file4 (ZIP 111545 KB) [file 10822_2021_421_MOESM4_ESM.zip › 069/tp_cluster_3/tp3_mols_4.png]

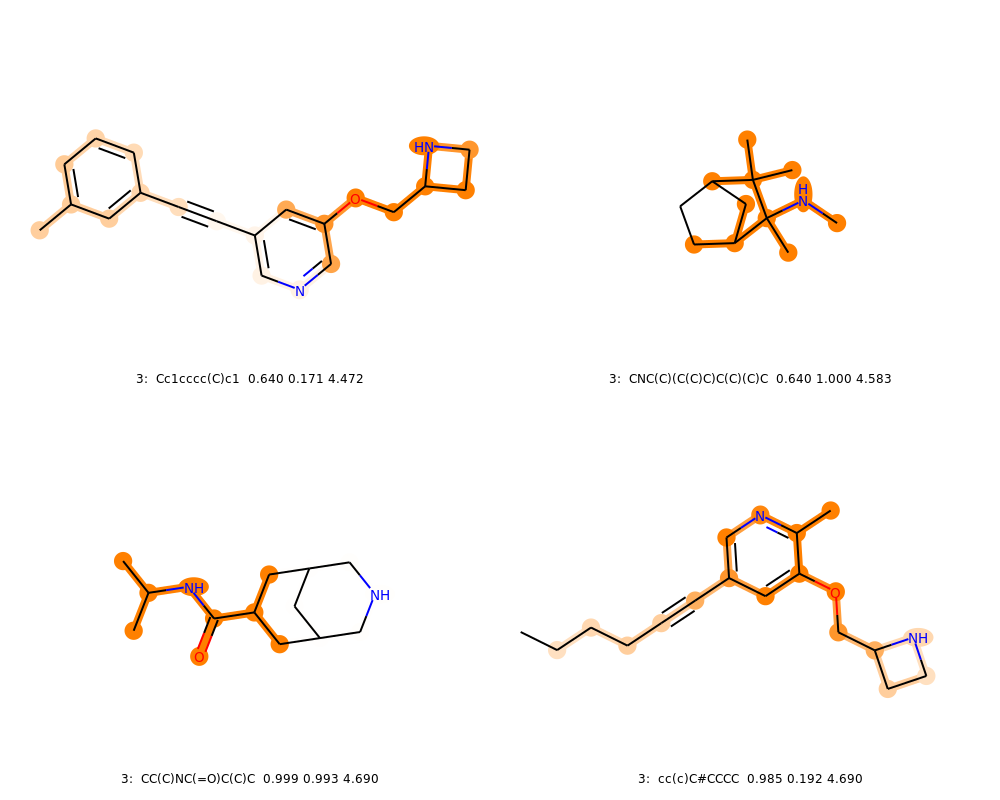

Supplement: Supplementary file 4 — Supplementary file4 (ZIP 111545 KB) [file 10822_2021_421_MOESM4_ESM.zip › 069/tp_cluster_3/tp3_mols_12.png]

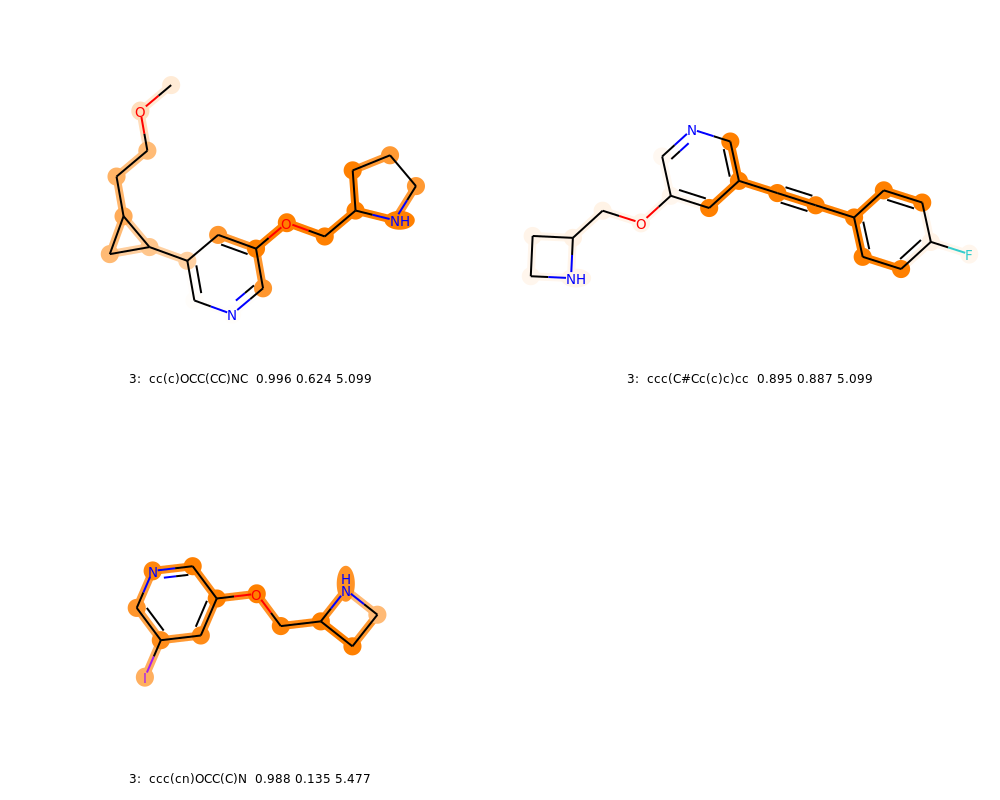

Supplement: Supplementary file 4 — Supplementary file4 (ZIP 111545 KB) [file 10822_2021_421_MOESM4_ESM.zip › 069/tp_cluster_3/tp3_mols_16.png]

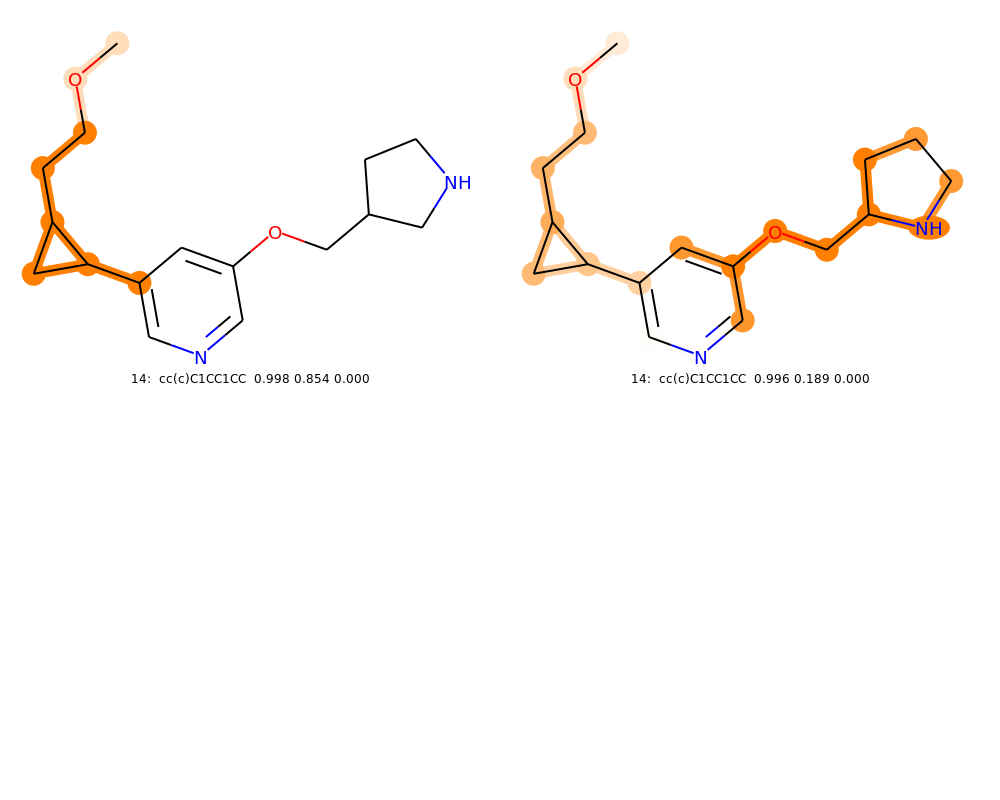

Supplement: Supplementary file 4 — Supplementary file4 (ZIP 111545 KB) [file 10822_2021_421_MOESM4_ESM.zip › 069/tp_cluster_14/tp14_mols_0.png]

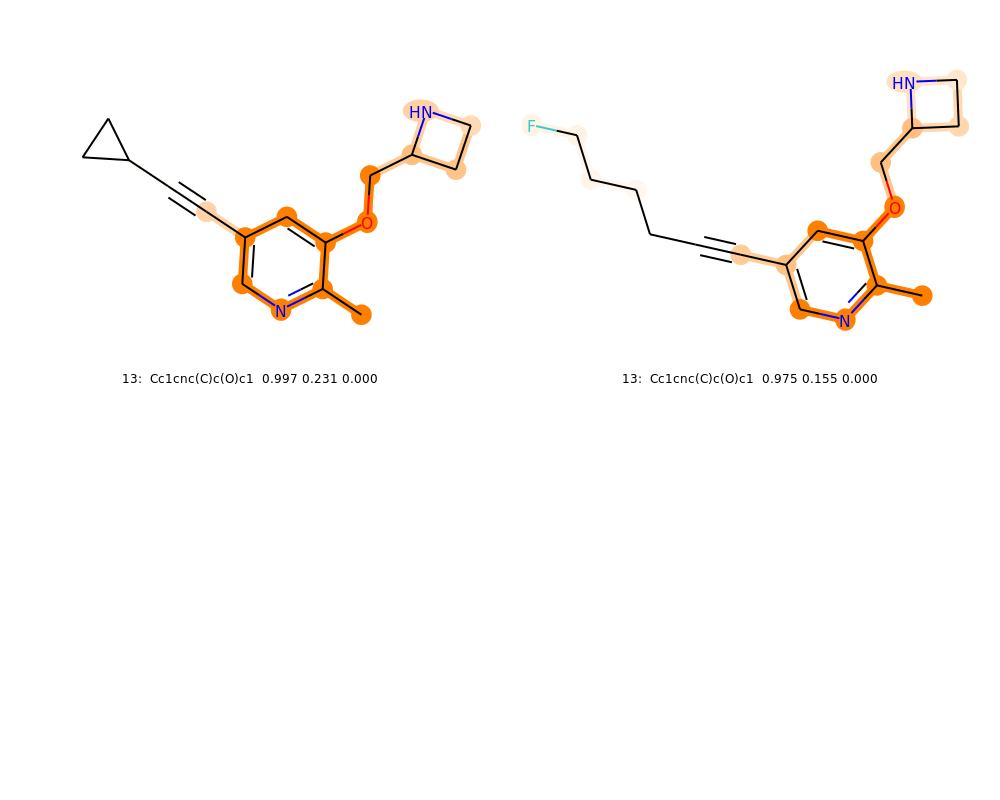

Supplement: Supplementary file 4 — Supplementary file4 (ZIP 111545 KB) [file 10822_2021_421_MOESM4_ESM.zip › 069/tp_cluster_13/tp13_mols_0.png]

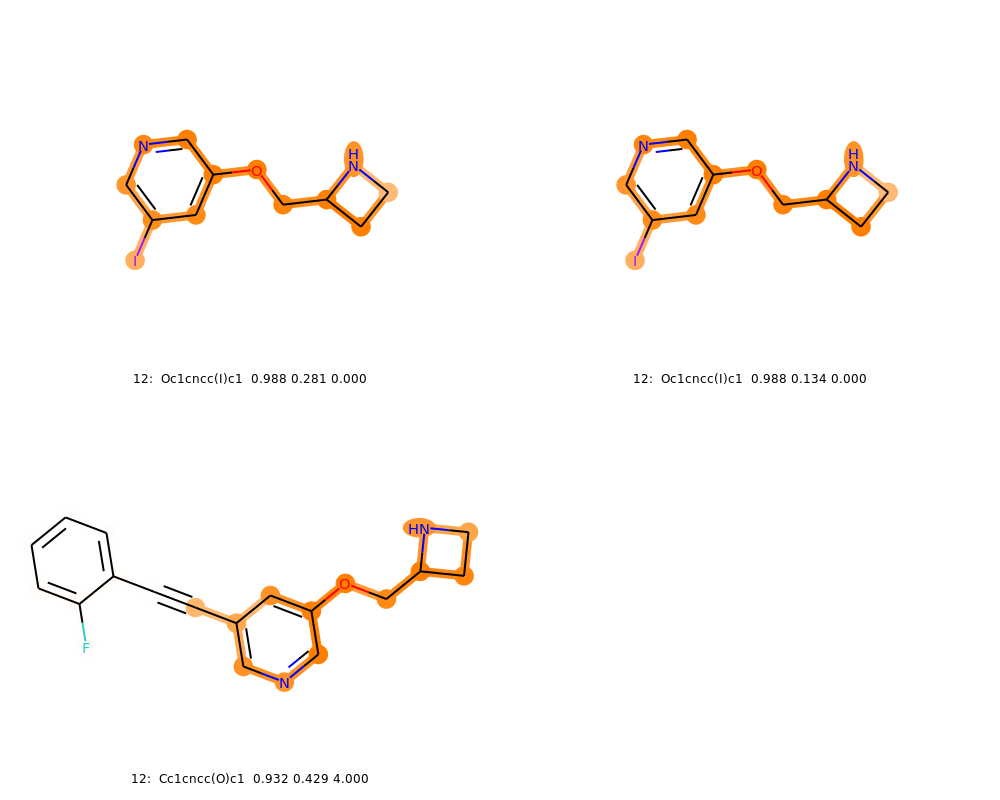

Supplement: Supplementary file 4 — Supplementary file4 (ZIP 111545 KB) [file 10822_2021_421_MOESM4_ESM.zip › 069/tp_cluster_12/tp12_mols_0.png]

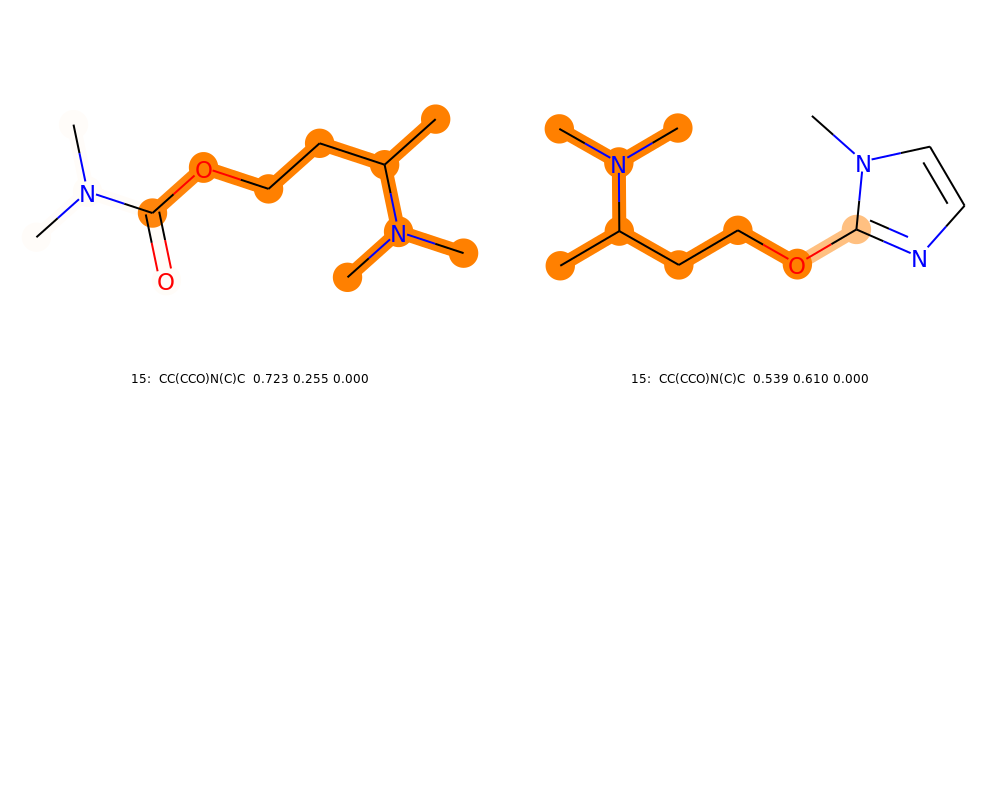

Supplement: Supplementary file 4 — Supplementary file4 (ZIP 111545 KB) [file 10822_2021_421_MOESM4_ESM.zip › 069/tp_cluster_15/tp15_mols_0.png]

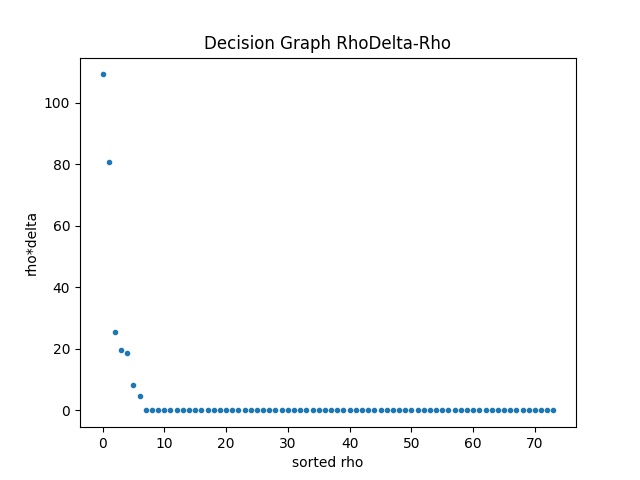

Supplement: Supplementary file 4 — Supplementary file4 (ZIP 111545 KB) [file 10822_2021_421_MOESM4_ESM.zip › 075/tp_decision_graphs/RhoDelta-Rho.jpg]

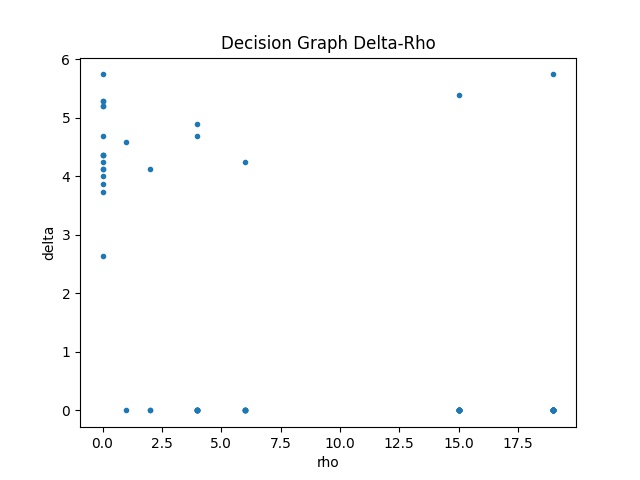

Supplement: Supplementary file 4 — Supplementary file4 (ZIP 111545 KB) [file 10822_2021_421_MOESM4_ESM.zip › 075/tp_decision_graphs/Delta-Rho.jpg]

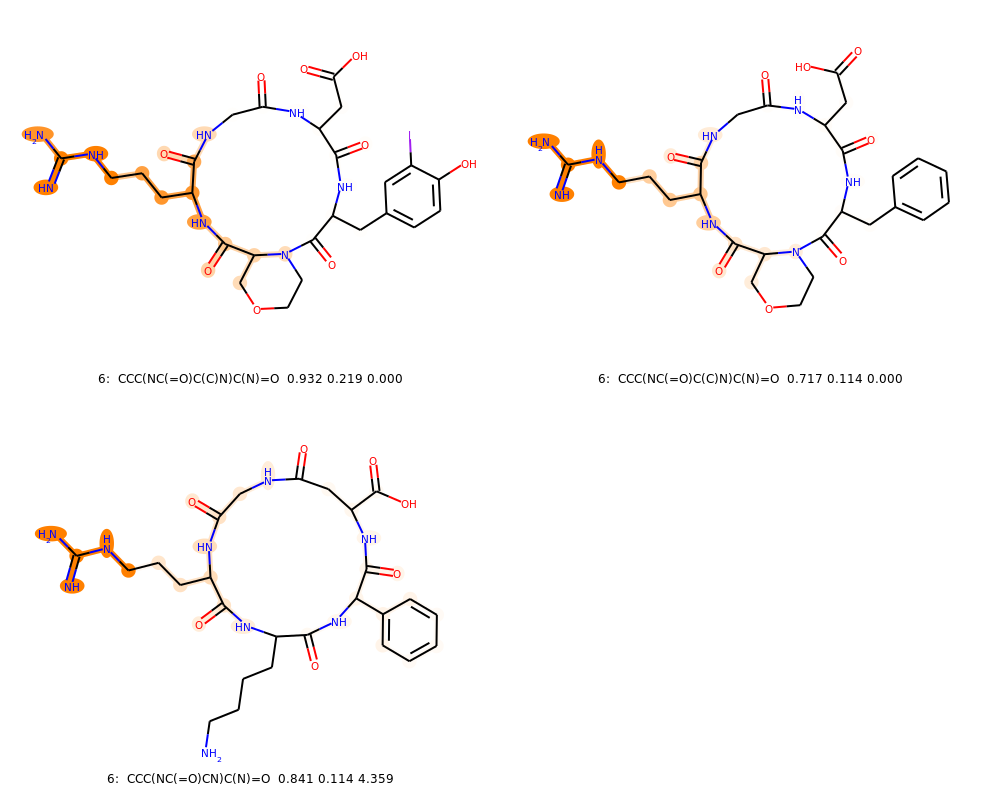

Supplement: Supplementary file 4 — Supplementary file4 (ZIP 111545 KB) [file 10822_2021_421_MOESM4_ESM.zip › 075/tp_cluster_6/tp6_mols_0.png]

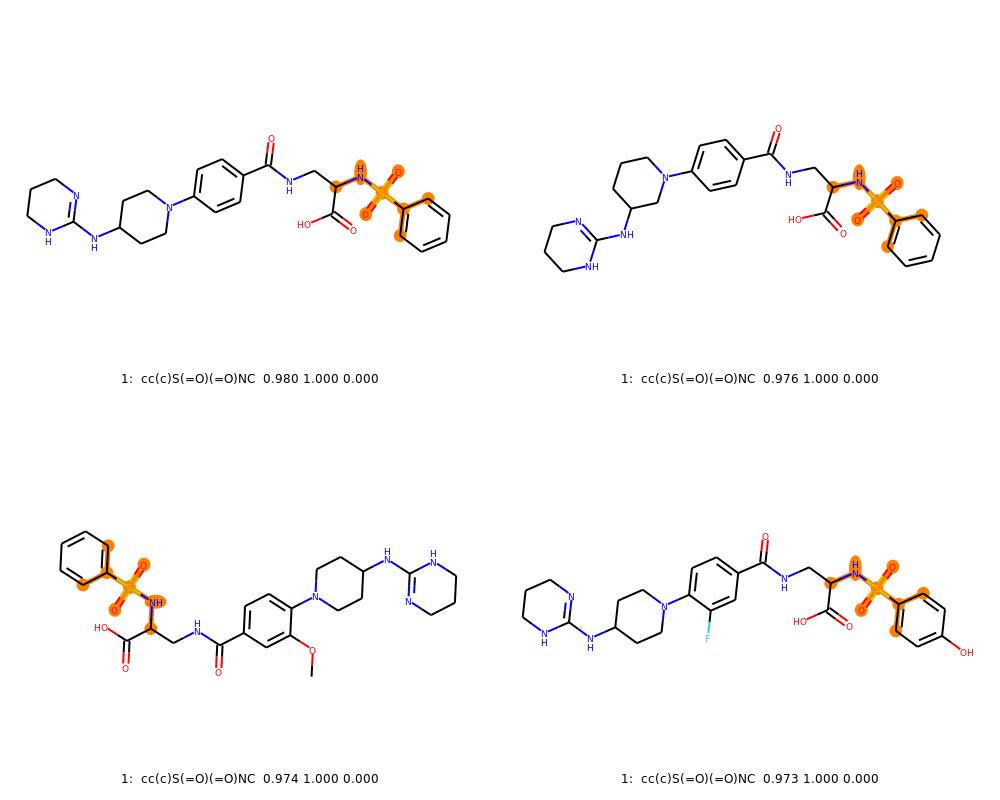

Supplement: Supplementary file 4 — Supplementary file4 (ZIP 111545 KB) [file 10822_2021_421_MOESM4_ESM.zip › 075/tp_cluster_1/tp1_mols_8.png]

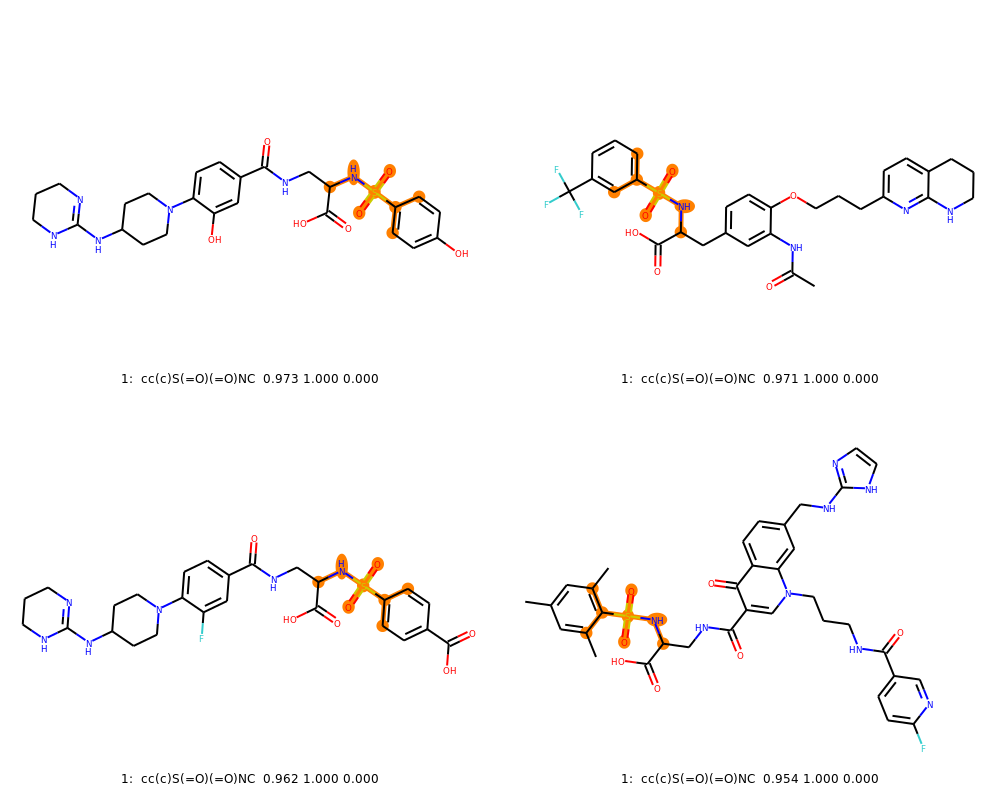

Supplement: Supplementary file 4 — Supplementary file4 (ZIP 111545 KB) [file 10822_2021_421_MOESM4_ESM.zip › 075/tp_cluster_1/tp1_mols_12.png]

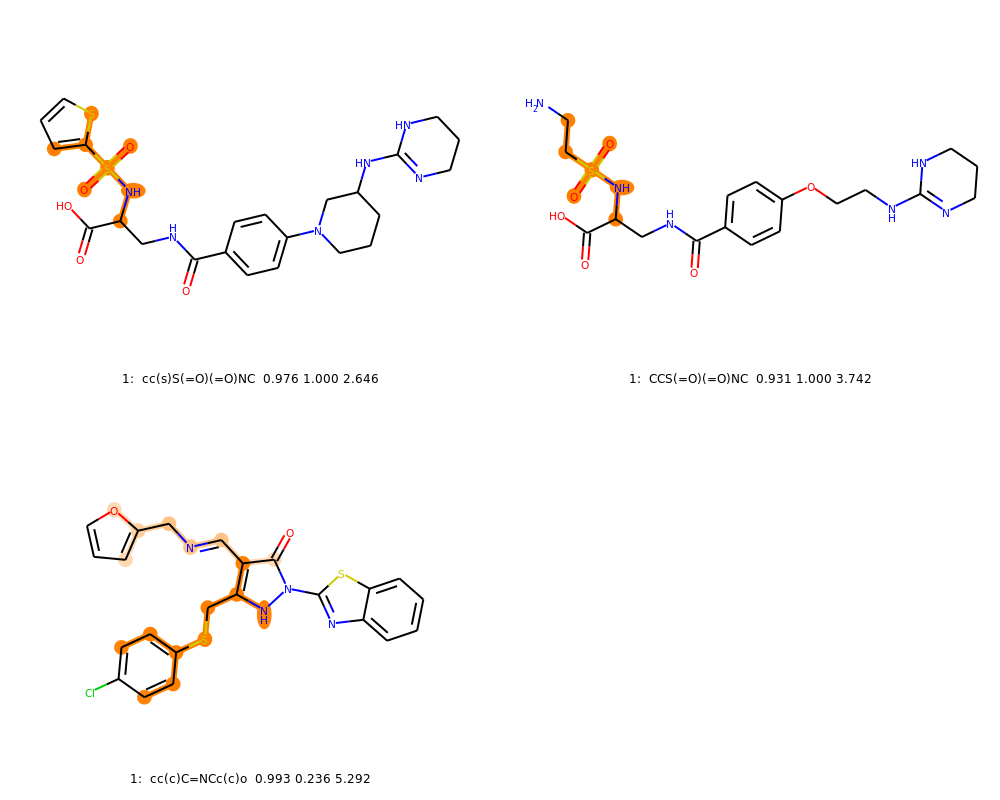

Supplement: Supplementary file 4 — Supplementary file4 (ZIP 111545 KB) [file 10822_2021_421_MOESM4_ESM.zip › 075/tp_cluster_1/tp1_mols_16.png]

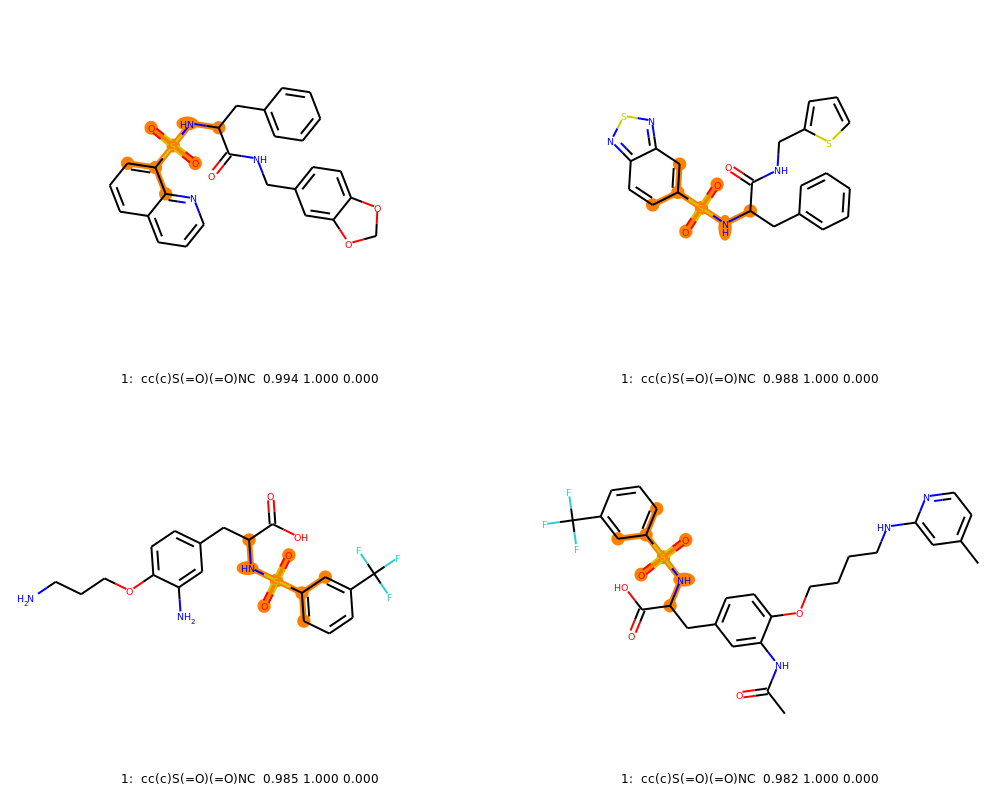

Supplement: Supplementary file 4 — Supplementary file4 (ZIP 111545 KB) [file 10822_2021_421_MOESM4_ESM.zip › 075/tp_cluster_1/tp1_mols_0.png]

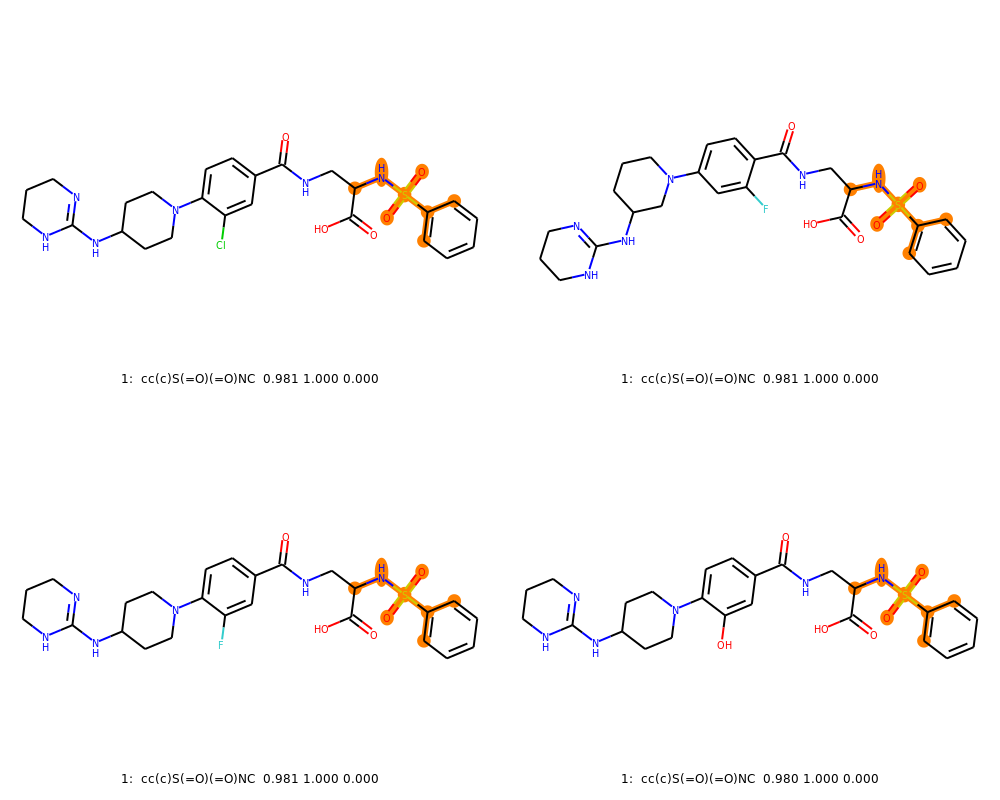

Supplement: Supplementary file 4 — Supplementary file4 (ZIP 111545 KB) [file 10822_2021_421_MOESM4_ESM.zip › 075/tp_cluster_1/tp1_mols_4.png]

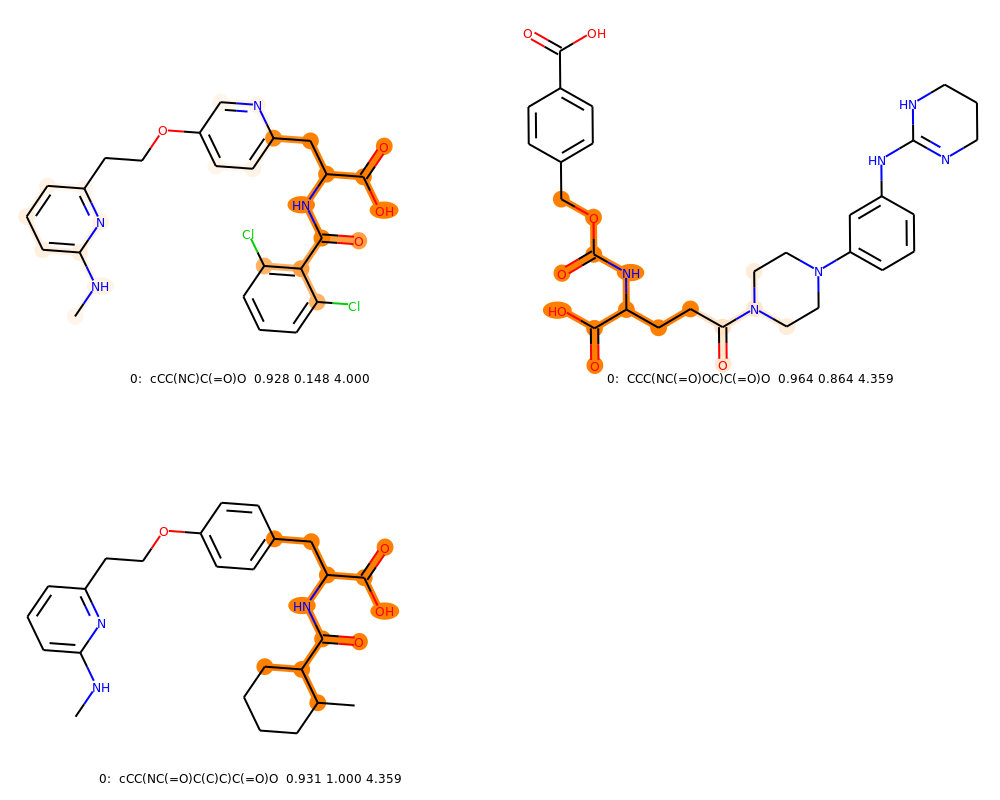

Supplement: Supplementary file 4 — Supplementary file4 (ZIP 111545 KB) [file 10822_2021_421_MOESM4_ESM.zip › 075/tp_cluster_0/tp0_mols_20.png]

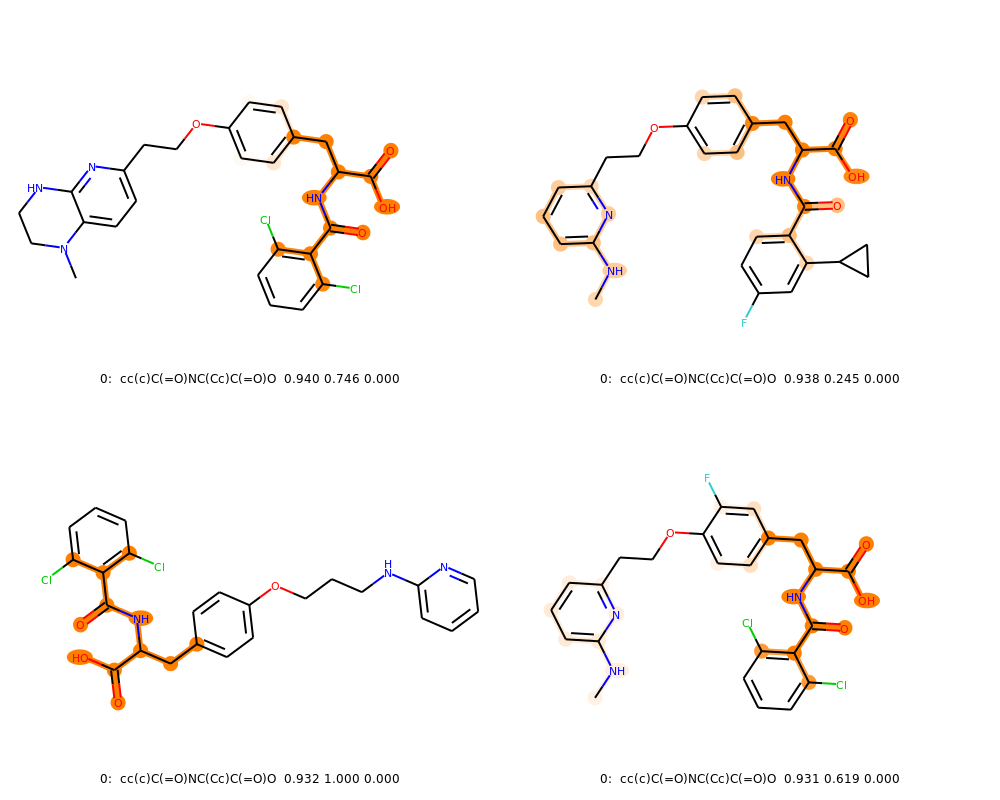

Supplement: Supplementary file 4 — Supplementary file4 (ZIP 111545 KB) [file 10822_2021_421_MOESM4_ESM.zip › 075/tp_cluster_0/tp0_mols_4.png]

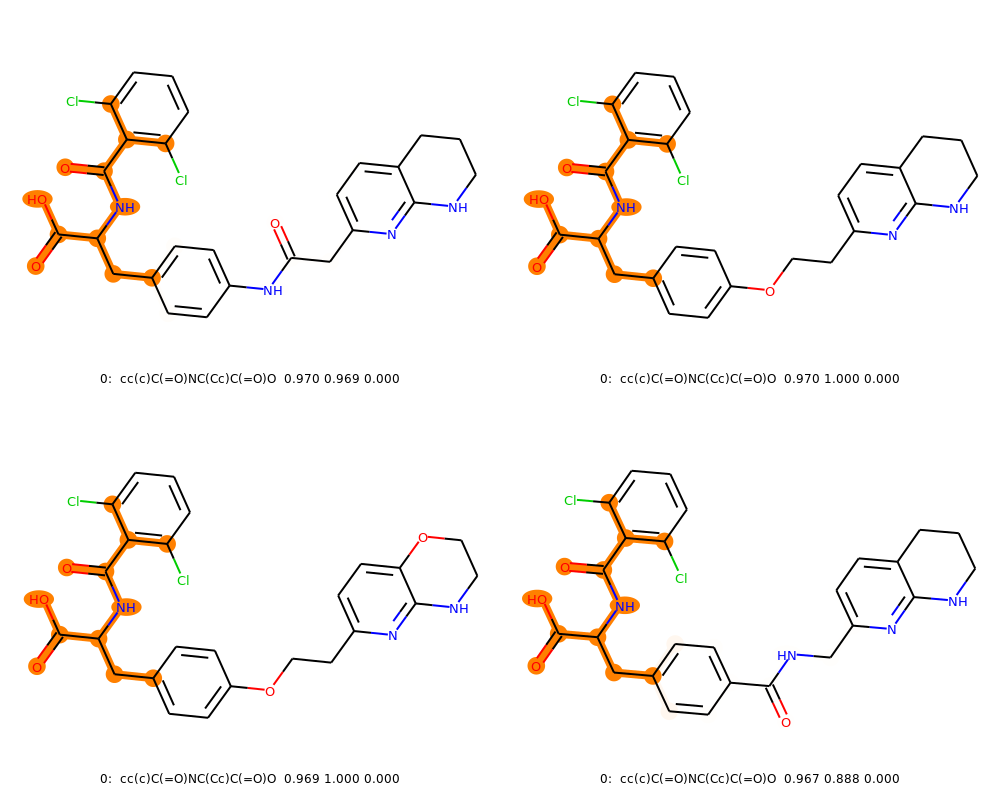

Supplement: Supplementary file 4 — Supplementary file4 (ZIP 111545 KB) [file 10822_2021_421_MOESM4_ESM.zip › 075/tp_cluster_0/tp0_mols_0.png]

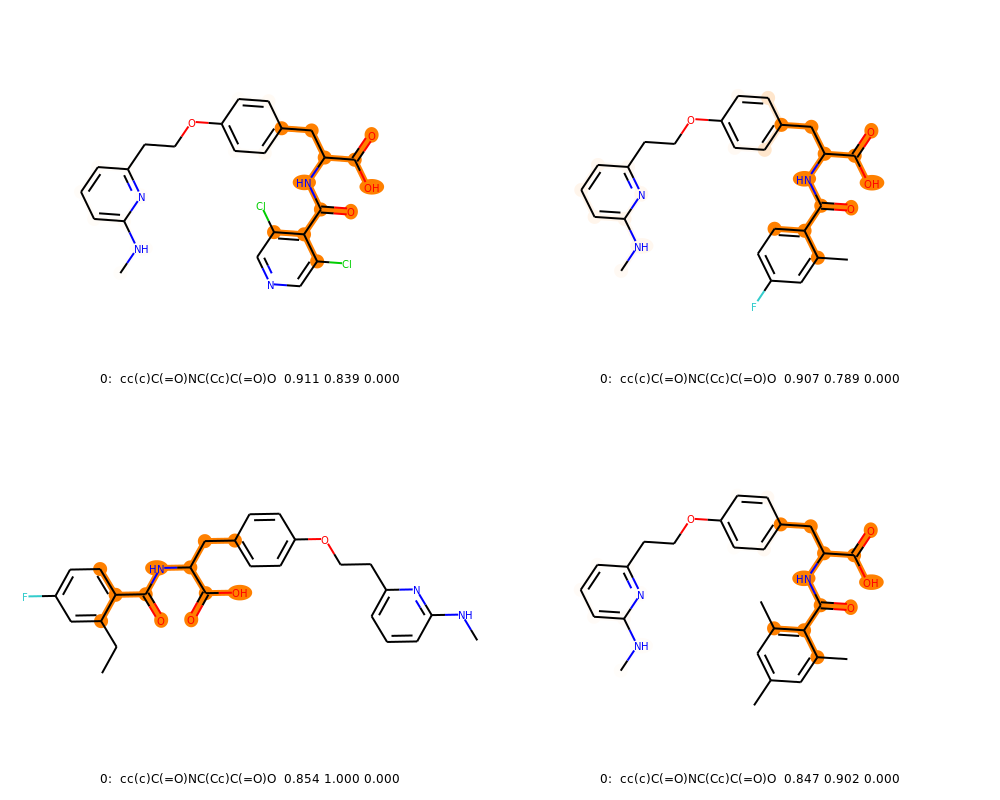

Supplement: Supplementary file 4 — Supplementary file4 (ZIP 111545 KB) [file 10822_2021_421_MOESM4_ESM.zip › 075/tp_cluster_0/tp0_mols_16.png]

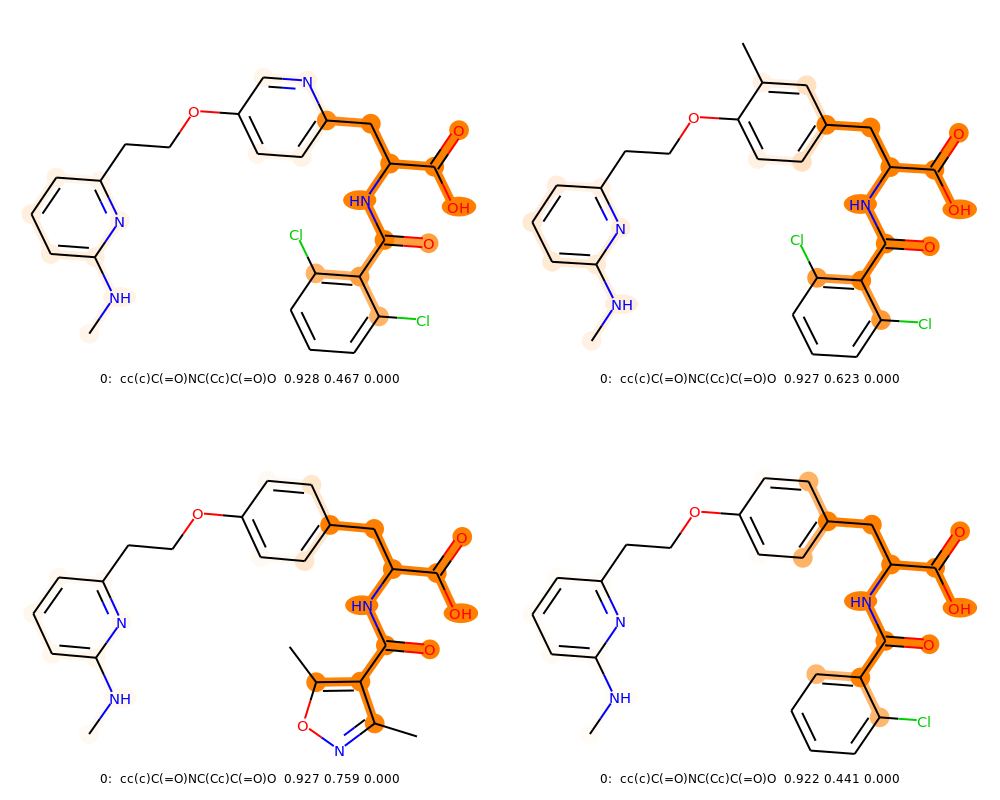

Supplement: Supplementary file 4 — Supplementary file4 (ZIP 111545 KB) [file 10822_2021_421_MOESM4_ESM.zip › 075/tp_cluster_0/tp0_mols_8.png]

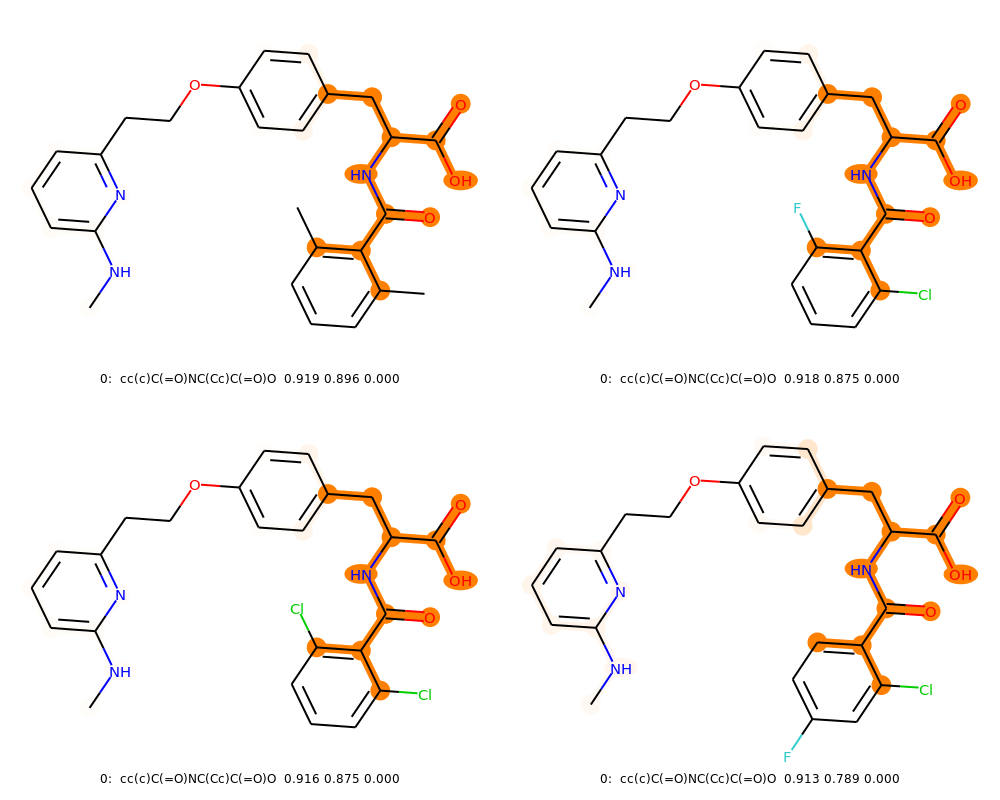

Supplement: Supplementary file 4 — Supplementary file4 (ZIP 111545 KB) [file 10822_2021_421_MOESM4_ESM.zip › 075/tp_cluster_0/tp0_mols_12.png]

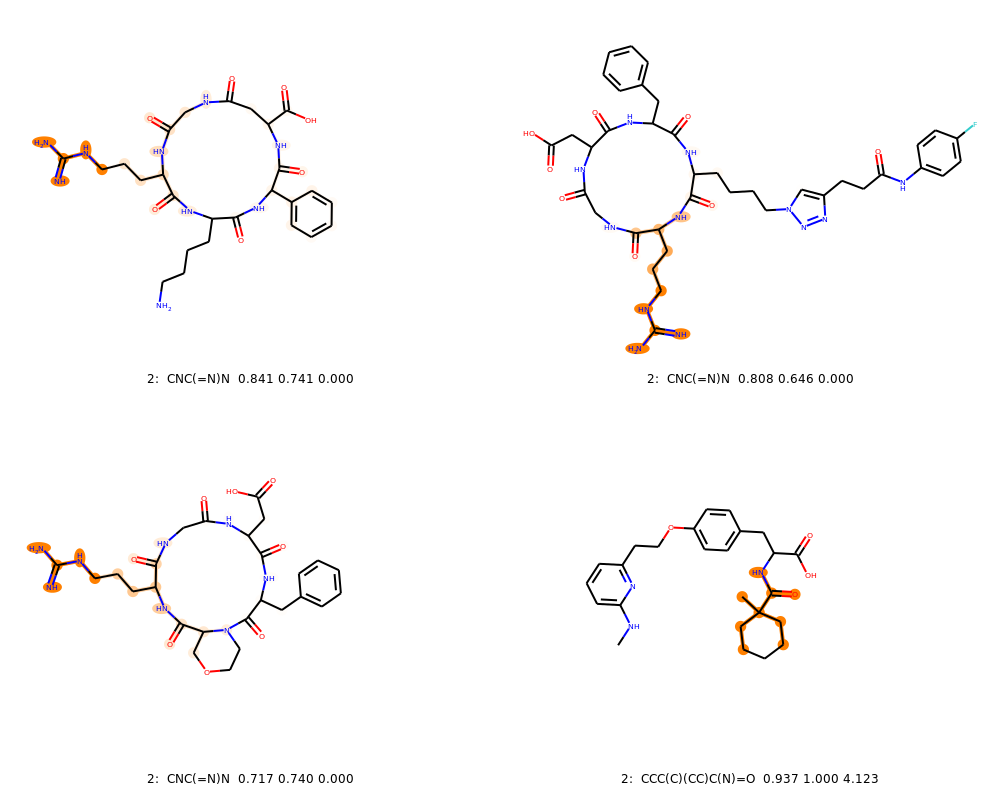

Supplement: Supplementary file 4 — Supplementary file4 (ZIP 111545 KB) [file 10822_2021_421_MOESM4_ESM.zip › 075/tp_cluster_2/tp2_mols_4.png]

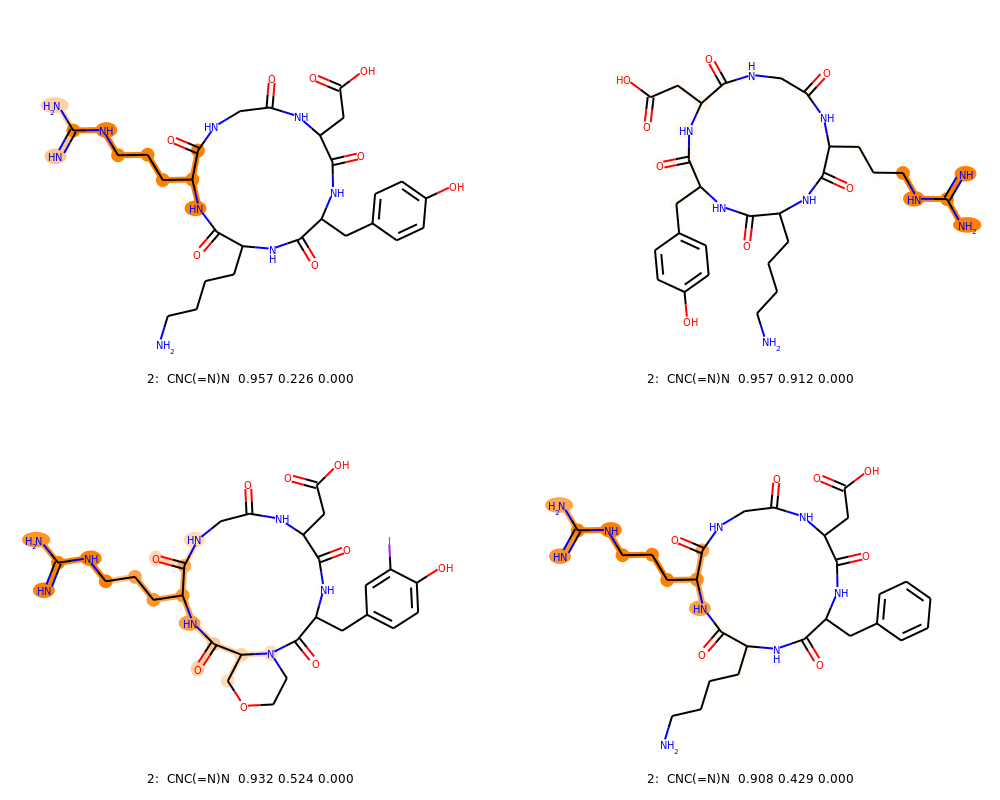

Supplement: Supplementary file 4 — Supplementary file4 (ZIP 111545 KB) [file 10822_2021_421_MOESM4_ESM.zip › 075/tp_cluster_2/tp2_mols_0.png]

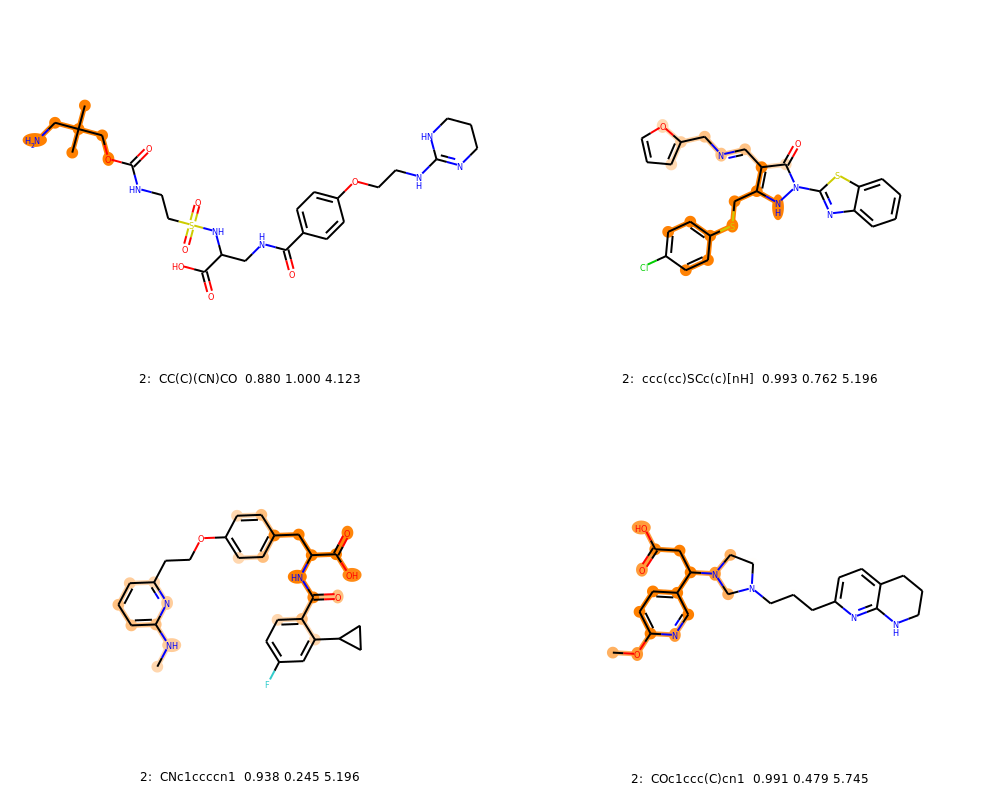

Supplement: Supplementary file 4 — Supplementary file4 (ZIP 111545 KB) [file 10822_2021_421_MOESM4_ESM.zip › 075/tp_cluster_2/tp2_mols_8.png]

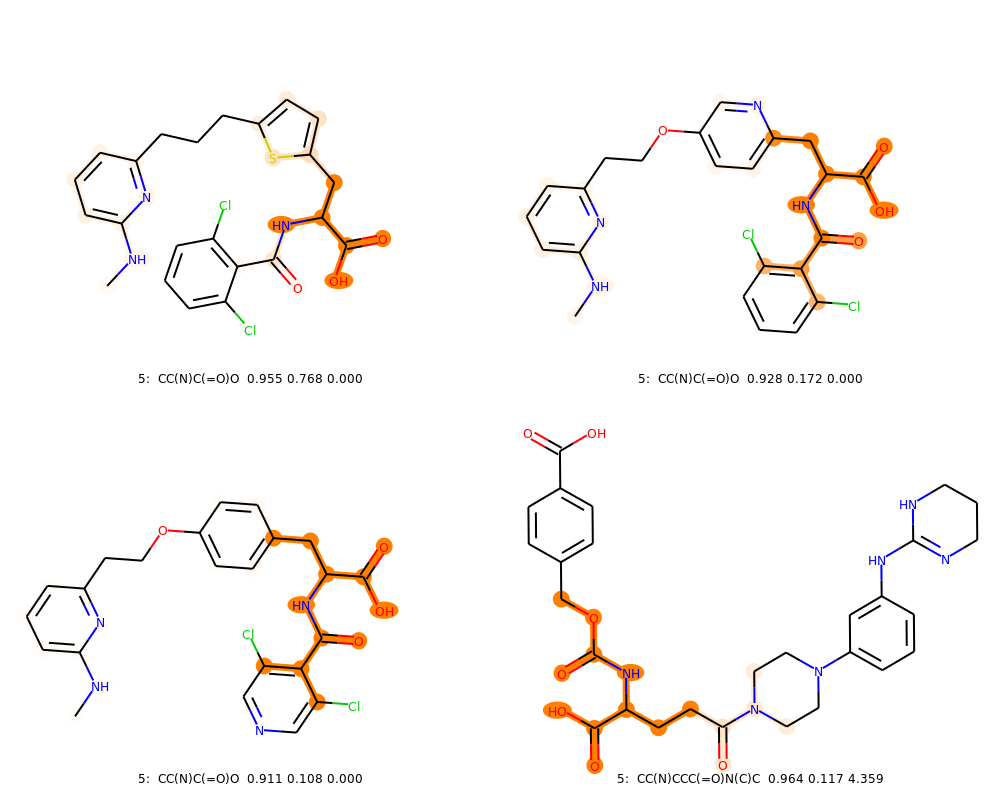

Supplement: Supplementary file 4 — Supplementary file4 (ZIP 111545 KB) [file 10822_2021_421_MOESM4_ESM.zip › 075/tp_cluster_5/tp5_mols_0.png]

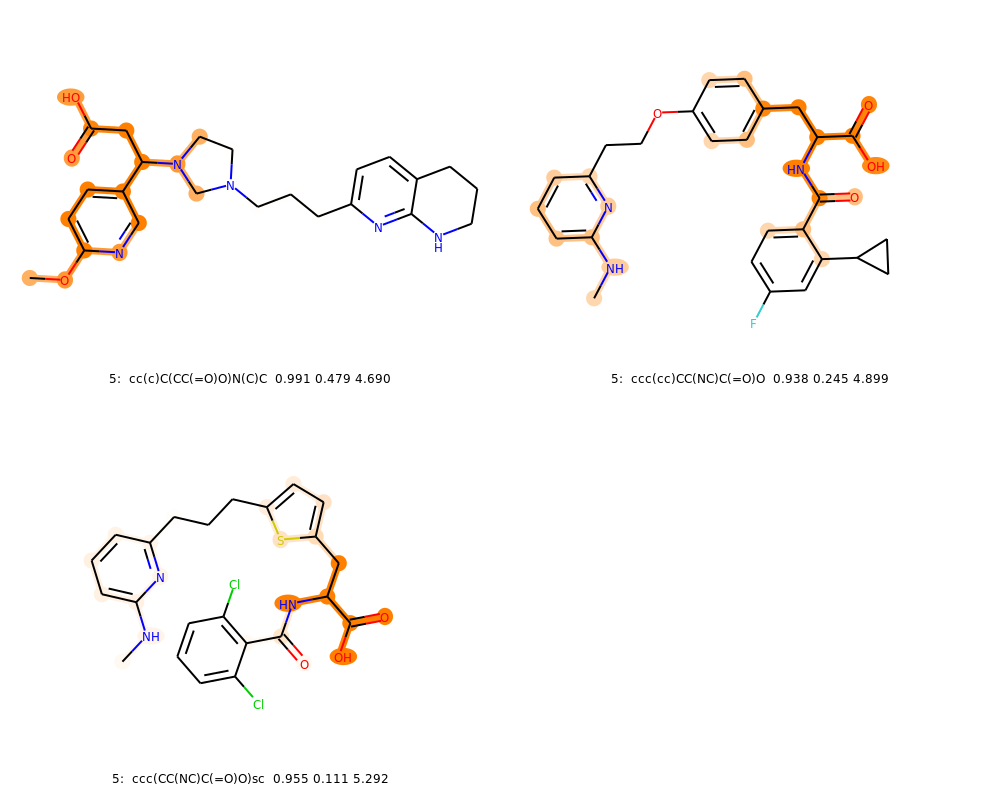

Supplement: Supplementary file 4 — Supplementary file4 (ZIP 111545 KB) [file 10822_2021_421_MOESM4_ESM.zip › 075/tp_cluster_5/tp5_mols_4.png]

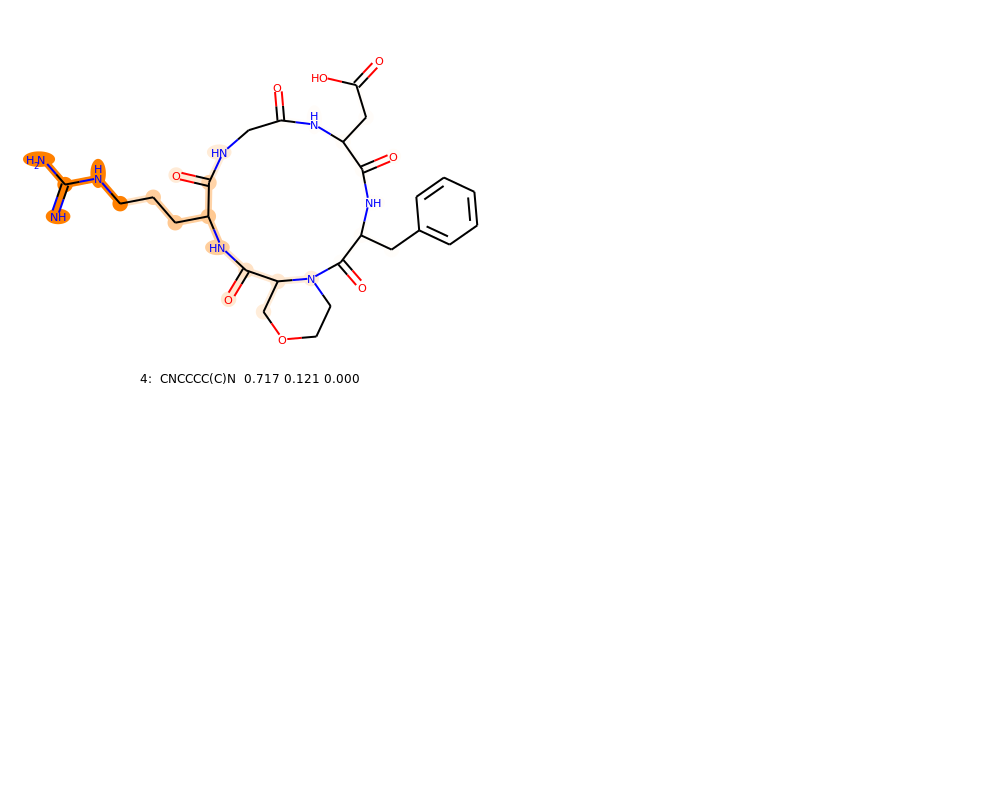

Supplement: Supplementary file 4 — Supplementary file4 (ZIP 111545 KB) [file 10822_2021_421_MOESM4_ESM.zip › 075/tp_cluster_4/tp4_mols_4.png]

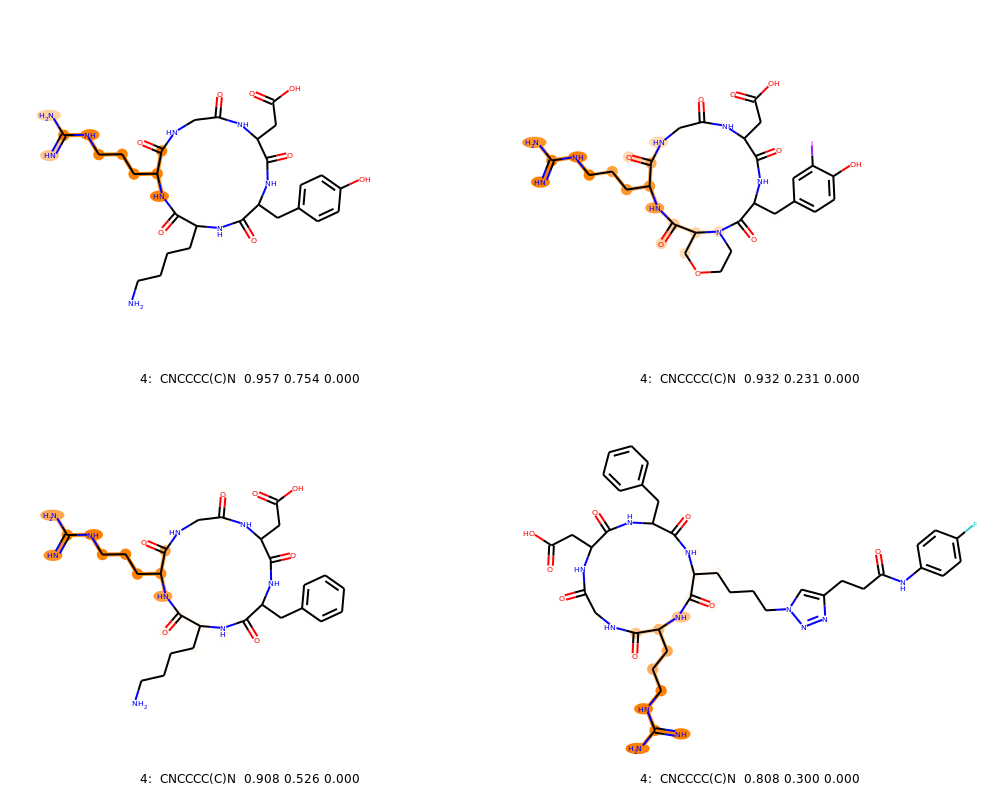

Supplement: Supplementary file 4 — Supplementary file4 (ZIP 111545 KB) [file 10822_2021_421_MOESM4_ESM.zip › 075/tp_cluster_4/tp4_mols_0.png]

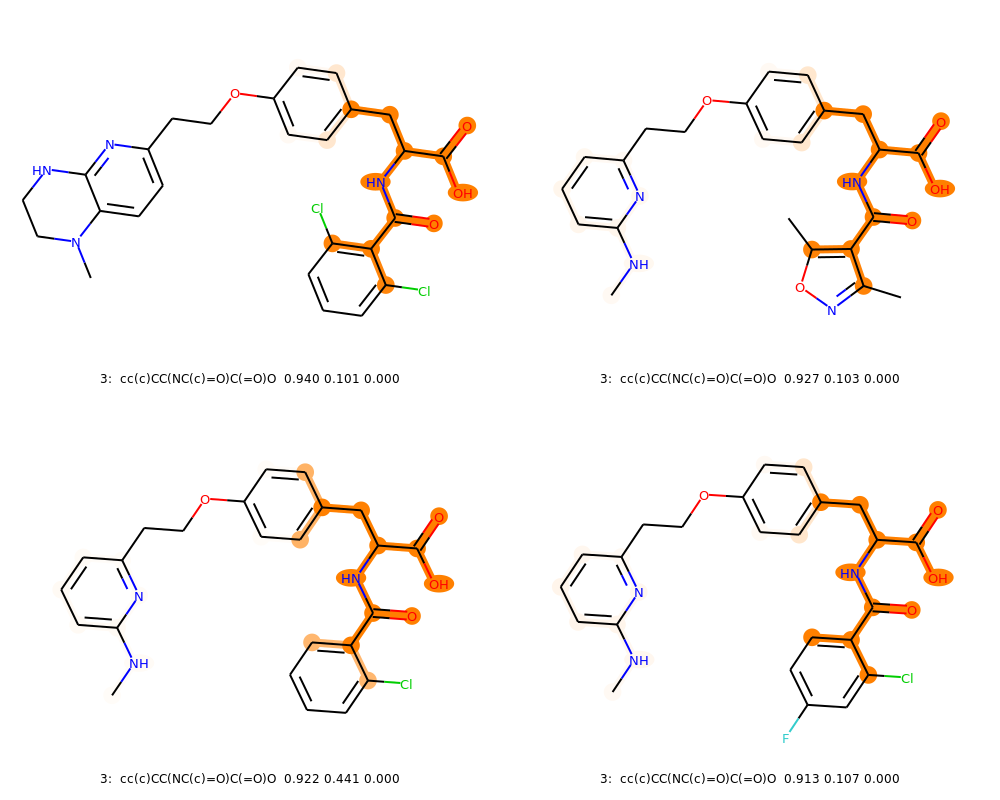

Supplement: Supplementary file 4 — Supplementary file4 (ZIP 111545 KB) [file 10822_2021_421_MOESM4_ESM.zip › 075/tp_cluster_3/tp3_mols_0.png]

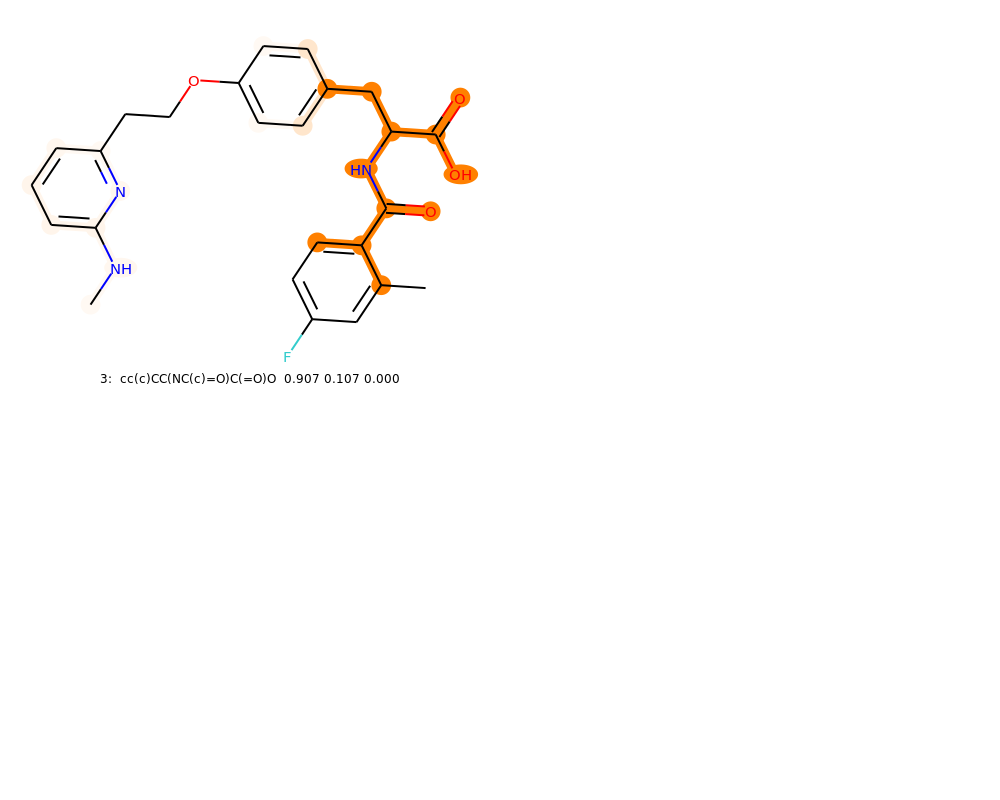

Supplement: Supplementary file 4 — Supplementary file4 (ZIP 111545 KB) [file 10822_2021_421_MOESM4_ESM.zip › 075/tp_cluster_3/tp3_mols_4.png]

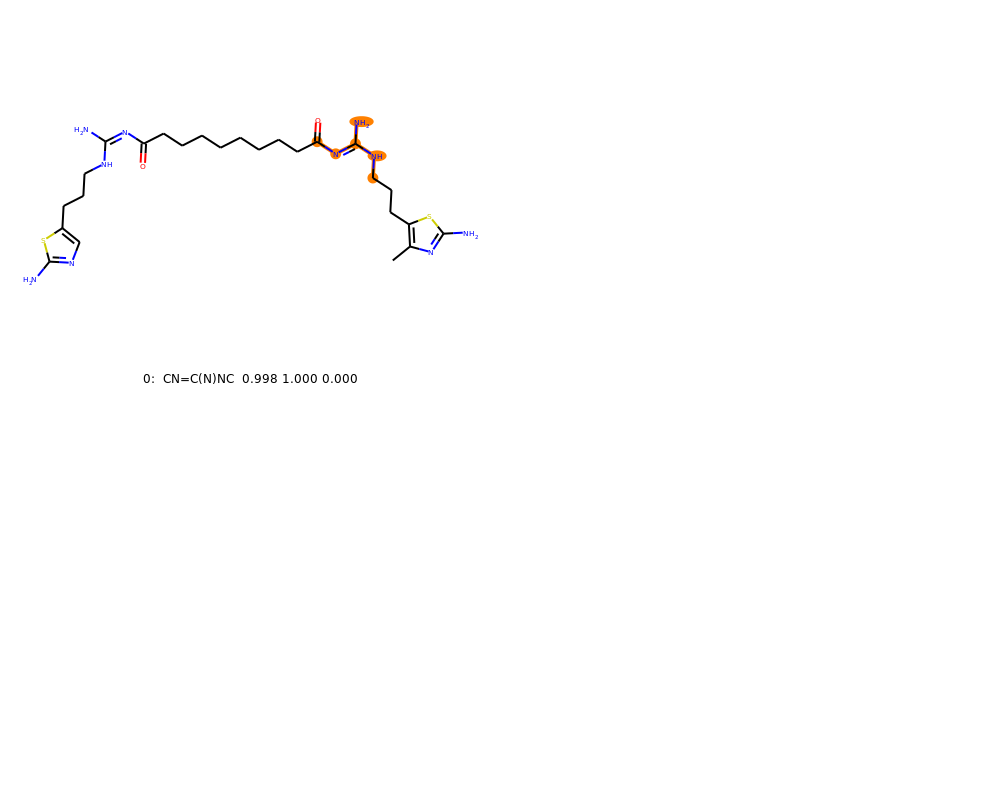

Supplement: Supplementary file 4 — Supplementary file4 (ZIP 111545 KB) [file 10822_2021_421_MOESM4_ESM.zip › 096/tp_cluster_0/tp0_mols_4.png]

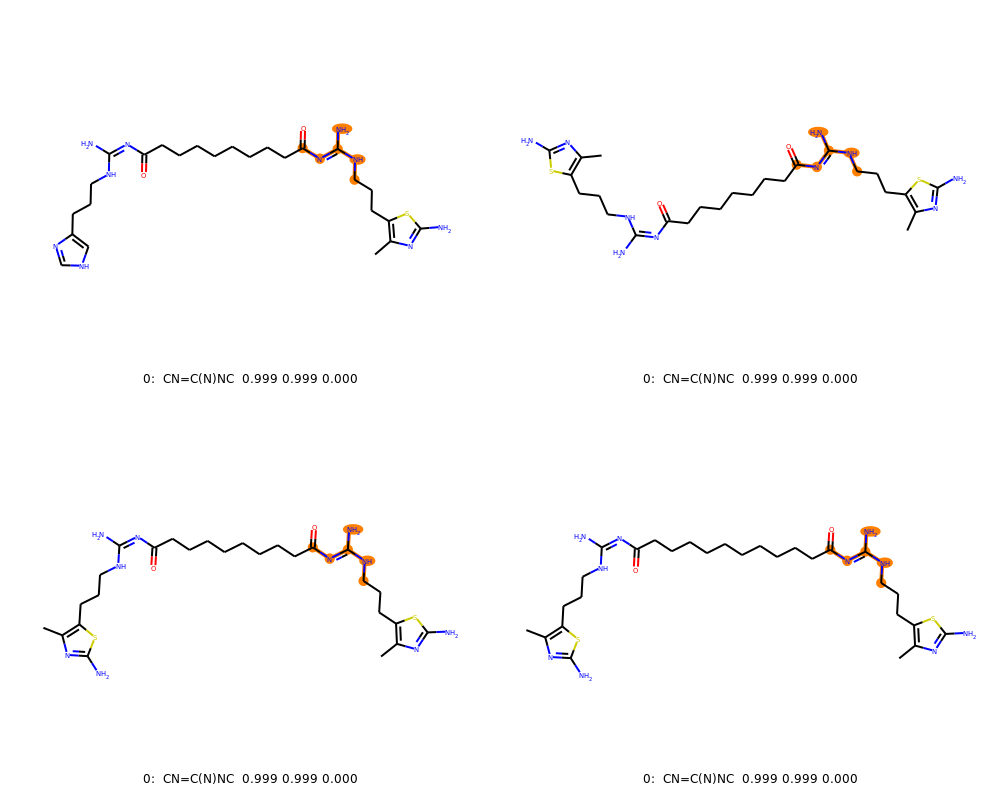

Supplement: Supplementary file 4 — Supplementary file4 (ZIP 111545 KB) [file 10822_2021_421_MOESM4_ESM.zip › 096/tp_cluster_0/tp0_mols_0.png]

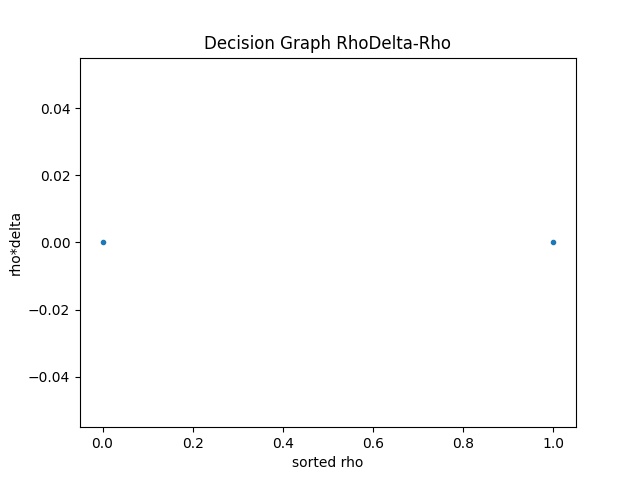

Supplement: Supplementary file 4 — Supplementary file4 (ZIP 111545 KB) [file 10822_2021_421_MOESM4_ESM.zip › 097/tp_decision_graphs/RhoDelta-Rho.jpg]

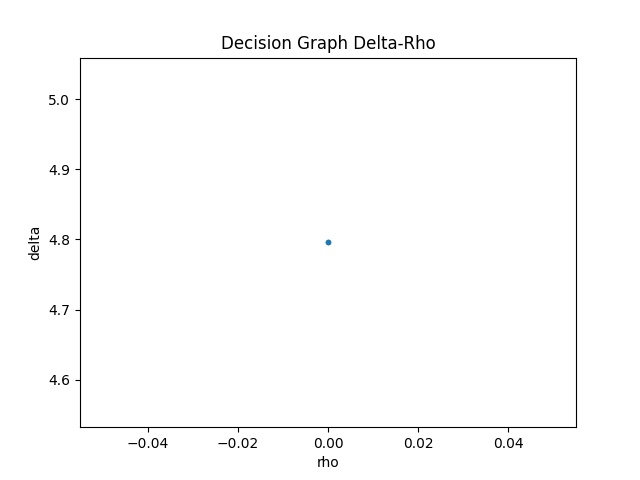

Supplement: Supplementary file 4 — Supplementary file4 (ZIP 111545 KB) [file 10822_2021_421_MOESM4_ESM.zip › 097/tp_decision_graphs/Delta-Rho.jpg]

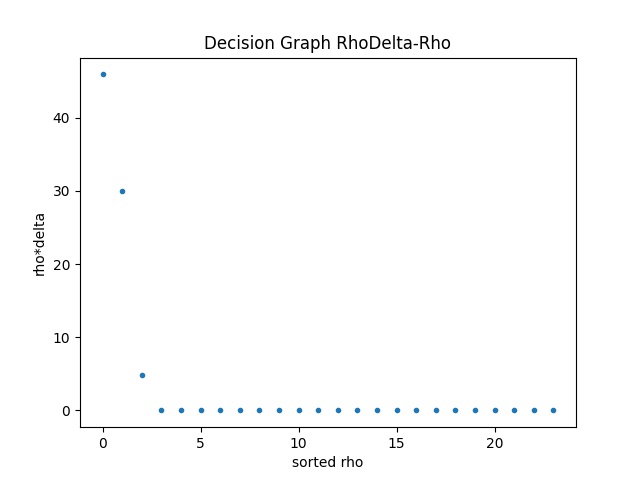

Supplement: Supplementary file 4 — Supplementary file4 (ZIP 111545 KB) [file 10822_2021_421_MOESM4_ESM.zip › 101/tp_decision_graphs/RhoDelta-Rho.jpg]

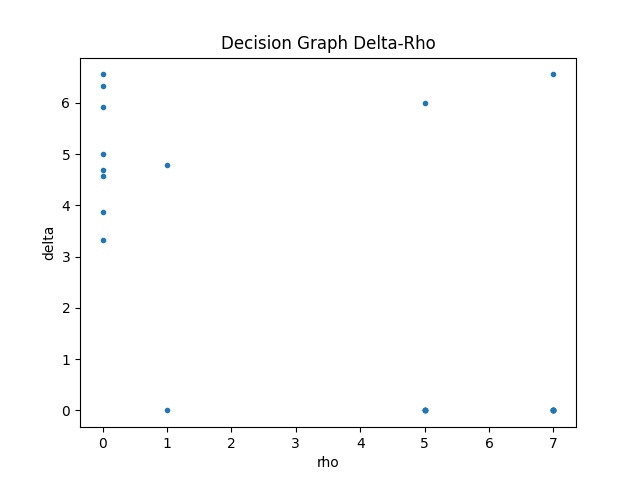

Supplement: Supplementary file 4 — Supplementary file4 (ZIP 111545 KB) [file 10822_2021_421_MOESM4_ESM.zip › 101/tp_decision_graphs/Delta-Rho.jpg]

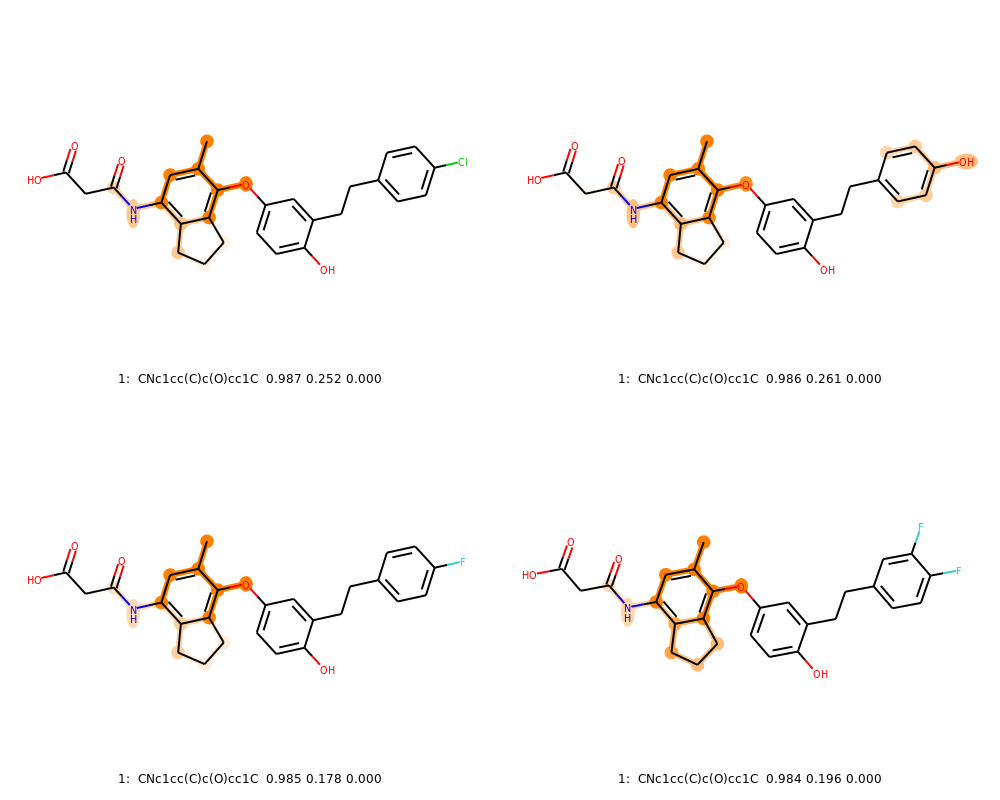

Supplement: Supplementary file 4 — Supplementary file4 (ZIP 111545 KB) [file 10822_2021_421_MOESM4_ESM.zip › 101/tp_cluster_1/tp1_mols_0.png]

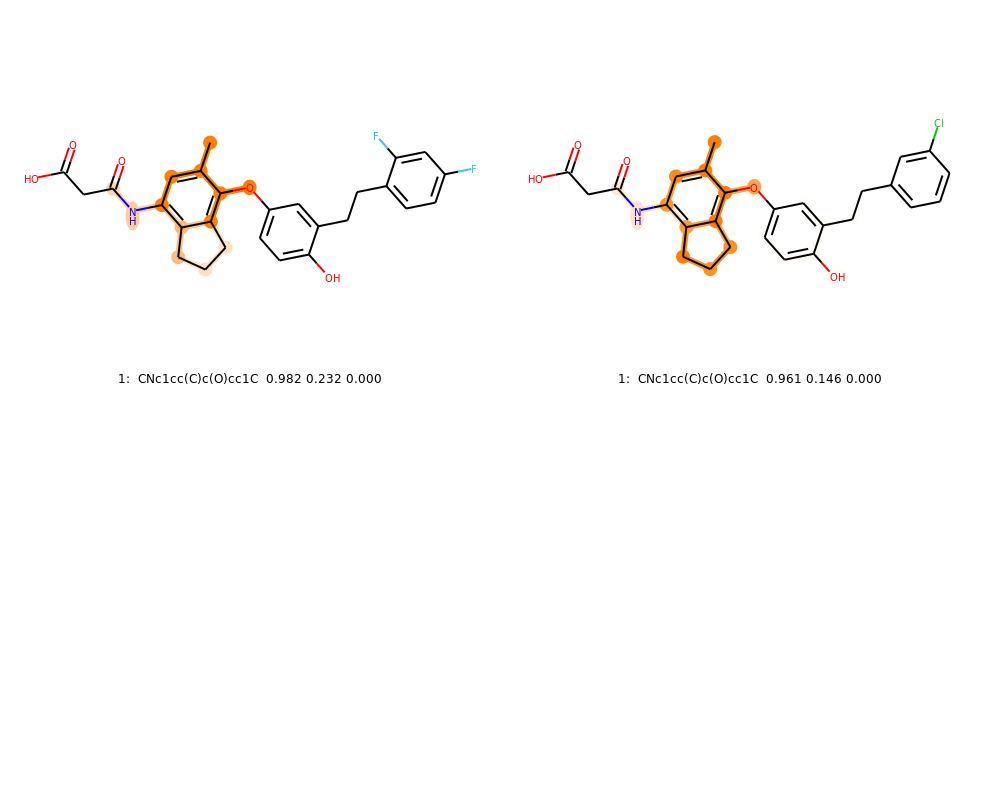

Supplement: Supplementary file 4 — Supplementary file4 (ZIP 111545 KB) [file 10822_2021_421_MOESM4_ESM.zip › 101/tp_cluster_1/tp1_mols_4.png]

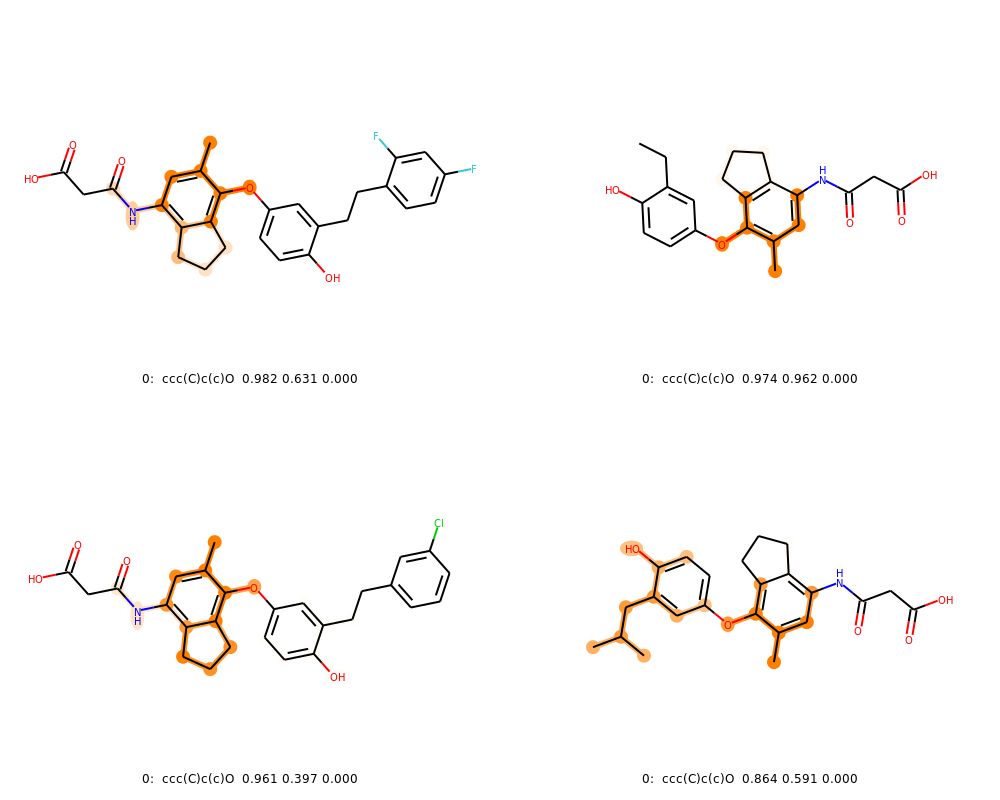

Supplement: Supplementary file 4 — Supplementary file4 (ZIP 111545 KB) [file 10822_2021_421_MOESM4_ESM.zip › 101/tp_cluster_0/tp0_mols_4.png]

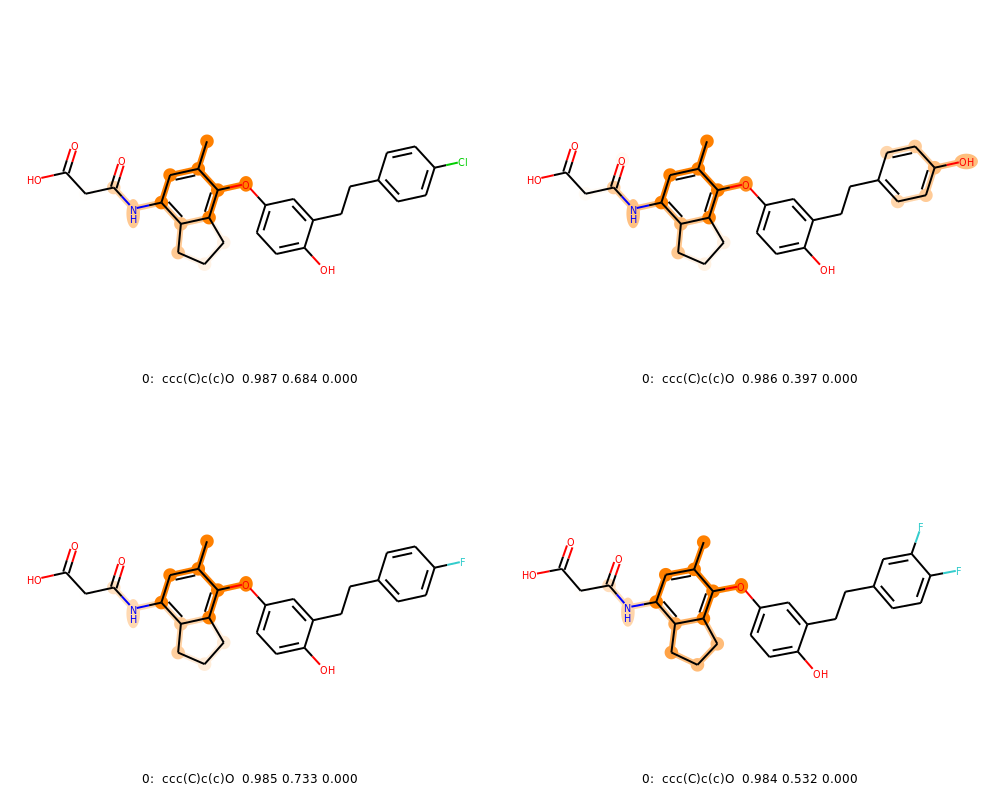

Supplement: Supplementary file 4 — Supplementary file4 (ZIP 111545 KB) [file 10822_2021_421_MOESM4_ESM.zip › 101/tp_cluster_0/tp0_mols_0.png]

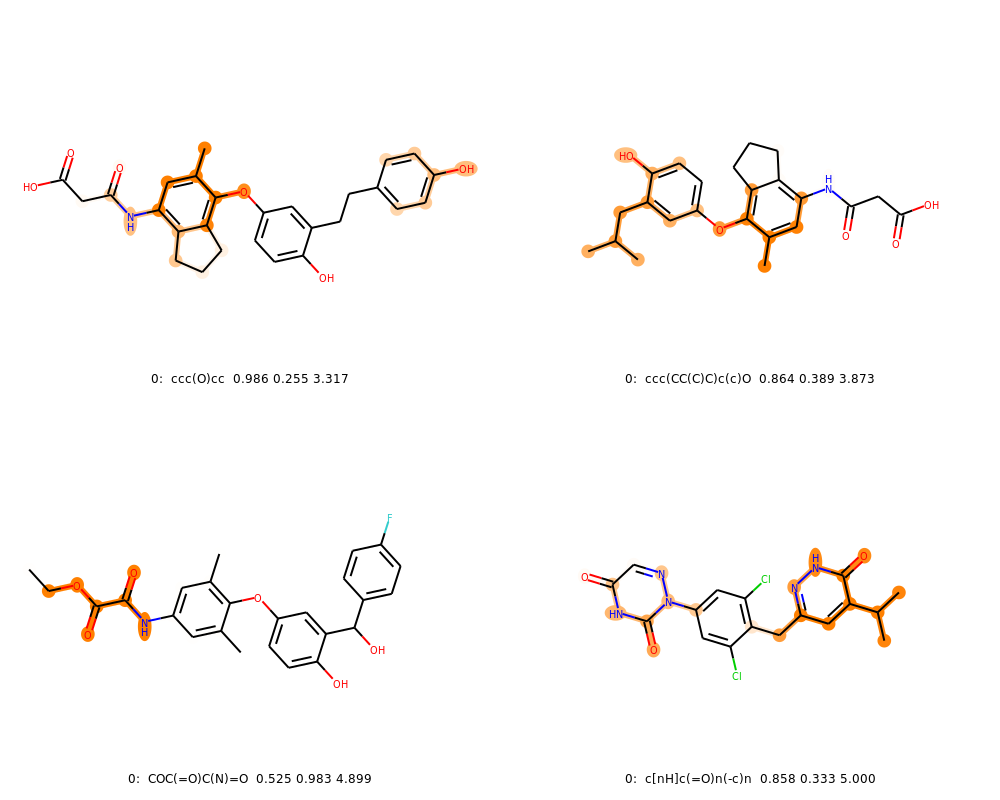

Supplement: Supplementary file 4 — Supplementary file4 (ZIP 111545 KB) [file 10822_2021_421_MOESM4_ESM.zip › 101/tp_cluster_0/tp0_mols_8.png]

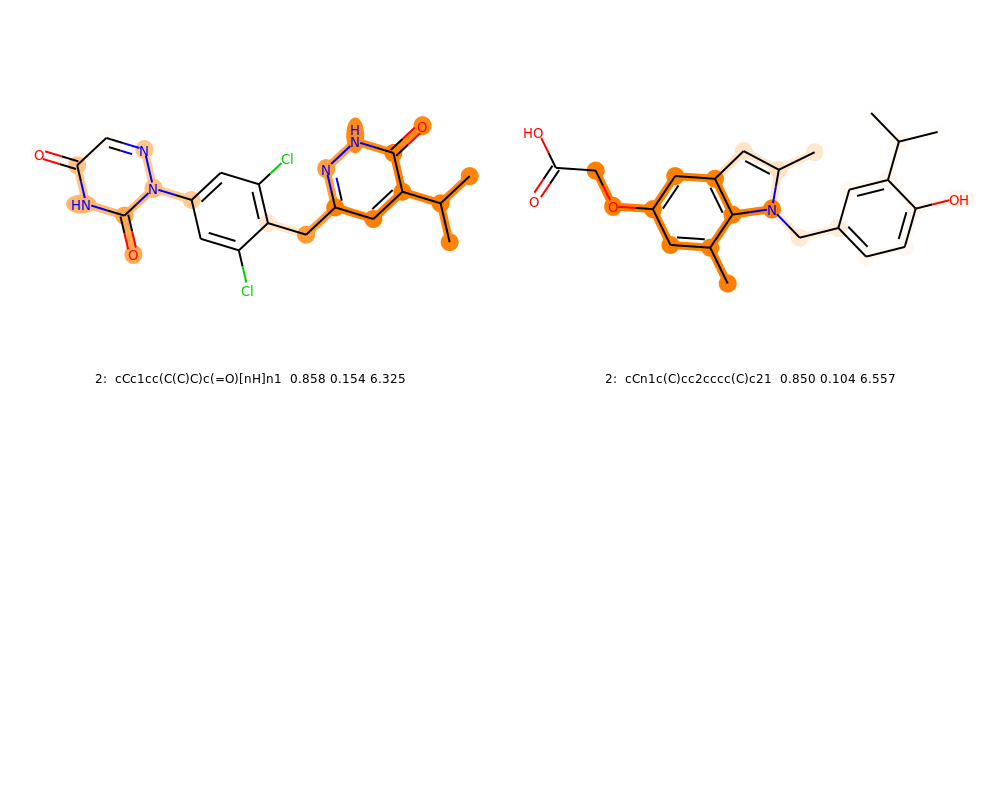

Supplement: Supplementary file 4 — Supplementary file4 (ZIP 111545 KB) [file 10822_2021_421_MOESM4_ESM.zip › 101/tp_cluster_2/tp2_mols_4.png]

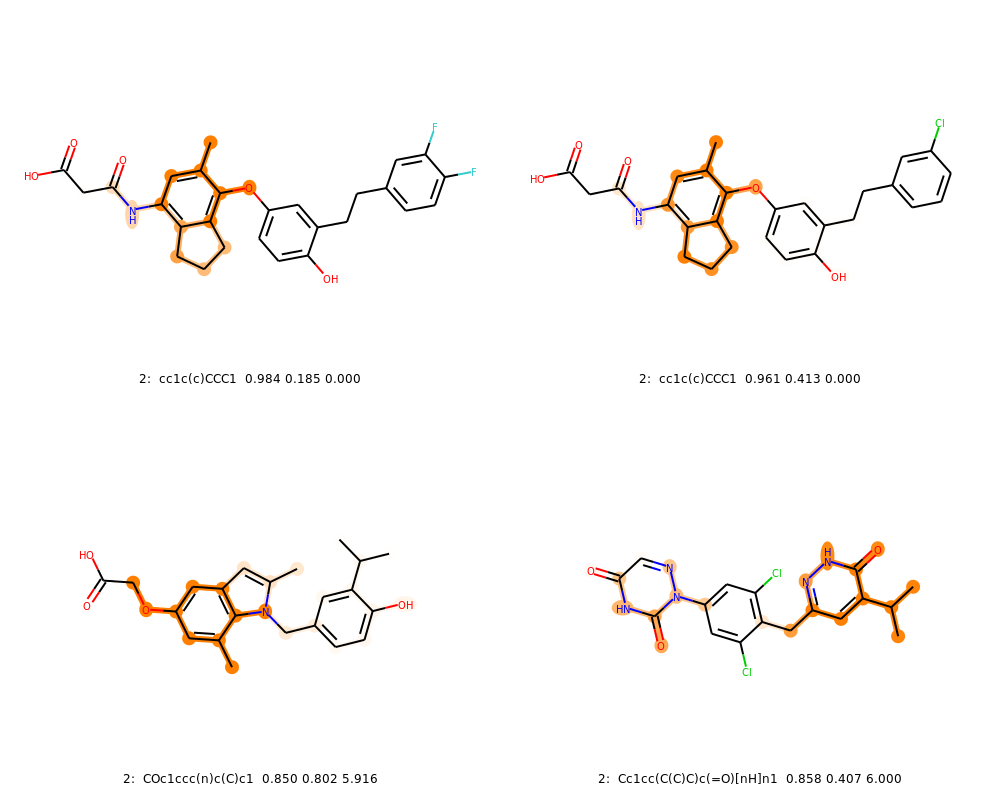

Supplement: Supplementary file 4 — Supplementary file4 (ZIP 111545 KB) [file 10822_2021_421_MOESM4_ESM.zip › 101/tp_cluster_2/tp2_mols_0.png]

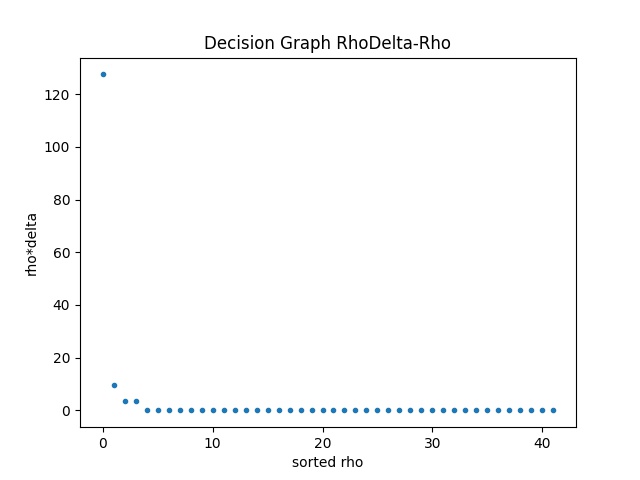

Supplement: Supplementary file 4 — Supplementary file4 (ZIP 111545 KB) [file 10822_2021_421_MOESM4_ESM.zip › 113/tp_decision_graphs/RhoDelta-Rho.jpg]

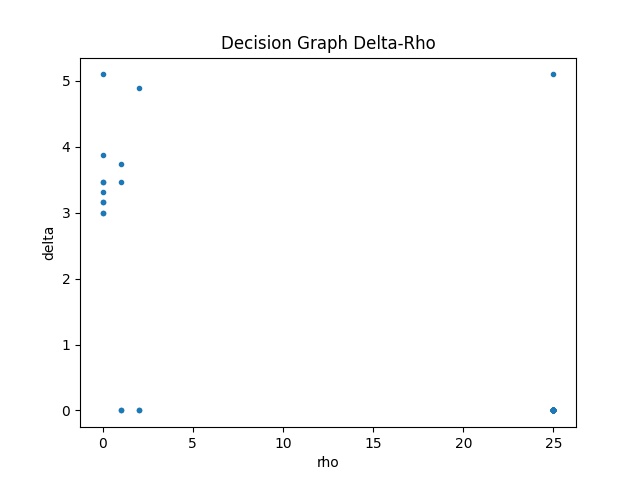

Supplement: Supplementary file 4 — Supplementary file4 (ZIP 111545 KB) [file 10822_2021_421_MOESM4_ESM.zip › 113/tp_decision_graphs/Delta-Rho.jpg]

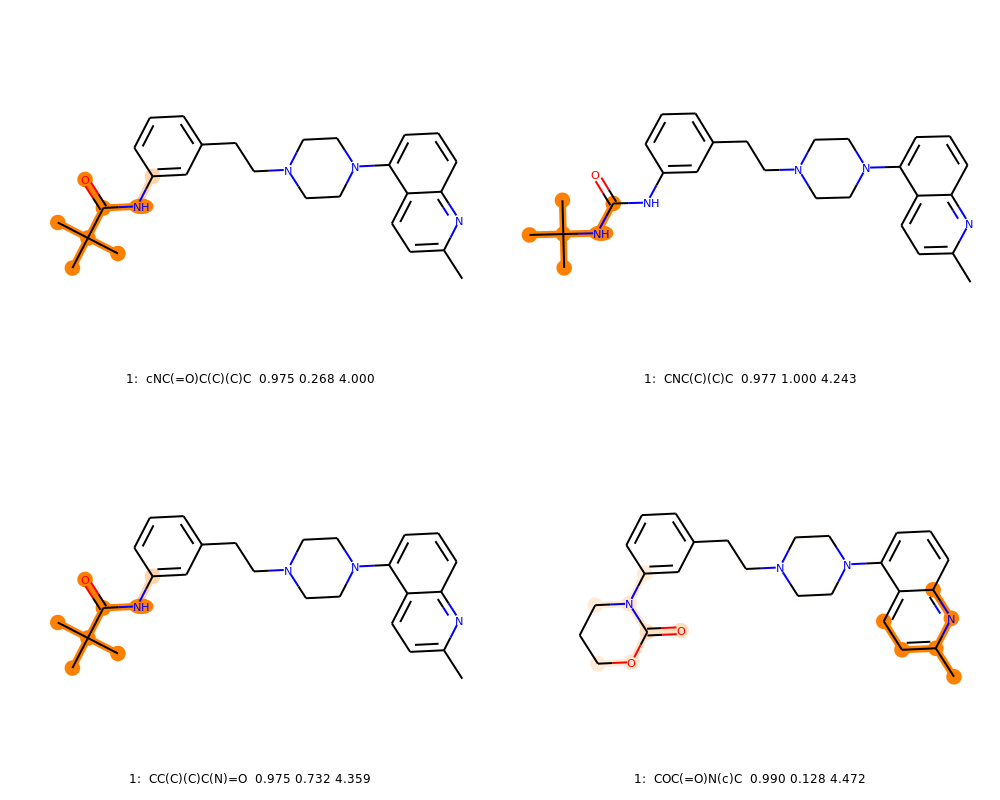

Supplement: Supplementary file 4 — Supplementary file4 (ZIP 111545 KB) [file 10822_2021_421_MOESM4_ESM.zip › 113/tp_cluster_1/tp1_mols_8.png]

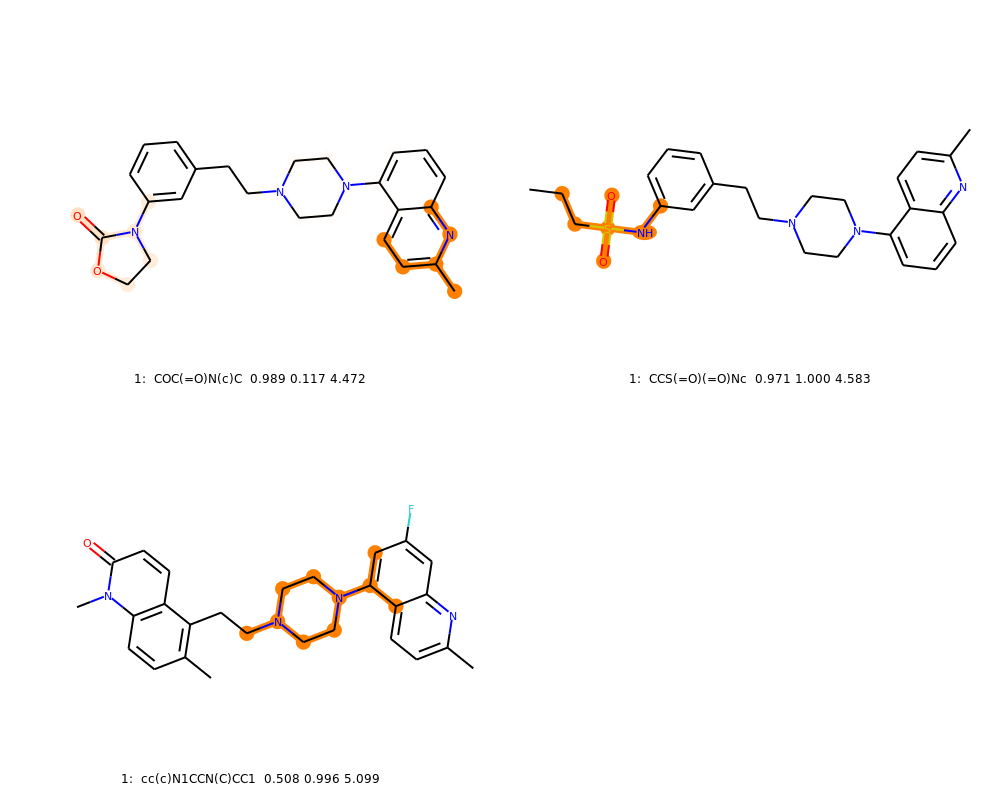

Supplement: Supplementary file 4 — Supplementary file4 (ZIP 111545 KB) [file 10822_2021_421_MOESM4_ESM.zip › 113/tp_cluster_1/tp1_mols_12.png]

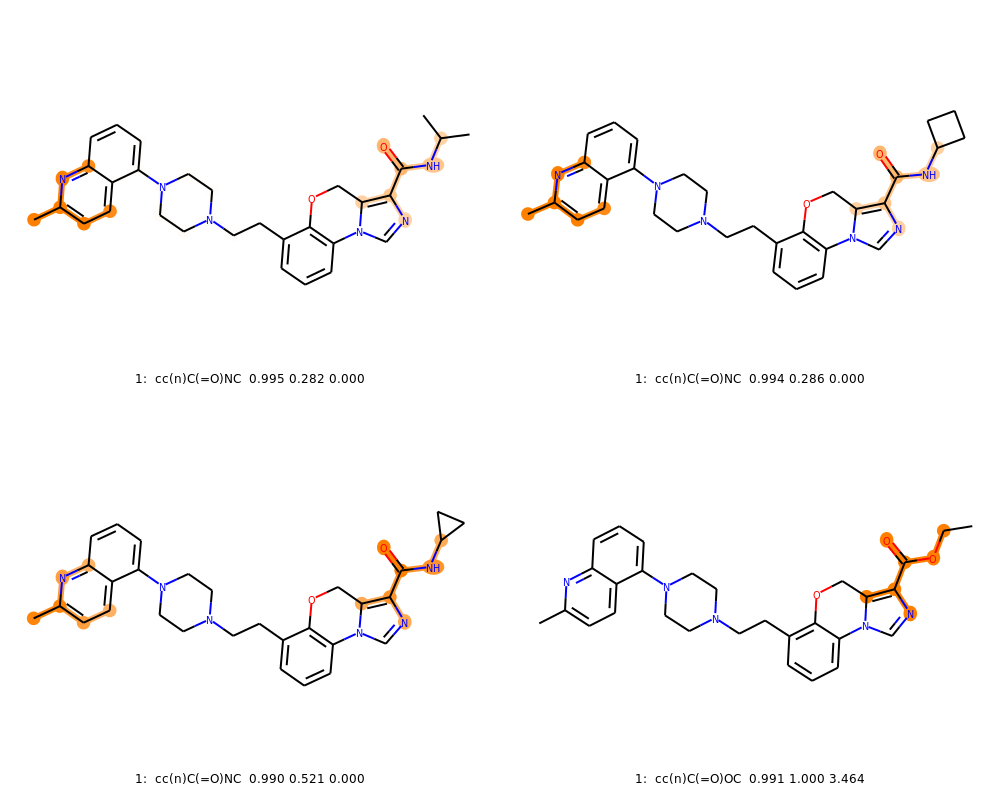

Supplement: Supplementary file 4 — Supplementary file4 (ZIP 111545 KB) [file 10822_2021_421_MOESM4_ESM.zip › 113/tp_cluster_1/tp1_mols_0.png]

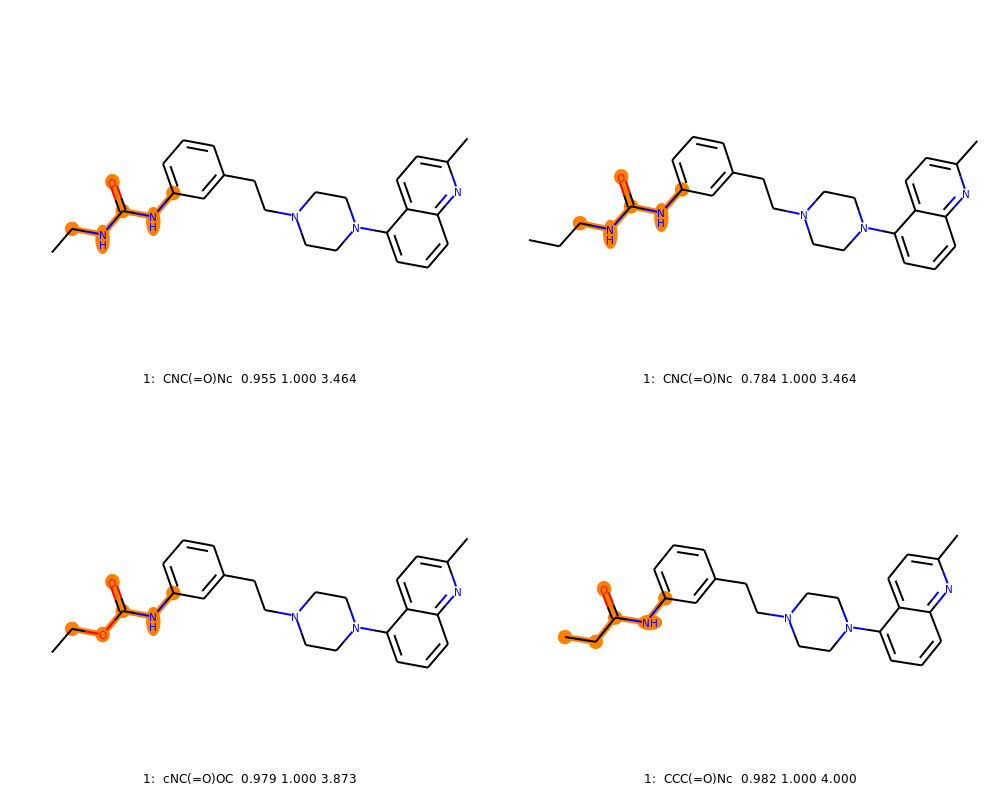

Supplement: Supplementary file 4 — Supplementary file4 (ZIP 111545 KB) [file 10822_2021_421_MOESM4_ESM.zip › 113/tp_cluster_1/tp1_mols_4.png]

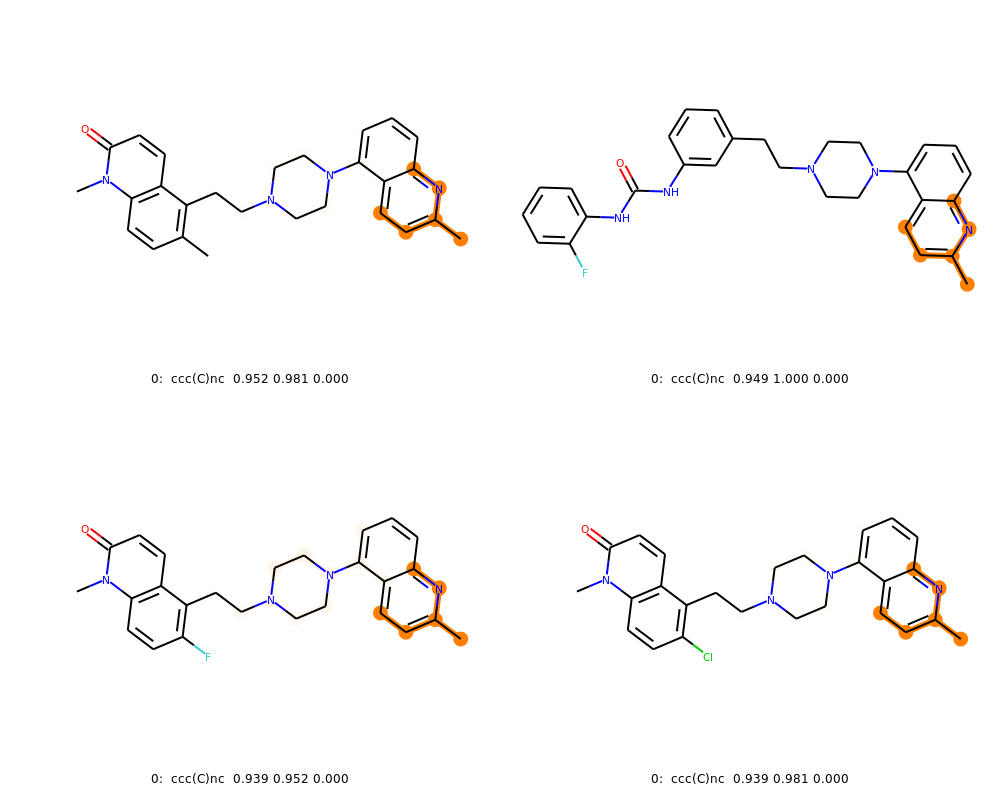

Supplement: Supplementary file 4 — Supplementary file4 (ZIP 111545 KB) [file 10822_2021_421_MOESM4_ESM.zip › 113/tp_cluster_0/tp0_mols_20.png]

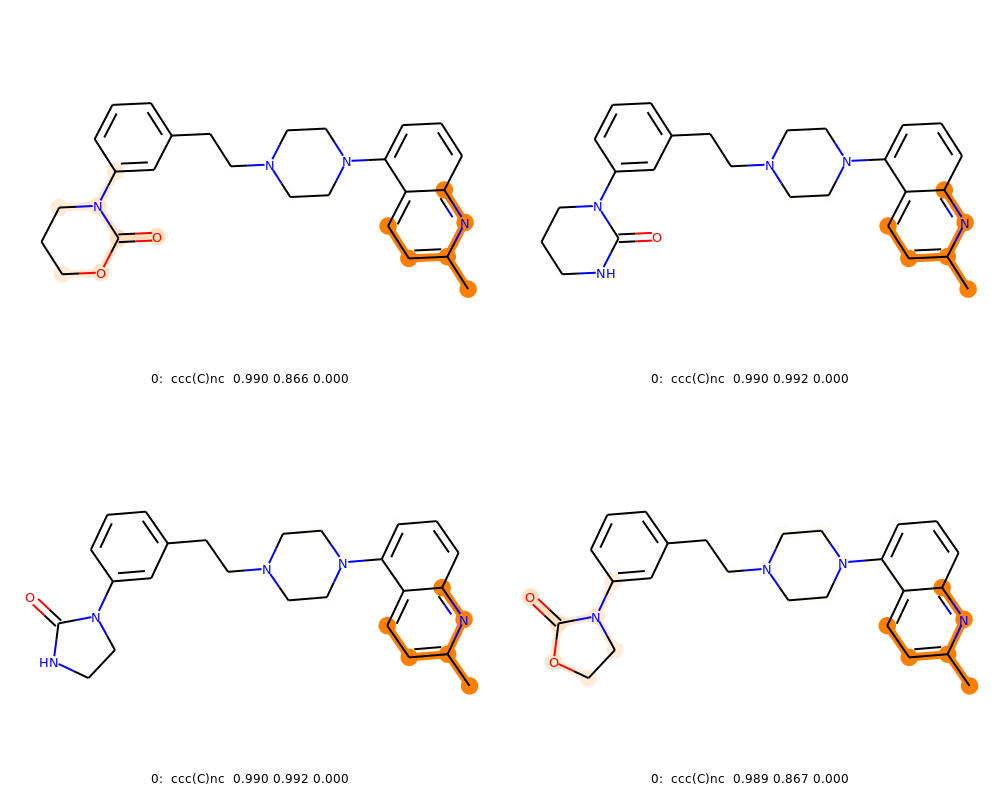

Supplement: Supplementary file 4 — Supplementary file4 (ZIP 111545 KB) [file 10822_2021_421_MOESM4_ESM.zip › 113/tp_cluster_0/tp0_mols_4.png]

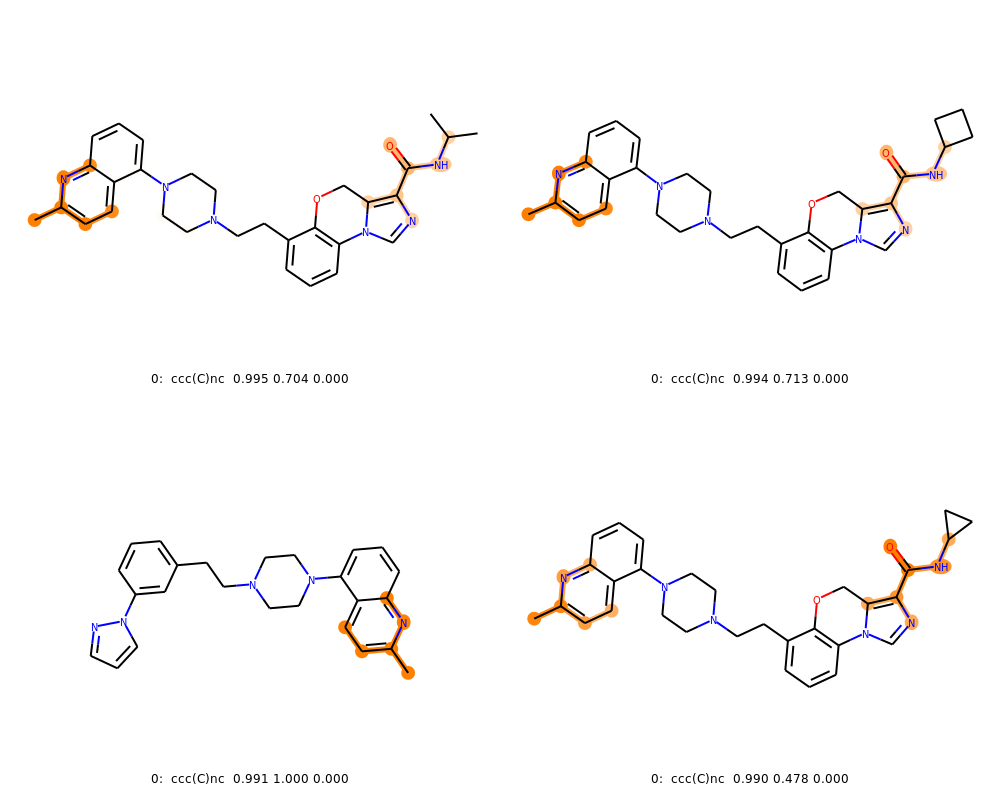

Supplement: Supplementary file 4 — Supplementary file4 (ZIP 111545 KB) [file 10822_2021_421_MOESM4_ESM.zip › 113/tp_cluster_0/tp0_mols_0.png]

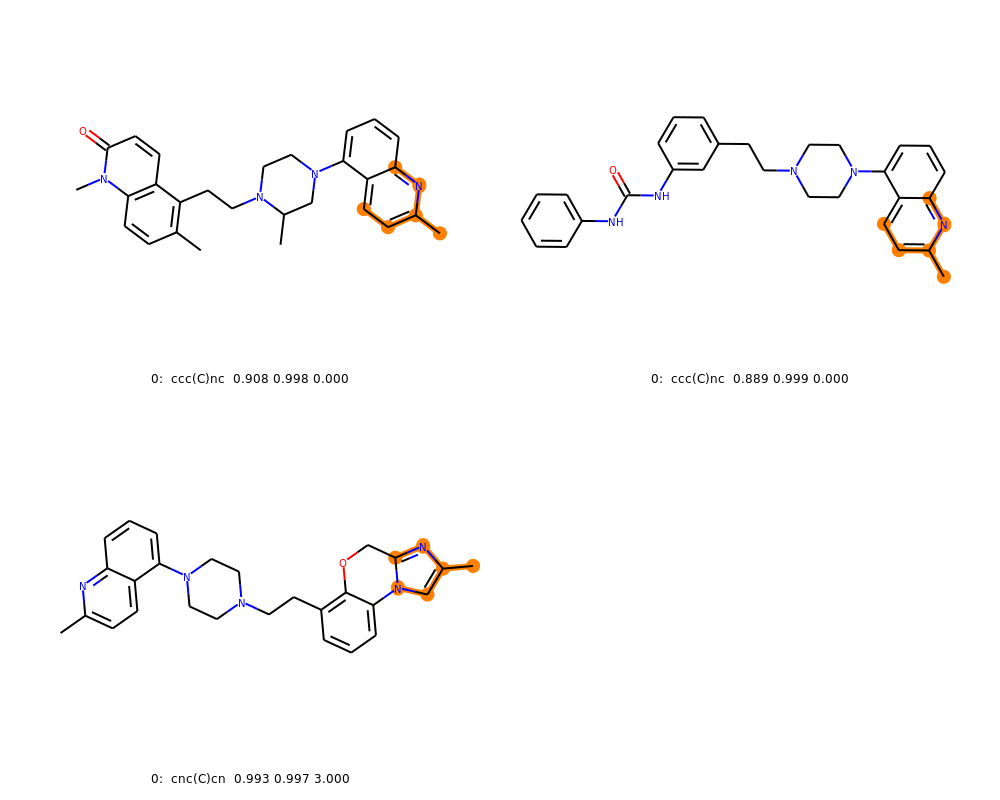

Supplement: Supplementary file 4 — Supplementary file4 (ZIP 111545 KB) [file 10822_2021_421_MOESM4_ESM.zip › 113/tp_cluster_0/tp0_mols_24.png]

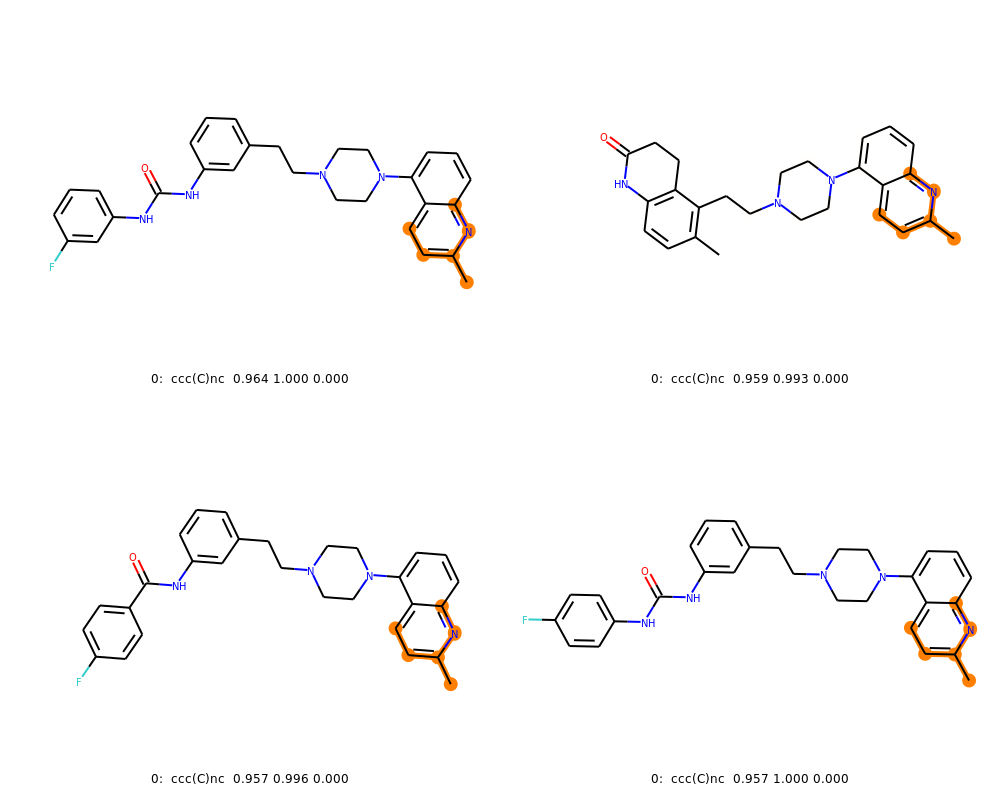

Supplement: Supplementary file 4 — Supplementary file4 (ZIP 111545 KB) [file 10822_2021_421_MOESM4_ESM.zip › 113/tp_cluster_0/tp0_mols_16.png]

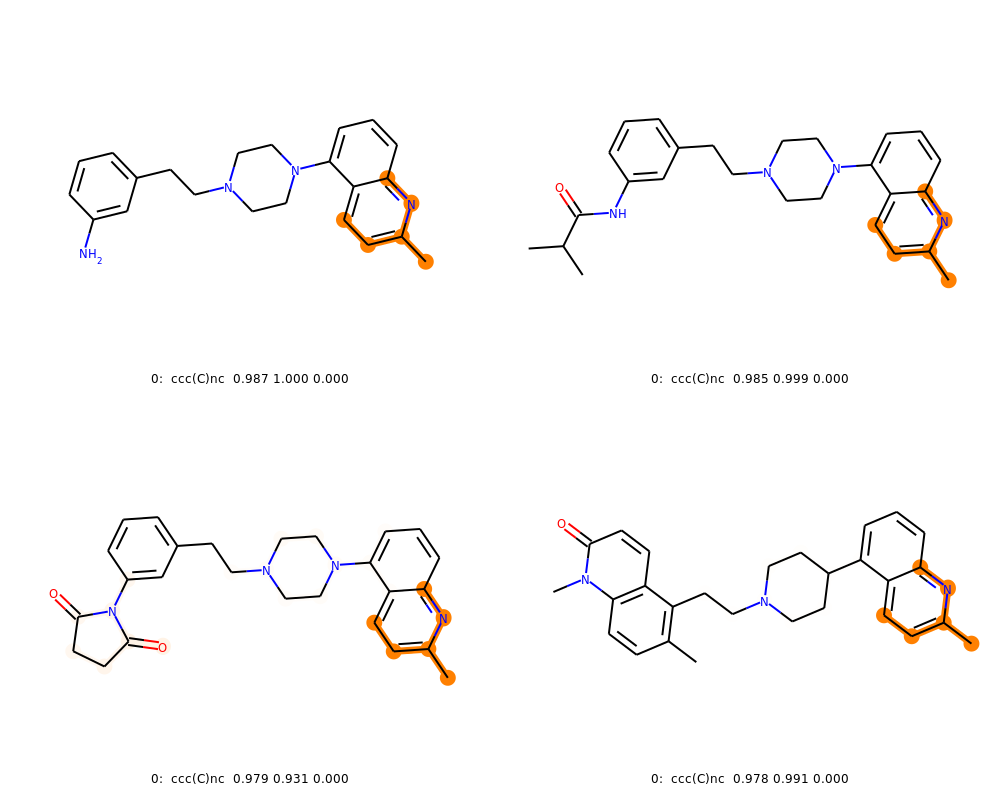

Supplement: Supplementary file 4 — Supplementary file4 (ZIP 111545 KB) [file 10822_2021_421_MOESM4_ESM.zip › 113/tp_cluster_0/tp0_mols_8.png]

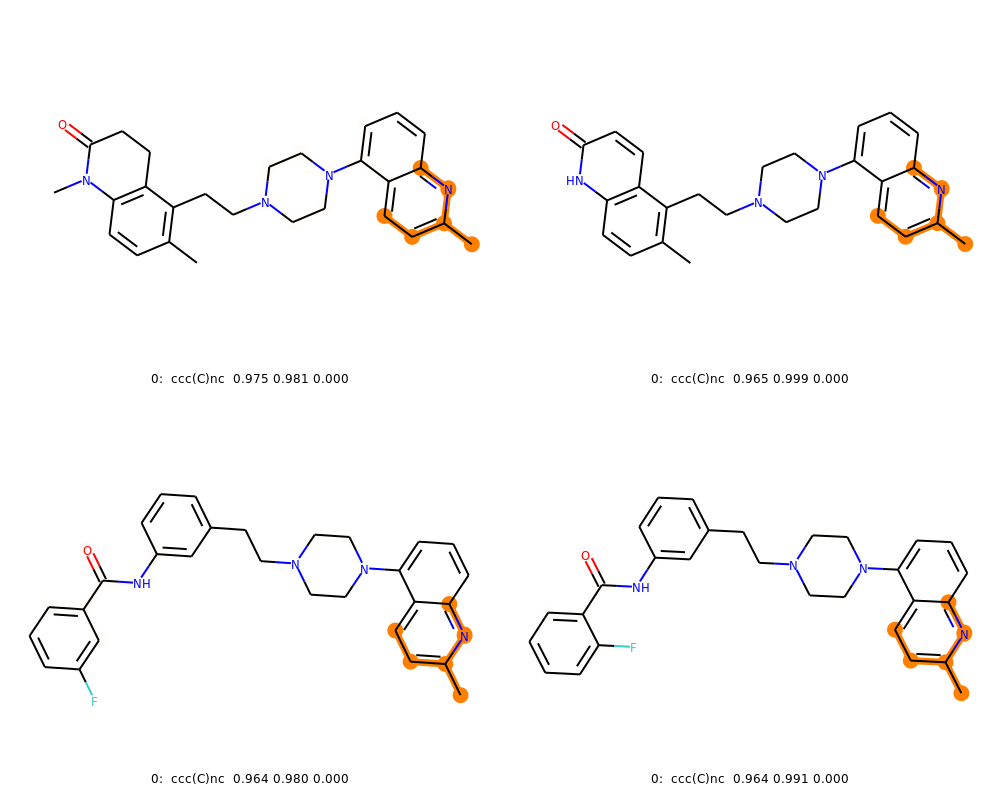

Supplement: Supplementary file 4 — Supplementary file4 (ZIP 111545 KB) [file 10822_2021_421_MOESM4_ESM.zip › 113/tp_cluster_0/tp0_mols_12.png]

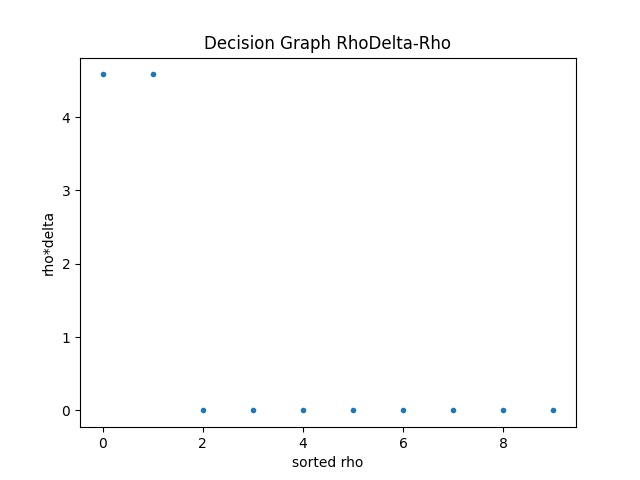

Supplement: Supplementary file 4 — Supplementary file4 (ZIP 111545 KB) [file 10822_2021_421_MOESM4_ESM.zip › 115/tp_decision_graphs/RhoDelta-Rho.jpg]

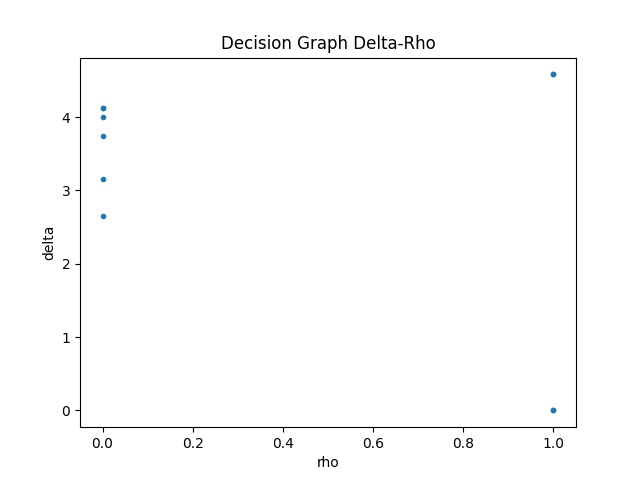

Supplement: Supplementary file 4 — Supplementary file4 (ZIP 111545 KB) [file 10822_2021_421_MOESM4_ESM.zip › 115/tp_decision_graphs/Delta-Rho.jpg]

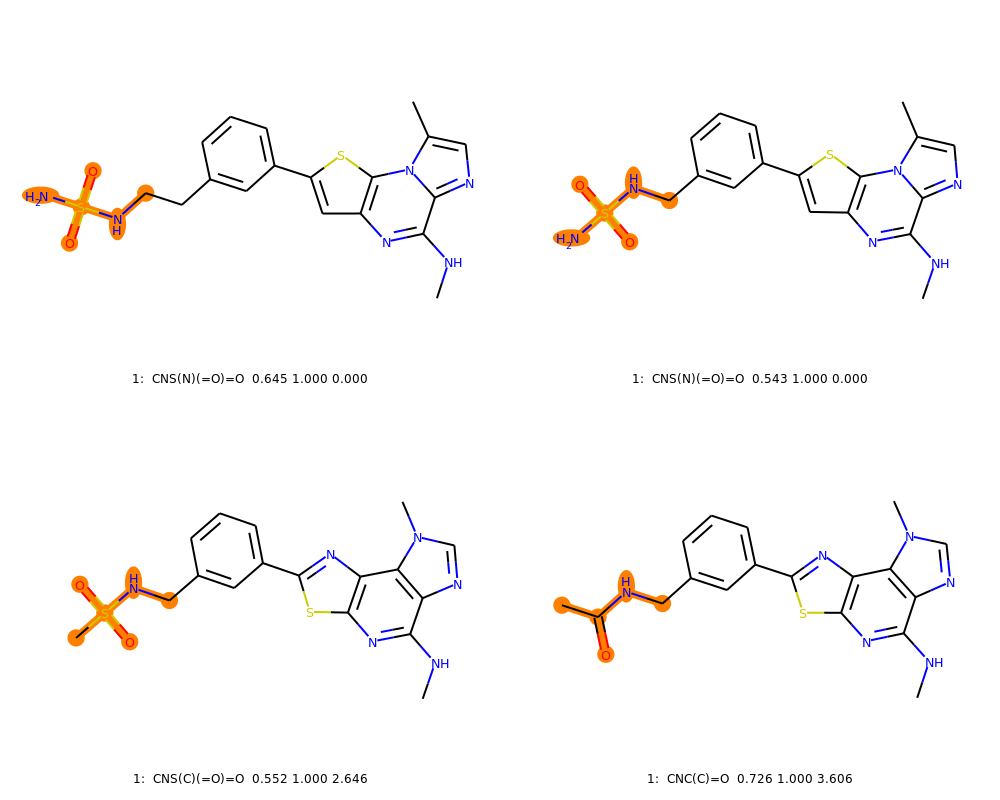

Supplement: Supplementary file 4 — Supplementary file4 (ZIP 111545 KB) [file 10822_2021_421_MOESM4_ESM.zip › 115/tp_cluster_1/tp1_mols_0.png]

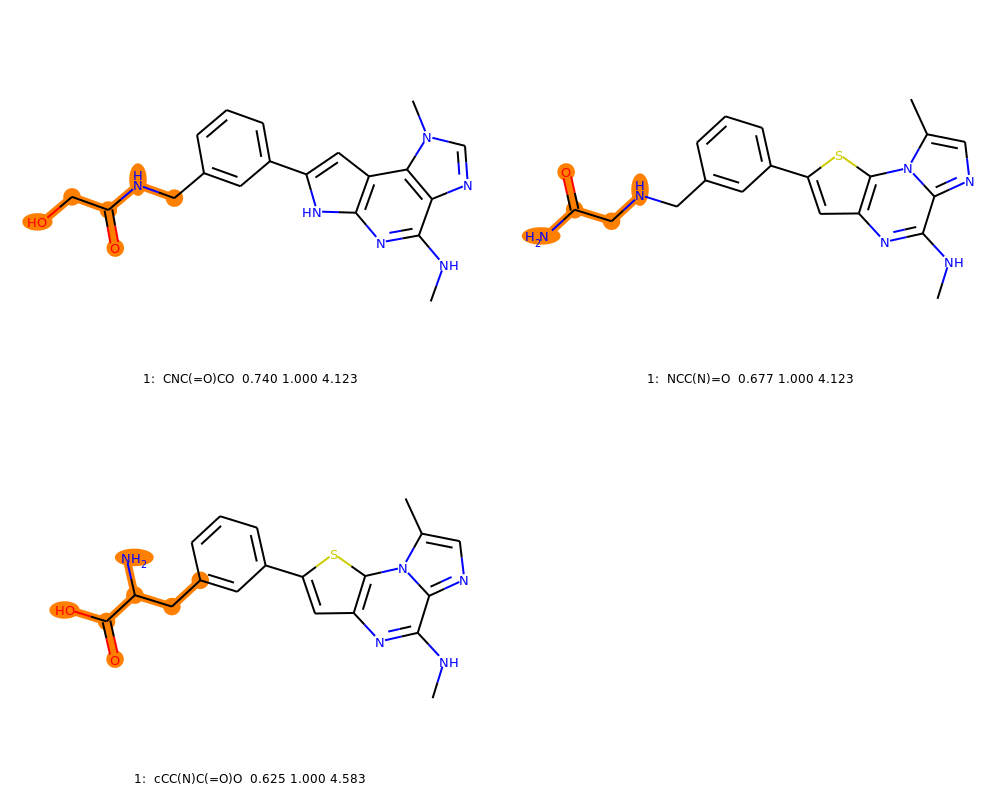

Supplement: Supplementary file 4 — Supplementary file4 (ZIP 111545 KB) [file 10822_2021_421_MOESM4_ESM.zip › 115/tp_cluster_1/tp1_mols_4.png]

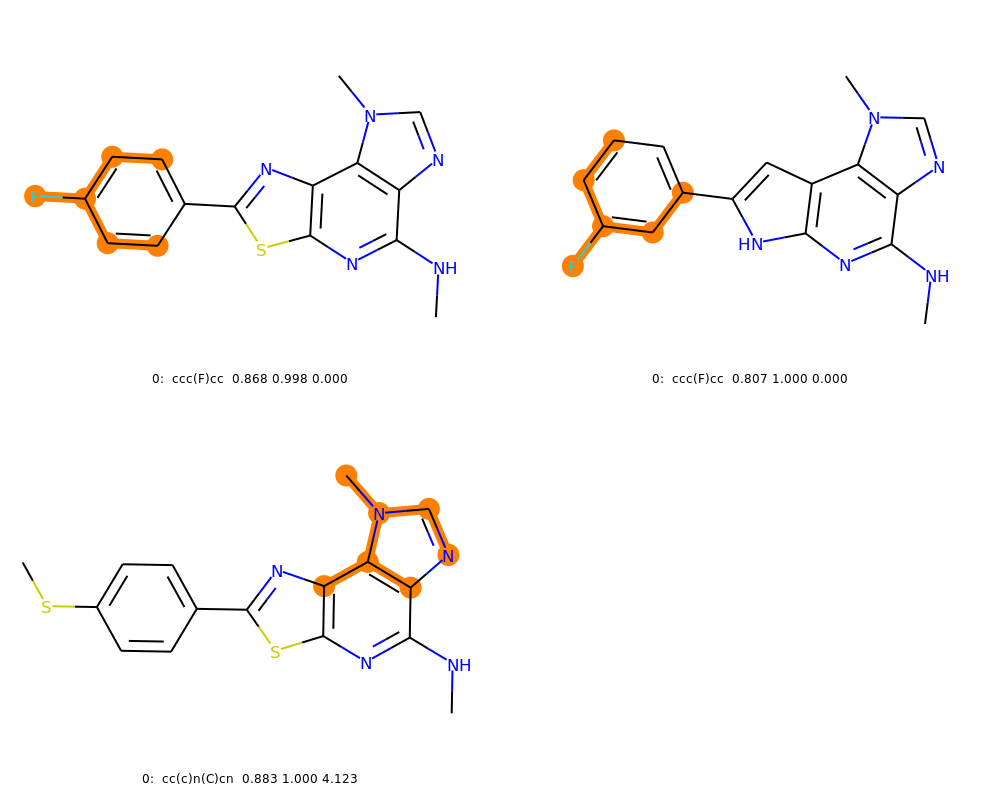

Supplement: Supplementary file 4 — Supplementary file4 (ZIP 111545 KB) [file 10822_2021_421_MOESM4_ESM.zip › 115/tp_cluster_0/tp0_mols_0.png]

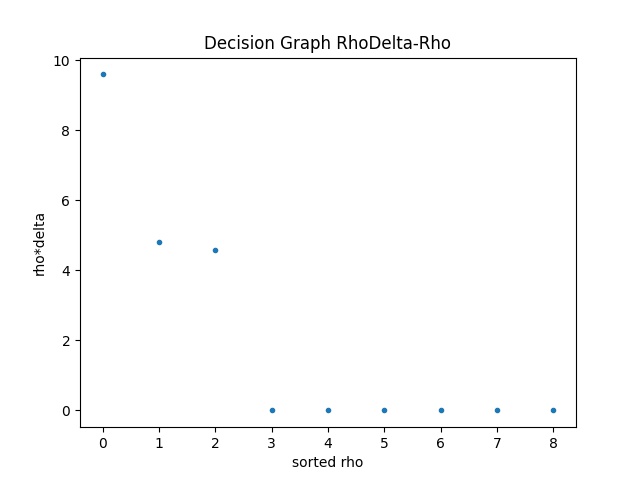

Supplement: Supplementary file 4 — Supplementary file4 (ZIP 111545 KB) [file 10822_2021_421_MOESM4_ESM.zip › 131/tp_decision_graphs/RhoDelta-Rho.jpg]

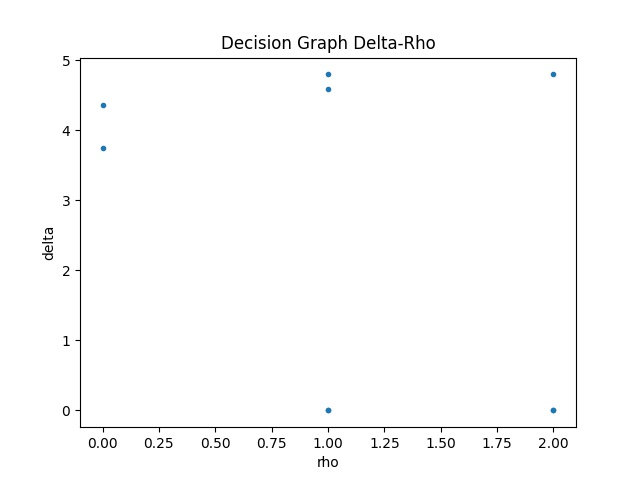

Supplement: Supplementary file 4 — Supplementary file4 (ZIP 111545 KB) [file 10822_2021_421_MOESM4_ESM.zip › 131/tp_decision_graphs/Delta-Rho.jpg]

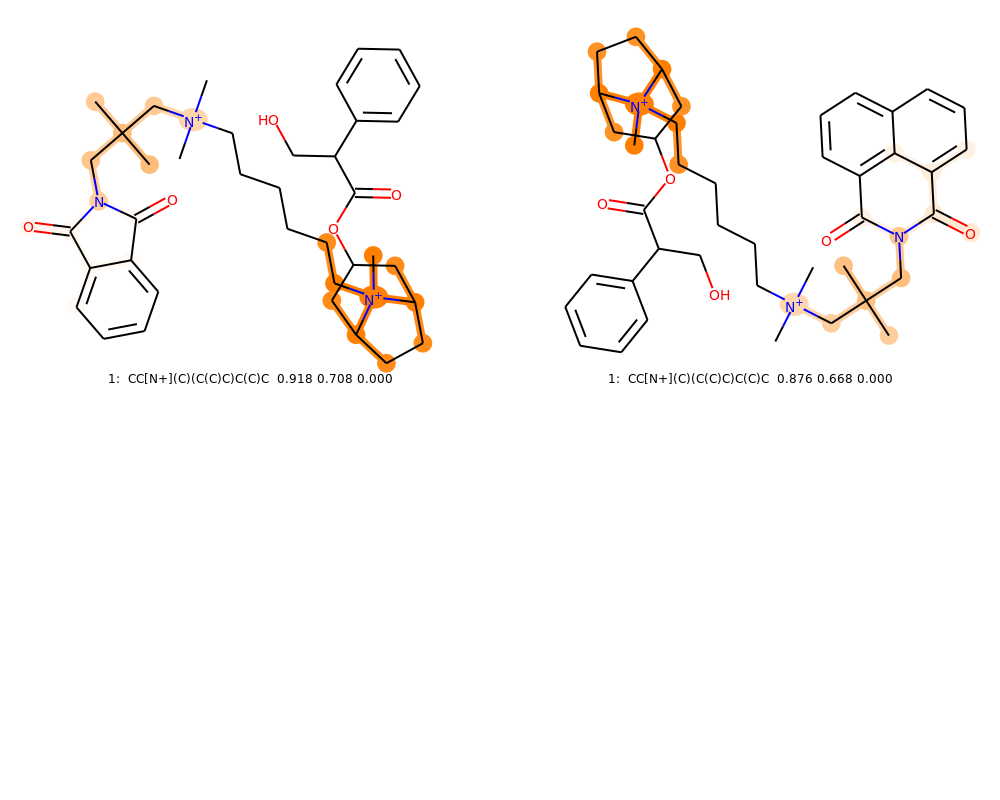

Supplement: Supplementary file 4 — Supplementary file4 (ZIP 111545 KB) [file 10822_2021_421_MOESM4_ESM.zip › 131/tp_cluster_1/tp1_mols_0.png]

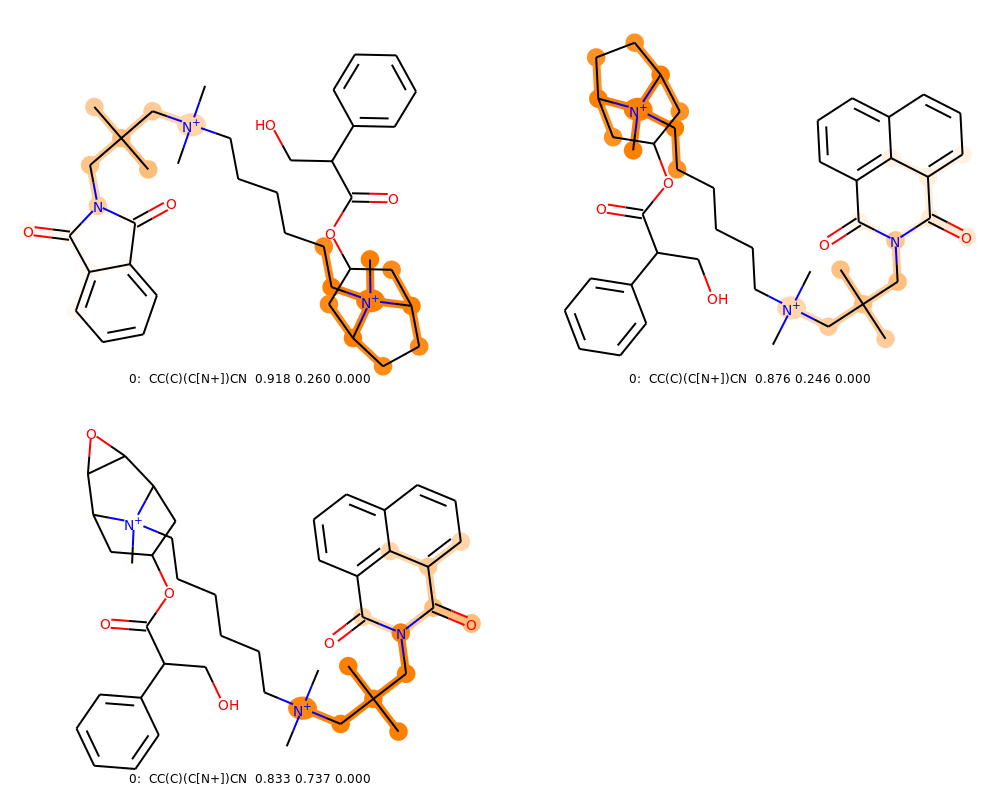

Supplement: Supplementary file 4 — Supplementary file4 (ZIP 111545 KB) [file 10822_2021_421_MOESM4_ESM.zip › 131/tp_cluster_0/tp0_mols_0.png]

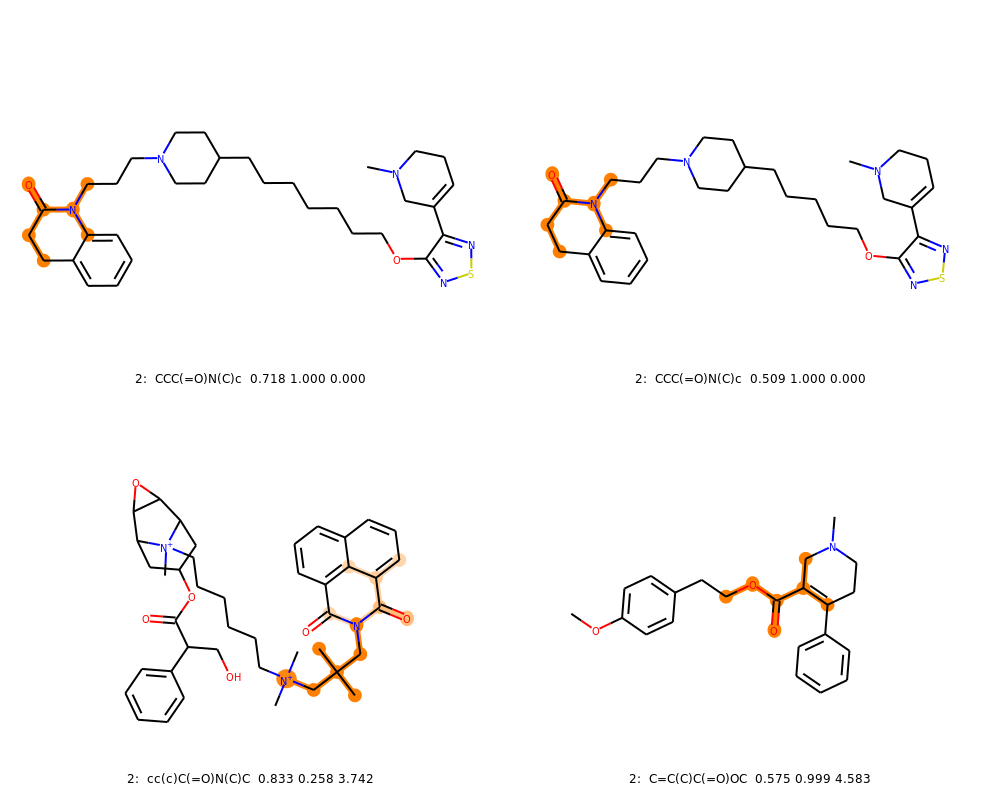

Supplement: Supplementary file 4 — Supplementary file4 (ZIP 111545 KB) [file 10822_2021_421_MOESM4_ESM.zip › 131/tp_cluster_2/tp2_mols_0.png]

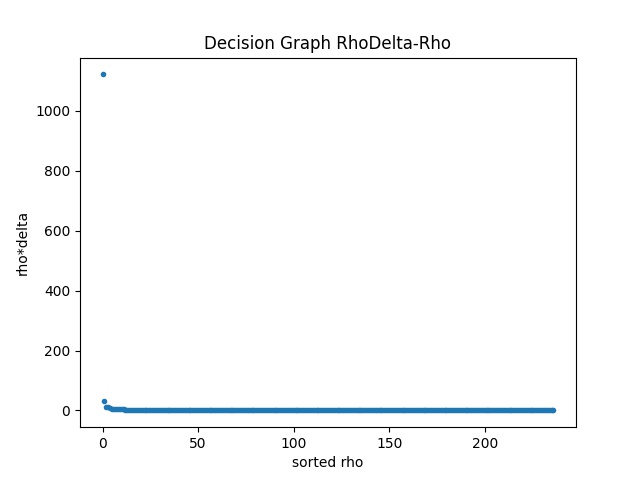

Supplement: Supplementary file 4 — Supplementary file4 (ZIP 111545 KB) [file 10822_2021_421_MOESM4_ESM.zip › 137/tp_decision_graphs/RhoDelta-Rho.jpg]

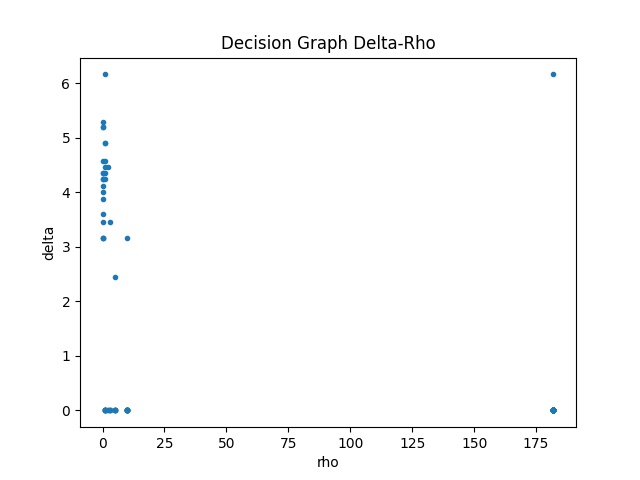

Supplement: Supplementary file 4 — Supplementary file4 (ZIP 111545 KB) [file 10822_2021_421_MOESM4_ESM.zip › 137/tp_decision_graphs/Delta-Rho.jpg]

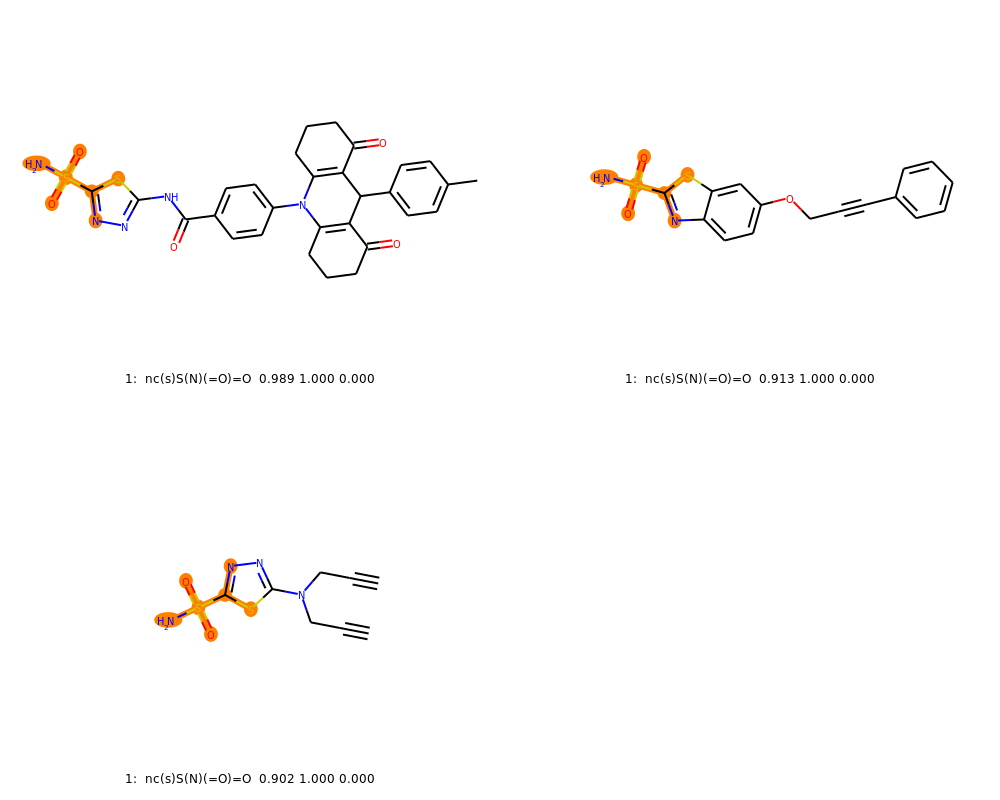

Supplement: Supplementary file 4 — Supplementary file4 (ZIP 111545 KB) [file 10822_2021_421_MOESM4_ESM.zip › 137/tp_cluster_1/tp1_mols_8.png]

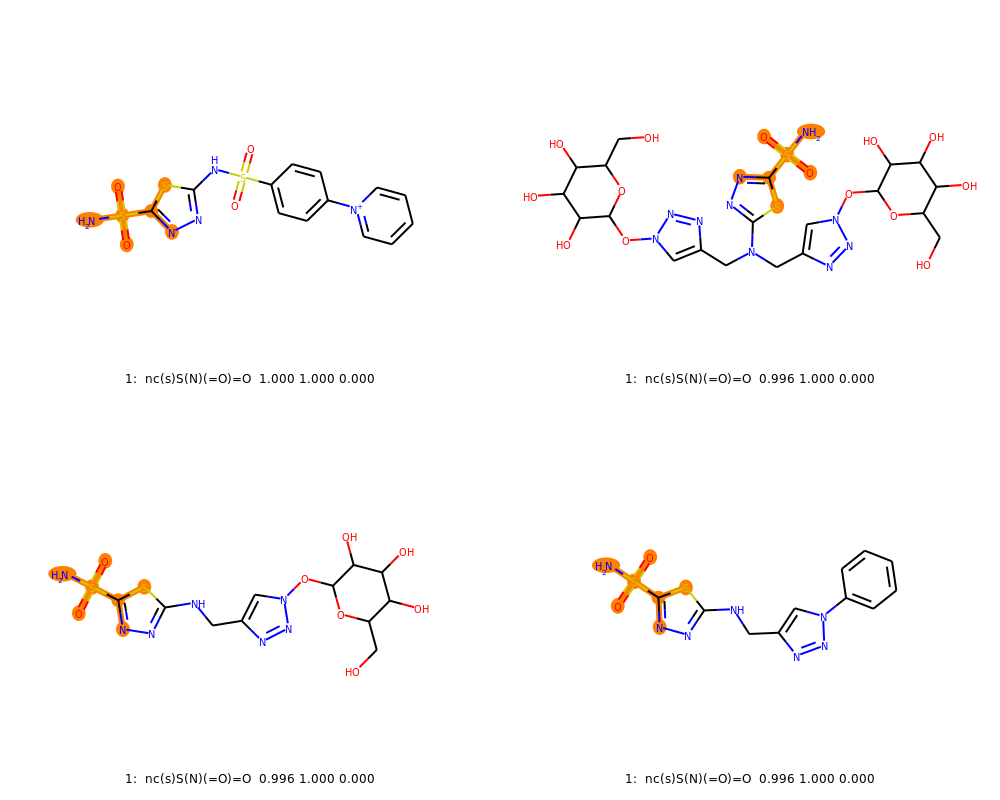

Supplement: Supplementary file 4 — Supplementary file4 (ZIP 111545 KB) [file 10822_2021_421_MOESM4_ESM.zip › 137/tp_cluster_1/tp1_mols_0.png]

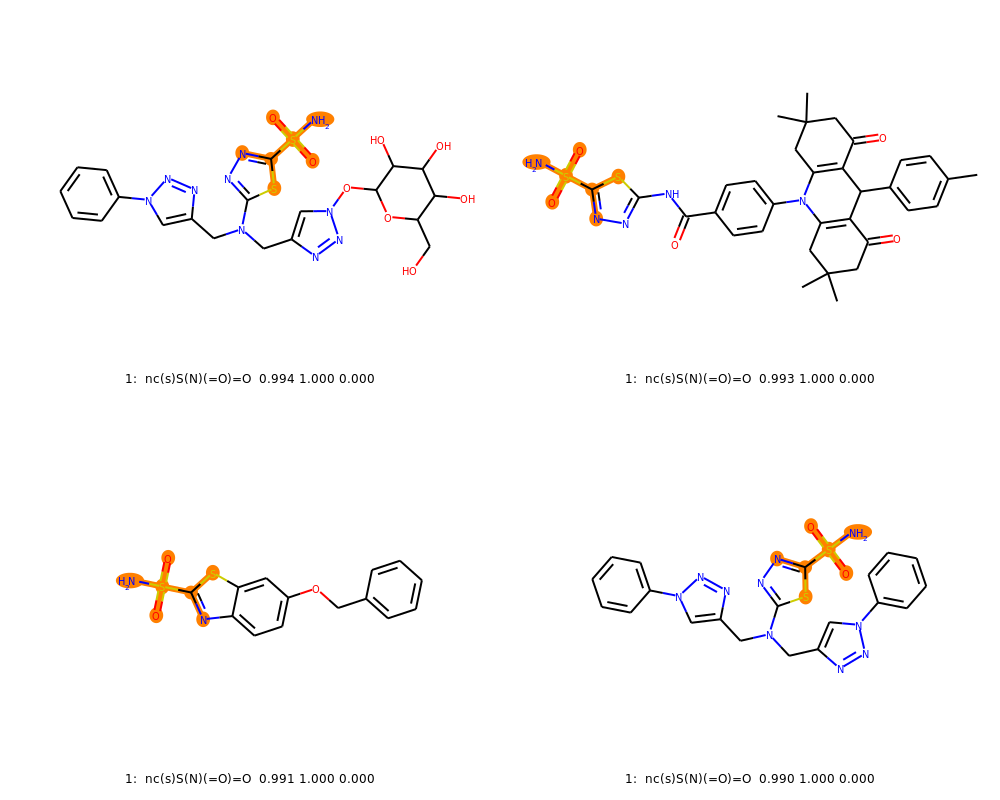

Supplement: Supplementary file 4 — Supplementary file4 (ZIP 111545 KB) [file 10822_2021_421_MOESM4_ESM.zip › 137/tp_cluster_1/tp1_mols_4.png]

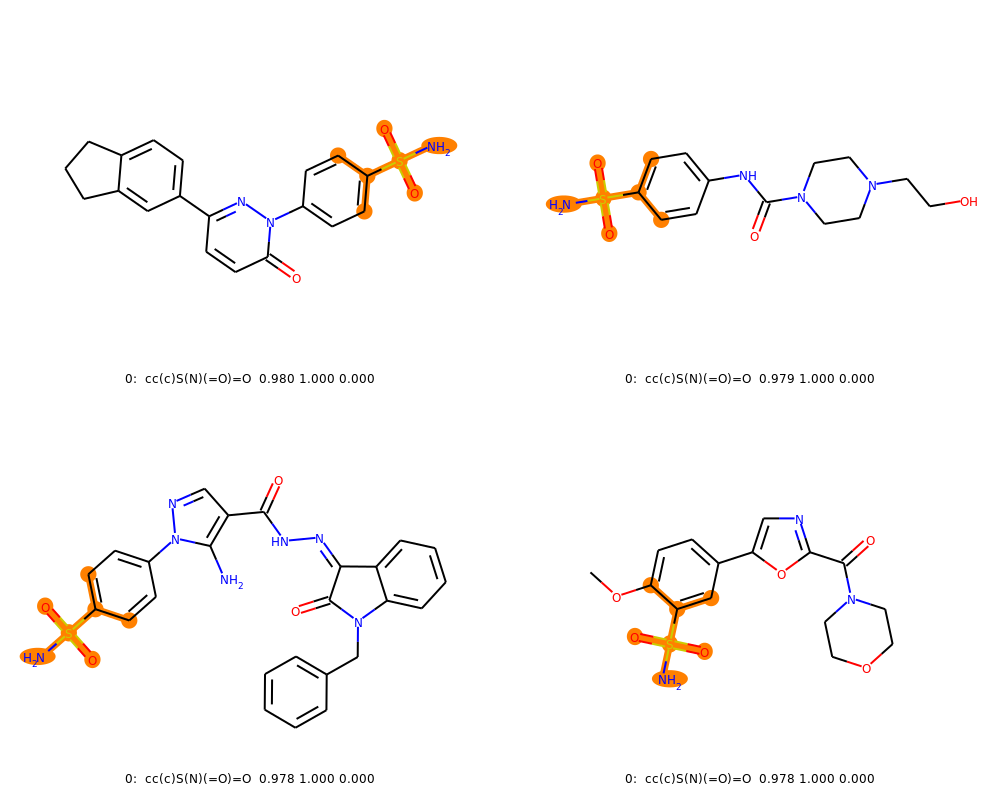

Supplement: Supplementary file 4 — Supplementary file4 (ZIP 111545 KB) [file 10822_2021_421_MOESM4_ESM.zip › 137/tp_cluster_0/tp0_mols_124.png]

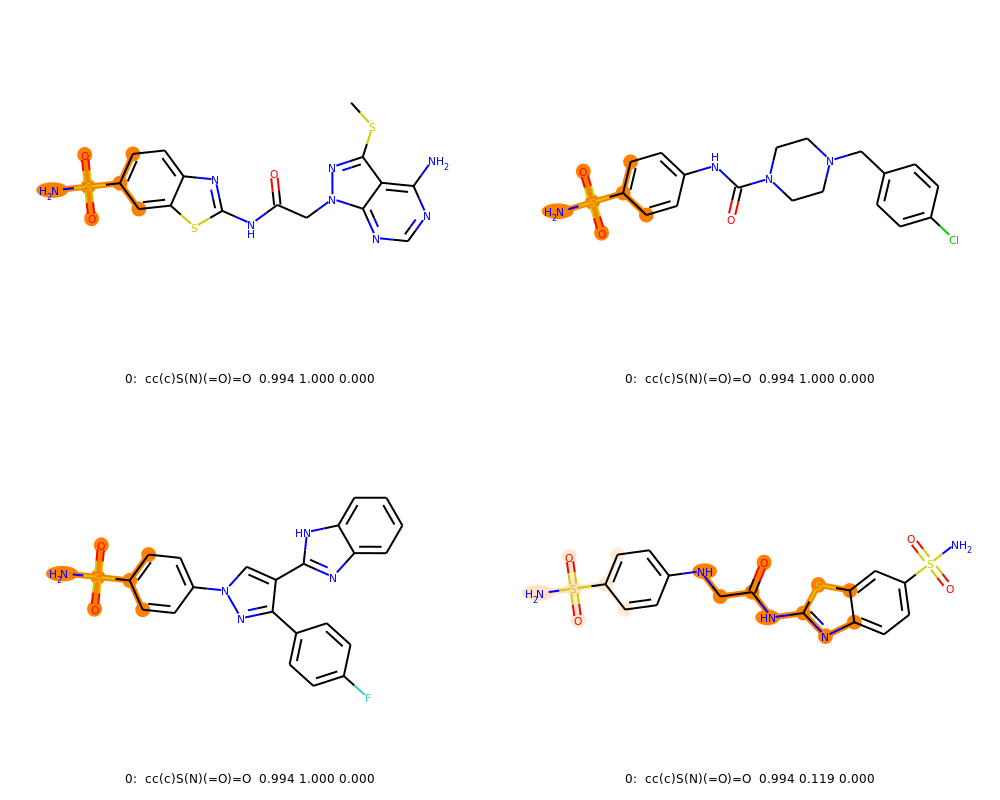

Supplement: Supplementary file 4 — Supplementary file4 (ZIP 111545 KB) [file 10822_2021_421_MOESM4_ESM.zip › 137/tp_cluster_0/tp0_mols_80.png]

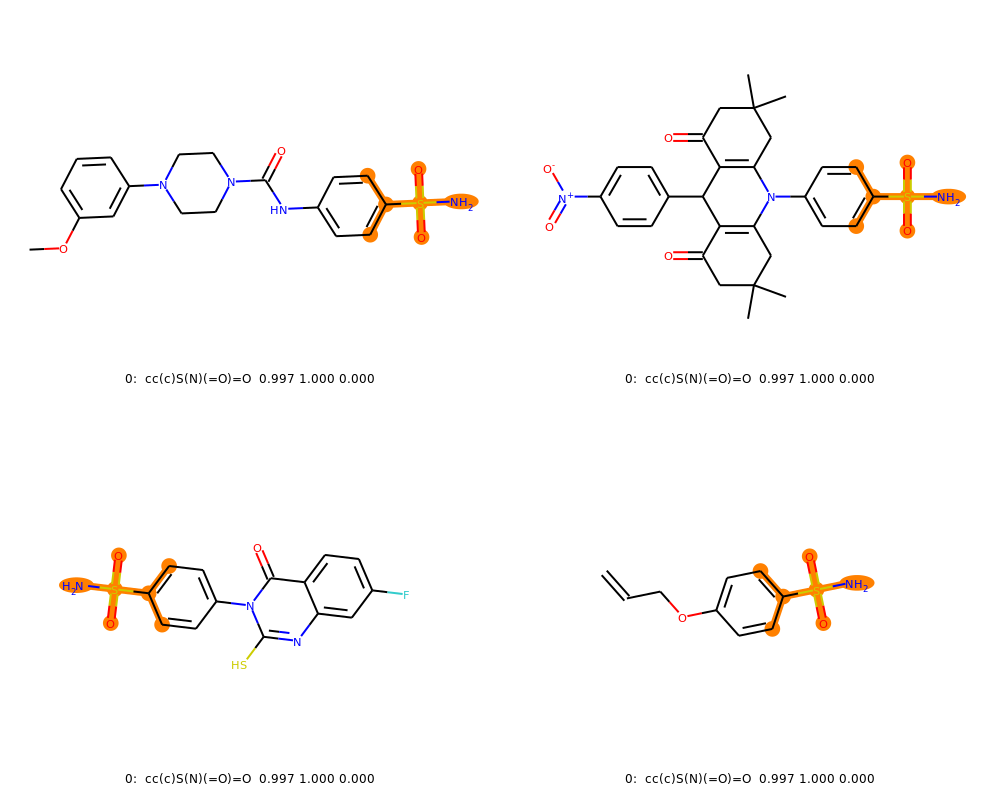

Supplement: Supplementary file 4 — Supplementary file4 (ZIP 111545 KB) [file 10822_2021_421_MOESM4_ESM.zip › 137/tp_cluster_0/tp0_mols_56.png]

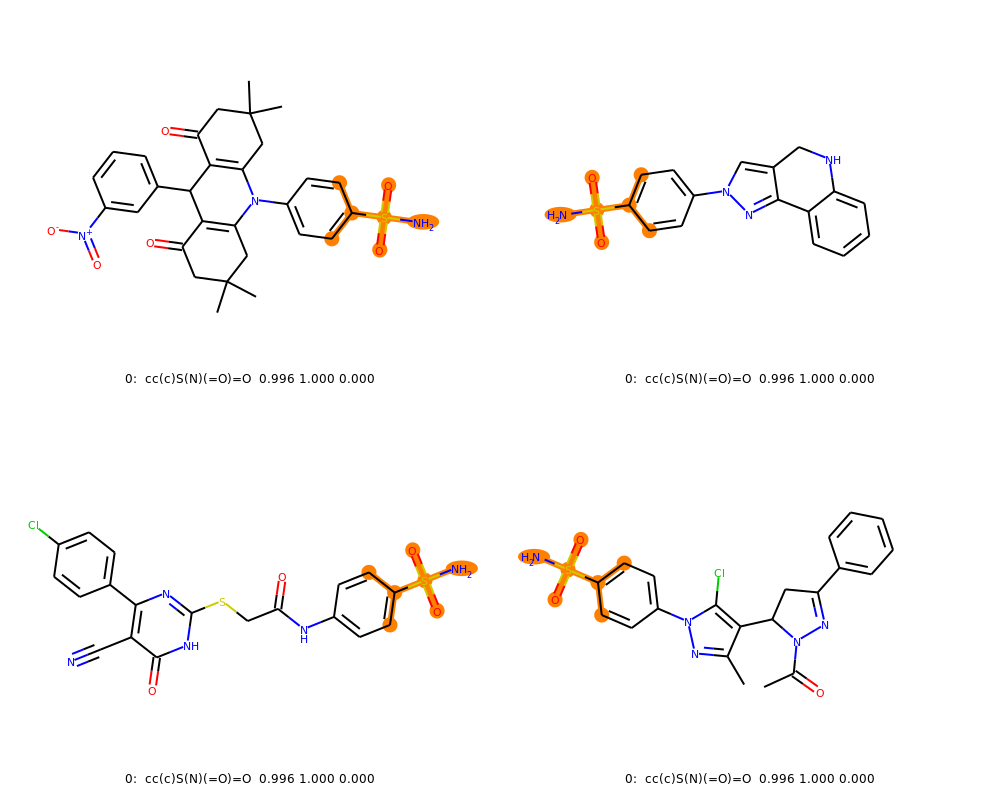

Supplement: Supplementary file 4 — Supplementary file4 (ZIP 111545 KB) [file 10822_2021_421_MOESM4_ESM.zip › 137/tp_cluster_0/tp0_mols_68.png]

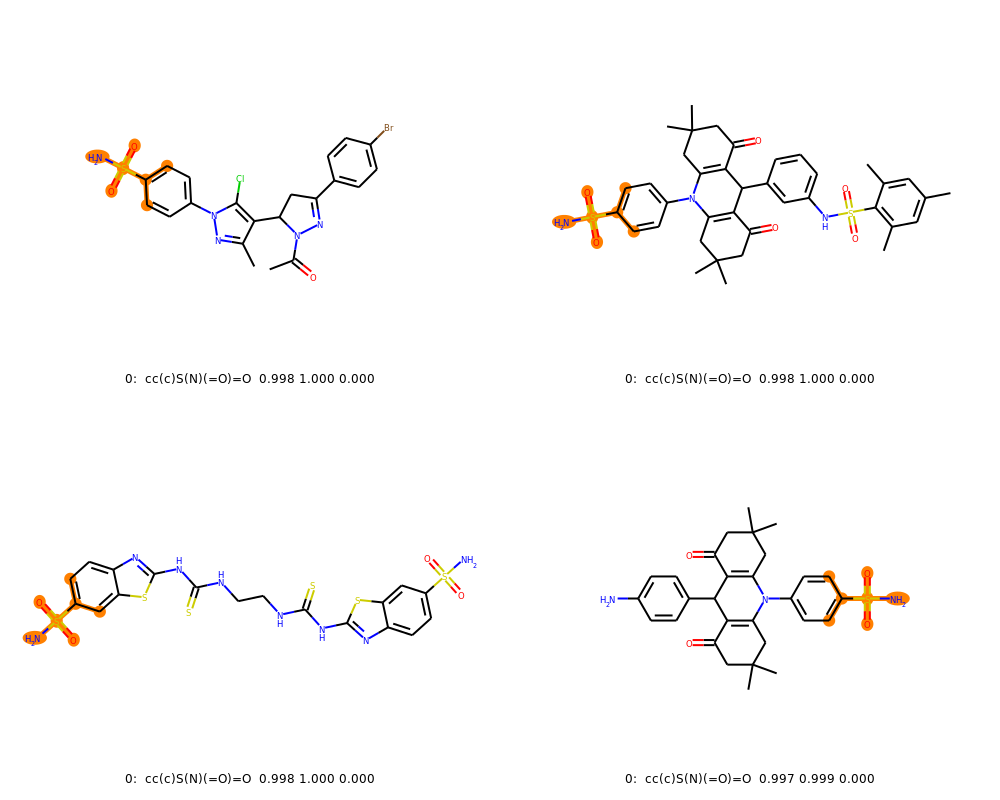

Supplement: Supplementary file 4 — Supplementary file4 (ZIP 111545 KB) [file 10822_2021_421_MOESM4_ESM.zip › 137/tp_cluster_0/tp0_mols_40.png]

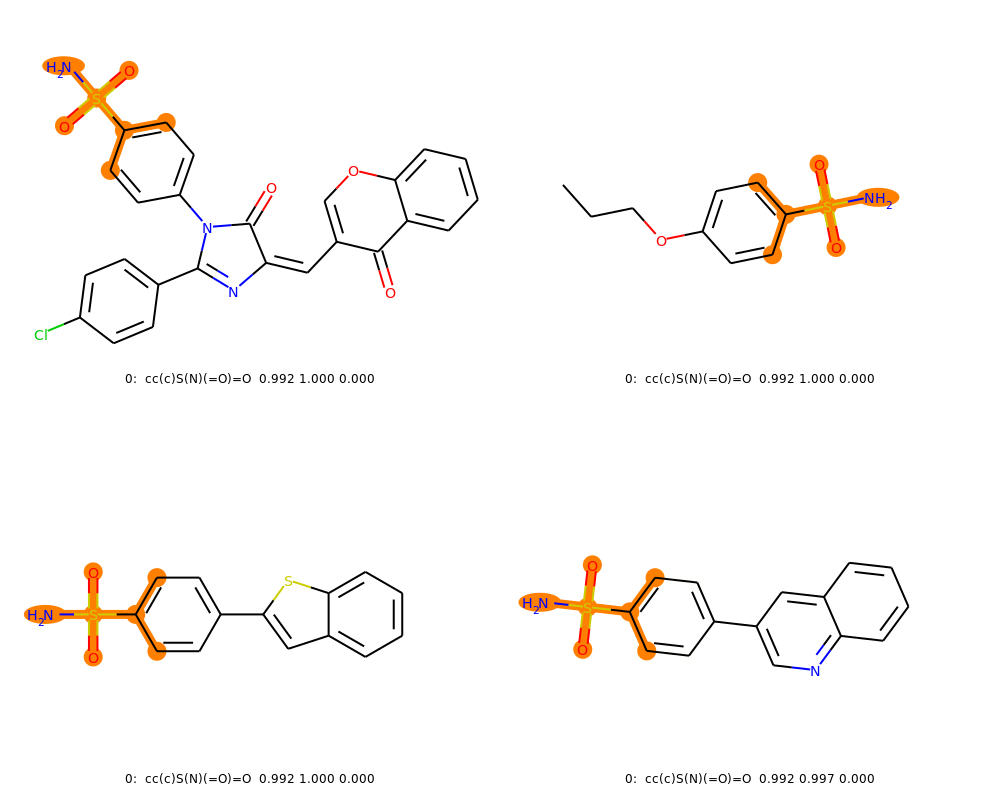

Supplement: Supplementary file 4 — Supplementary file4 (ZIP 111545 KB) [file 10822_2021_421_MOESM4_ESM.zip › 137/tp_cluster_0/tp0_mols_96.png]

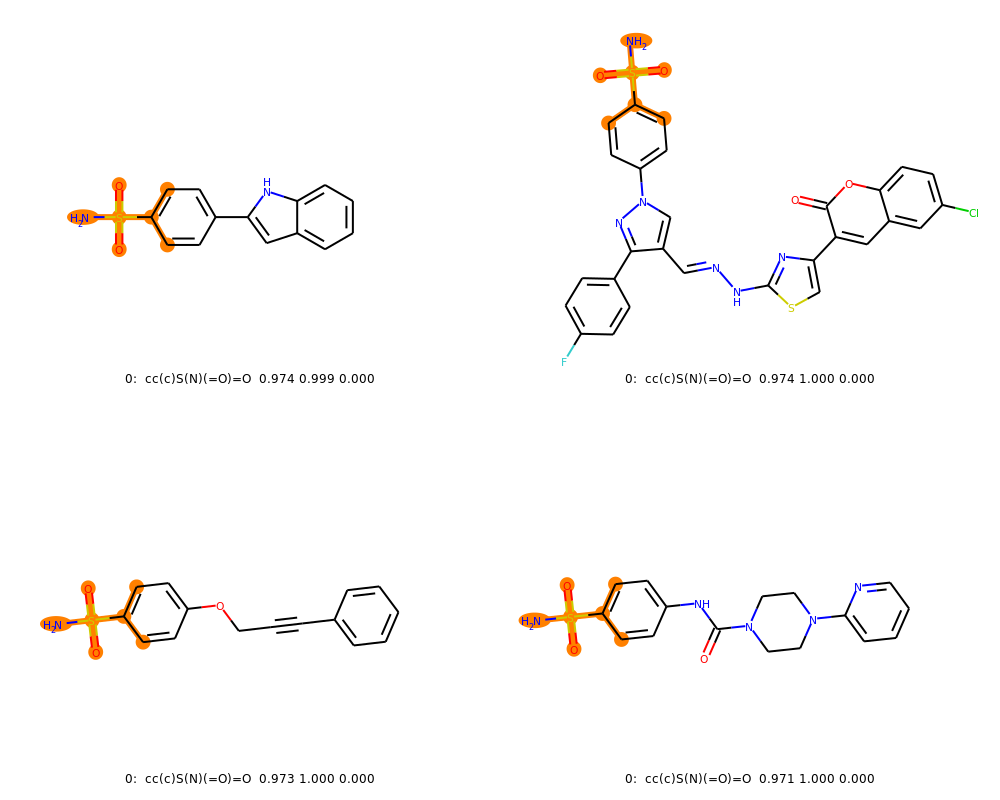

Supplement: Supplementary file 4 — Supplementary file4 (ZIP 111545 KB) [file 10822_2021_421_MOESM4_ESM.zip › 137/tp_cluster_0/tp0_mols_132.png]

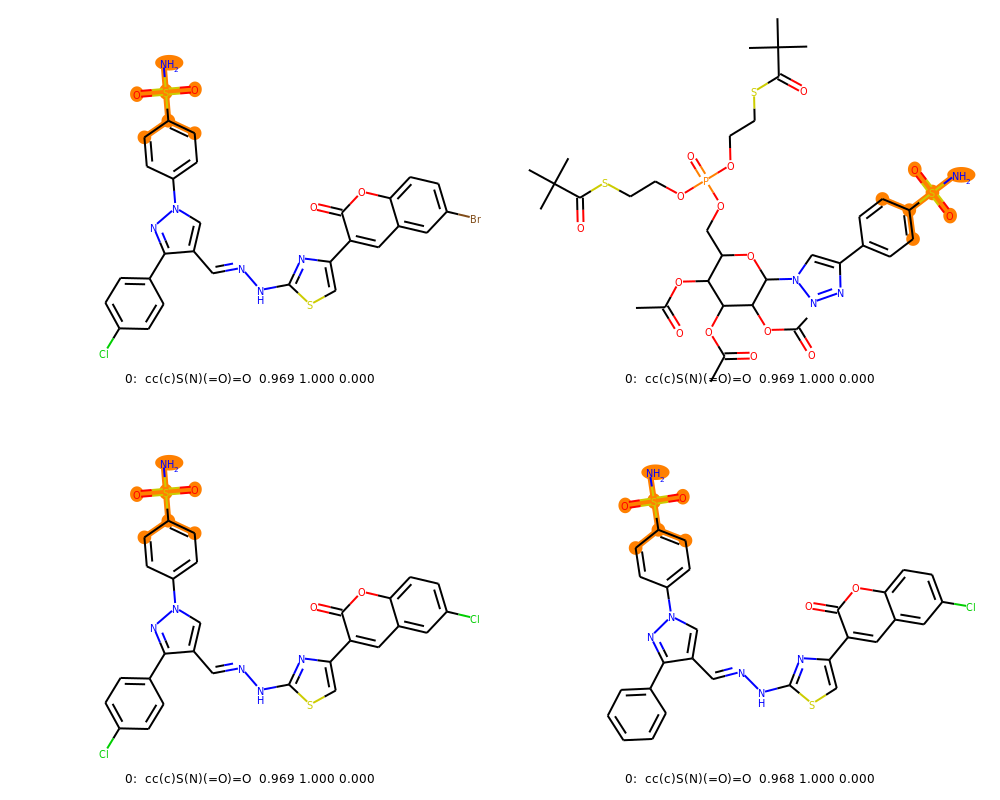

Supplement: Supplementary file 4 — Supplementary file4 (ZIP 111545 KB) [file 10822_2021_421_MOESM4_ESM.zip › 137/tp_cluster_0/tp0_mols_136.png]

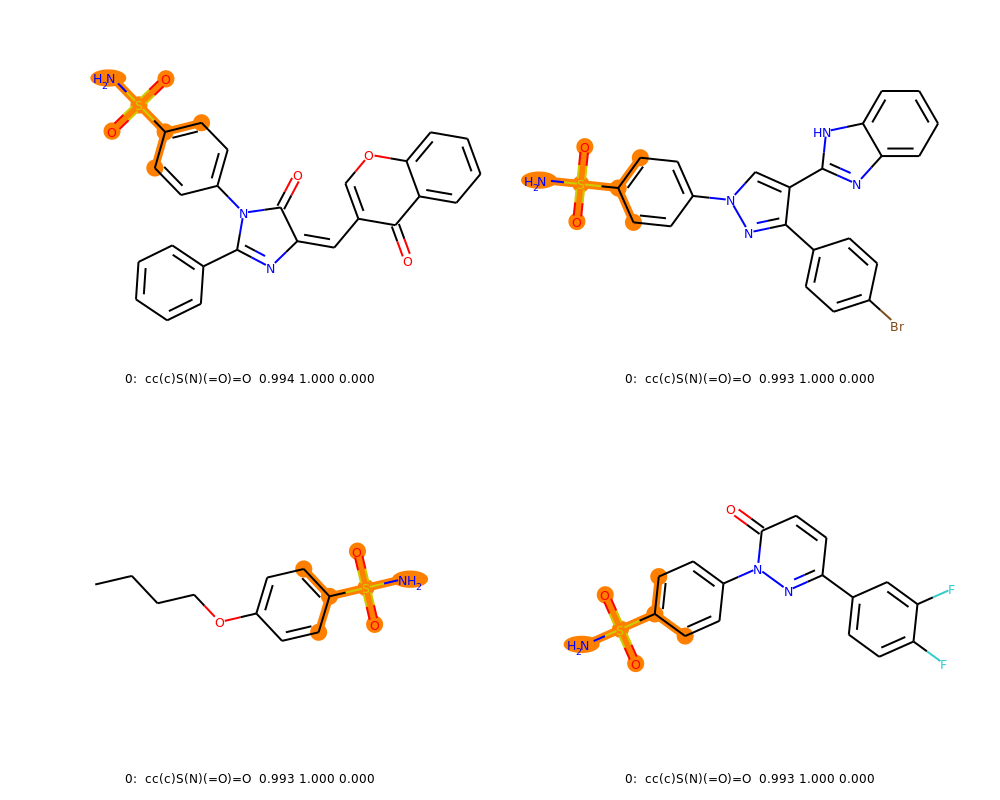

Supplement: Supplementary file 4 — Supplementary file4 (ZIP 111545 KB) [file 10822_2021_421_MOESM4_ESM.zip › 137/tp_cluster_0/tp0_mols_92.png]

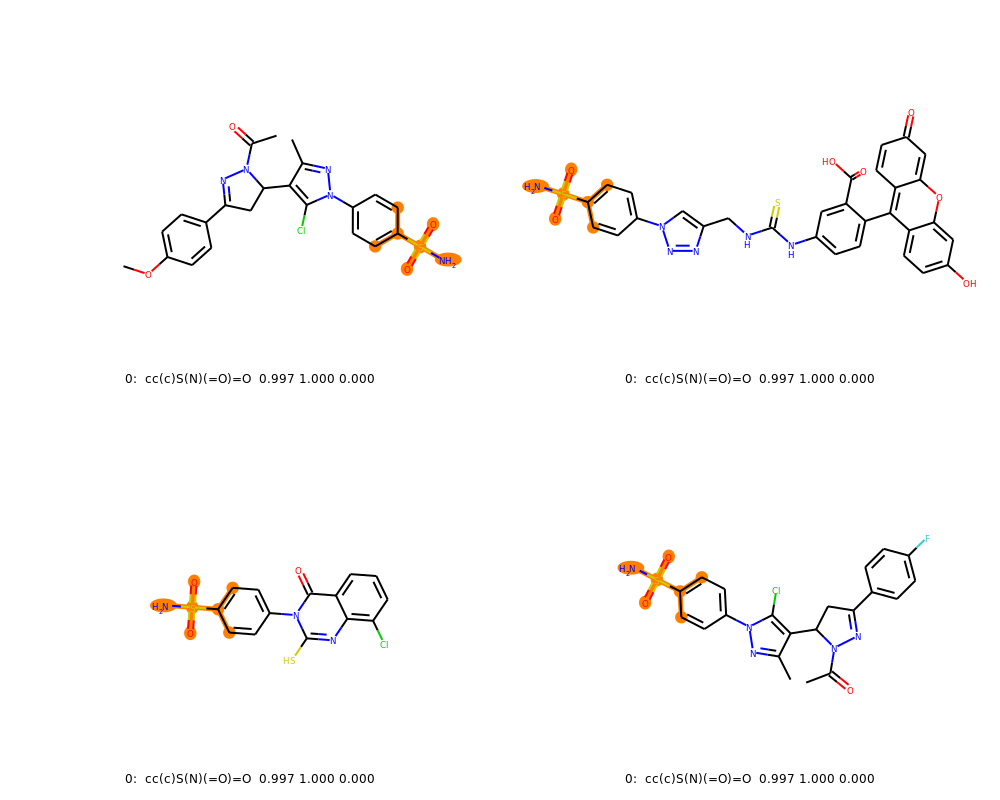

Supplement: Supplementary file 4 — Supplementary file4 (ZIP 111545 KB) [file 10822_2021_421_MOESM4_ESM.zip › 137/tp_cluster_0/tp0_mols_44.png]

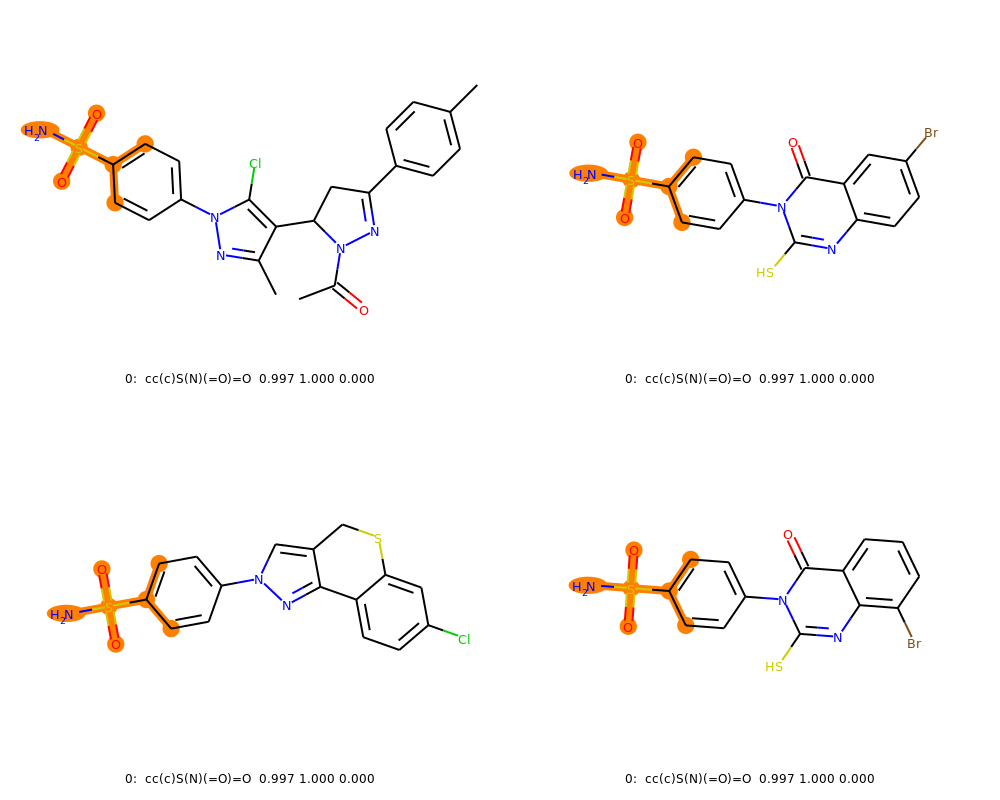

Supplement: Supplementary file 4 — Supplementary file4 (ZIP 111545 KB) [file 10822_2021_421_MOESM4_ESM.zip › 137/tp_cluster_0/tp0_mols_52.png]

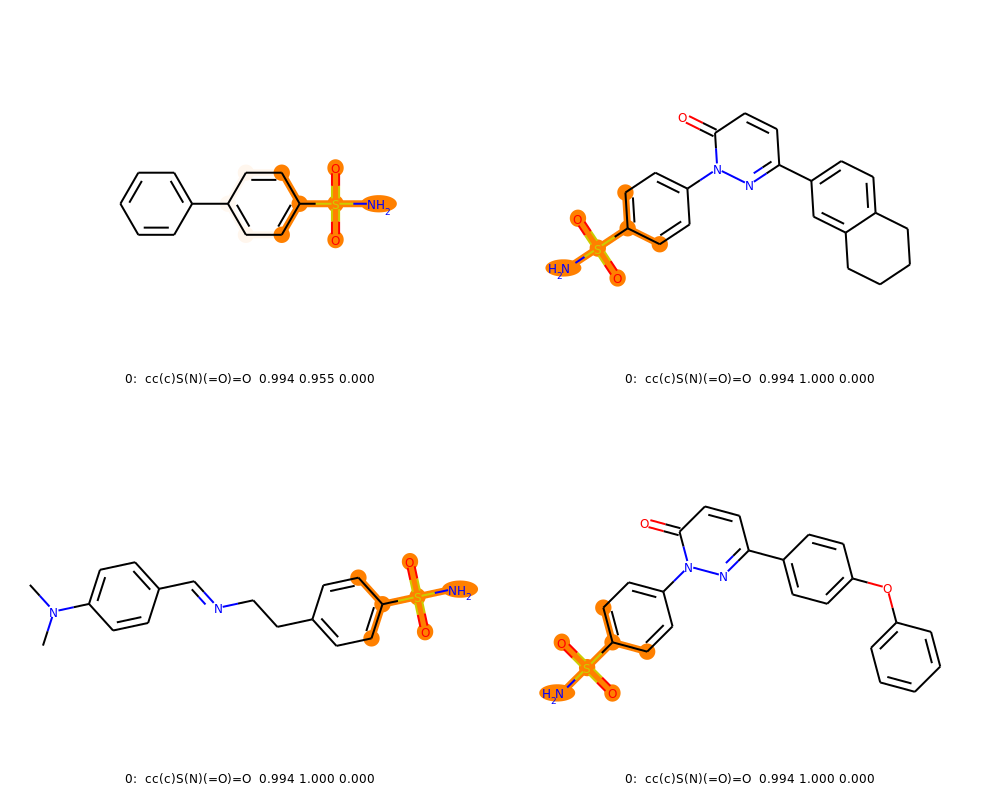

Supplement: Supplementary file 4 — Supplementary file4 (ZIP 111545 KB) [file 10822_2021_421_MOESM4_ESM.zip › 137/tp_cluster_0/tp0_mols_84.png]

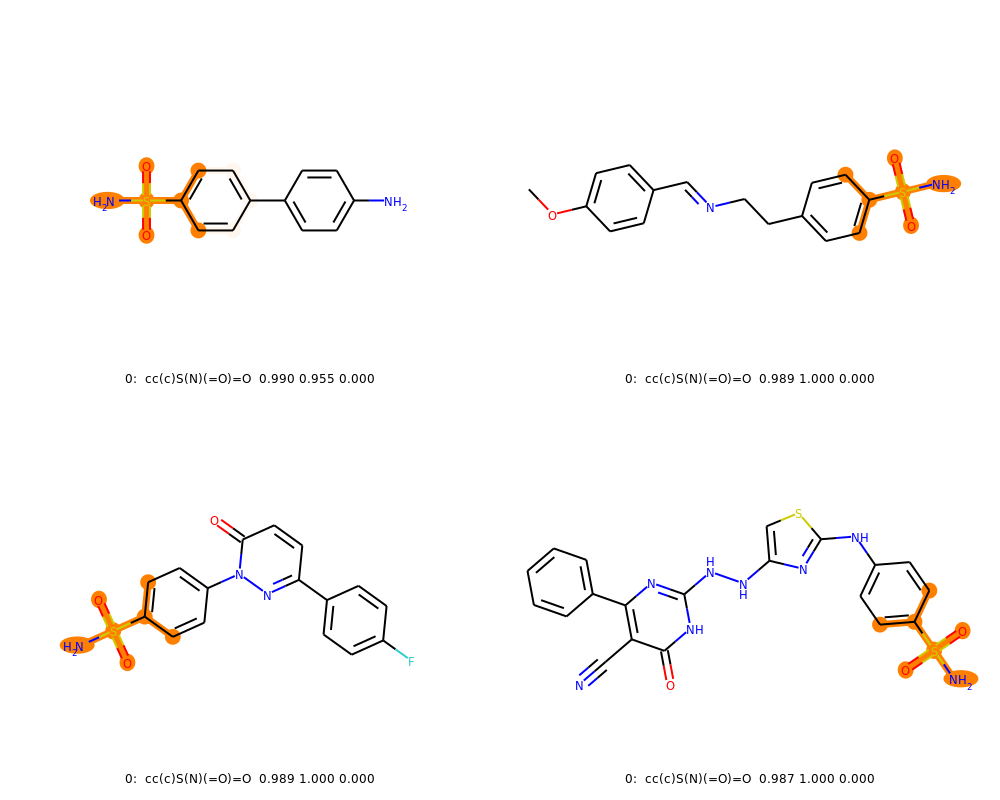

Supplement: Supplementary file 4 — Supplementary file4 (ZIP 111545 KB) [file 10822_2021_421_MOESM4_ESM.zip › 137/tp_cluster_0/tp0_mols_108.png]

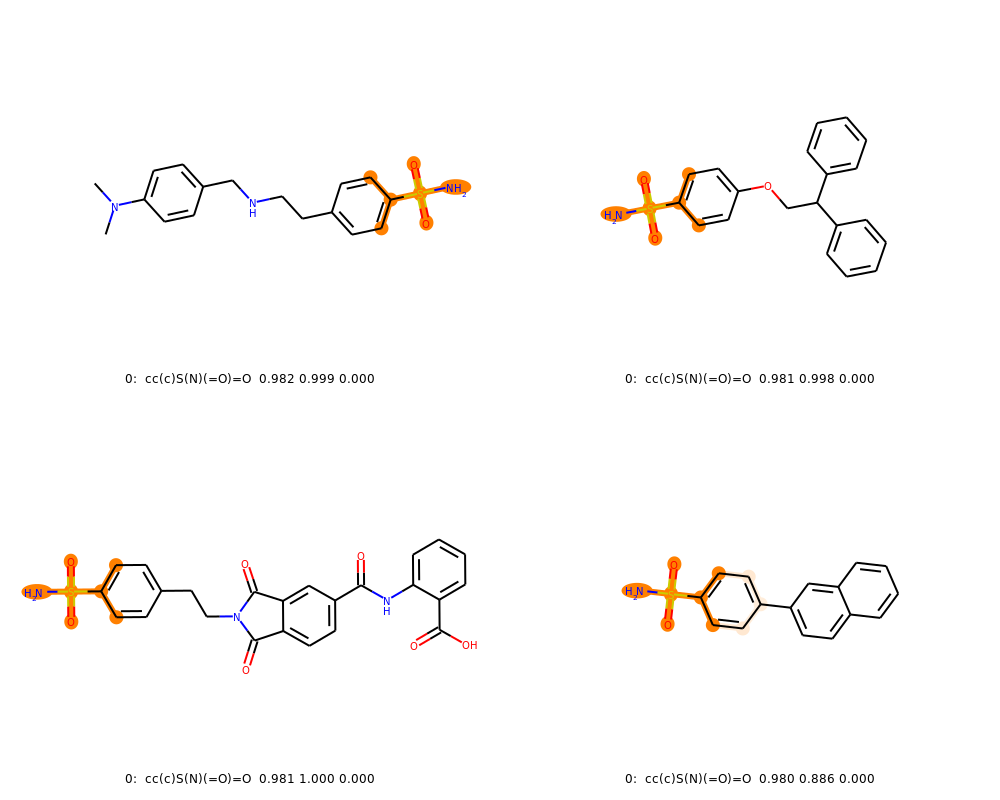

Supplement: Supplementary file 4 — Supplementary file4 (ZIP 111545 KB) [file 10822_2021_421_MOESM4_ESM.zip › 137/tp_cluster_0/tp0_mols_120.png]

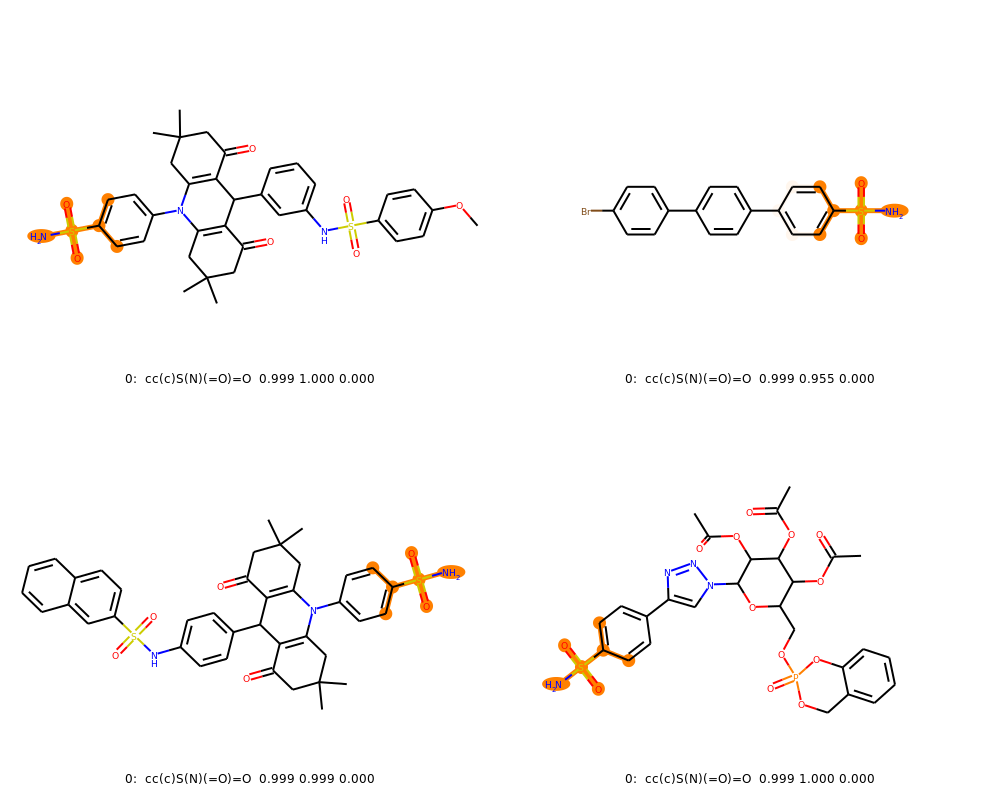

Supplement: Supplementary file 4 — Supplementary file4 (ZIP 111545 KB) [file 10822_2021_421_MOESM4_ESM.zip › 137/tp_cluster_0/tp0_mols_20.png]

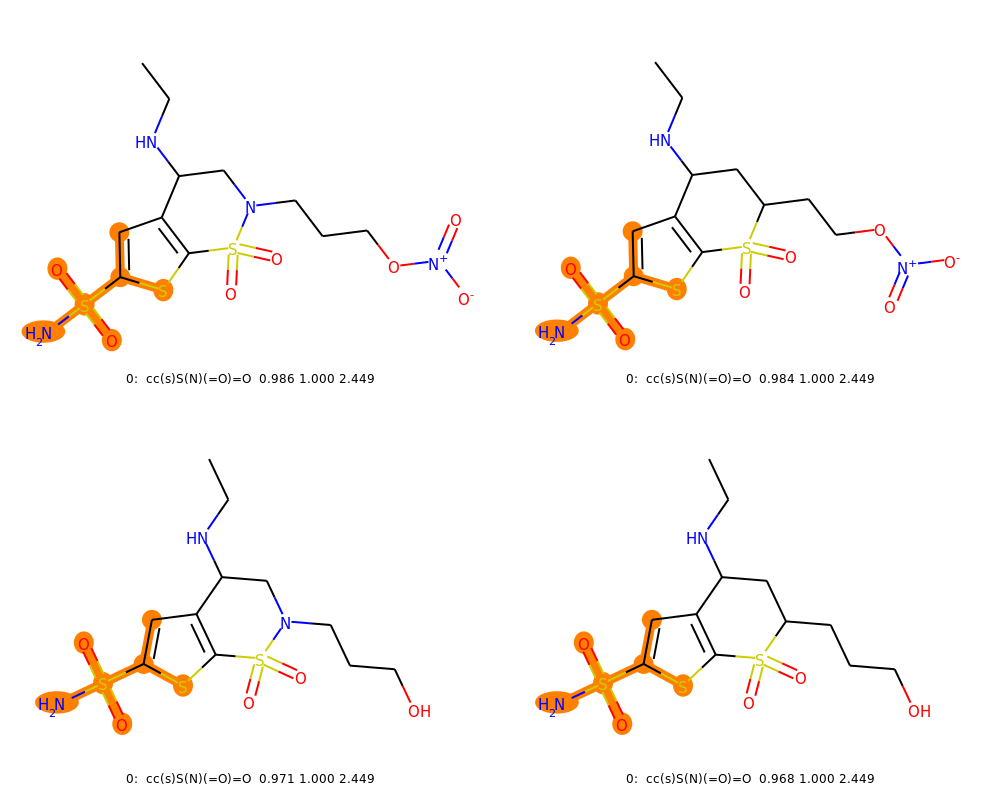

Supplement: Supplementary file 4 — Supplementary file4 (ZIP 111545 KB) [file 10822_2021_421_MOESM4_ESM.zip › 137/tp_cluster_0/tp0_mols_184.png]

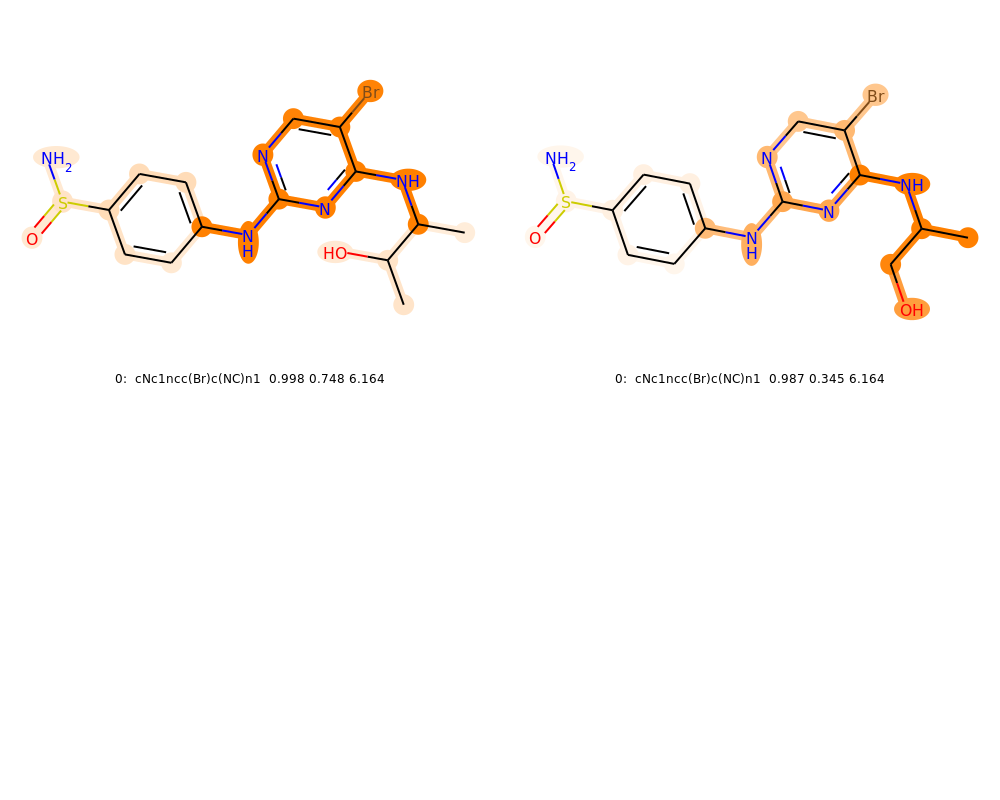

Supplement: Supplementary file 4 — Supplementary file4 (ZIP 111545 KB) [file 10822_2021_421_MOESM4_ESM.zip › 137/tp_cluster_0/tp0_mols_224.png]

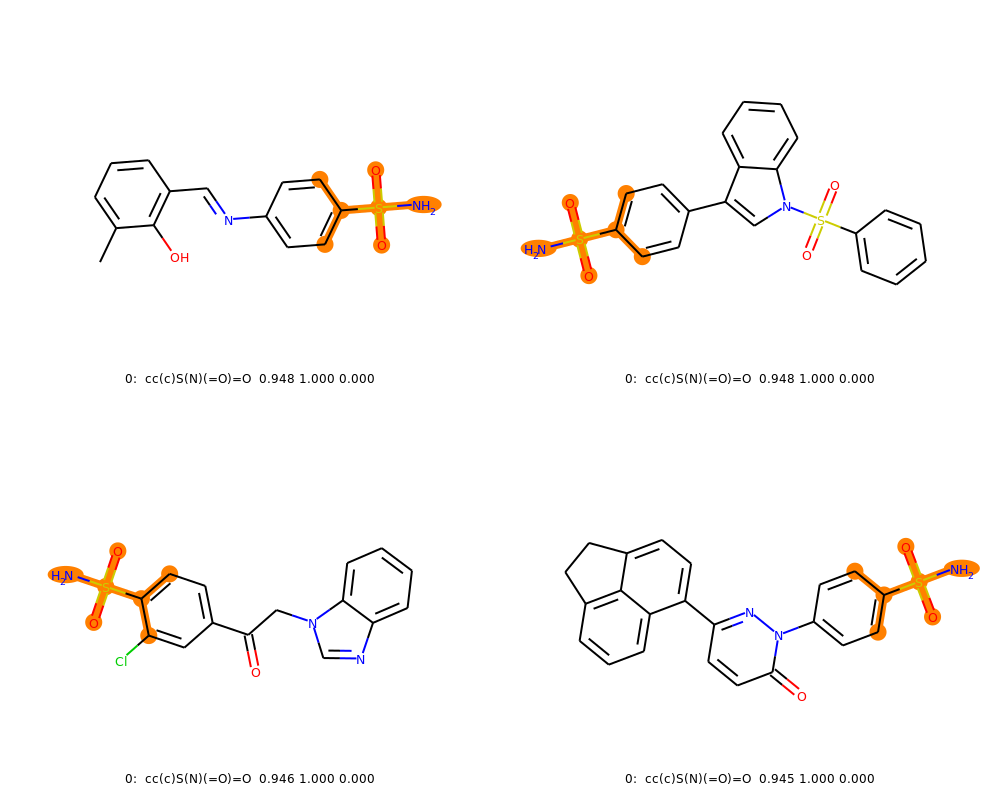

Supplement: Supplementary file 4 — Supplementary file4 (ZIP 111545 KB) [file 10822_2021_421_MOESM4_ESM.zip › 137/tp_cluster_0/tp0_mols_152.png]

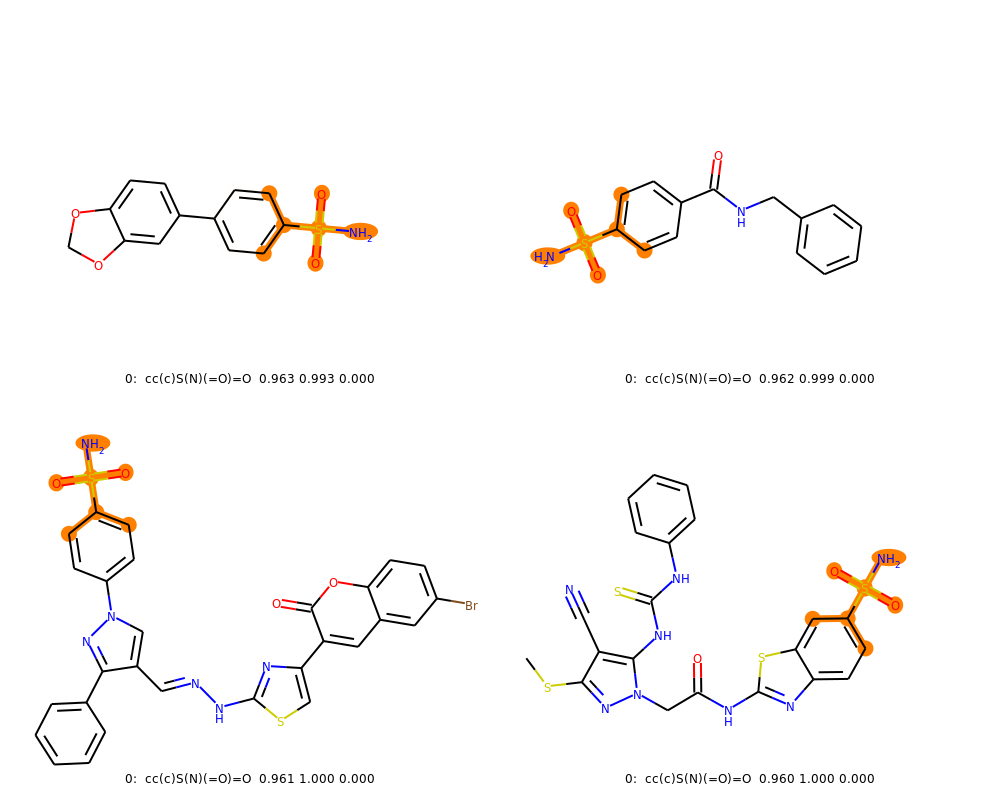

Supplement: Supplementary file 4 — Supplementary file4 (ZIP 111545 KB) [file 10822_2021_421_MOESM4_ESM.zip › 137/tp_cluster_0/tp0_mols_144.png]

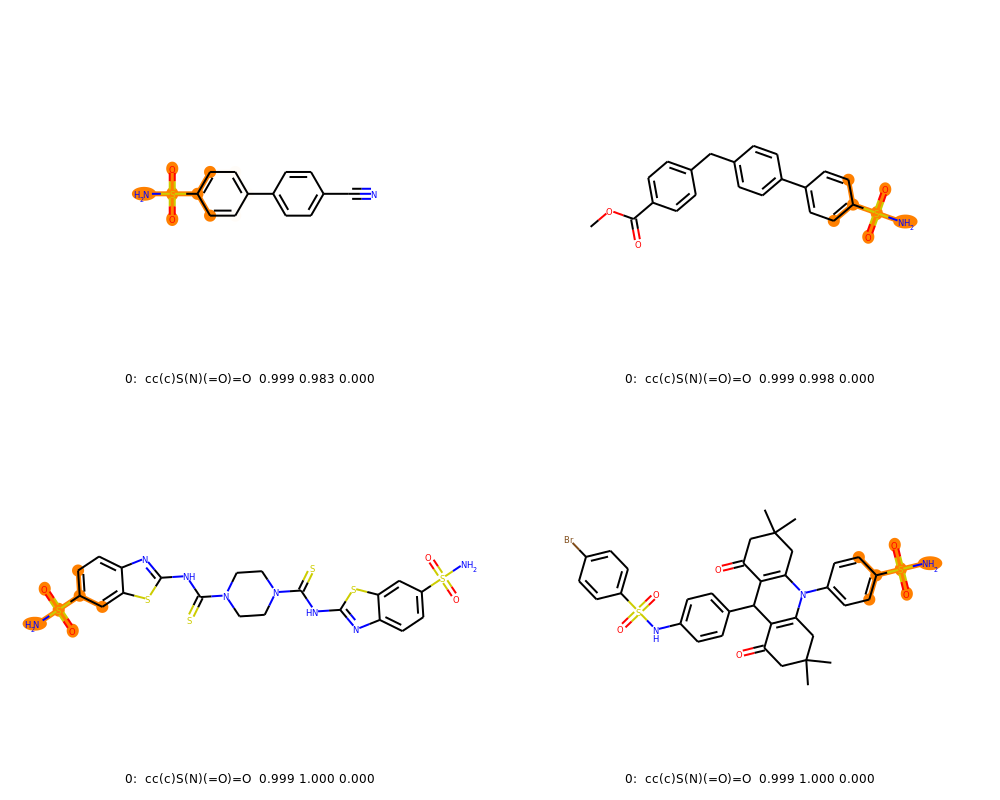

Supplement: Supplementary file 4 — Supplementary file4 (ZIP 111545 KB) [file 10822_2021_421_MOESM4_ESM.zip › 137/tp_cluster_0/tp0_mols_4.png]
