# Supplementary figures and images for: Simplified, interpretable graph convolutional neural networks for small molecule activity prediction (part 1 of 2)
Source: J Comput Aided Mol Des. 2021 Nov 24;36(5):391–404. doi: 10.1007/s10822-021-00421-6 (PMC9325818; doi:10.1007/s10822-021-00421-6)

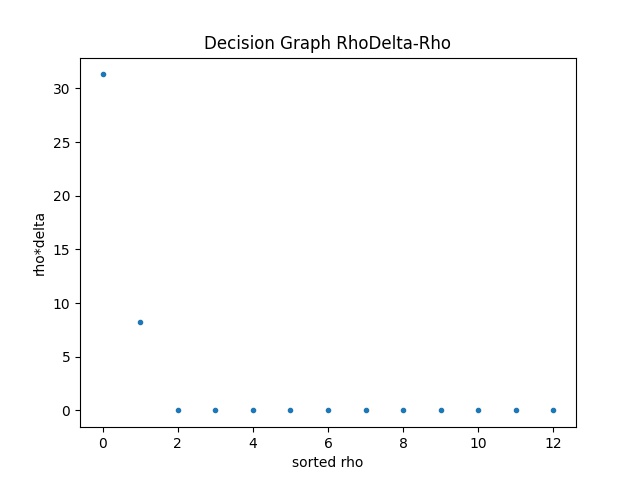

Supplement: Supplementary file 4 — Supplementary file4 (ZIP 111545 KB) [file 10822_2021_421_MOESM4_ESM.zip › 028/tp_decision_graphs/RhoDelta-Rho.jpg]

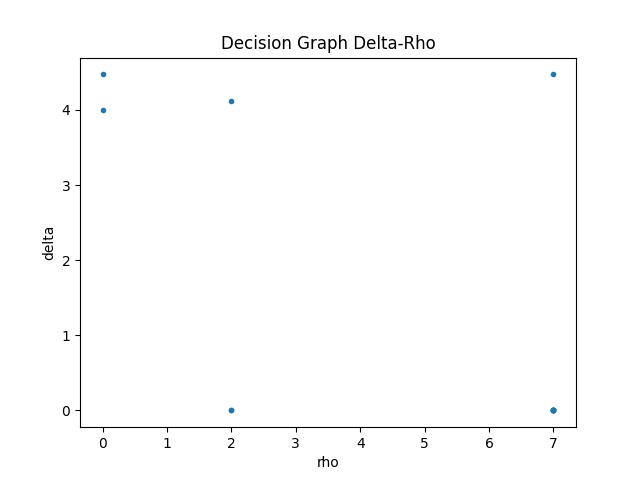

Supplement: Supplementary file 4 — Supplementary file4 (ZIP 111545 KB) [file 10822_2021_421_MOESM4_ESM.zip › 028/tp_decision_graphs/Delta-Rho.jpg]

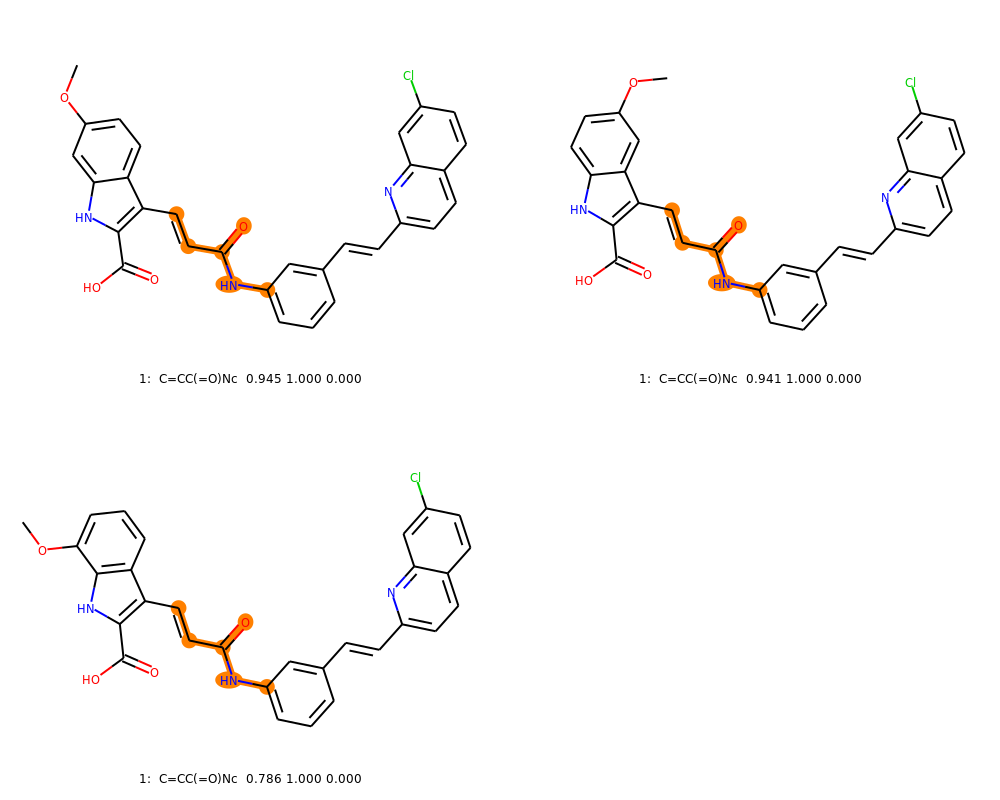

Supplement: Supplementary file 4 — Supplementary file4 (ZIP 111545 KB) [file 10822_2021_421_MOESM4_ESM.zip › 028/tp_cluster_1/tp1_mols_0.png]

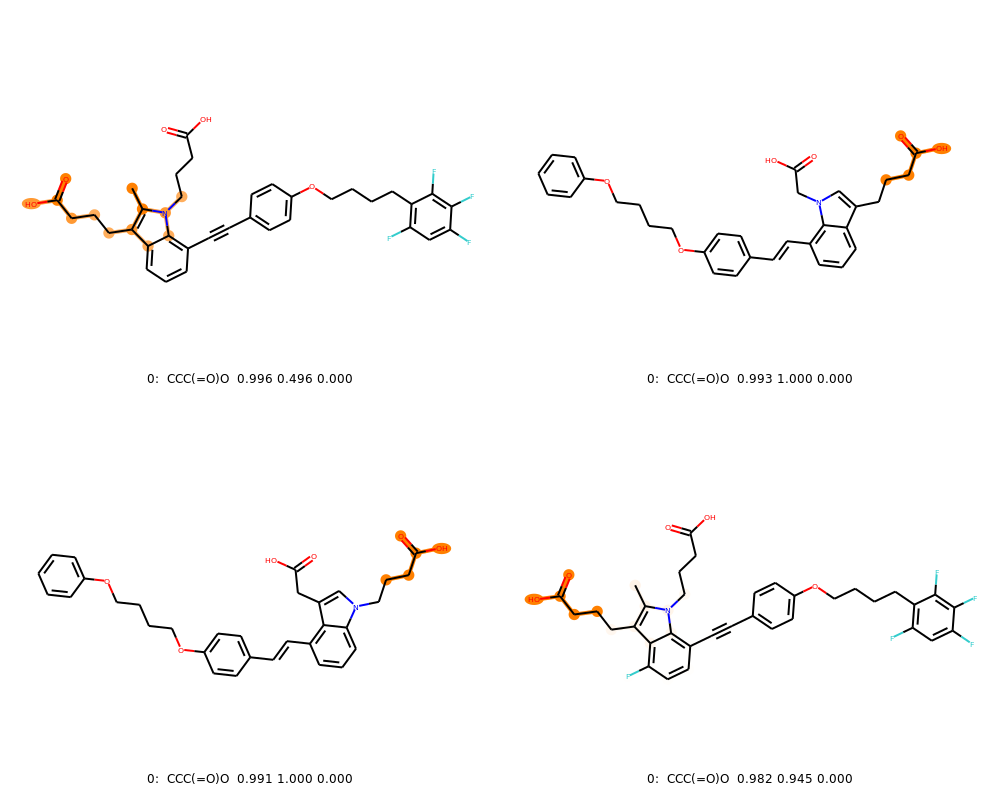

Supplement: Supplementary file 4 — Supplementary file4 (ZIP 111545 KB) [file 10822_2021_421_MOESM4_ESM.zip › 028/tp_cluster_0/tp0_mols_4.png]

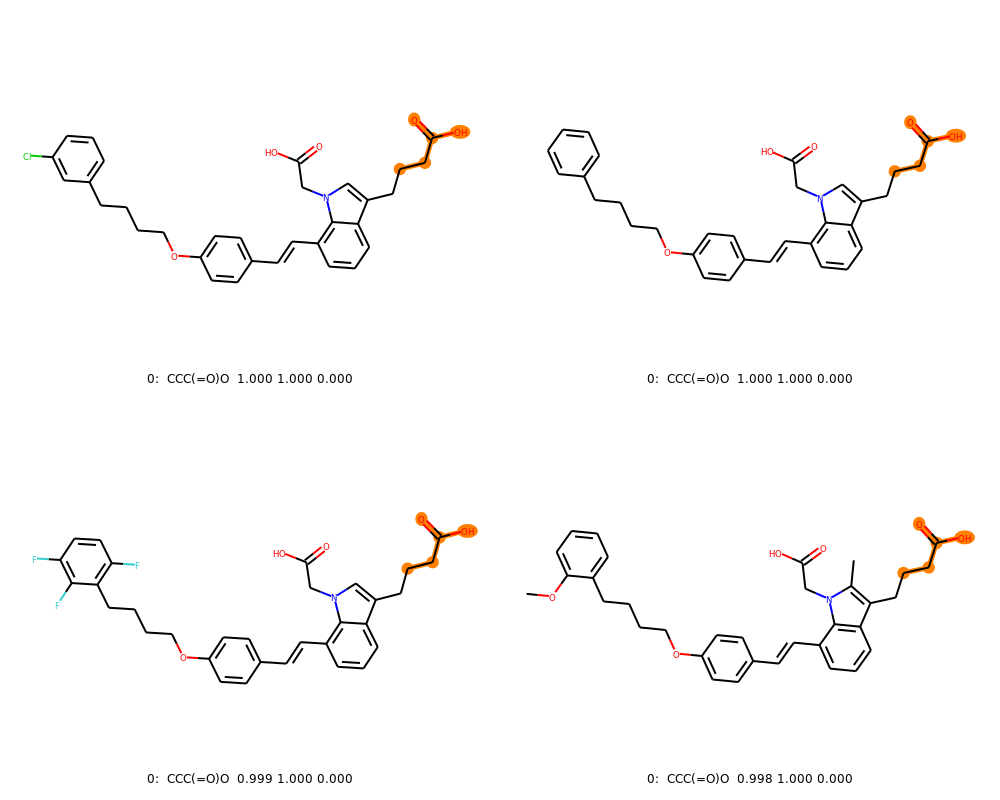

Supplement: Supplementary file 4 — Supplementary file4 (ZIP 111545 KB) [file 10822_2021_421_MOESM4_ESM.zip › 028/tp_cluster_0/tp0_mols_0.png]

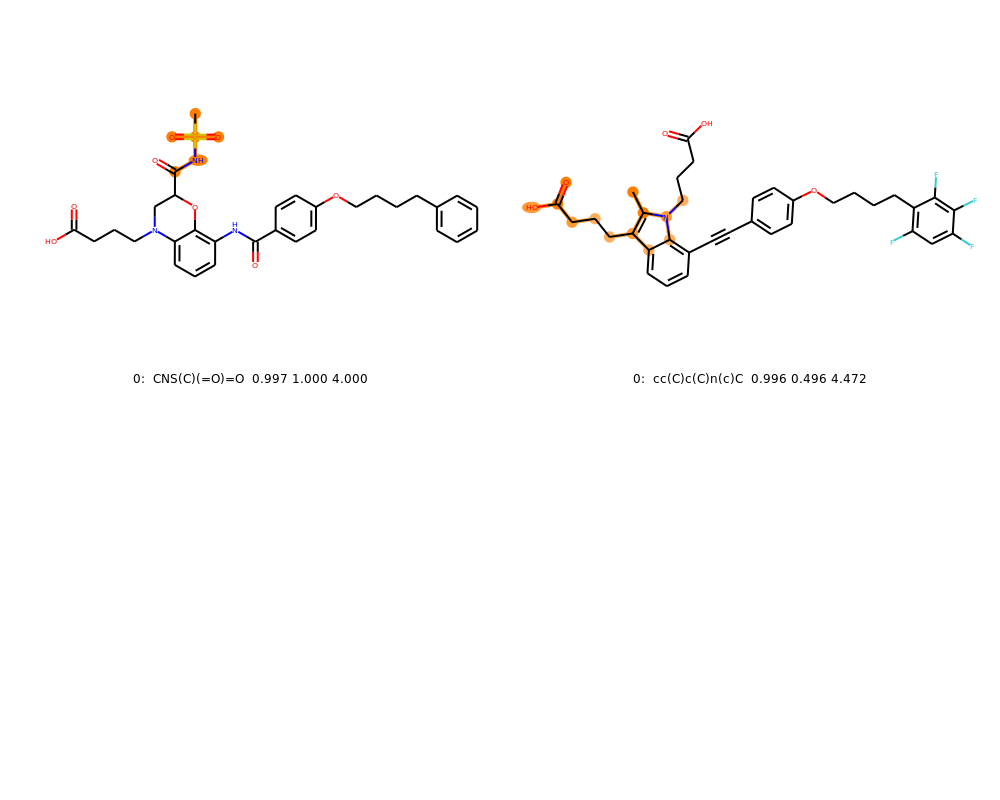

Supplement: Supplementary file 4 — Supplementary file4 (ZIP 111545 KB) [file 10822_2021_421_MOESM4_ESM.zip › 028/tp_cluster_0/tp0_mols_8.png]

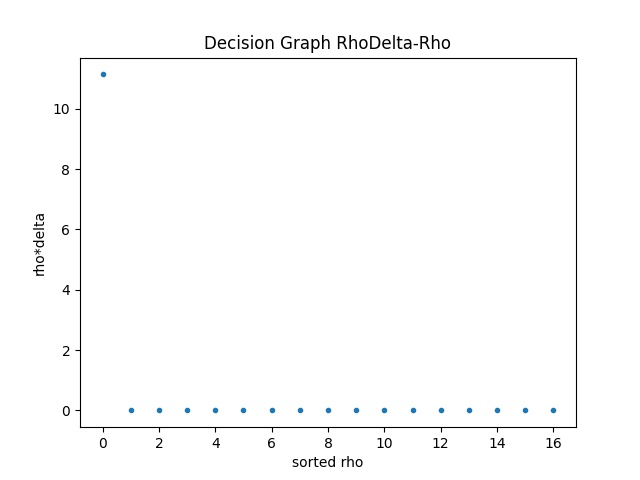

Supplement: Supplementary file 4 — Supplementary file4 (ZIP 111545 KB) [file 10822_2021_421_MOESM4_ESM.zip › 036/tp_decision_graphs/RhoDelta-Rho.jpg]

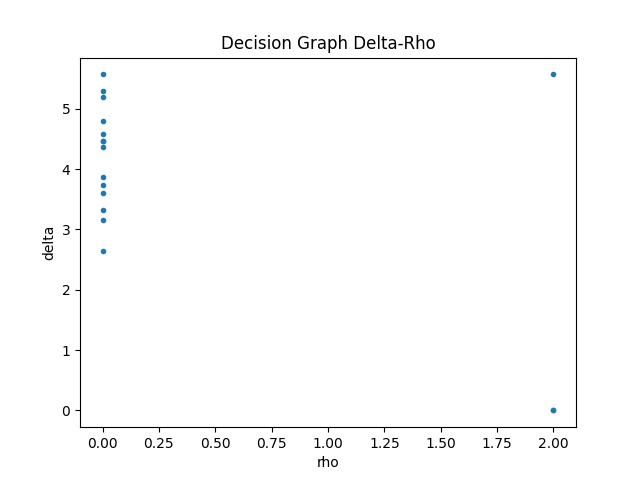

Supplement: Supplementary file 4 — Supplementary file4 (ZIP 111545 KB) [file 10822_2021_421_MOESM4_ESM.zip › 036/tp_decision_graphs/Delta-Rho.jpg]

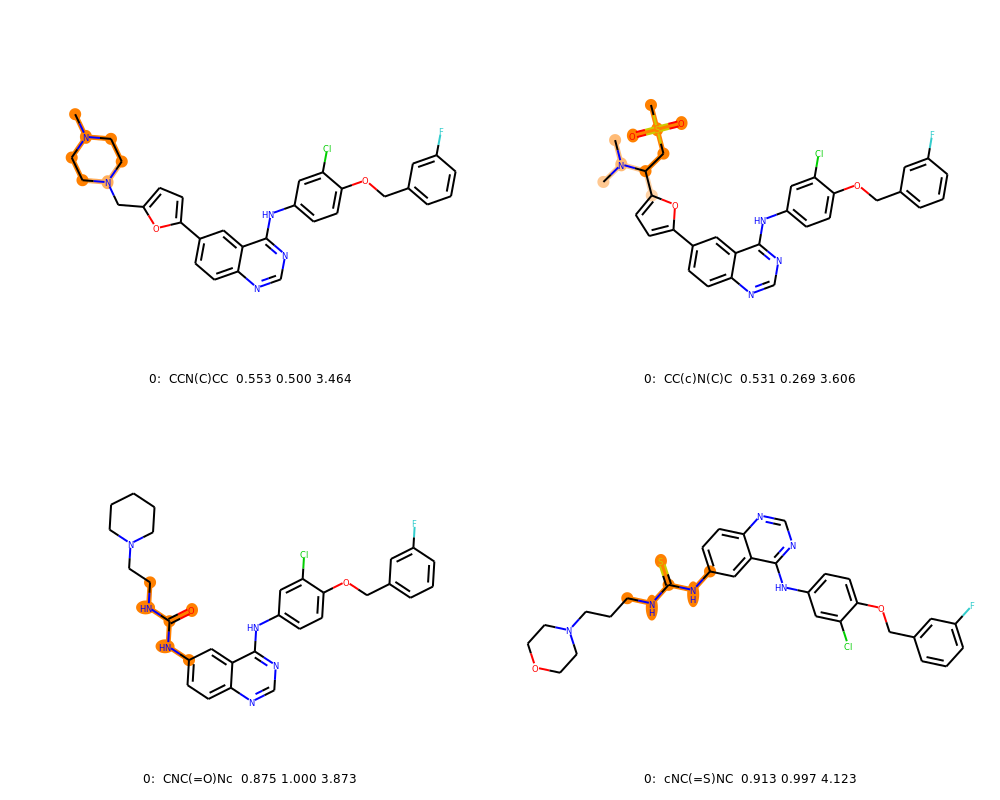

Supplement: Supplementary file 4 — Supplementary file4 (ZIP 111545 KB) [file 10822_2021_421_MOESM4_ESM.zip › 036/tp_cluster_0/tp0_mols_4.png]

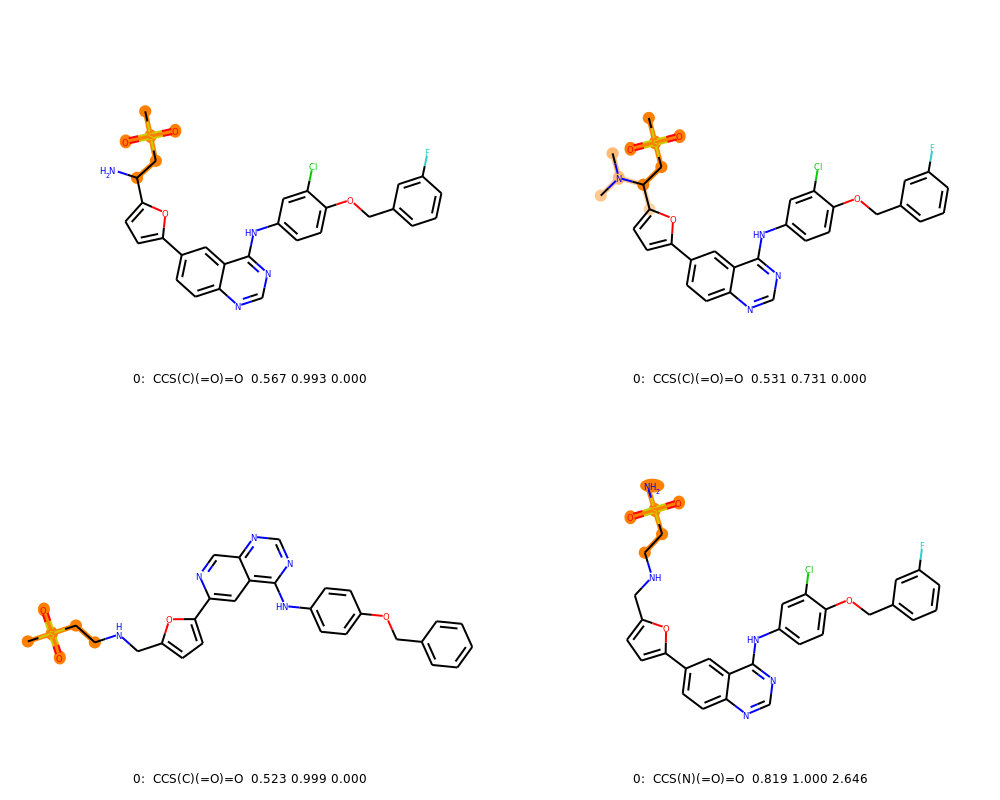

Supplement: Supplementary file 4 — Supplementary file4 (ZIP 111545 KB) [file 10822_2021_421_MOESM4_ESM.zip › 036/tp_cluster_0/tp0_mols_0.png]

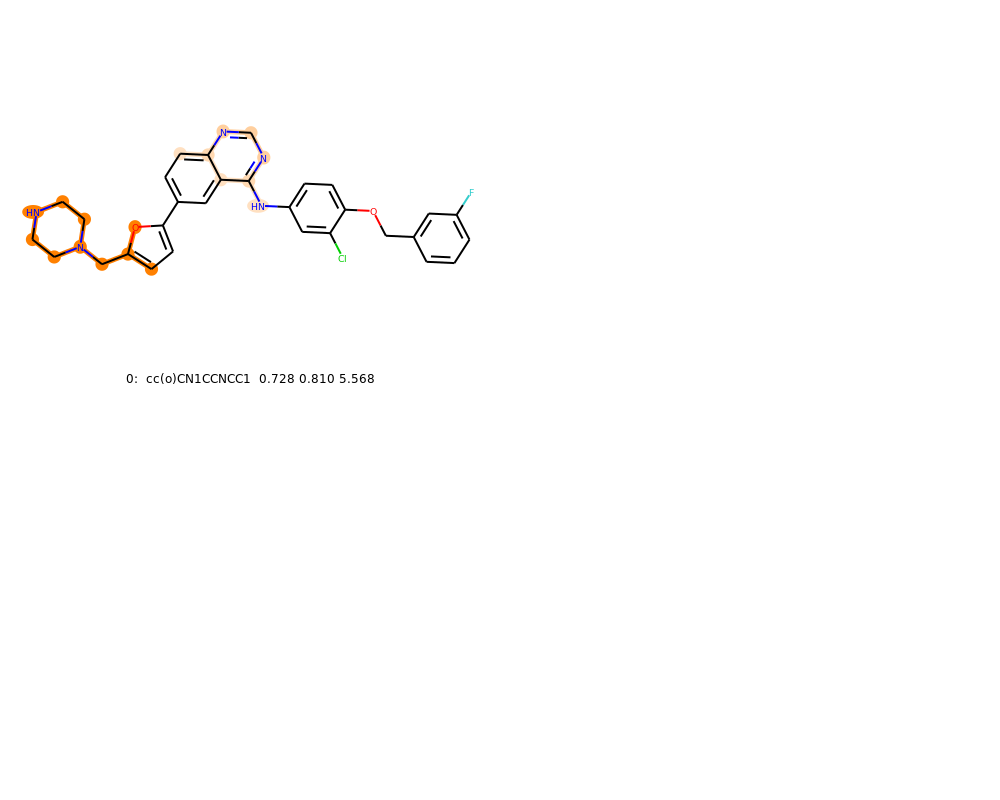

Supplement: Supplementary file 4 — Supplementary file4 (ZIP 111545 KB) [file 10822_2021_421_MOESM4_ESM.zip › 036/tp_cluster_0/tp0_mols_16.png]

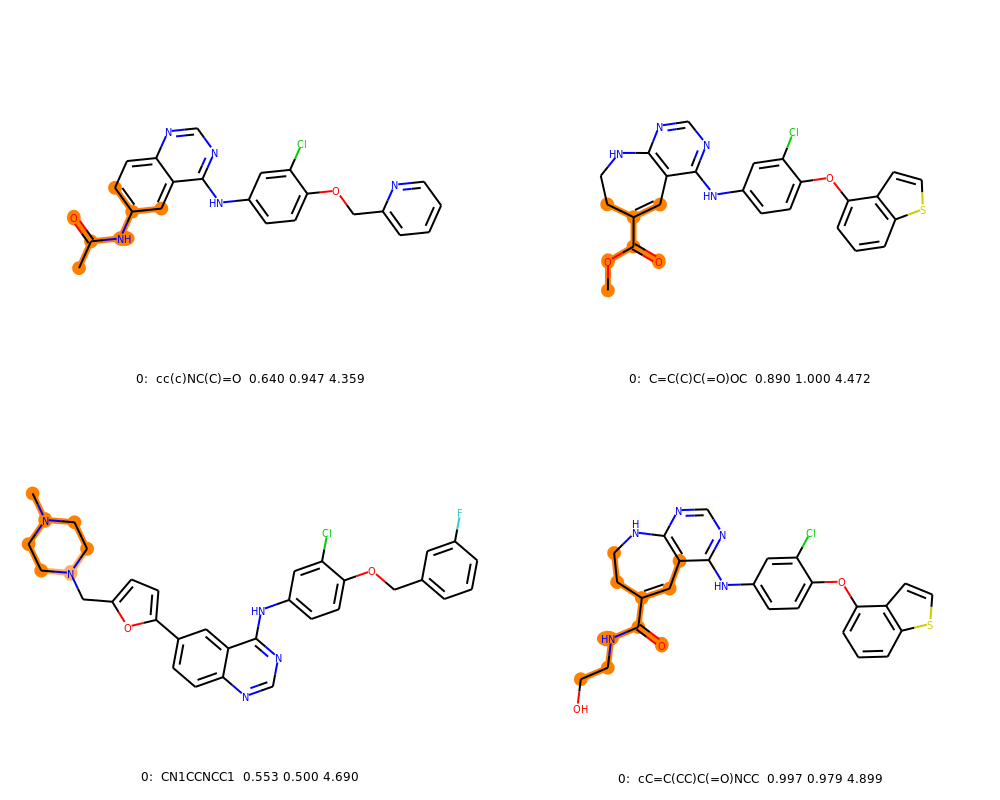

Supplement: Supplementary file 4 — Supplementary file4 (ZIP 111545 KB) [file 10822_2021_421_MOESM4_ESM.zip › 036/tp_cluster_0/tp0_mols_8.png]

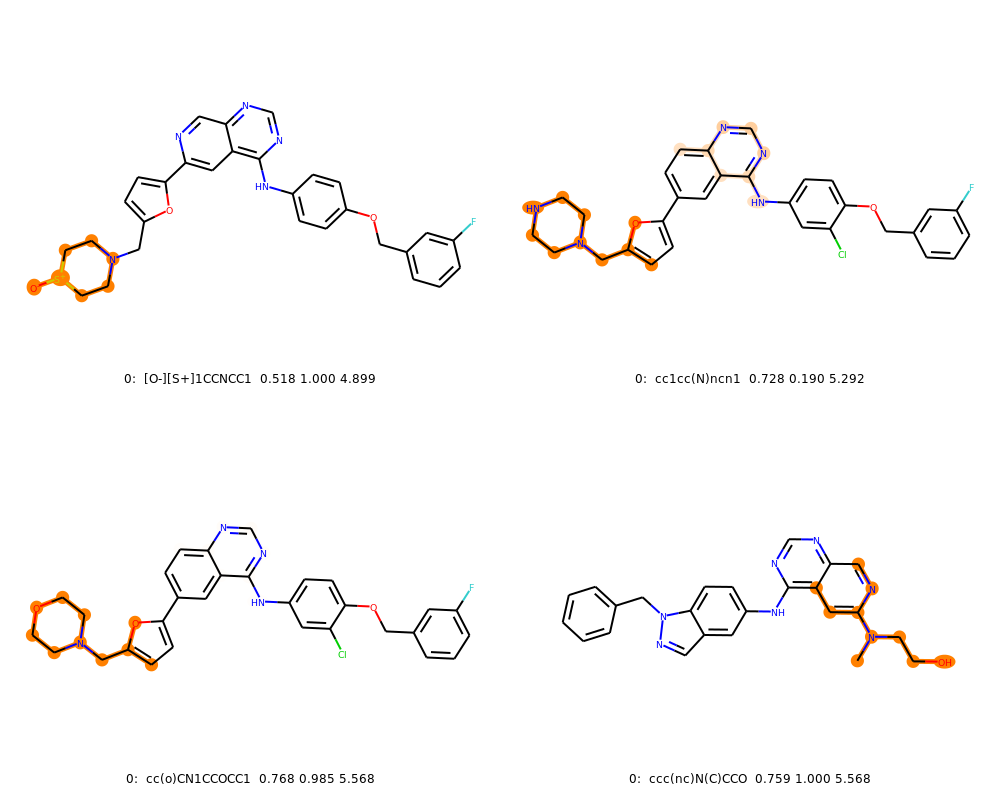

Supplement: Supplementary file 4 — Supplementary file4 (ZIP 111545 KB) [file 10822_2021_421_MOESM4_ESM.zip › 036/tp_cluster_0/tp0_mols_12.png]

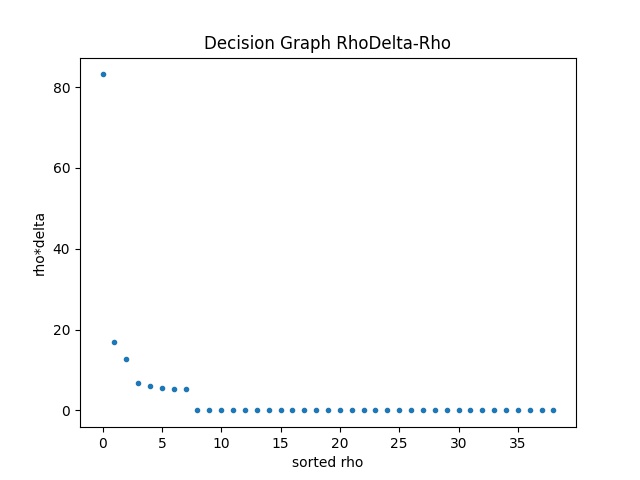

Supplement: Supplementary file 4 — Supplementary file4 (ZIP 111545 KB) [file 10822_2021_421_MOESM4_ESM.zip › 037/tp_decision_graphs/RhoDelta-Rho.jpg]

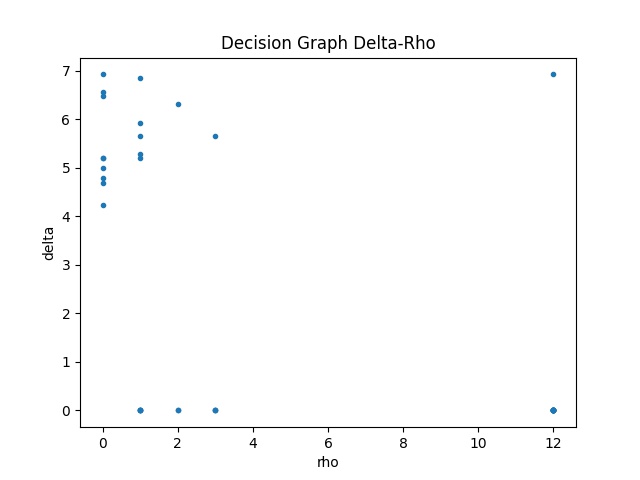

Supplement: Supplementary file 4 — Supplementary file4 (ZIP 111545 KB) [file 10822_2021_421_MOESM4_ESM.zip › 037/tp_decision_graphs/Delta-Rho.jpg]

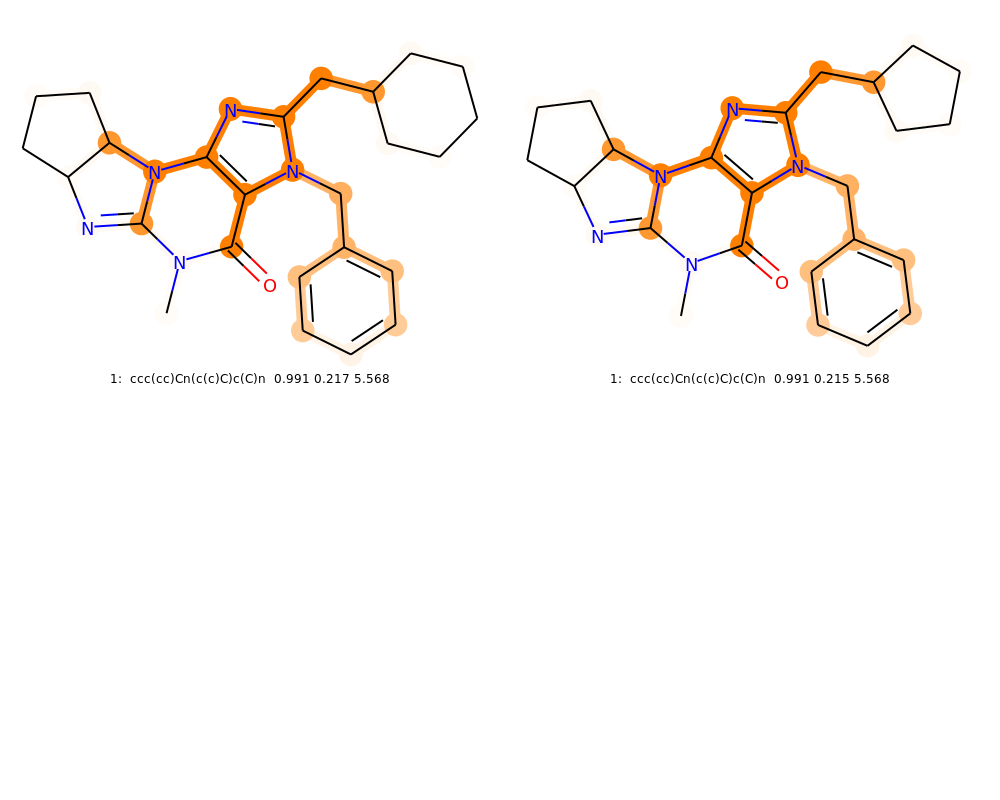

Supplement: Supplementary file 4 — Supplementary file4 (ZIP 111545 KB) [file 10822_2021_421_MOESM4_ESM.zip › 037/tp_cluster_1/tp1_mols_8.png]

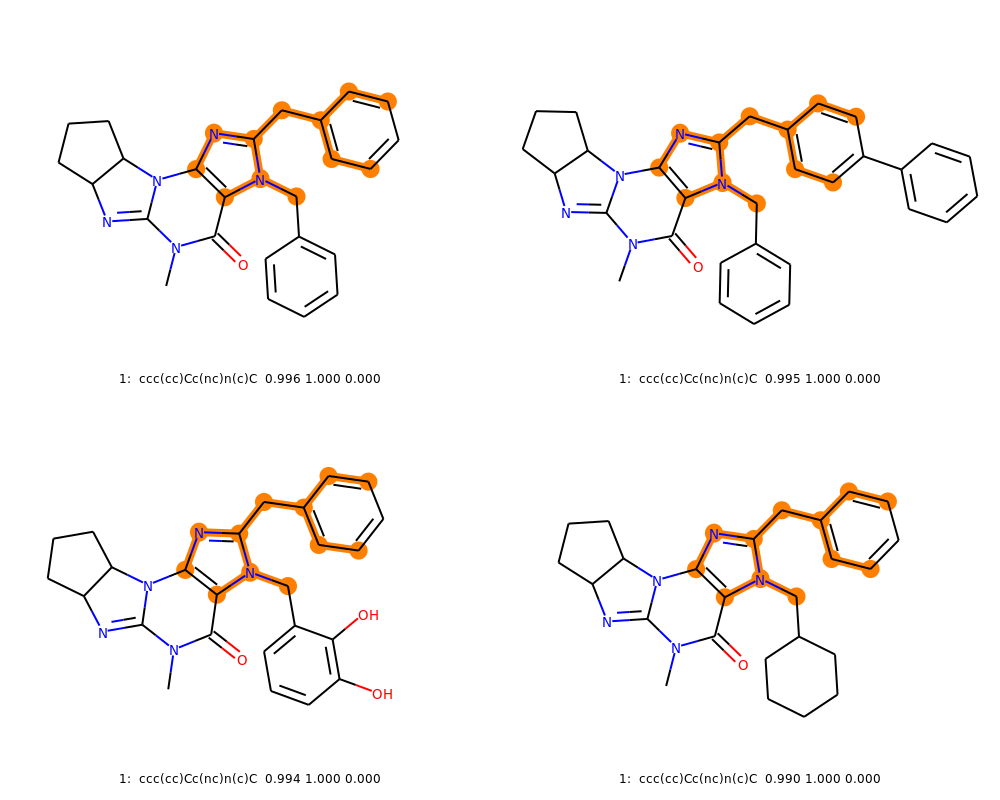

Supplement: Supplementary file 4 — Supplementary file4 (ZIP 111545 KB) [file 10822_2021_421_MOESM4_ESM.zip › 037/tp_cluster_1/tp1_mols_0.png]

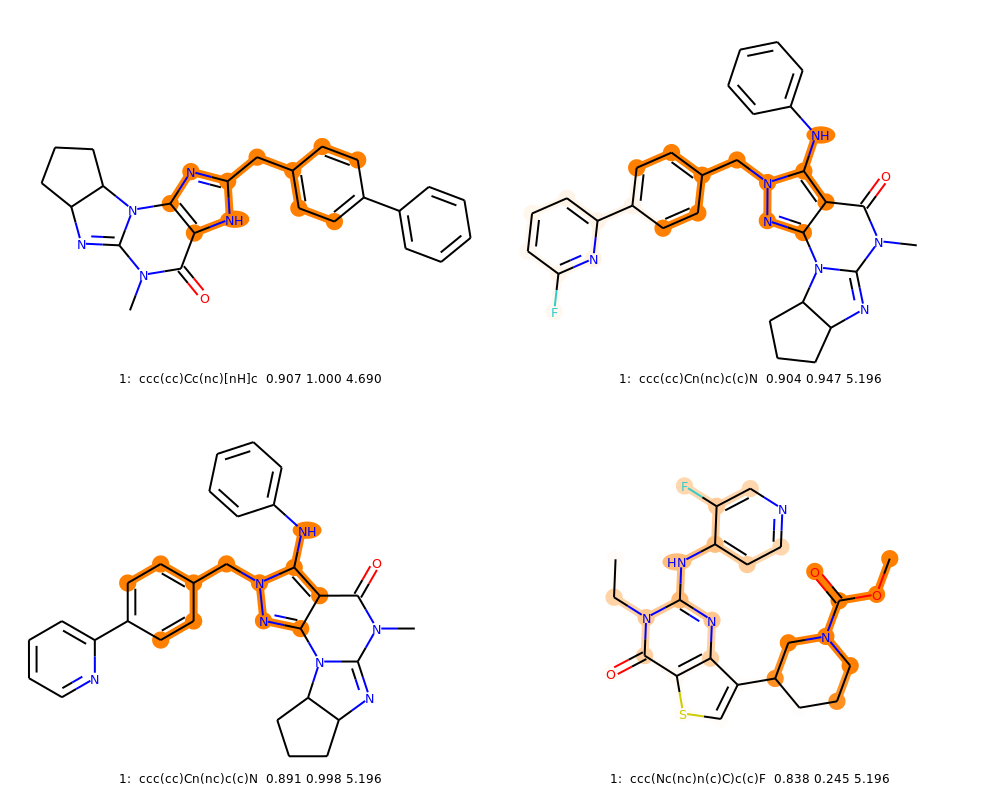

Supplement: Supplementary file 4 — Supplementary file4 (ZIP 111545 KB) [file 10822_2021_421_MOESM4_ESM.zip › 037/tp_cluster_1/tp1_mols_4.png]

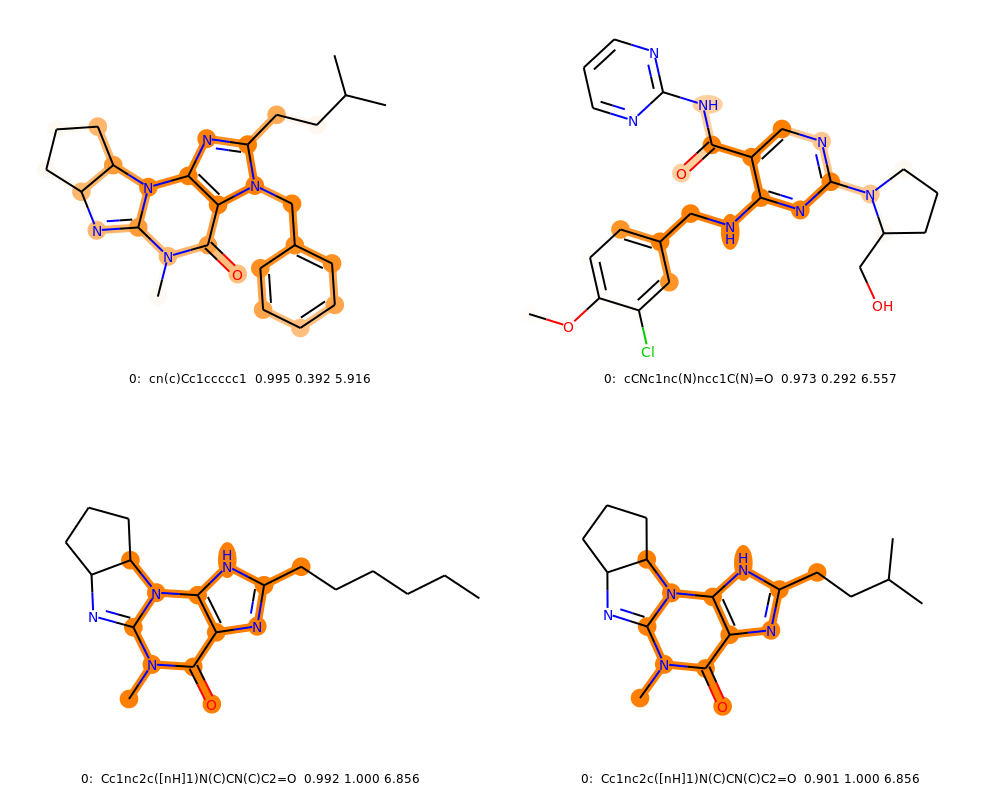

Supplement: Supplementary file 4 — Supplementary file4 (ZIP 111545 KB) [file 10822_2021_421_MOESM4_ESM.zip › 037/tp_cluster_0/tp0_mols_20.png]

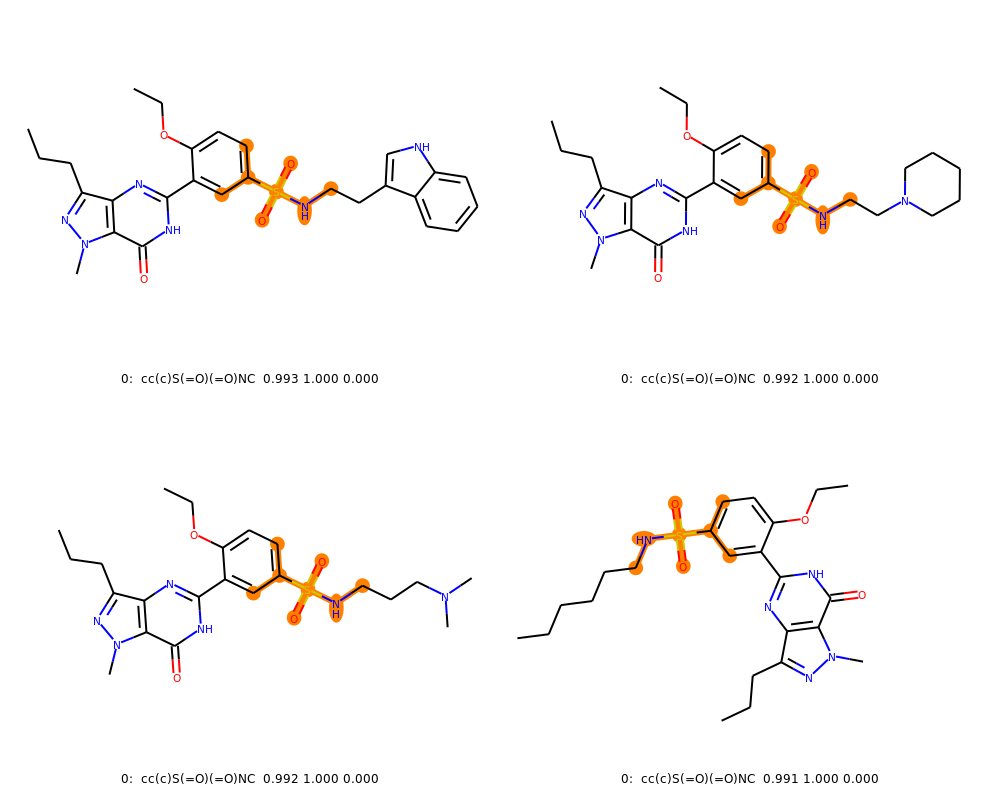

Supplement: Supplementary file 4 — Supplementary file4 (ZIP 111545 KB) [file 10822_2021_421_MOESM4_ESM.zip › 037/tp_cluster_0/tp0_mols_4.png]

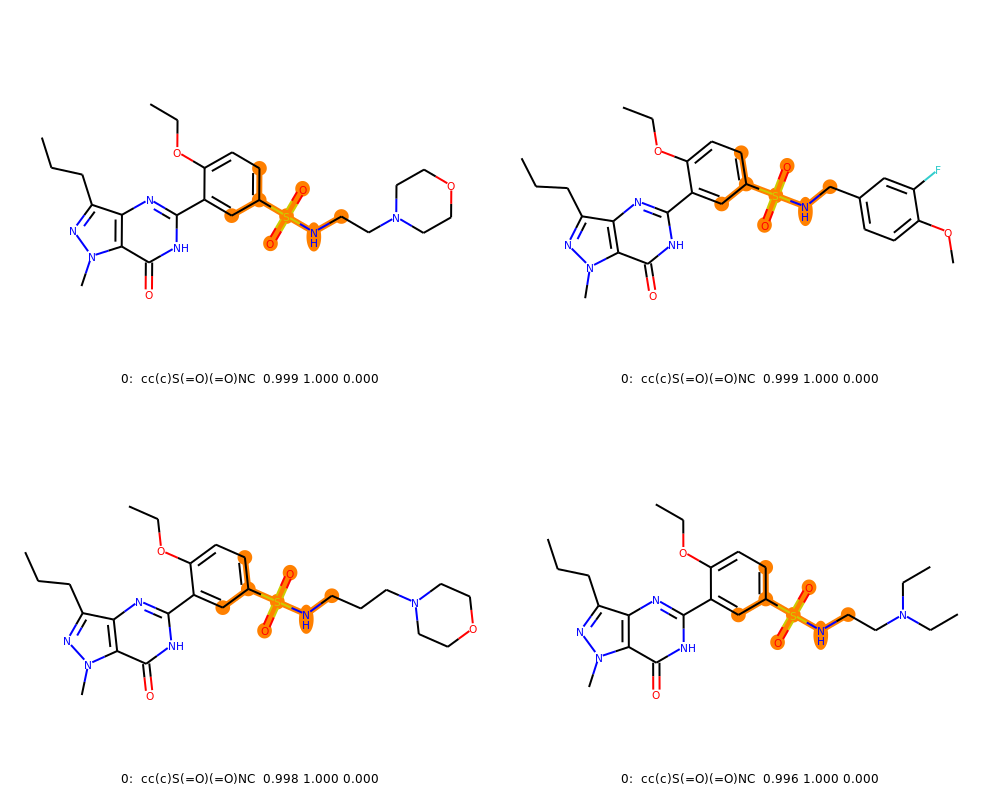

Supplement: Supplementary file 4 — Supplementary file4 (ZIP 111545 KB) [file 10822_2021_421_MOESM4_ESM.zip › 037/tp_cluster_0/tp0_mols_0.png]

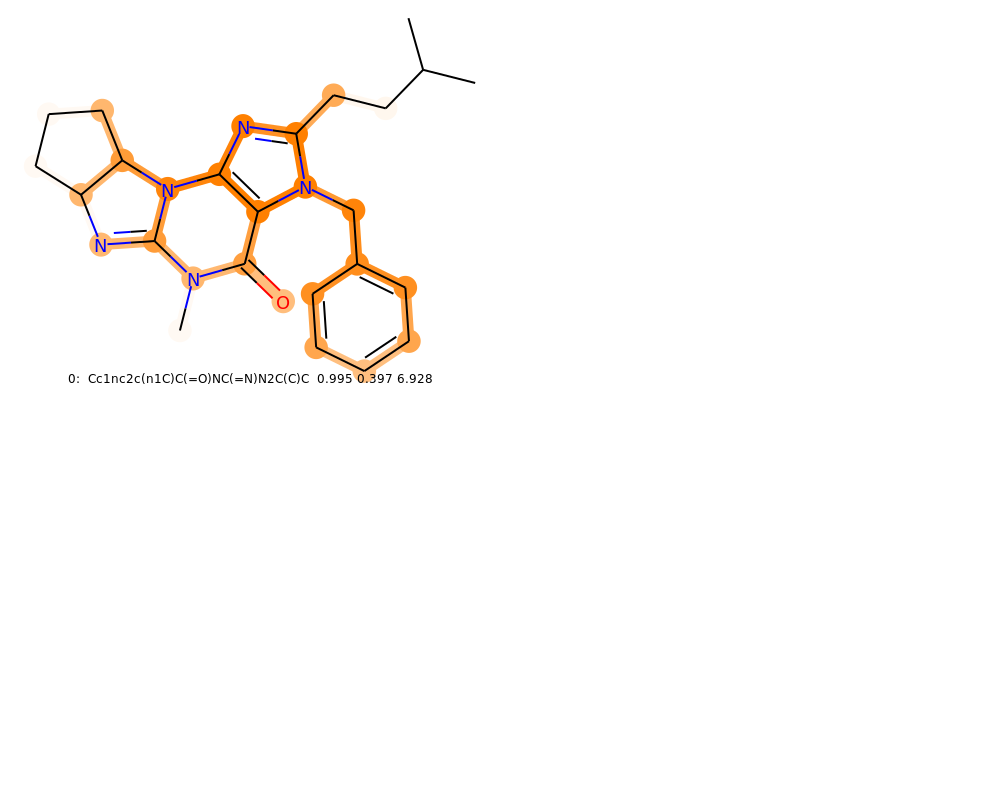

Supplement: Supplementary file 4 — Supplementary file4 (ZIP 111545 KB) [file 10822_2021_421_MOESM4_ESM.zip › 037/tp_cluster_0/tp0_mols_24.png]

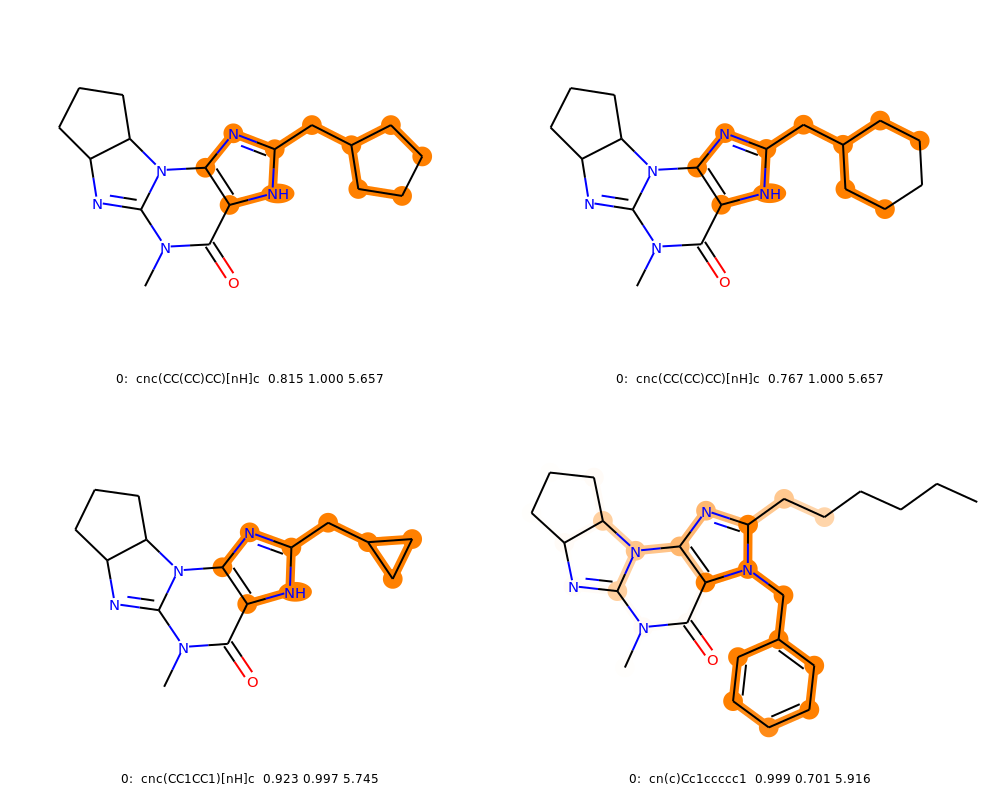

Supplement: Supplementary file 4 — Supplementary file4 (ZIP 111545 KB) [file 10822_2021_421_MOESM4_ESM.zip › 037/tp_cluster_0/tp0_mols_16.png]

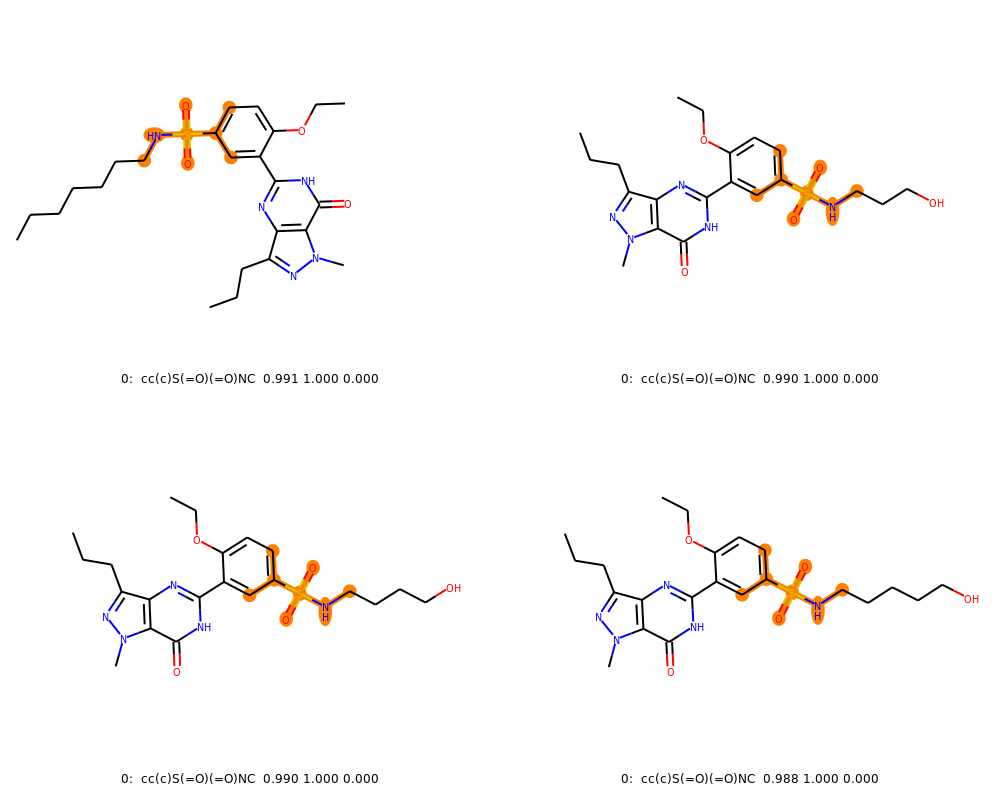

Supplement: Supplementary file 4 — Supplementary file4 (ZIP 111545 KB) [file 10822_2021_421_MOESM4_ESM.zip › 037/tp_cluster_0/tp0_mols_8.png]

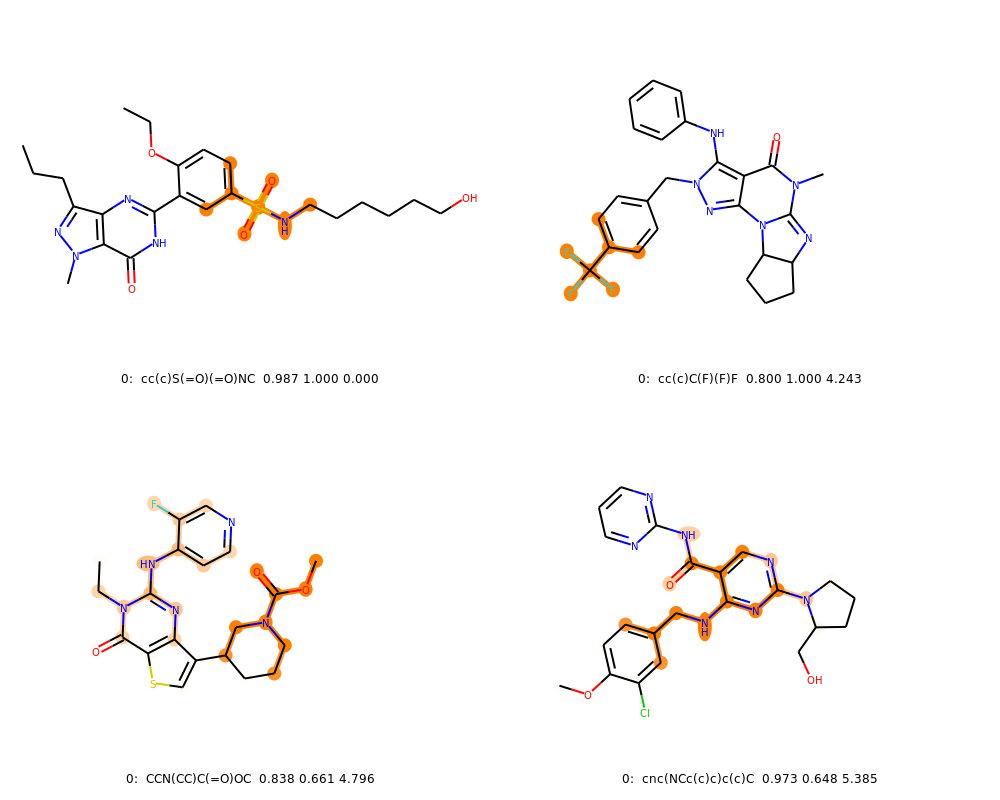

Supplement: Supplementary file 4 — Supplementary file4 (ZIP 111545 KB) [file 10822_2021_421_MOESM4_ESM.zip › 037/tp_cluster_0/tp0_mols_12.png]

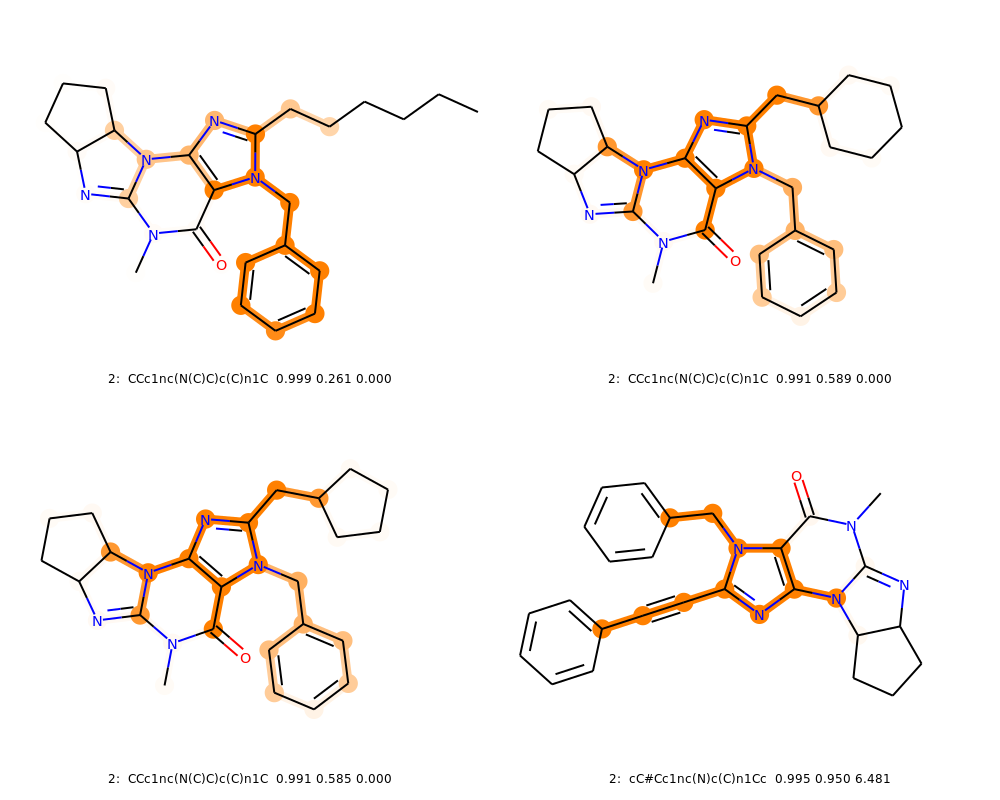

Supplement: Supplementary file 4 — Supplementary file4 (ZIP 111545 KB) [file 10822_2021_421_MOESM4_ESM.zip › 037/tp_cluster_2/tp2_mols_0.png]

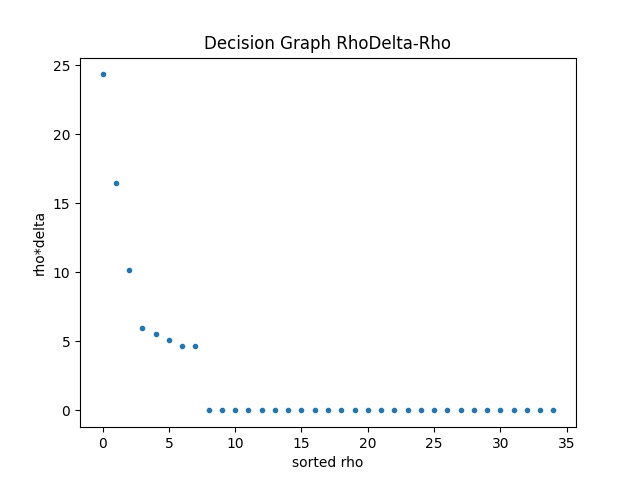

Supplement: Supplementary file 4 — Supplementary file4 (ZIP 111545 KB) [file 10822_2021_421_MOESM4_ESM.zip › 038/tp_decision_graphs/RhoDelta-Rho.jpg]

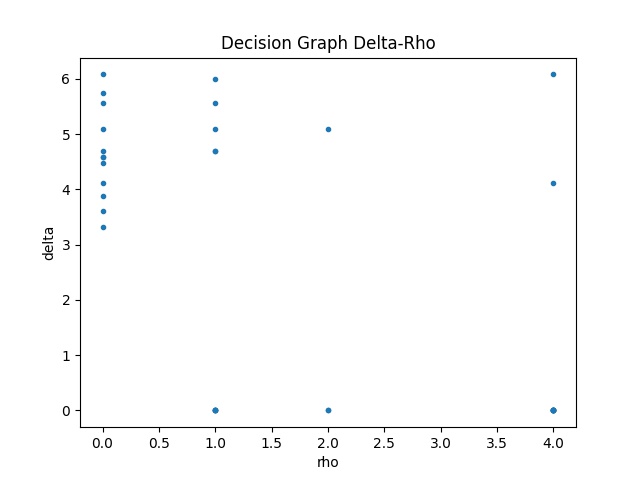

Supplement: Supplementary file 4 — Supplementary file4 (ZIP 111545 KB) [file 10822_2021_421_MOESM4_ESM.zip › 038/tp_decision_graphs/Delta-Rho.jpg]

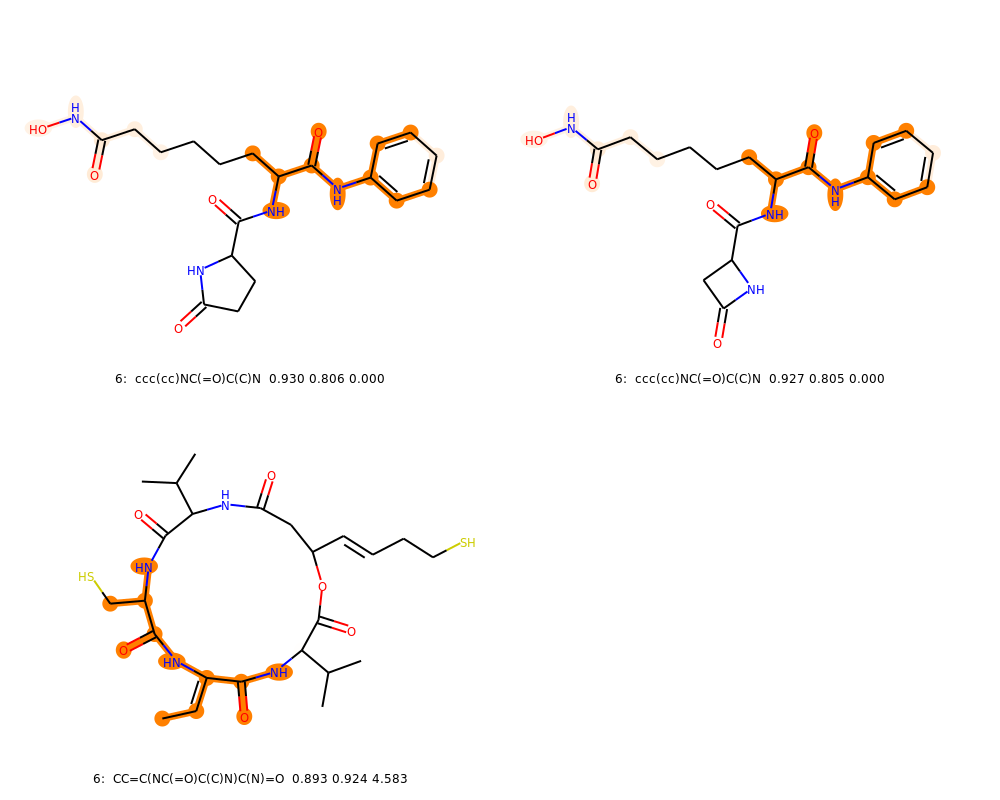

Supplement: Supplementary file 4 — Supplementary file4 (ZIP 111545 KB) [file 10822_2021_421_MOESM4_ESM.zip › 038/tp_cluster_6/tp6_mols_0.png]

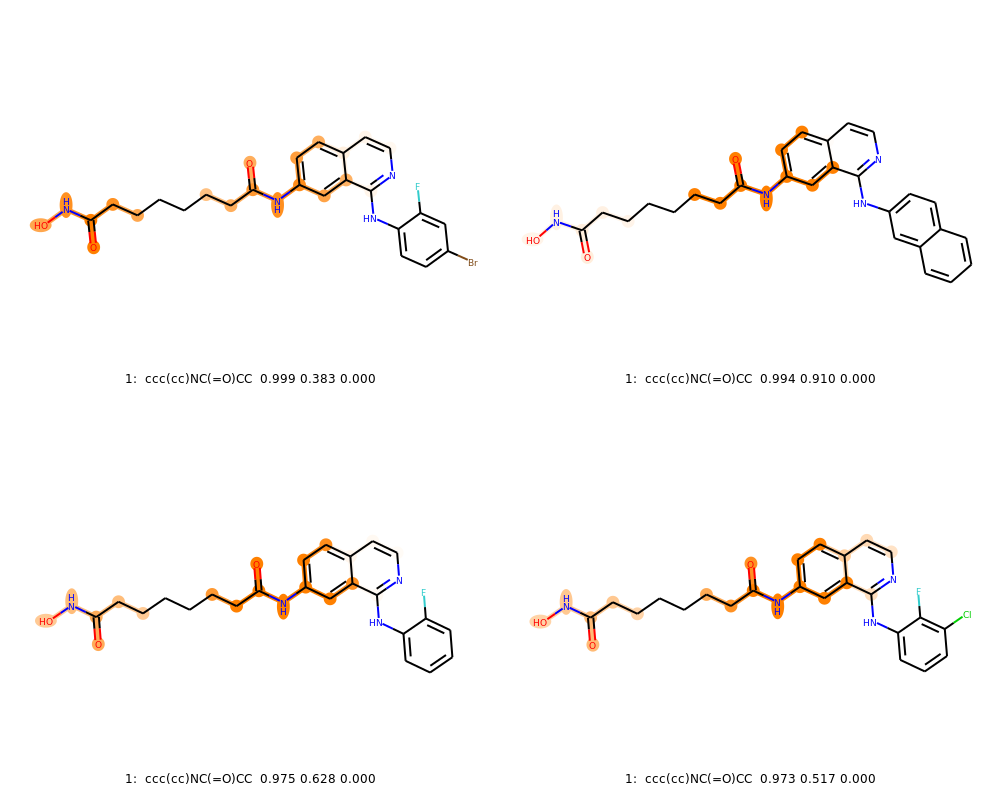

Supplement: Supplementary file 4 — Supplementary file4 (ZIP 111545 KB) [file 10822_2021_421_MOESM4_ESM.zip › 038/tp_cluster_1/tp1_mols_0.png]

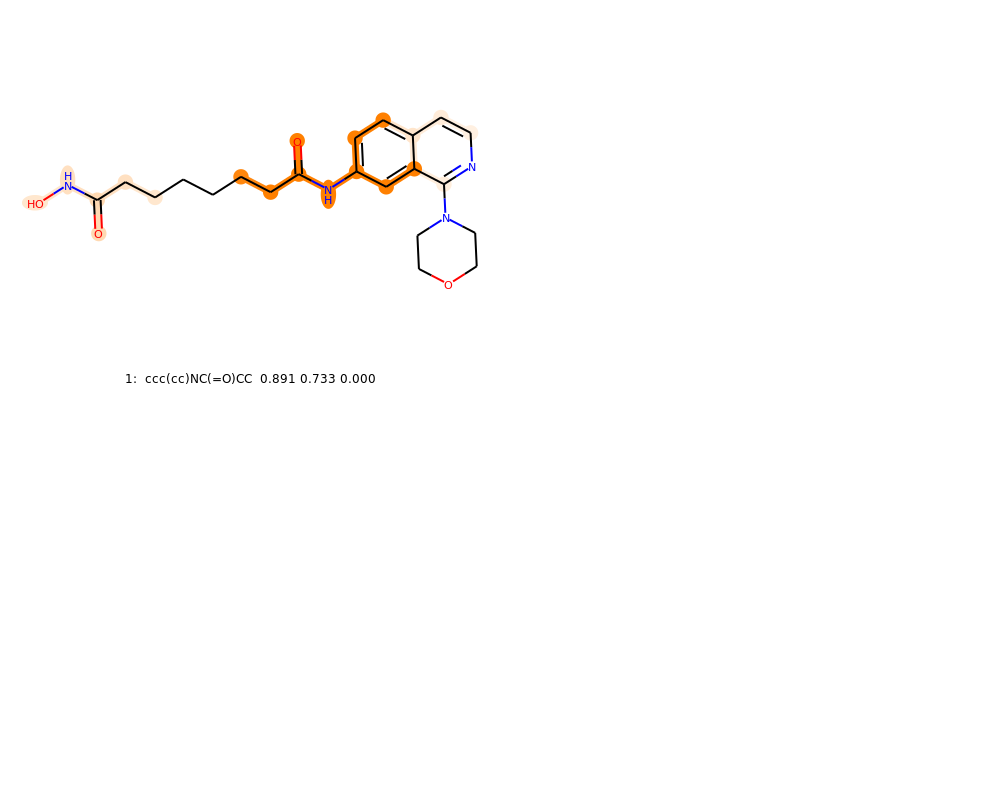

Supplement: Supplementary file 4 — Supplementary file4 (ZIP 111545 KB) [file 10822_2021_421_MOESM4_ESM.zip › 038/tp_cluster_1/tp1_mols_4.png]

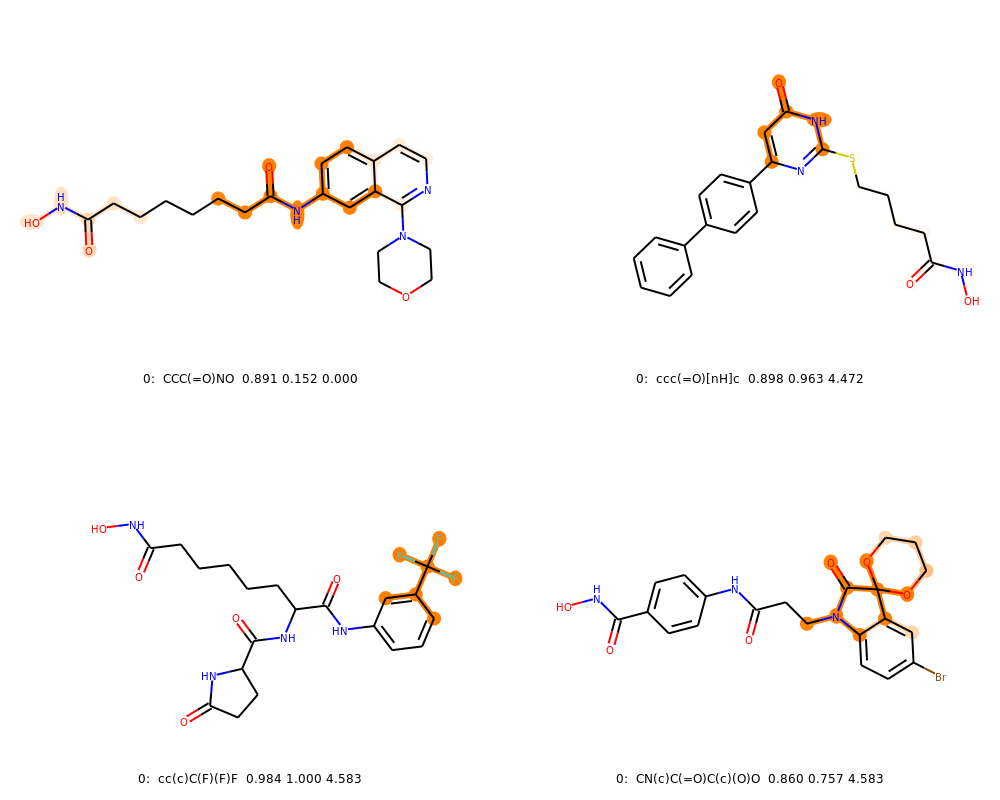

Supplement: Supplementary file 4 — Supplementary file4 (ZIP 111545 KB) [file 10822_2021_421_MOESM4_ESM.zip › 038/tp_cluster_0/tp0_mols_4.png]

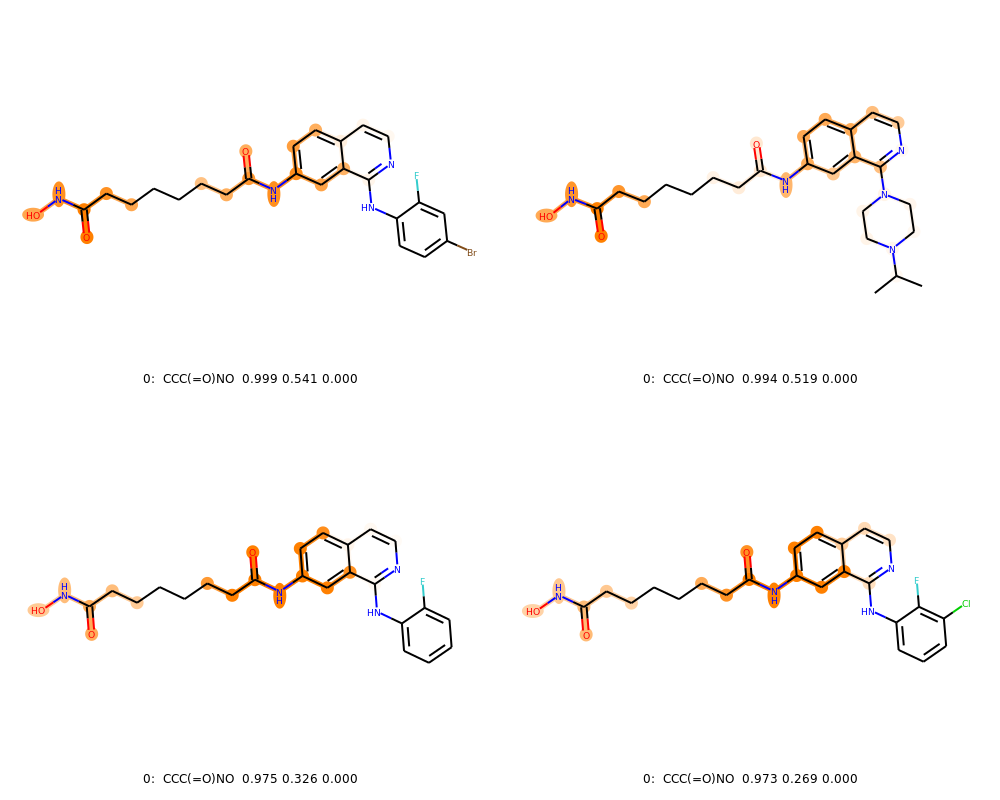

Supplement: Supplementary file 4 — Supplementary file4 (ZIP 111545 KB) [file 10822_2021_421_MOESM4_ESM.zip › 038/tp_cluster_0/tp0_mols_0.png]

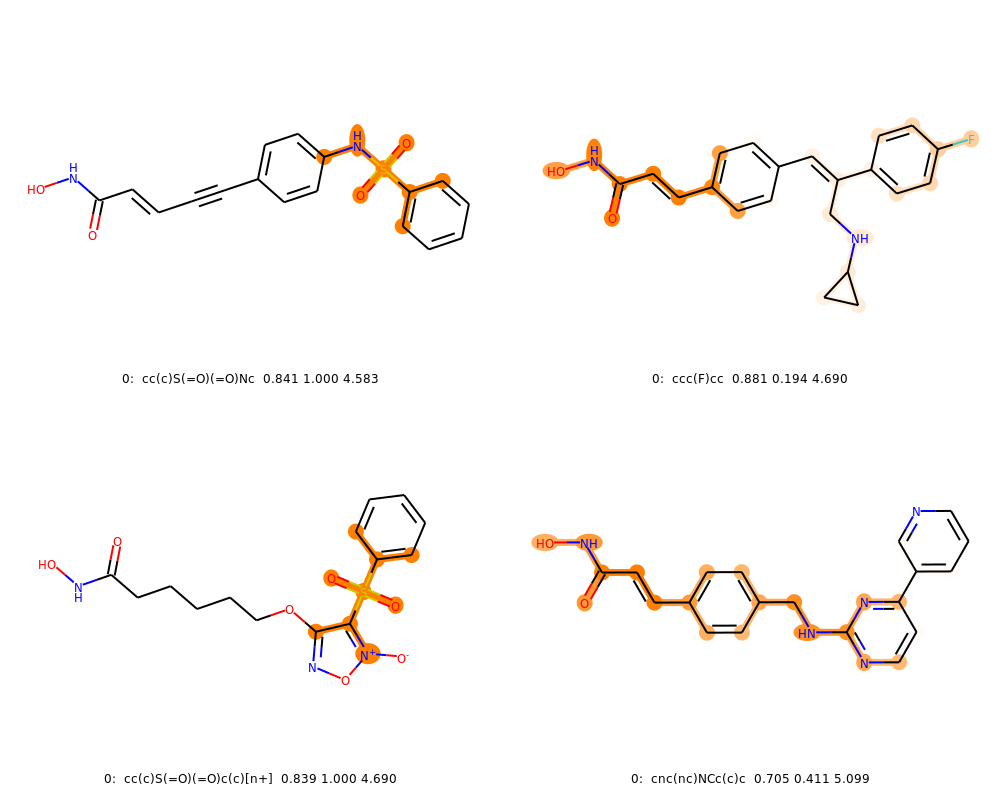

Supplement: Supplementary file 4 — Supplementary file4 (ZIP 111545 KB) [file 10822_2021_421_MOESM4_ESM.zip › 038/tp_cluster_0/tp0_mols_8.png]

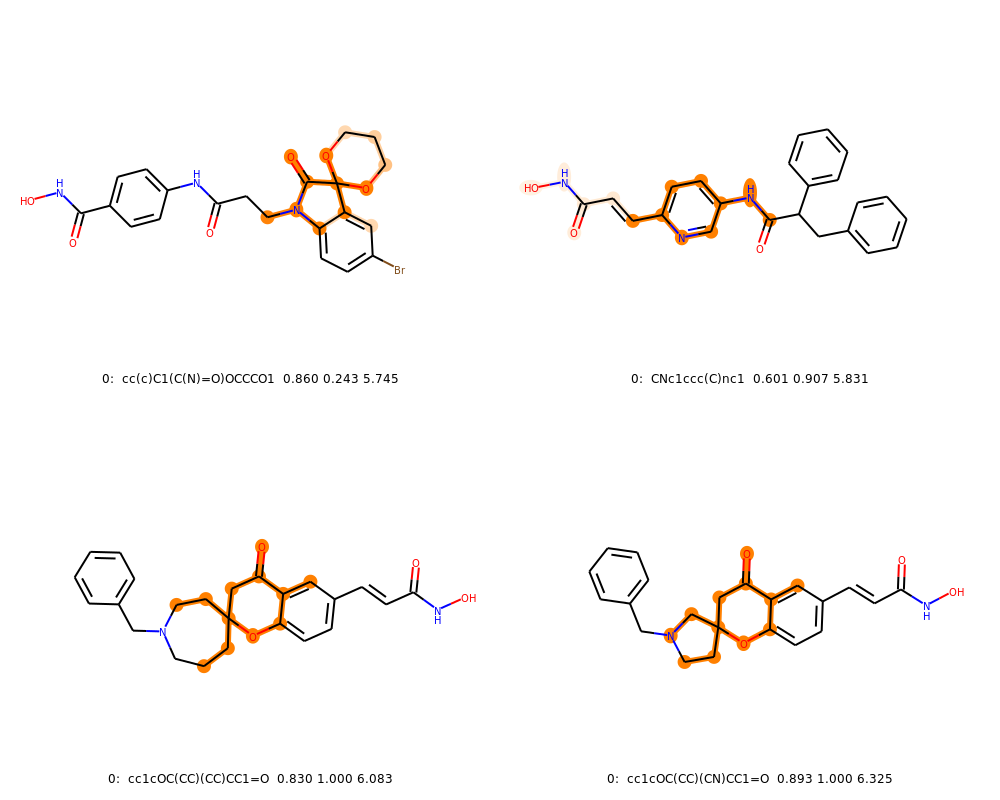

Supplement: Supplementary file 4 — Supplementary file4 (ZIP 111545 KB) [file 10822_2021_421_MOESM4_ESM.zip › 038/tp_cluster_0/tp0_mols_12.png]

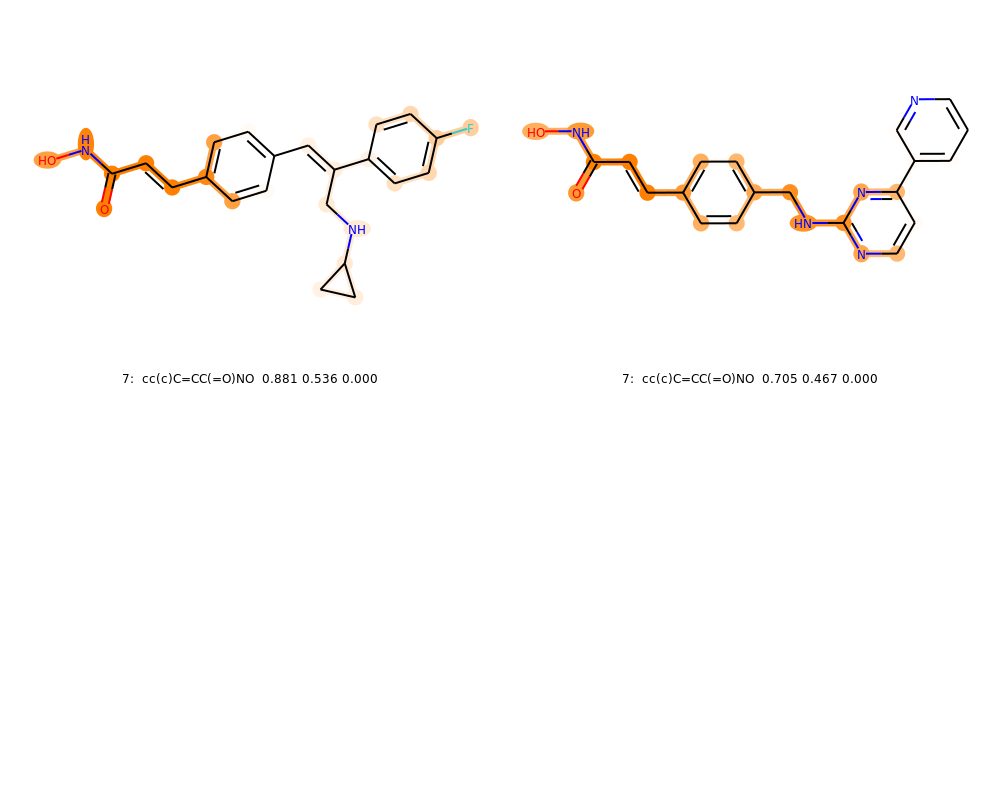

Supplement: Supplementary file 4 — Supplementary file4 (ZIP 111545 KB) [file 10822_2021_421_MOESM4_ESM.zip › 038/tp_cluster_7/tp7_mols_0.png]

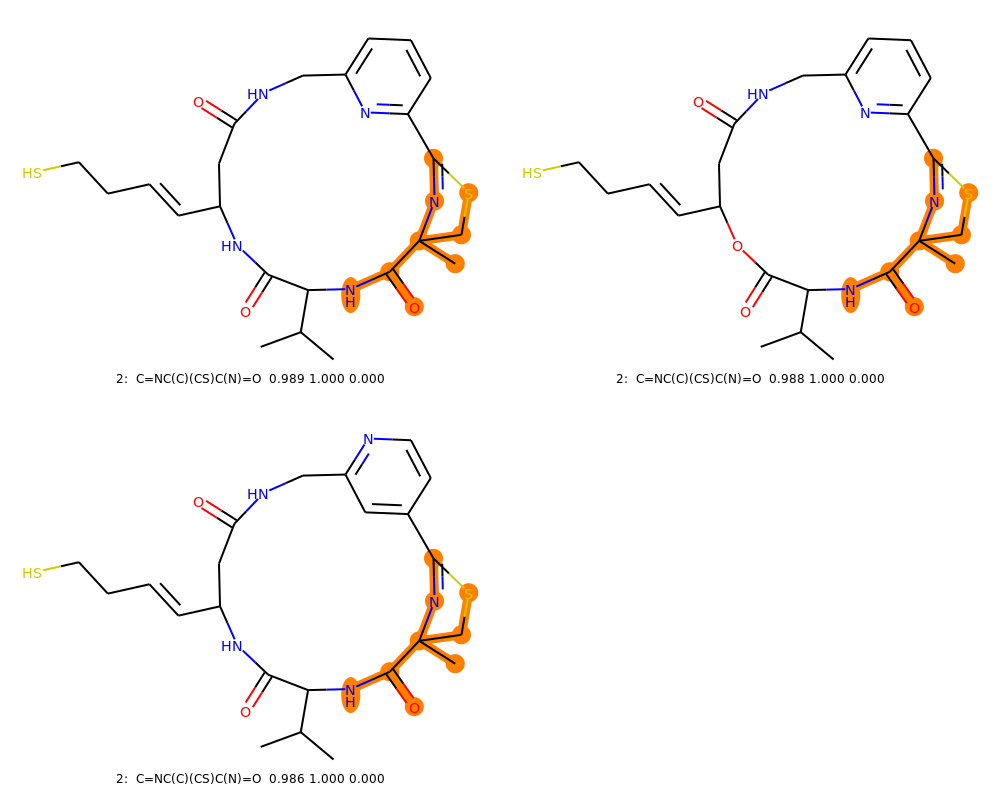

Supplement: Supplementary file 4 — Supplementary file4 (ZIP 111545 KB) [file 10822_2021_421_MOESM4_ESM.zip › 038/tp_cluster_2/tp2_mols_0.png]

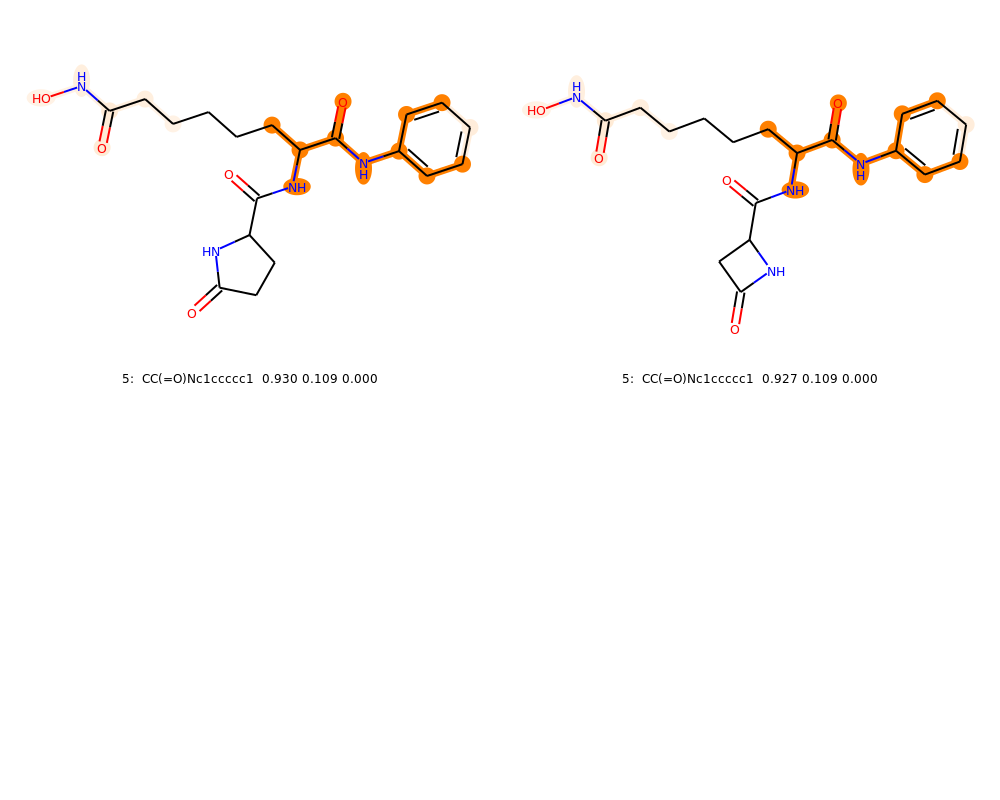

Supplement: Supplementary file 4 — Supplementary file4 (ZIP 111545 KB) [file 10822_2021_421_MOESM4_ESM.zip › 038/tp_cluster_5/tp5_mols_0.png]

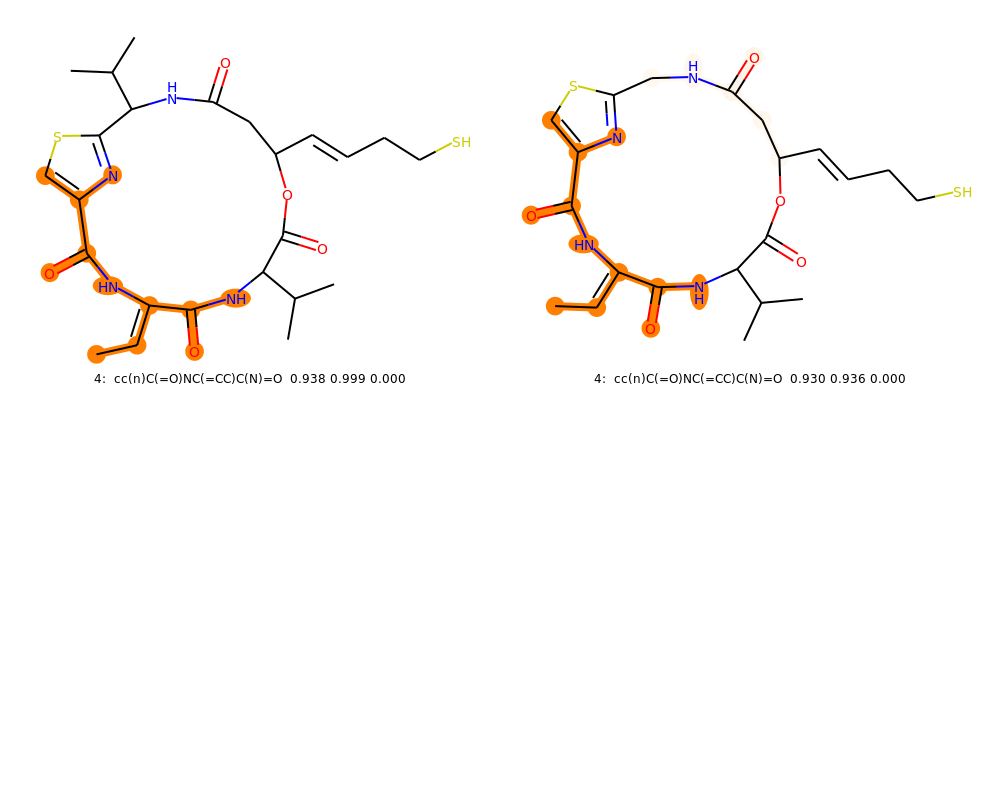

Supplement: Supplementary file 4 — Supplementary file4 (ZIP 111545 KB) [file 10822_2021_421_MOESM4_ESM.zip › 038/tp_cluster_4/tp4_mols_0.png]

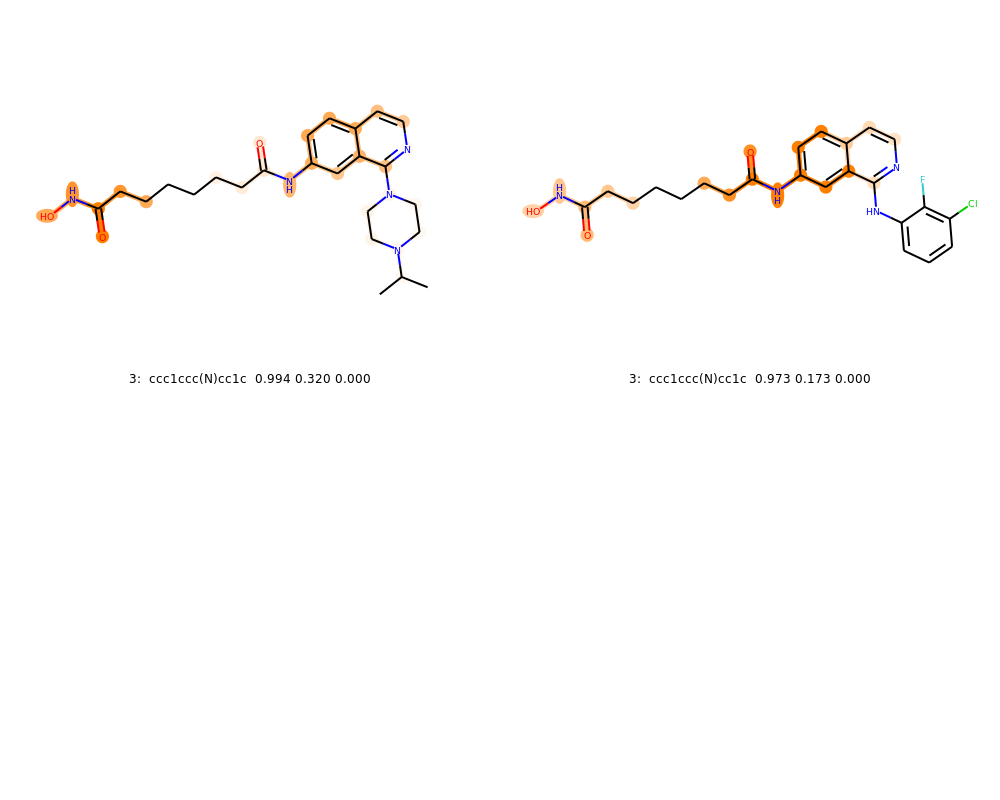

Supplement: Supplementary file 4 — Supplementary file4 (ZIP 111545 KB) [file 10822_2021_421_MOESM4_ESM.zip › 038/tp_cluster_3/tp3_mols_0.png]

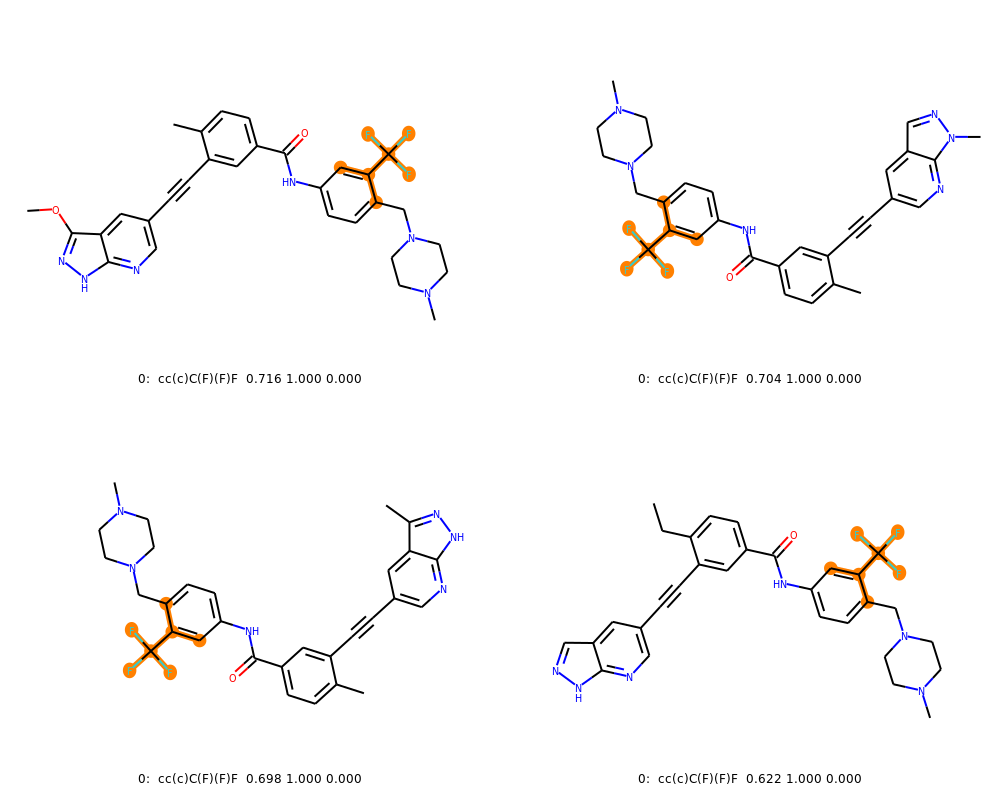

Supplement: Supplementary file 4 — Supplementary file4 (ZIP 111545 KB) [file 10822_2021_421_MOESM4_ESM.zip › 048/tp_cluster_0/tp0_mols_4.png]

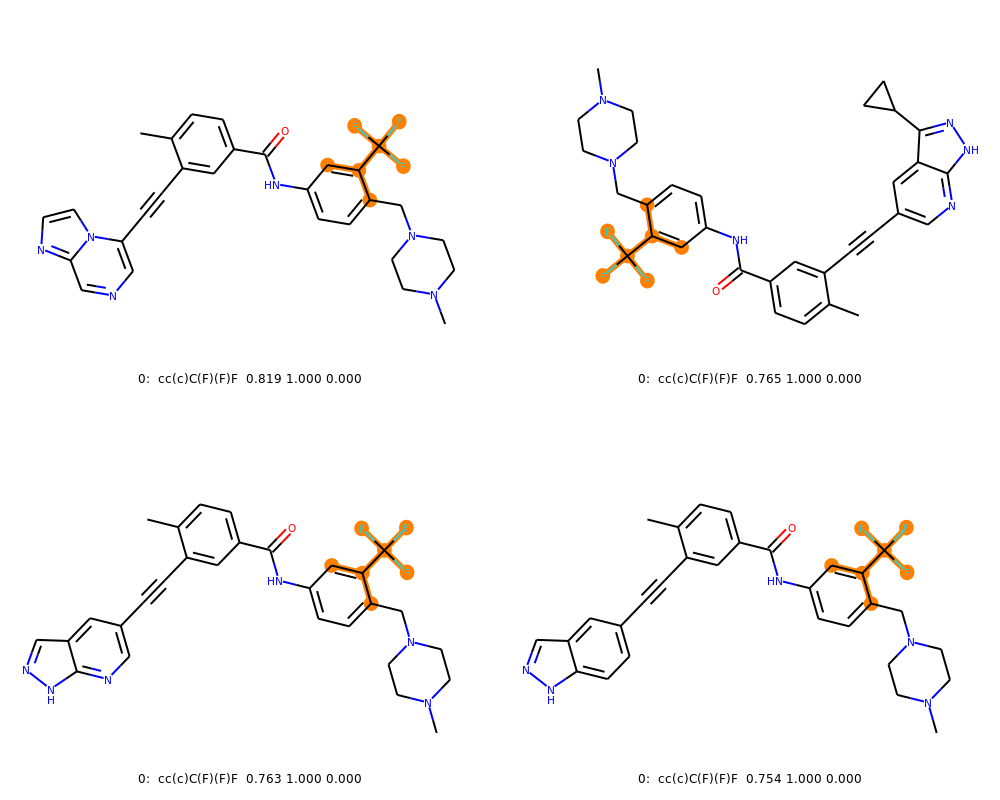

Supplement: Supplementary file 4 — Supplementary file4 (ZIP 111545 KB) [file 10822_2021_421_MOESM4_ESM.zip › 048/tp_cluster_0/tp0_mols_0.png]

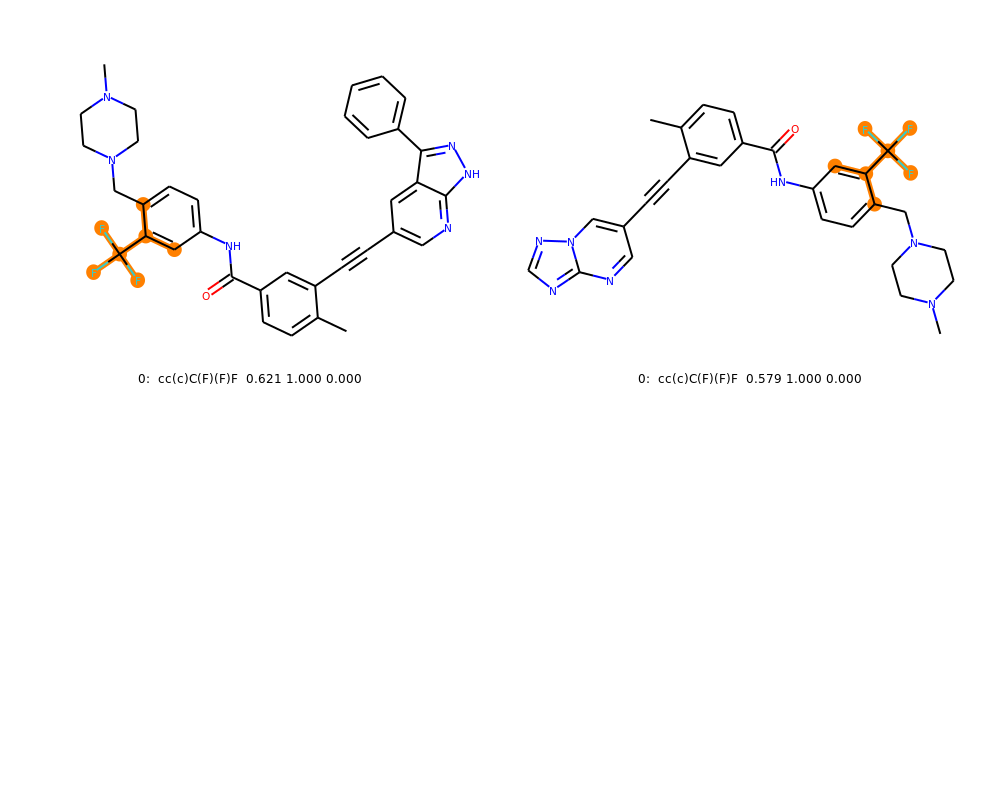

Supplement: Supplementary file 4 — Supplementary file4 (ZIP 111545 KB) [file 10822_2021_421_MOESM4_ESM.zip › 048/tp_cluster_0/tp0_mols_8.png]

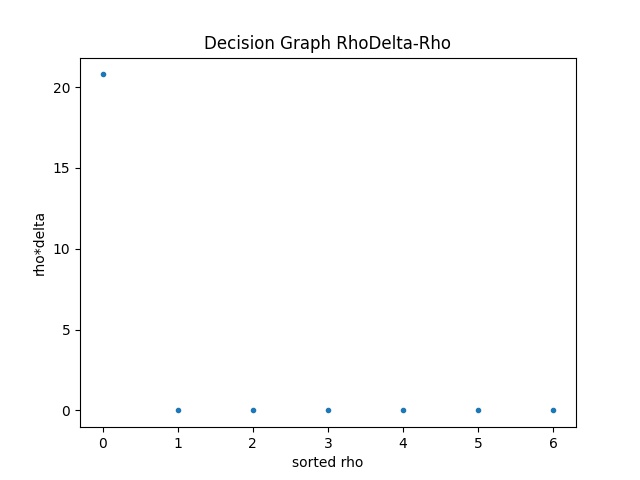

Supplement: Supplementary file 4 — Supplementary file4 (ZIP 111545 KB) [file 10822_2021_421_MOESM4_ESM.zip › 051/tp_decision_graphs/RhoDelta-Rho.jpg]

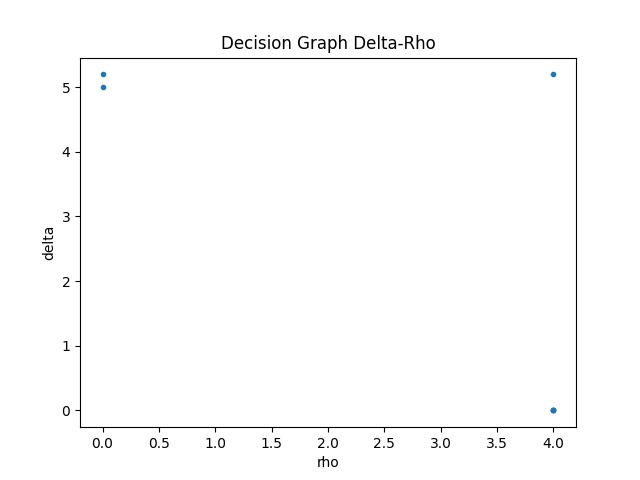

Supplement: Supplementary file 4 — Supplementary file4 (ZIP 111545 KB) [file 10822_2021_421_MOESM4_ESM.zip › 051/tp_decision_graphs/Delta-Rho.jpg]

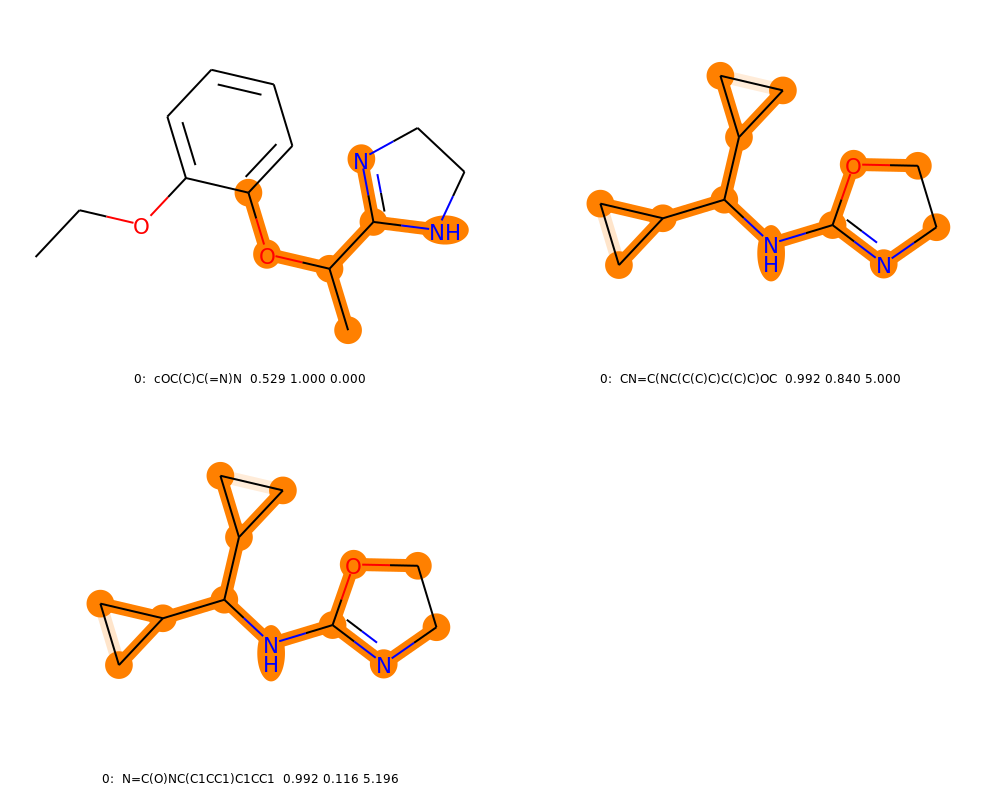

Supplement: Supplementary file 4 — Supplementary file4 (ZIP 111545 KB) [file 10822_2021_421_MOESM4_ESM.zip › 051/tp_cluster_0/tp0_mols_4.png]

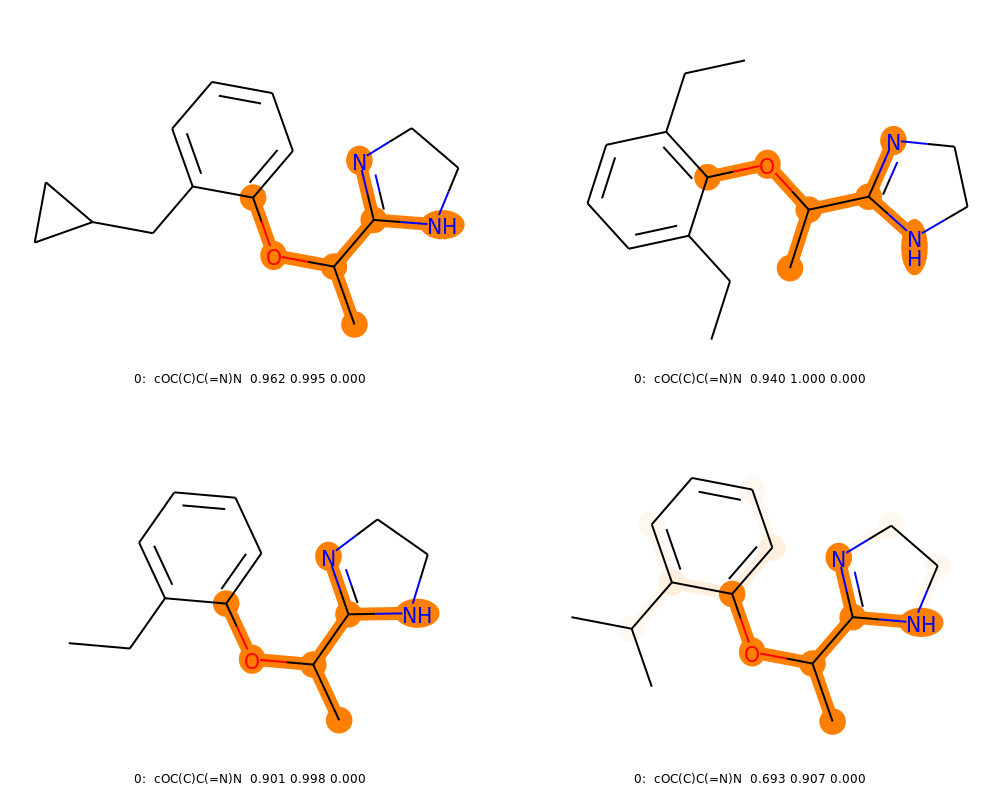

Supplement: Supplementary file 4 — Supplementary file4 (ZIP 111545 KB) [file 10822_2021_421_MOESM4_ESM.zip › 051/tp_cluster_0/tp0_mols_0.png]

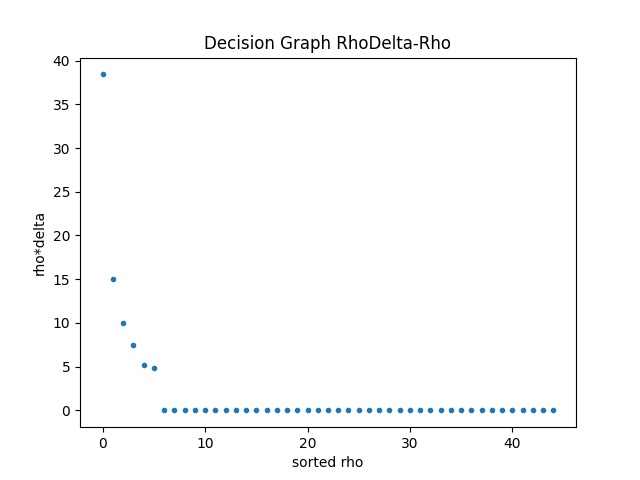

Supplement: Supplementary file 4 — Supplementary file4 (ZIP 111545 KB) [file 10822_2021_421_MOESM4_ESM.zip › 053/tp_decision_graphs/RhoDelta-Rho.jpg]

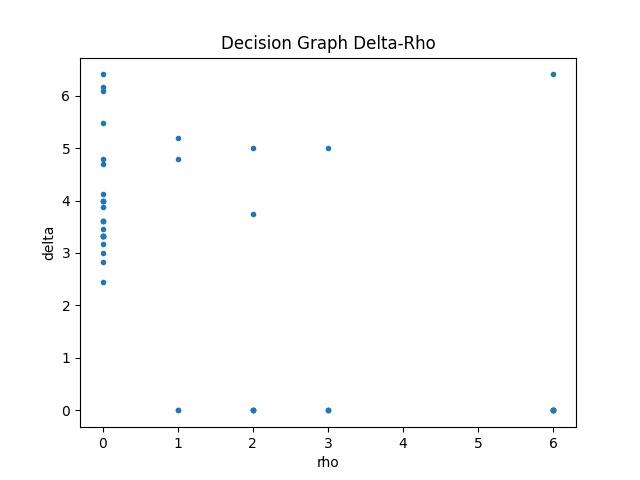

Supplement: Supplementary file 4 — Supplementary file4 (ZIP 111545 KB) [file 10822_2021_421_MOESM4_ESM.zip › 053/tp_decision_graphs/Delta-Rho.jpg]

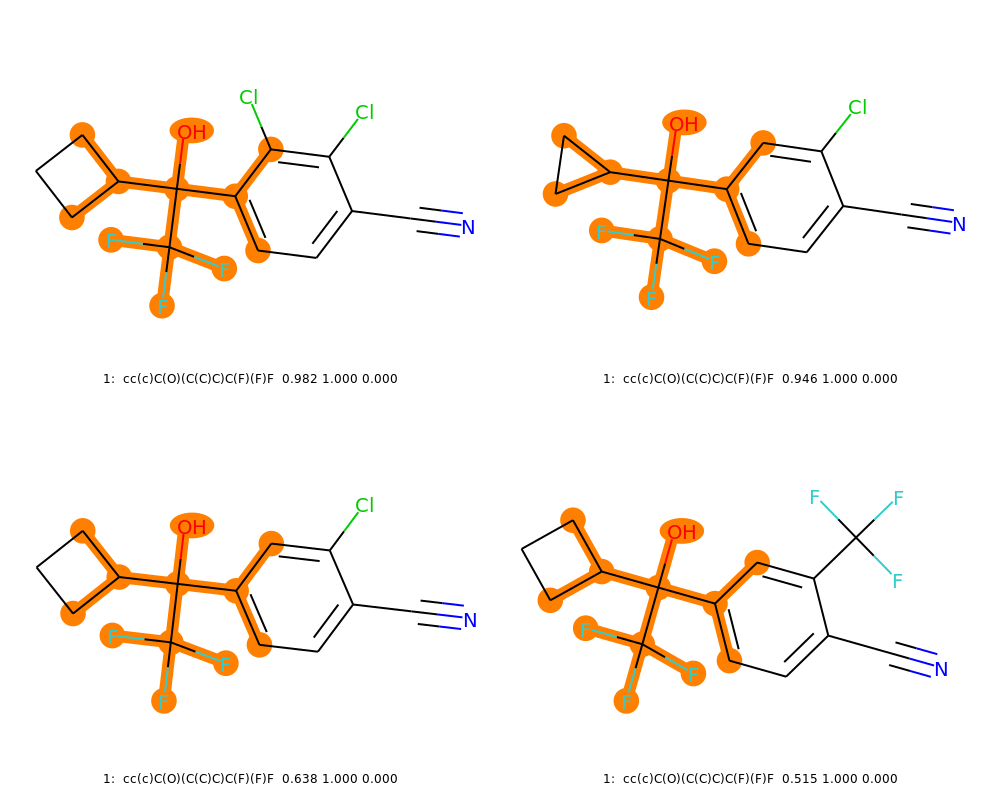

Supplement: Supplementary file 4 — Supplementary file4 (ZIP 111545 KB) [file 10822_2021_421_MOESM4_ESM.zip › 053/tp_cluster_1/tp1_mols_0.png]

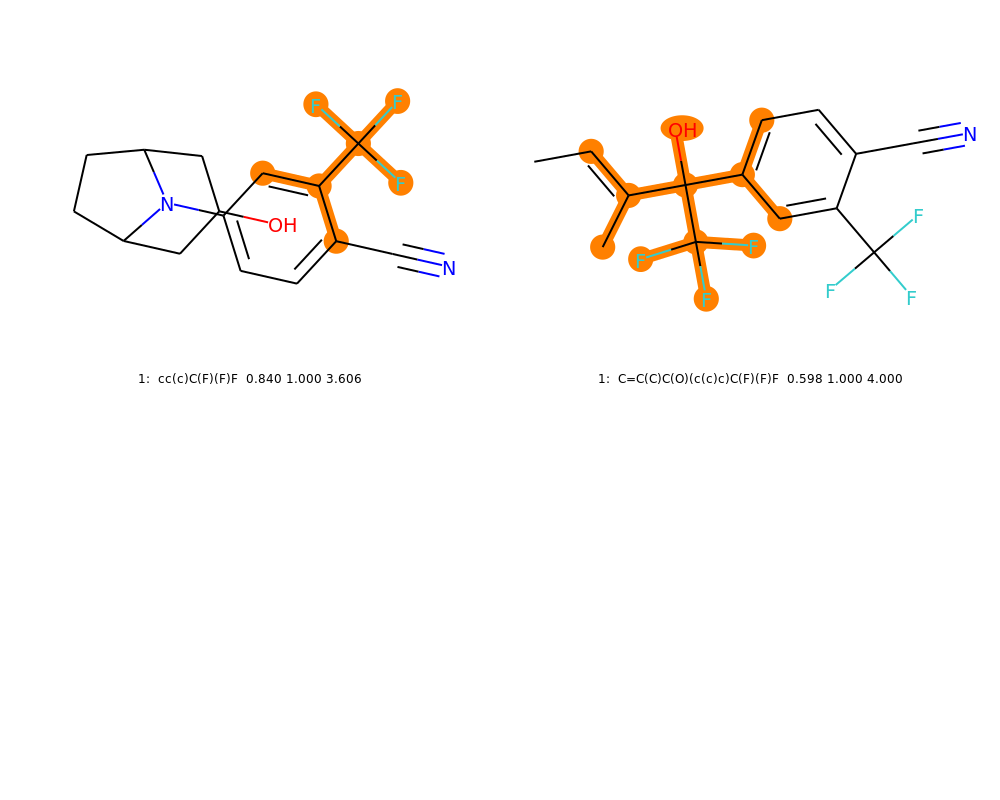

Supplement: Supplementary file 4 — Supplementary file4 (ZIP 111545 KB) [file 10822_2021_421_MOESM4_ESM.zip › 053/tp_cluster_1/tp1_mols_4.png]

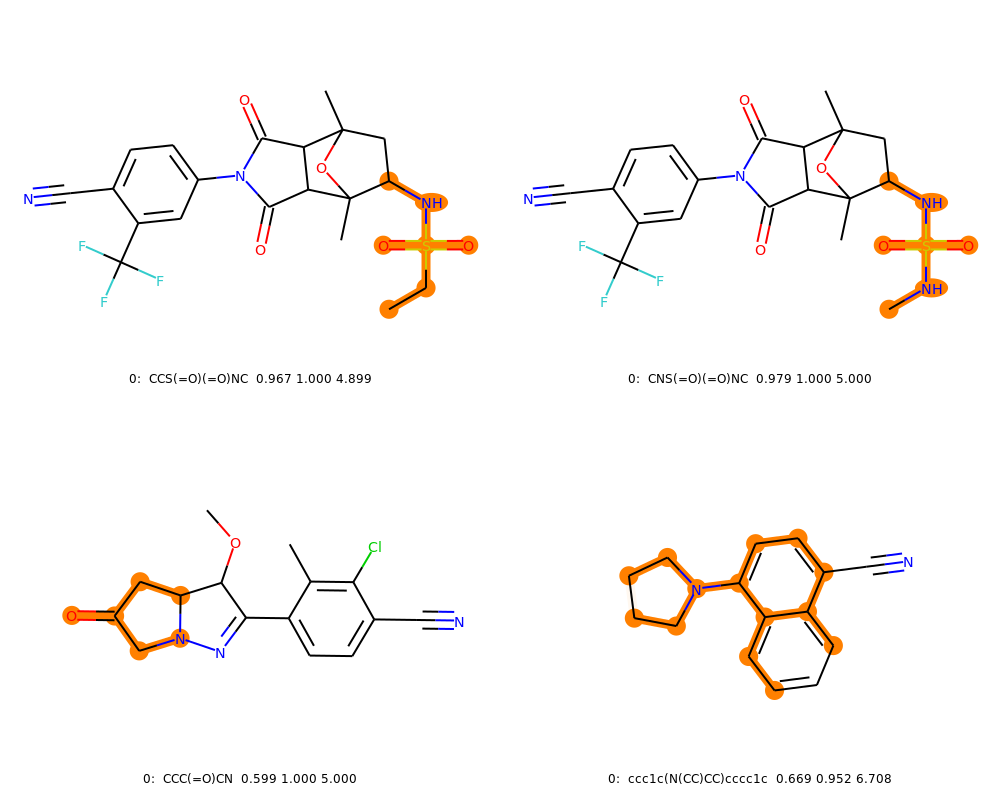

Supplement: Supplementary file 4 — Supplementary file4 (ZIP 111545 KB) [file 10822_2021_421_MOESM4_ESM.zip › 053/tp_cluster_0/tp0_mols_20.png]

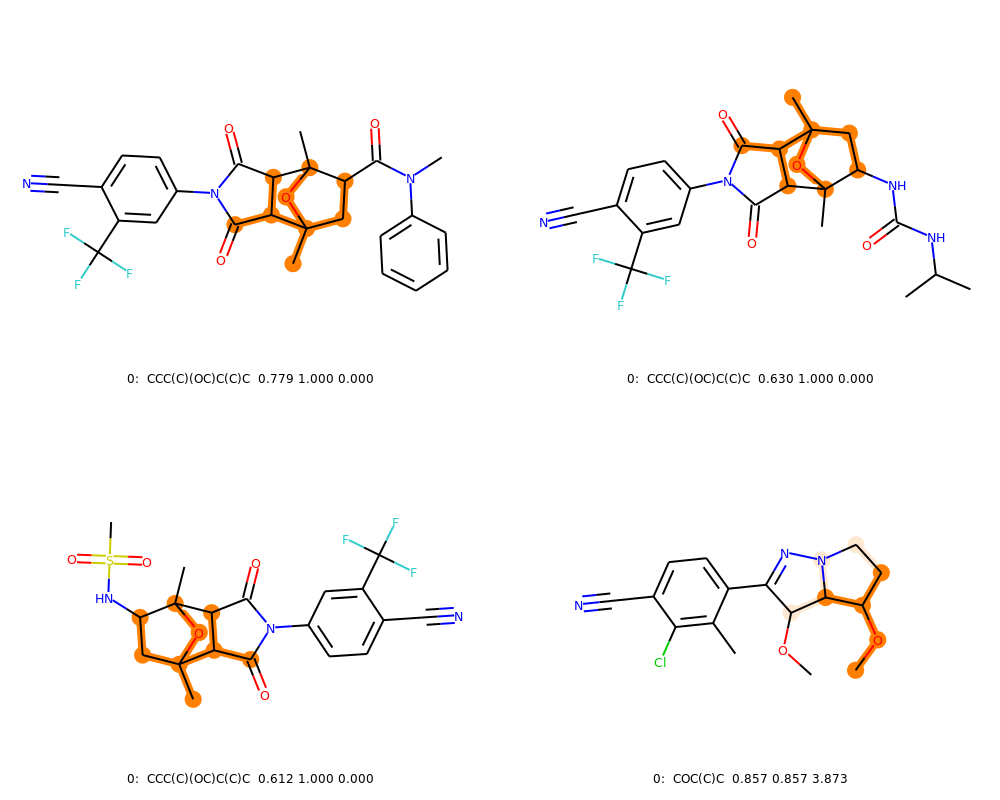

Supplement: Supplementary file 4 — Supplementary file4 (ZIP 111545 KB) [file 10822_2021_421_MOESM4_ESM.zip › 053/tp_cluster_0/tp0_mols_4.png]

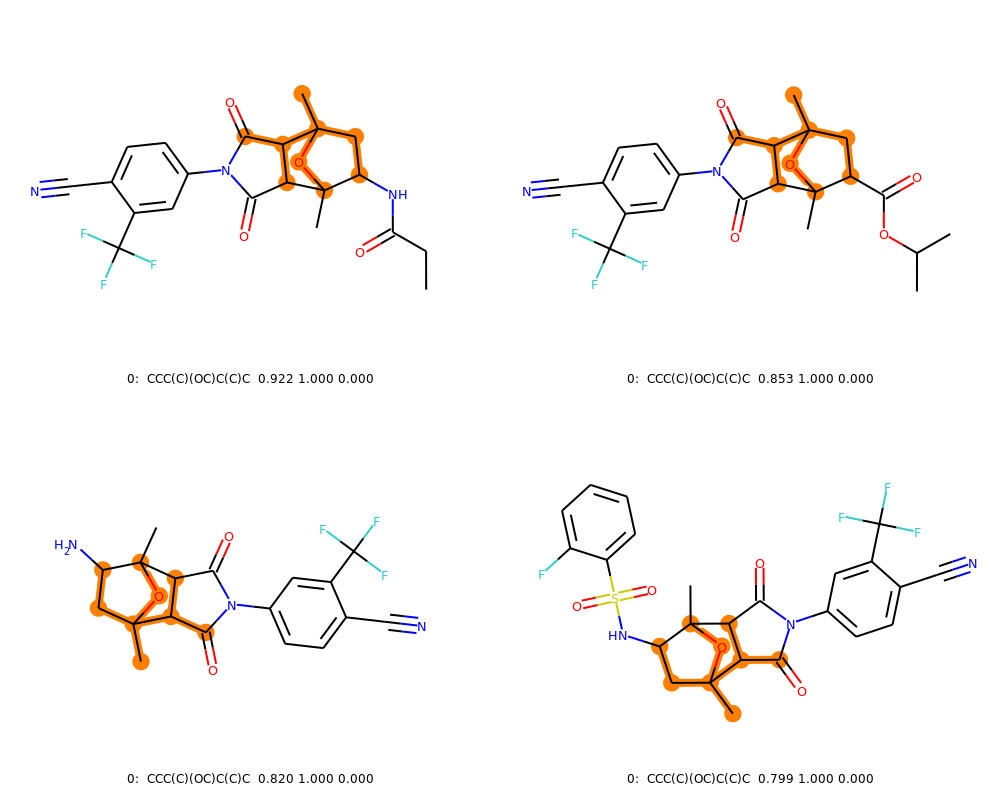

Supplement: Supplementary file 4 — Supplementary file4 (ZIP 111545 KB) [file 10822_2021_421_MOESM4_ESM.zip › 053/tp_cluster_0/tp0_mols_0.png]

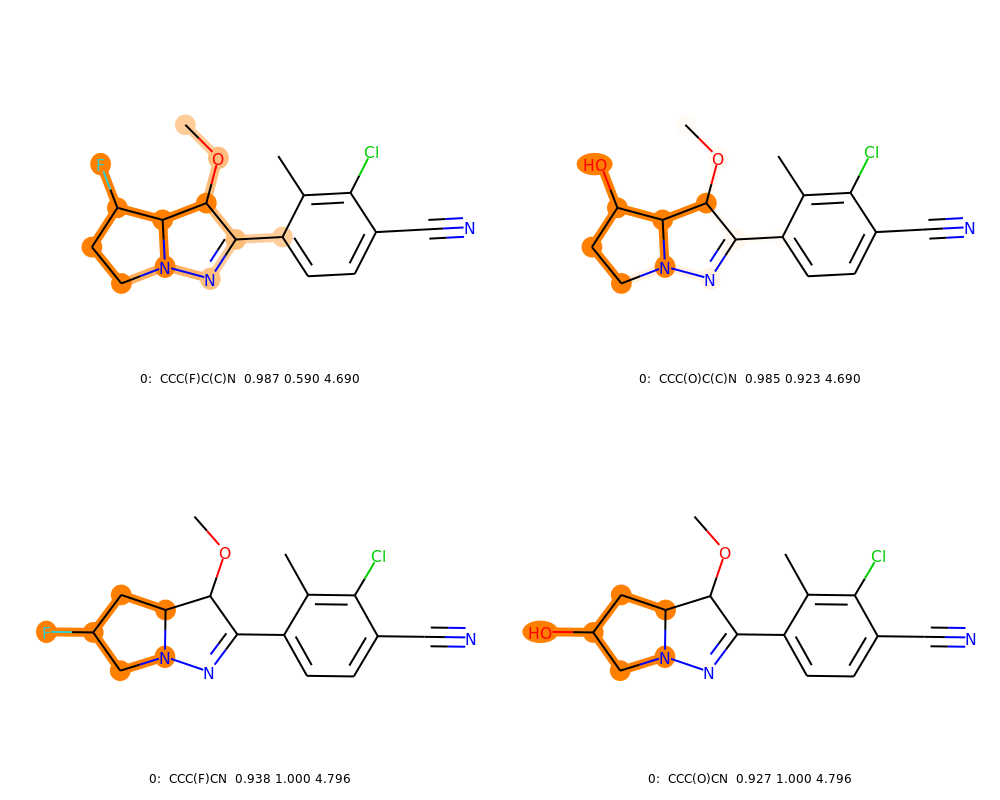

Supplement: Supplementary file 4 — Supplementary file4 (ZIP 111545 KB) [file 10822_2021_421_MOESM4_ESM.zip › 053/tp_cluster_0/tp0_mols_16.png]

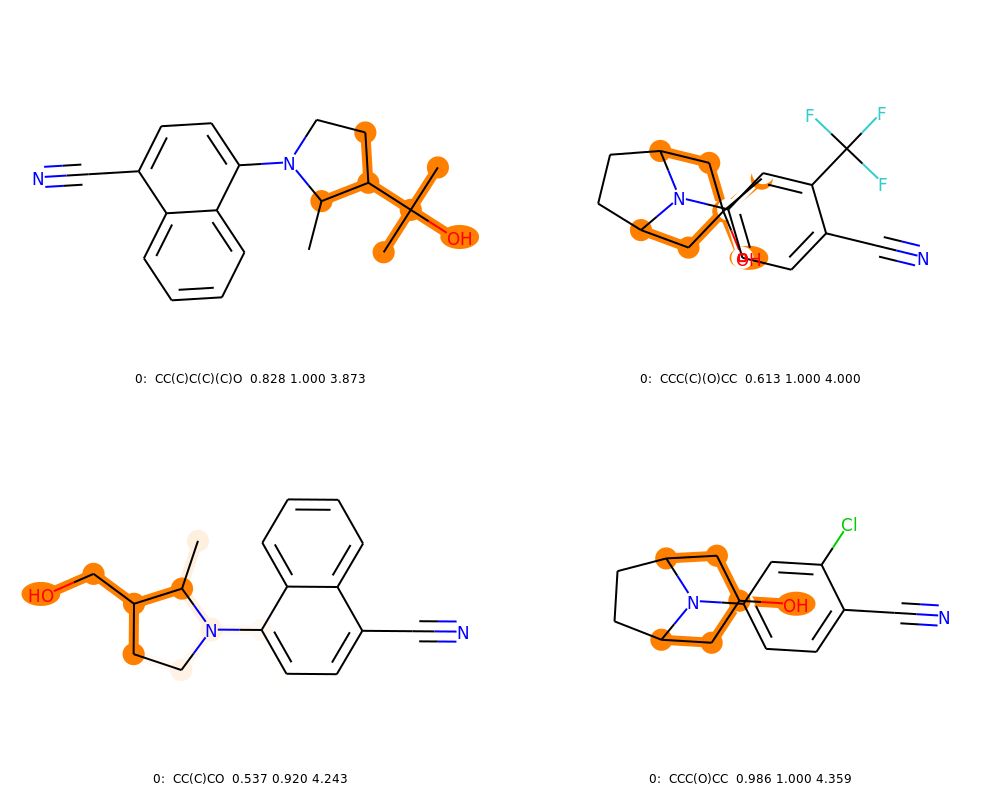

Supplement: Supplementary file 4 — Supplementary file4 (ZIP 111545 KB) [file 10822_2021_421_MOESM4_ESM.zip › 053/tp_cluster_0/tp0_mols_8.png]

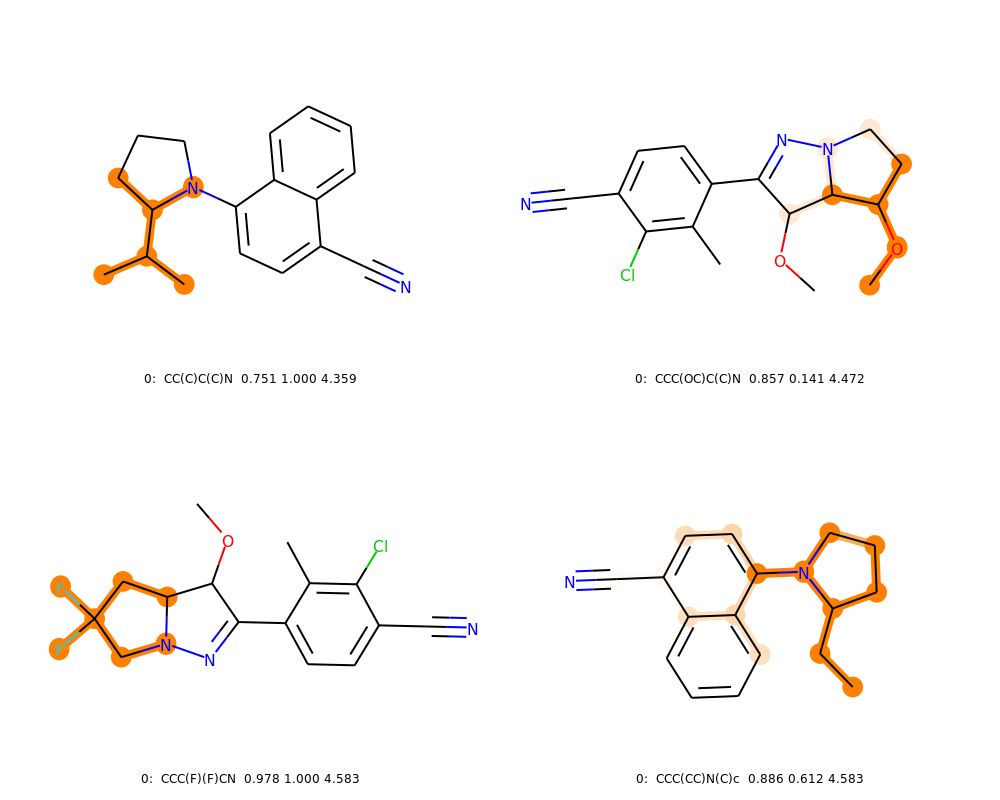

Supplement: Supplementary file 4 — Supplementary file4 (ZIP 111545 KB) [file 10822_2021_421_MOESM4_ESM.zip › 053/tp_cluster_0/tp0_mols_12.png]

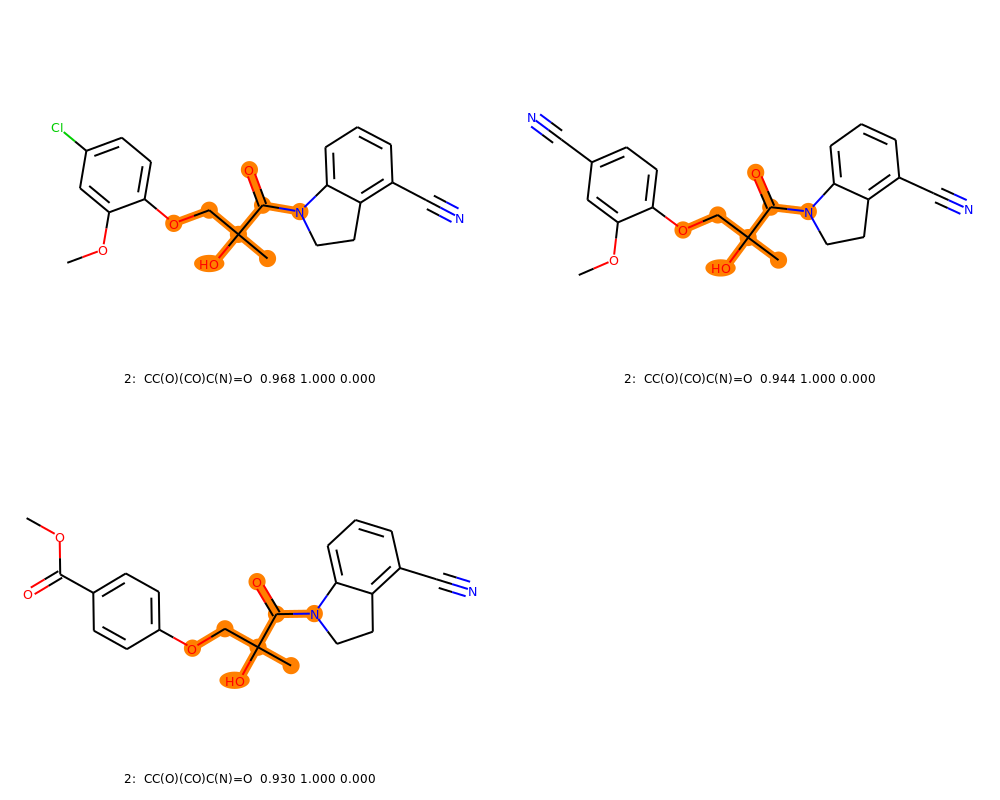

Supplement: Supplementary file 4 — Supplementary file4 (ZIP 111545 KB) [file 10822_2021_421_MOESM4_ESM.zip › 053/tp_cluster_2/tp2_mols_0.png]

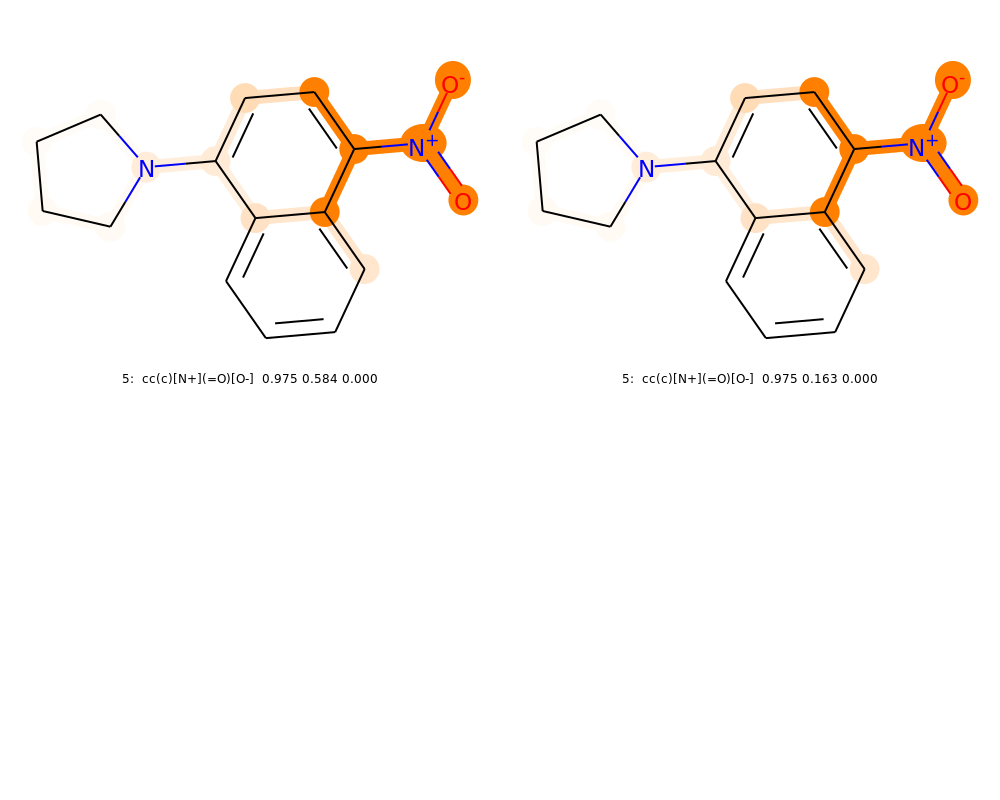

Supplement: Supplementary file 4 — Supplementary file4 (ZIP 111545 KB) [file 10822_2021_421_MOESM4_ESM.zip › 053/tp_cluster_5/tp5_mols_0.png]

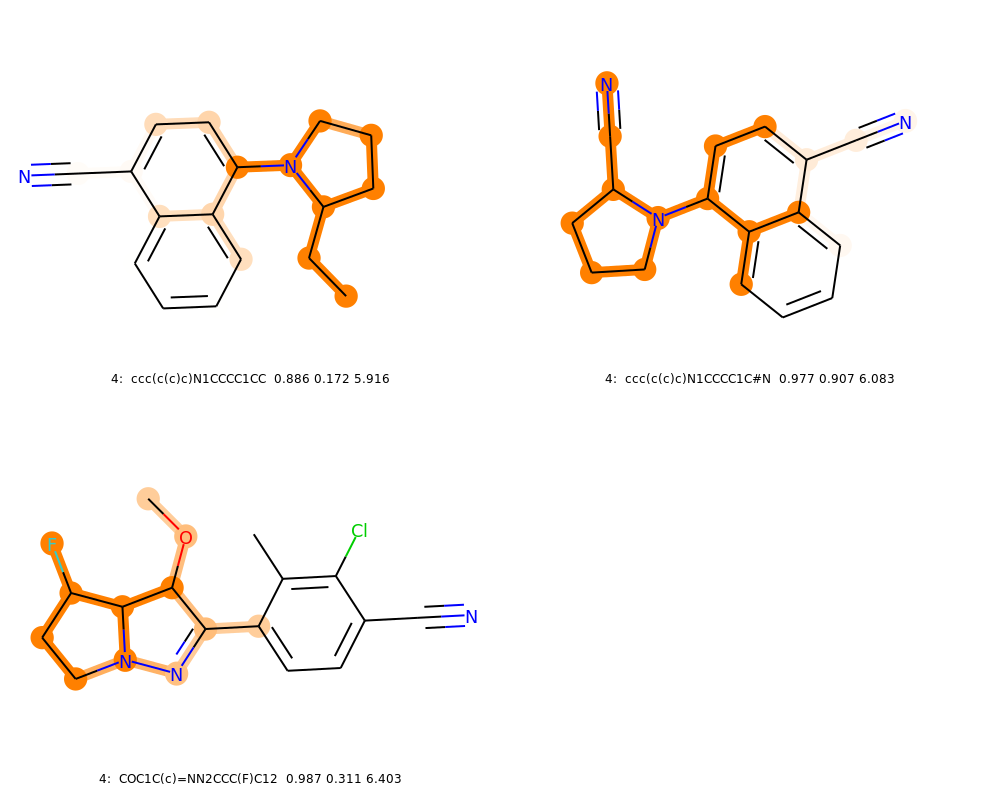

Supplement: Supplementary file 4 — Supplementary file4 (ZIP 111545 KB) [file 10822_2021_421_MOESM4_ESM.zip › 053/tp_cluster_4/tp4_mols_4.png]

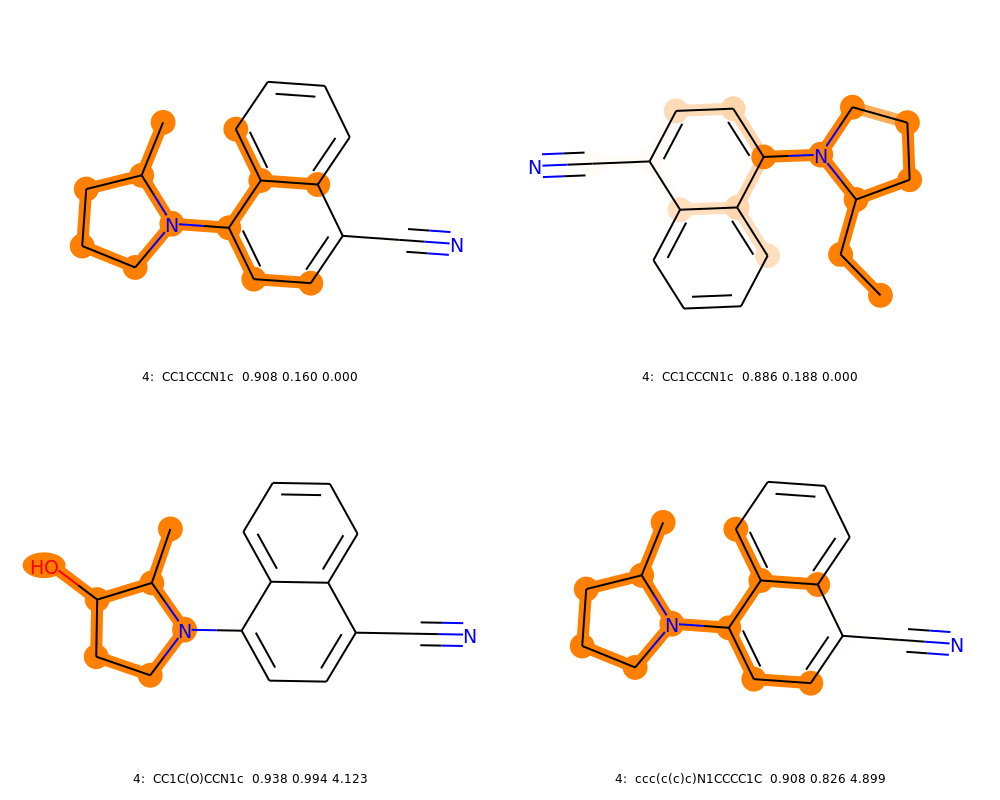

Supplement: Supplementary file 4 — Supplementary file4 (ZIP 111545 KB) [file 10822_2021_421_MOESM4_ESM.zip › 053/tp_cluster_4/tp4_mols_0.png]

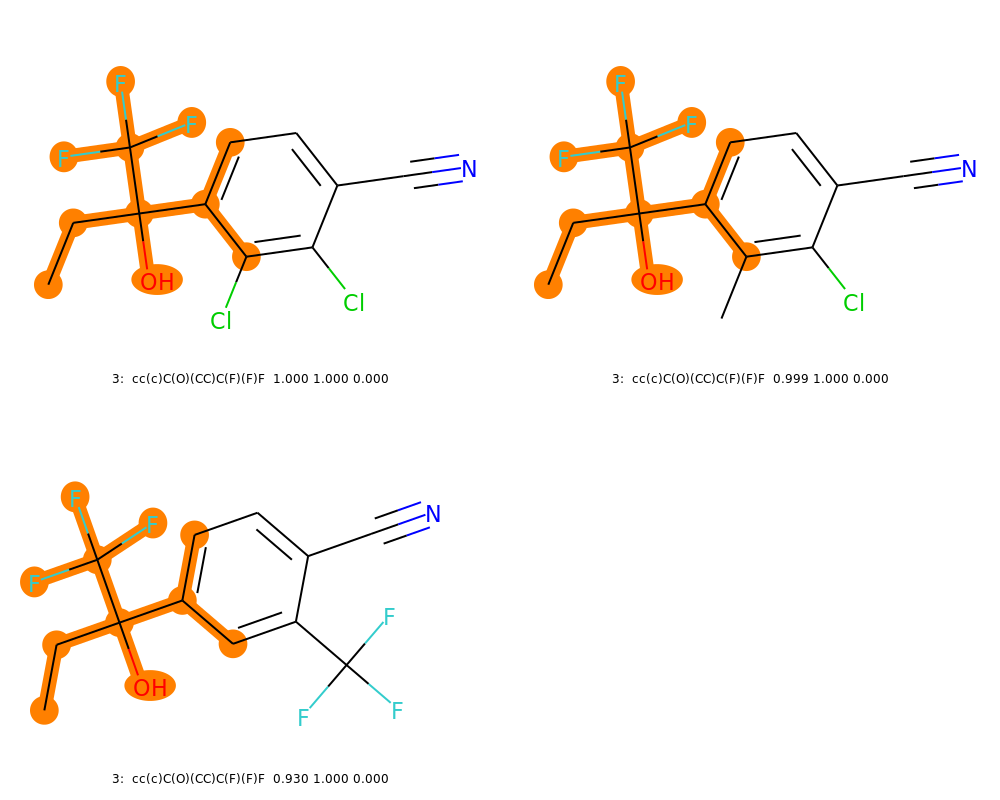

Supplement: Supplementary file 4 — Supplementary file4 (ZIP 111545 KB) [file 10822_2021_421_MOESM4_ESM.zip › 053/tp_cluster_3/tp3_mols_0.png]

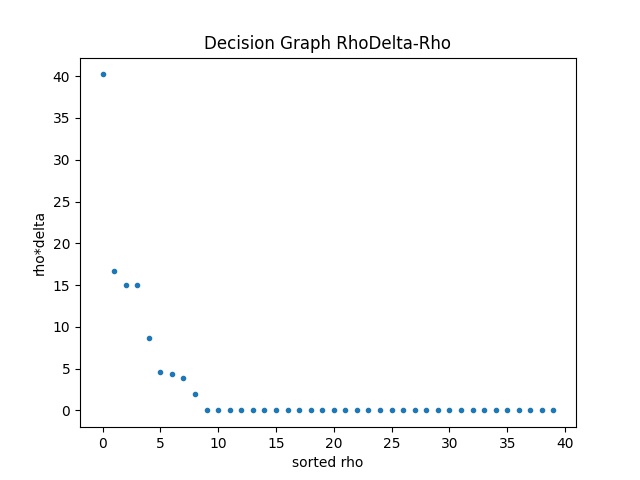

Supplement: Supplementary file 4 — Supplementary file4 (ZIP 111545 KB) [file 10822_2021_421_MOESM4_ESM.zip › 058/tp_decision_graphs/RhoDelta-Rho.jpg]

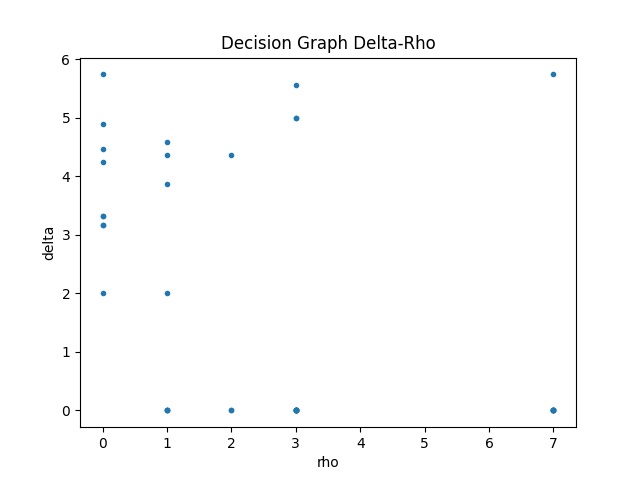

Supplement: Supplementary file 4 — Supplementary file4 (ZIP 111545 KB) [file 10822_2021_421_MOESM4_ESM.zip › 058/tp_decision_graphs/Delta-Rho.jpg]

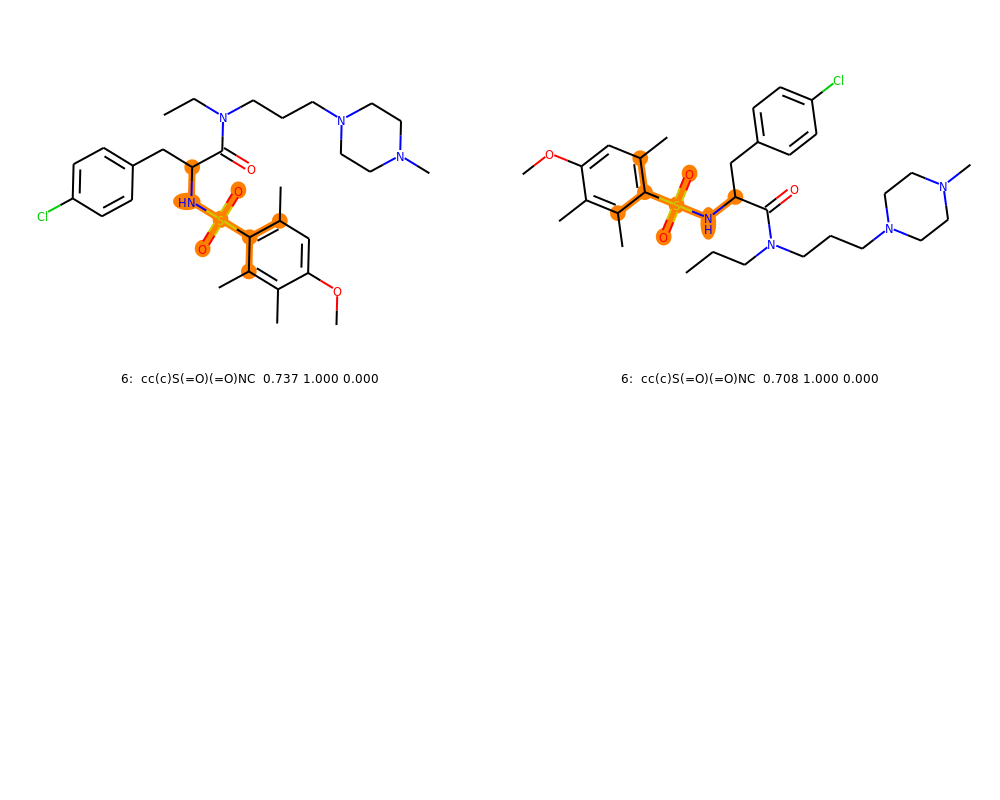

Supplement: Supplementary file 4 — Supplementary file4 (ZIP 111545 KB) [file 10822_2021_421_MOESM4_ESM.zip › 058/tp_cluster_6/tp6_mols_0.png]

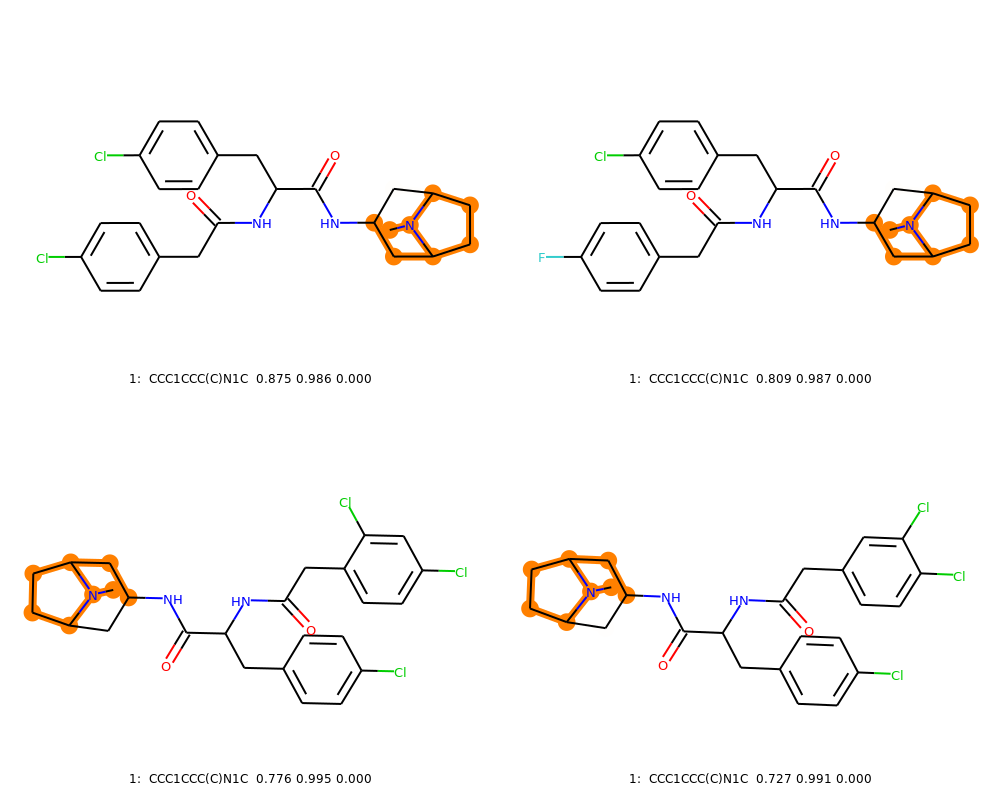

Supplement: Supplementary file 4 — Supplementary file4 (ZIP 111545 KB) [file 10822_2021_421_MOESM4_ESM.zip › 058/tp_cluster_1/tp1_mols_0.png]

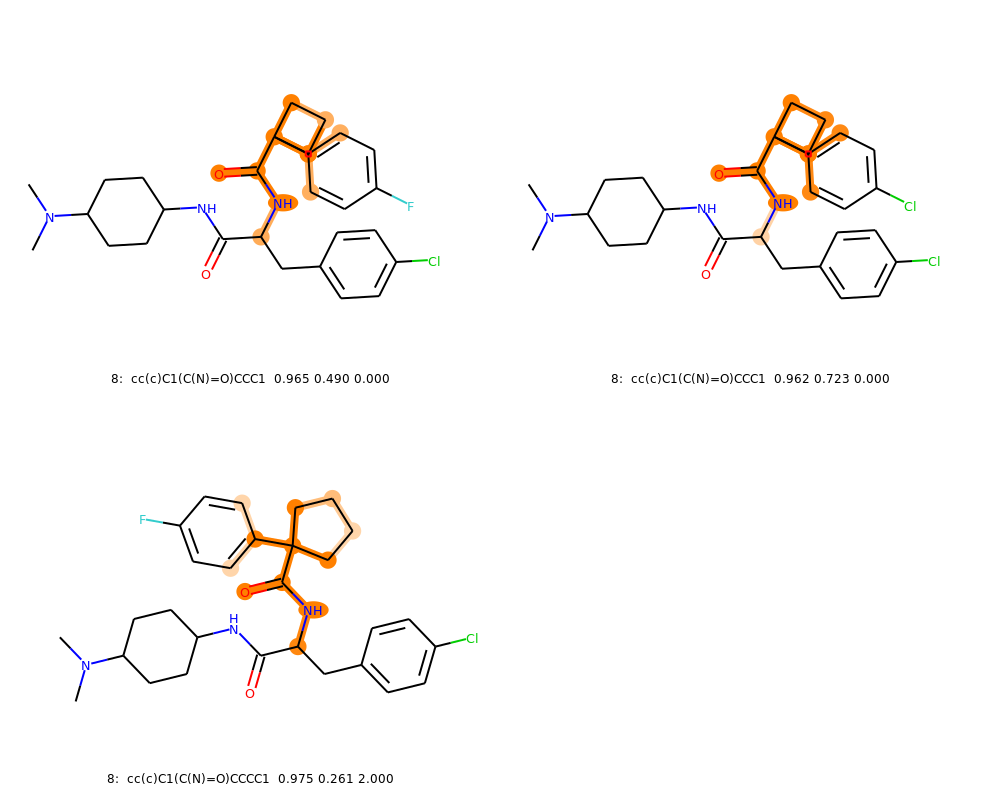

Supplement: Supplementary file 4 — Supplementary file4 (ZIP 111545 KB) [file 10822_2021_421_MOESM4_ESM.zip › 058/tp_cluster_8/tp8_mols_0.png]

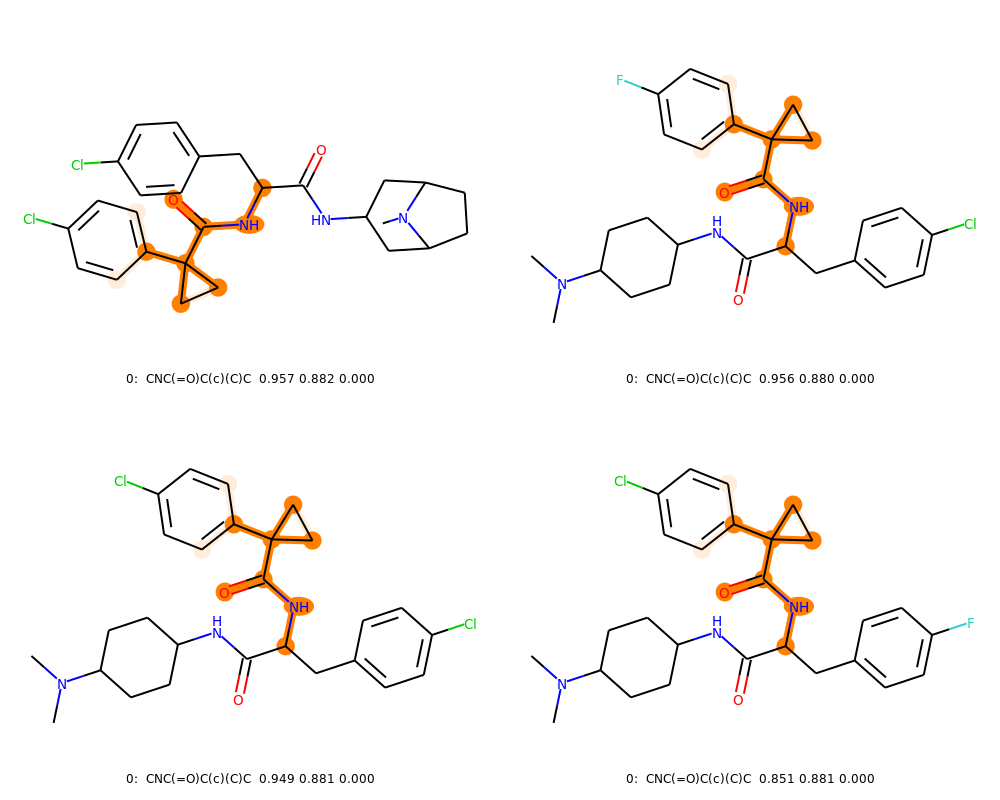

Supplement: Supplementary file 4 — Supplementary file4 (ZIP 111545 KB) [file 10822_2021_421_MOESM4_ESM.zip › 058/tp_cluster_0/tp0_mols_4.png]

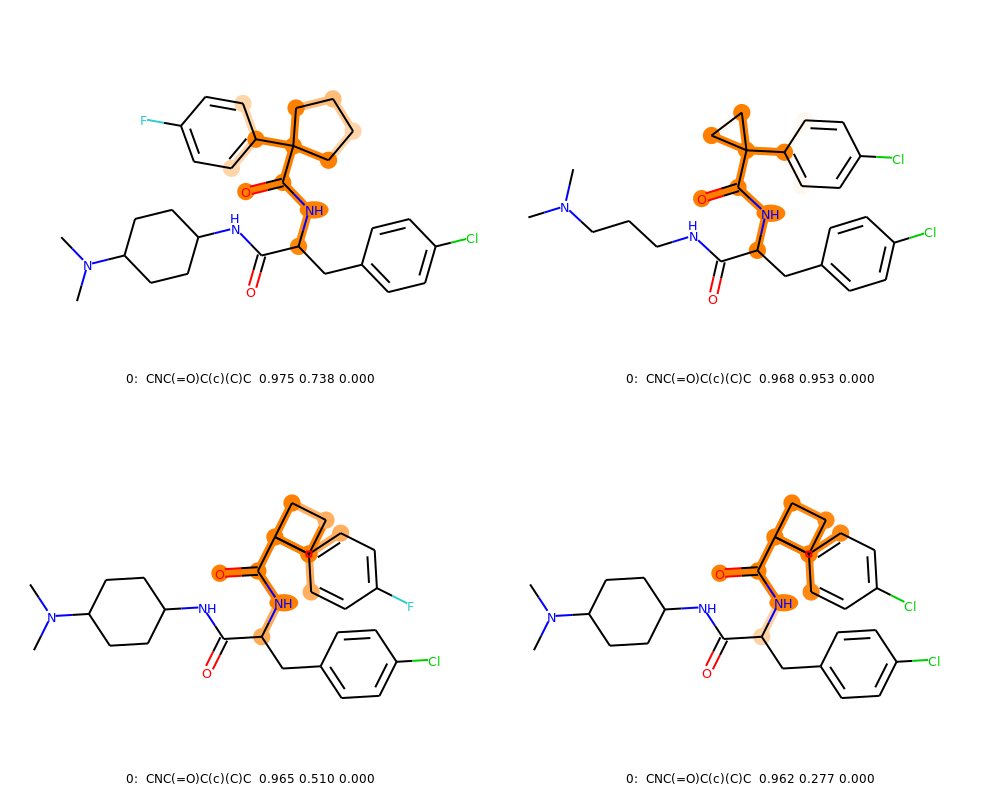

Supplement: Supplementary file 4 — Supplementary file4 (ZIP 111545 KB) [file 10822_2021_421_MOESM4_ESM.zip › 058/tp_cluster_0/tp0_mols_0.png]

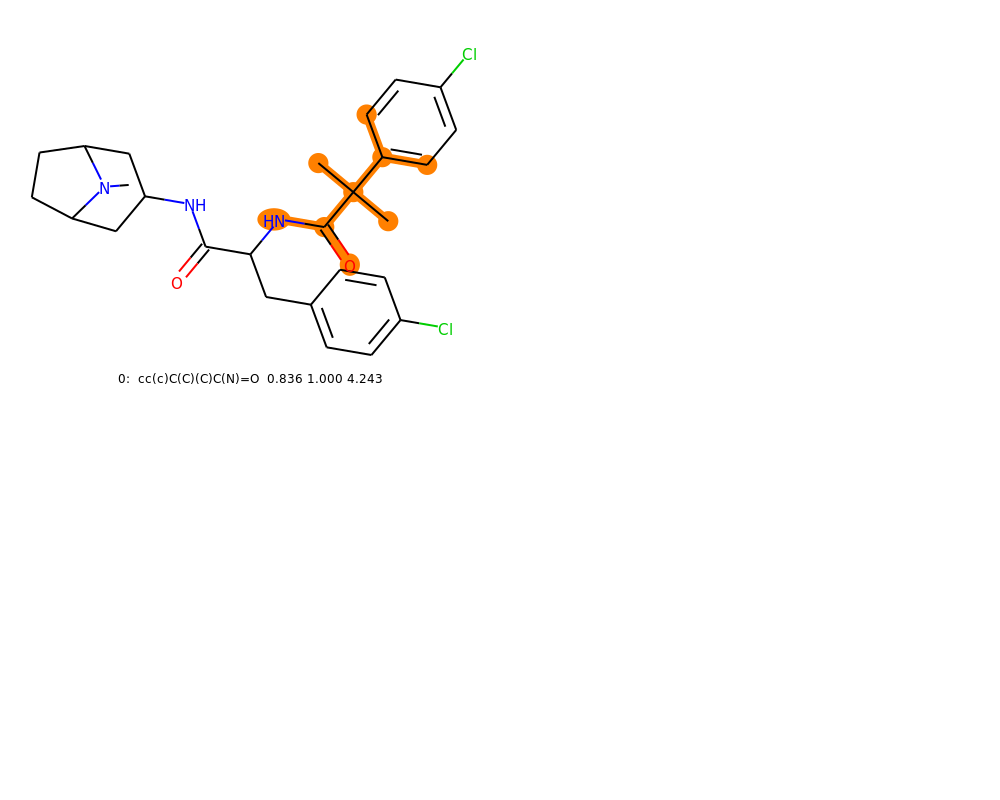

Supplement: Supplementary file 4 — Supplementary file4 (ZIP 111545 KB) [file 10822_2021_421_MOESM4_ESM.zip › 058/tp_cluster_0/tp0_mols_8.png]

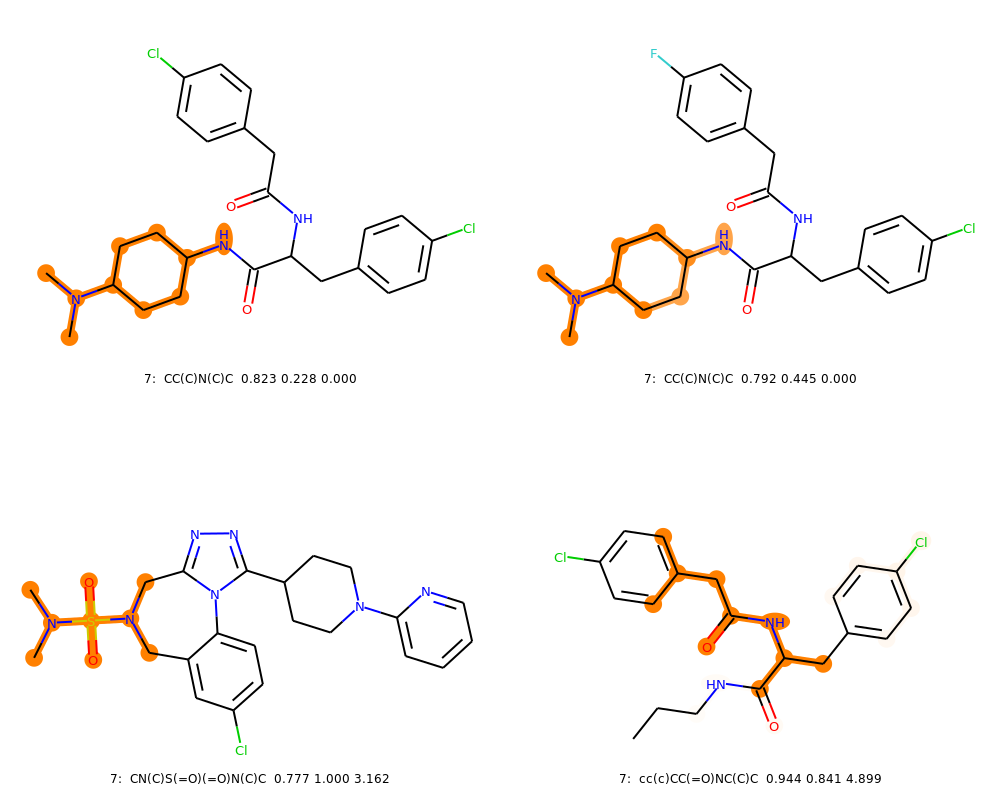

Supplement: Supplementary file 4 — Supplementary file4 (ZIP 111545 KB) [file 10822_2021_421_MOESM4_ESM.zip › 058/tp_cluster_7/tp7_mols_0.png]

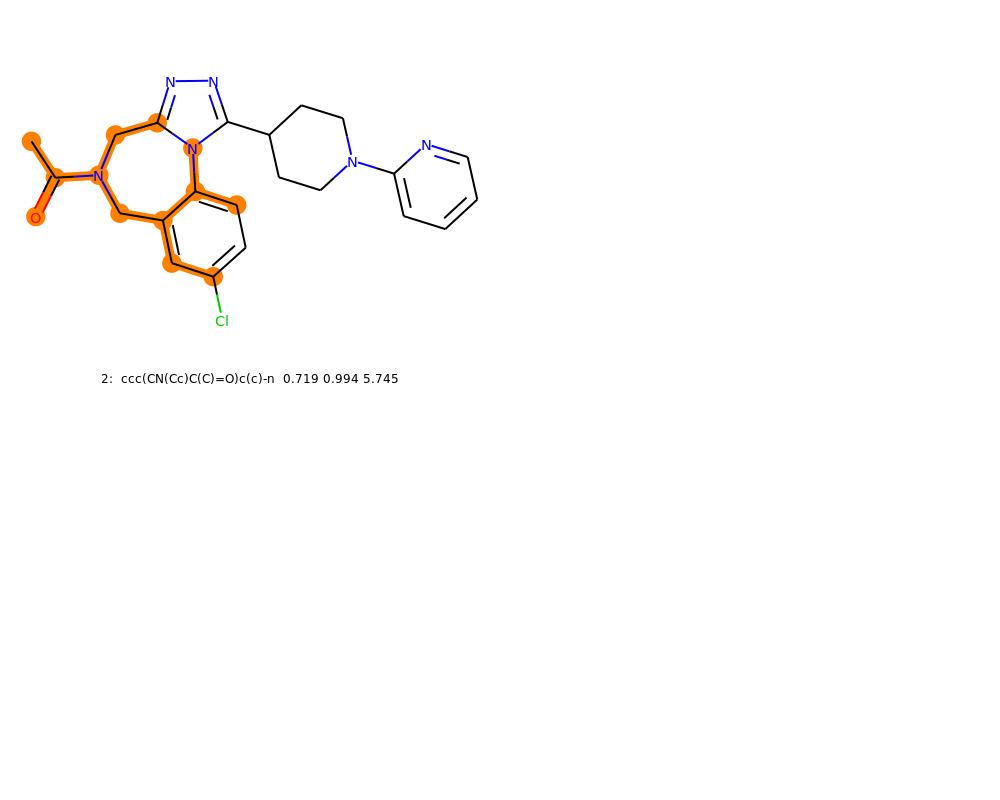

Supplement: Supplementary file 4 — Supplementary file4 (ZIP 111545 KB) [file 10822_2021_421_MOESM4_ESM.zip › 058/tp_cluster_2/tp2_mols_4.png]

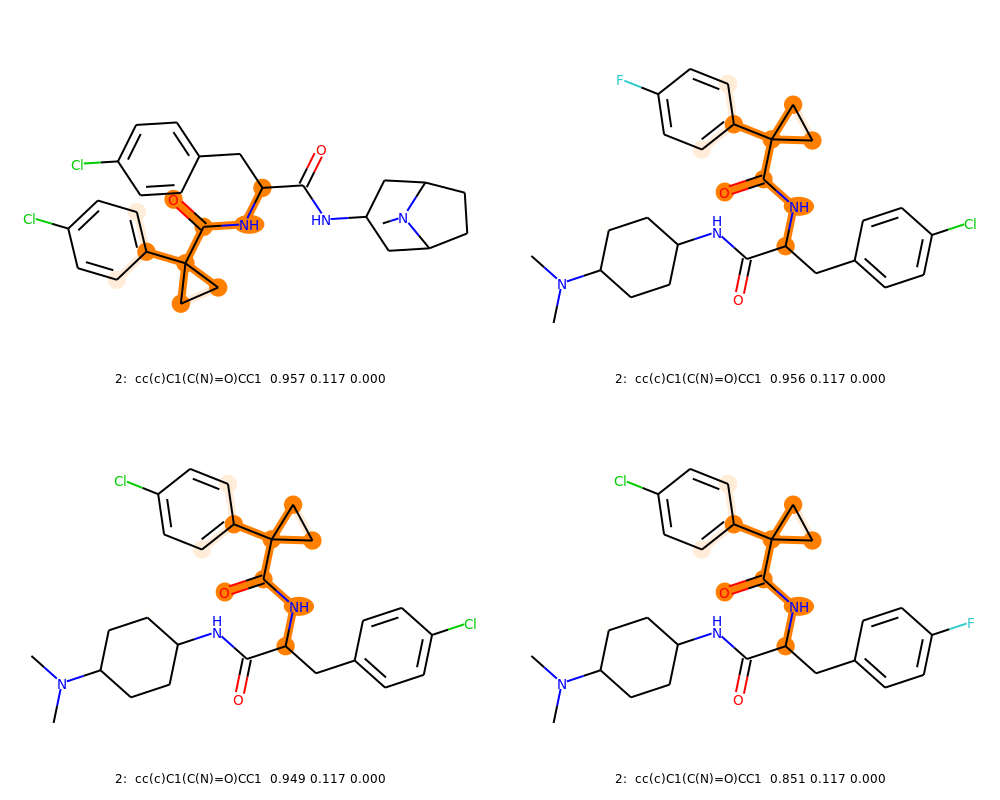

Supplement: Supplementary file 4 — Supplementary file4 (ZIP 111545 KB) [file 10822_2021_421_MOESM4_ESM.zip › 058/tp_cluster_2/tp2_mols_0.png]

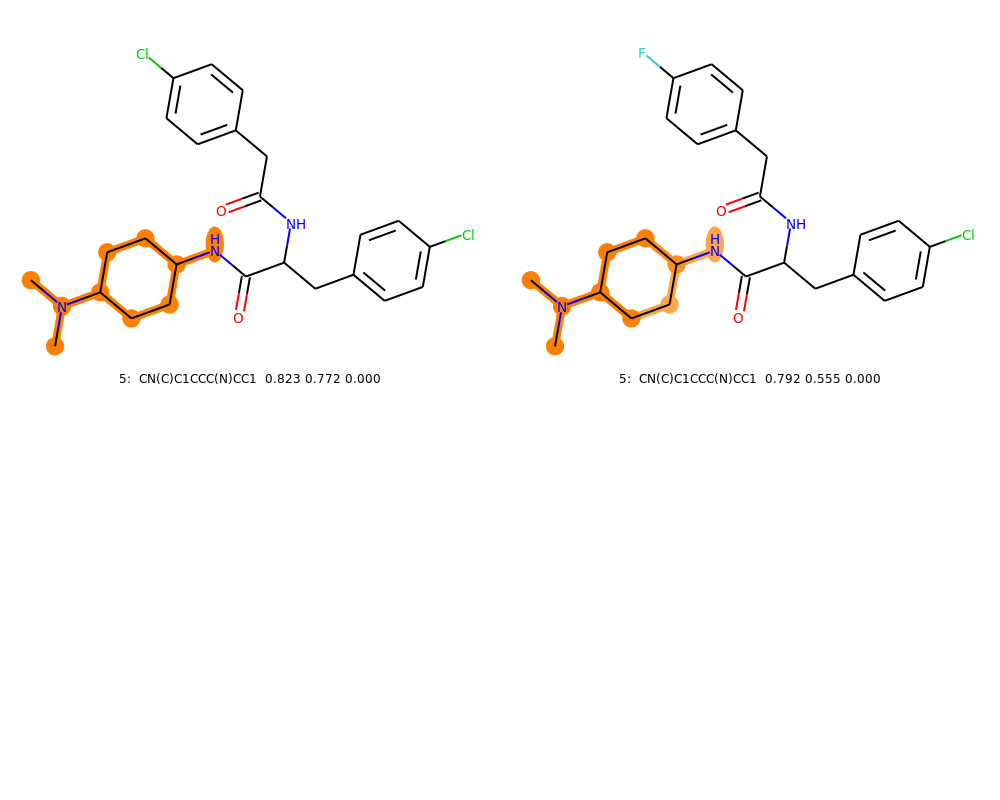

Supplement: Supplementary file 4 — Supplementary file4 (ZIP 111545 KB) [file 10822_2021_421_MOESM4_ESM.zip › 058/tp_cluster_5/tp5_mols_0.png]

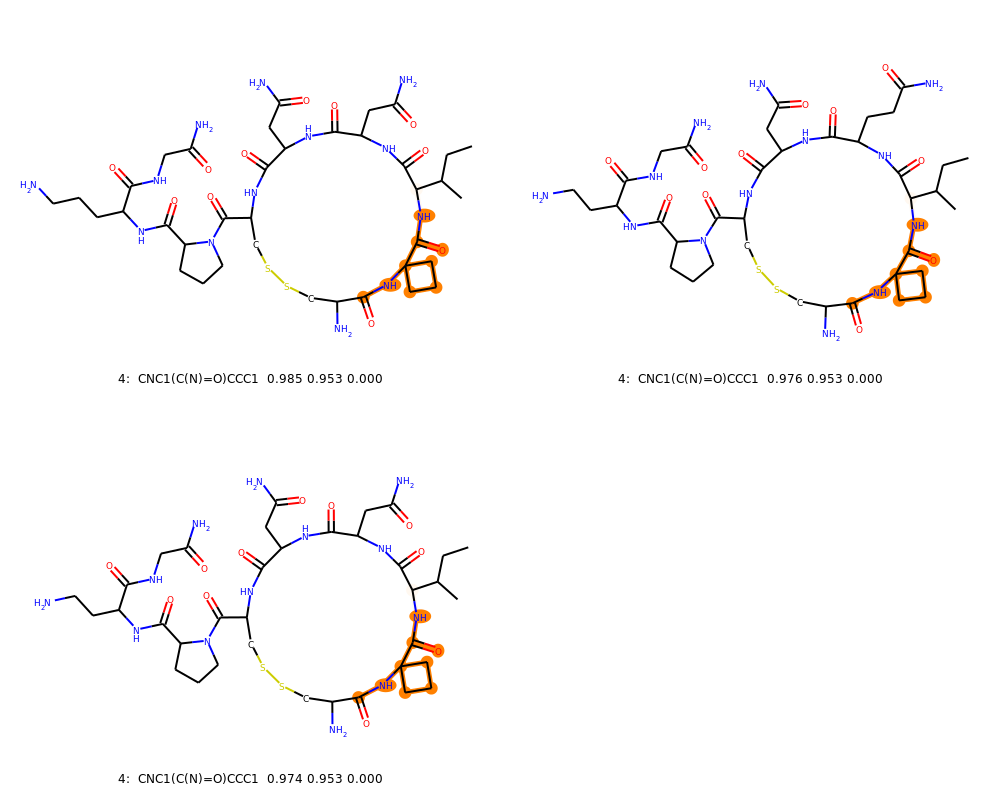

Supplement: Supplementary file 4 — Supplementary file4 (ZIP 111545 KB) [file 10822_2021_421_MOESM4_ESM.zip › 058/tp_cluster_4/tp4_mols_0.png]

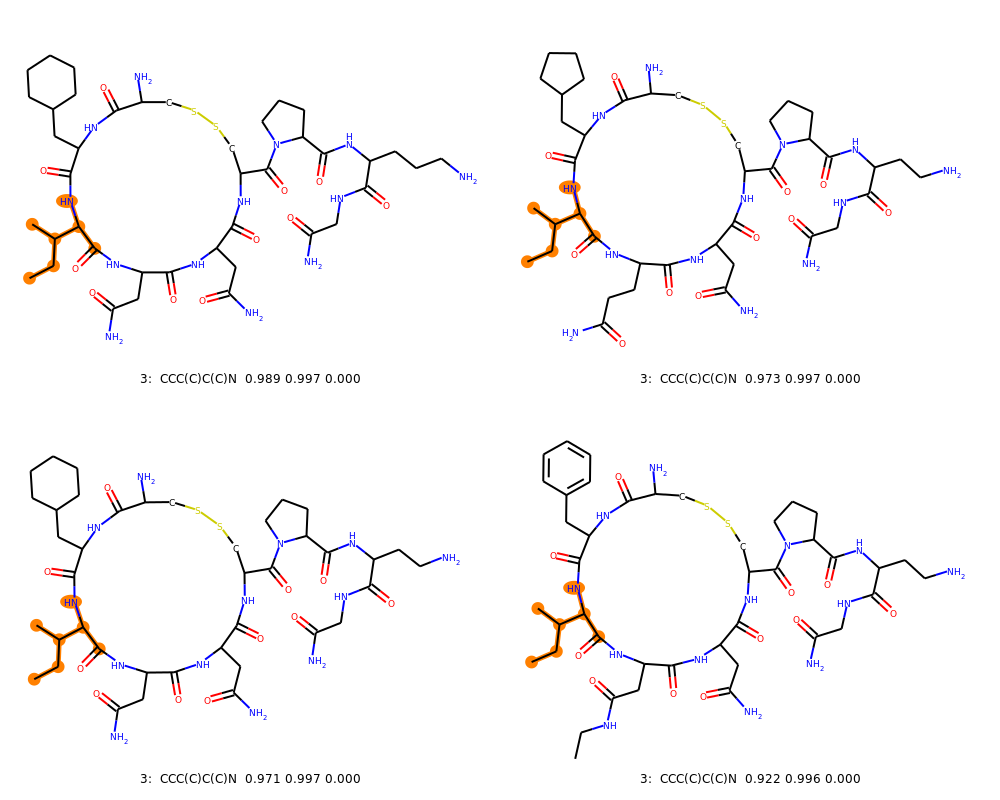

Supplement: Supplementary file 4 — Supplementary file4 (ZIP 111545 KB) [file 10822_2021_421_MOESM4_ESM.zip › 058/tp_cluster_3/tp3_mols_0.png]

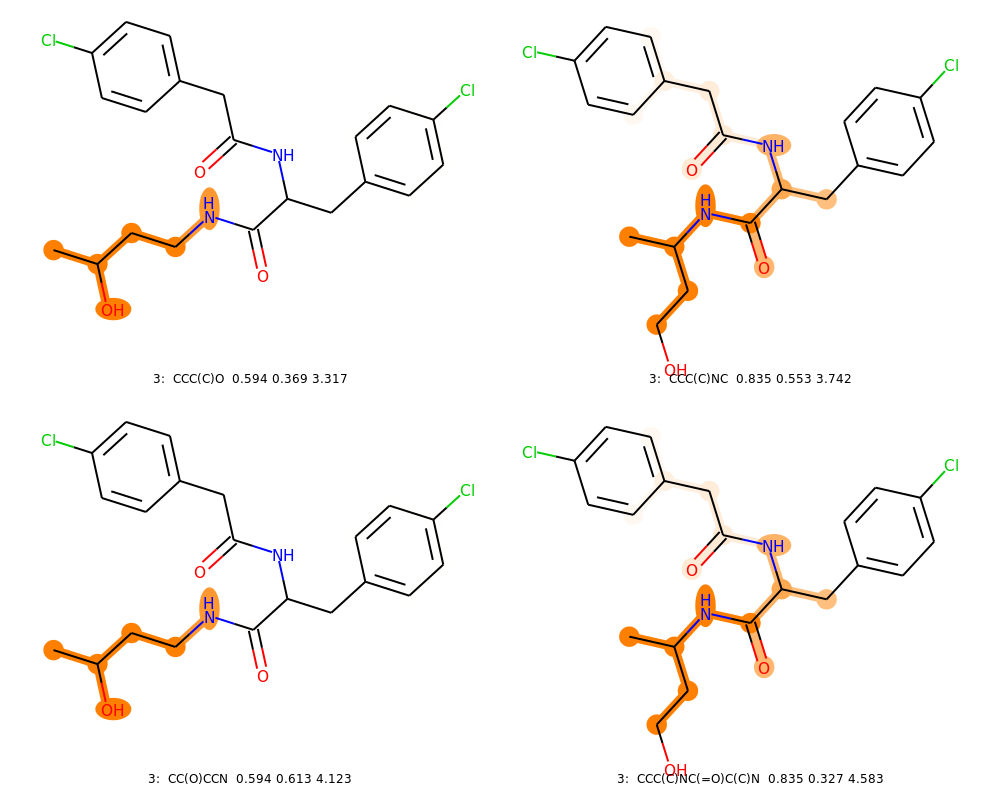

Supplement: Supplementary file 4 — Supplementary file4 (ZIP 111545 KB) [file 10822_2021_421_MOESM4_ESM.zip › 058/tp_cluster_3/tp3_mols_4.png]

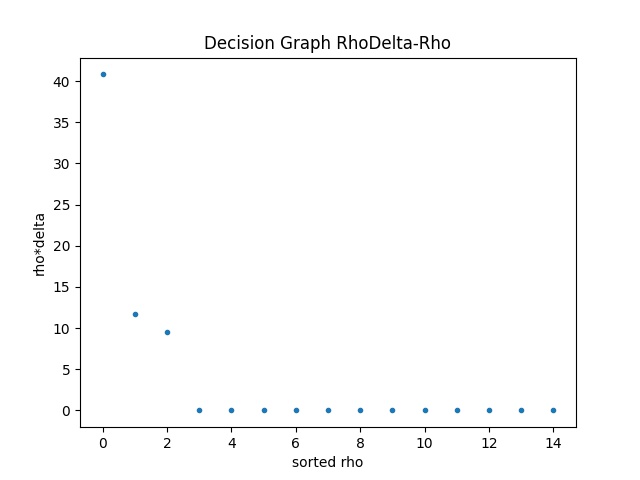

Supplement: Supplementary file 4 — Supplementary file4 (ZIP 111545 KB) [file 10822_2021_421_MOESM4_ESM.zip › 063/tp_decision_graphs/RhoDelta-Rho.jpg]

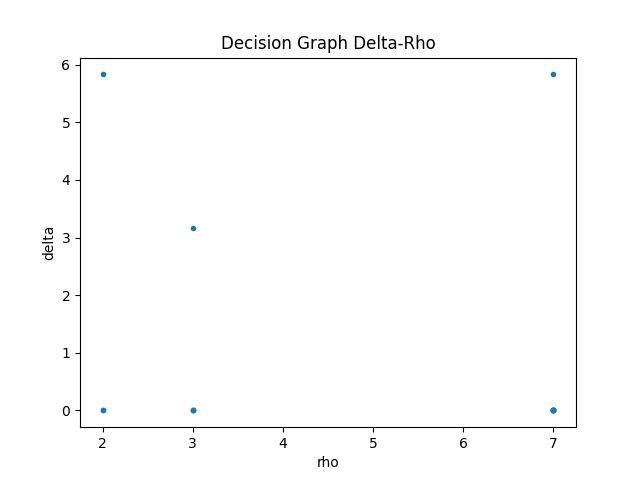

Supplement: Supplementary file 4 — Supplementary file4 (ZIP 111545 KB) [file 10822_2021_421_MOESM4_ESM.zip › 063/tp_decision_graphs/Delta-Rho.jpg]

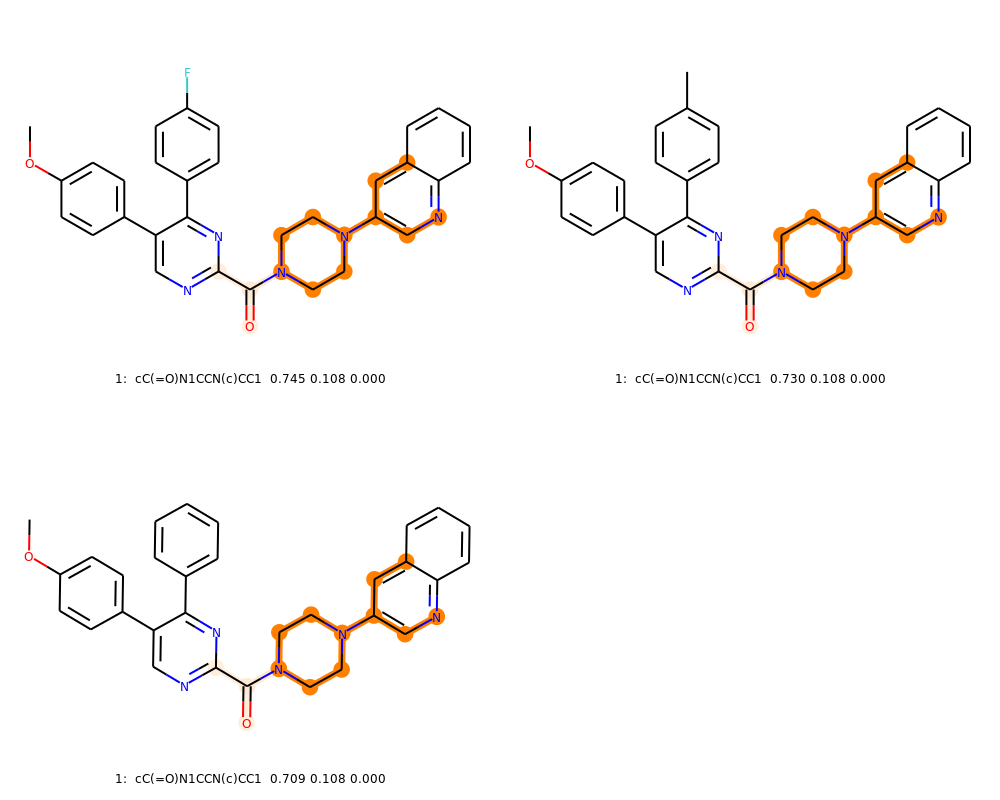

Supplement: Supplementary file 4 — Supplementary file4 (ZIP 111545 KB) [file 10822_2021_421_MOESM4_ESM.zip › 063/tp_cluster_1/tp1_mols_0.png]

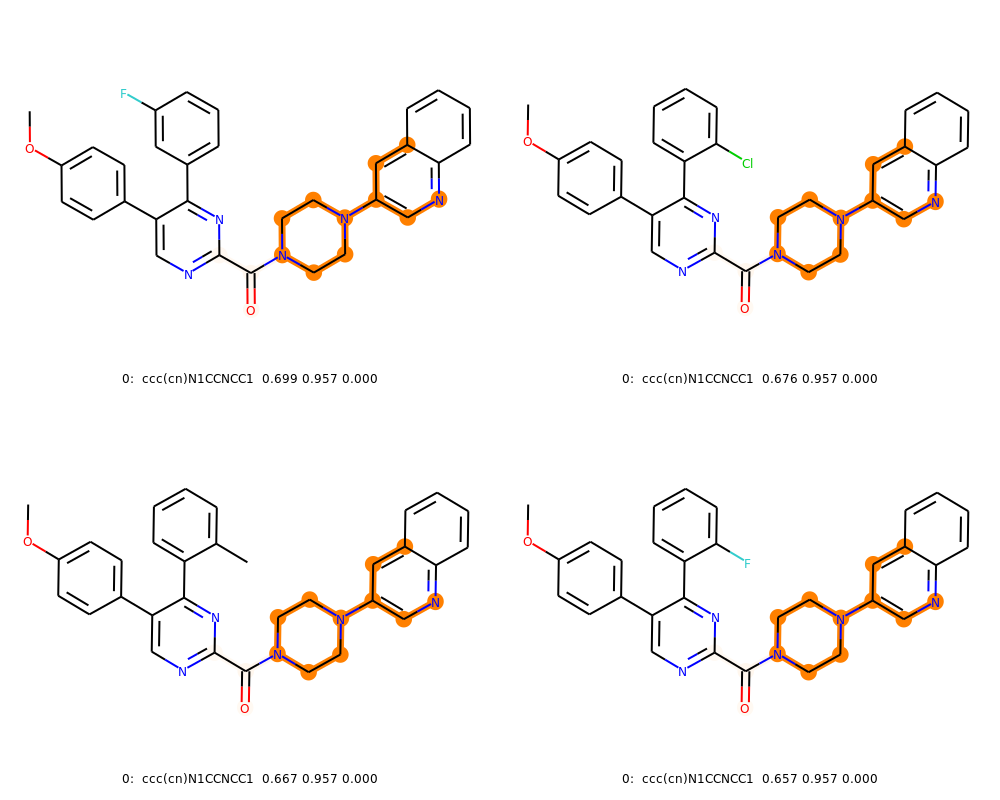

Supplement: Supplementary file 4 — Supplementary file4 (ZIP 111545 KB) [file 10822_2021_421_MOESM4_ESM.zip › 063/tp_cluster_0/tp0_mols_4.png]

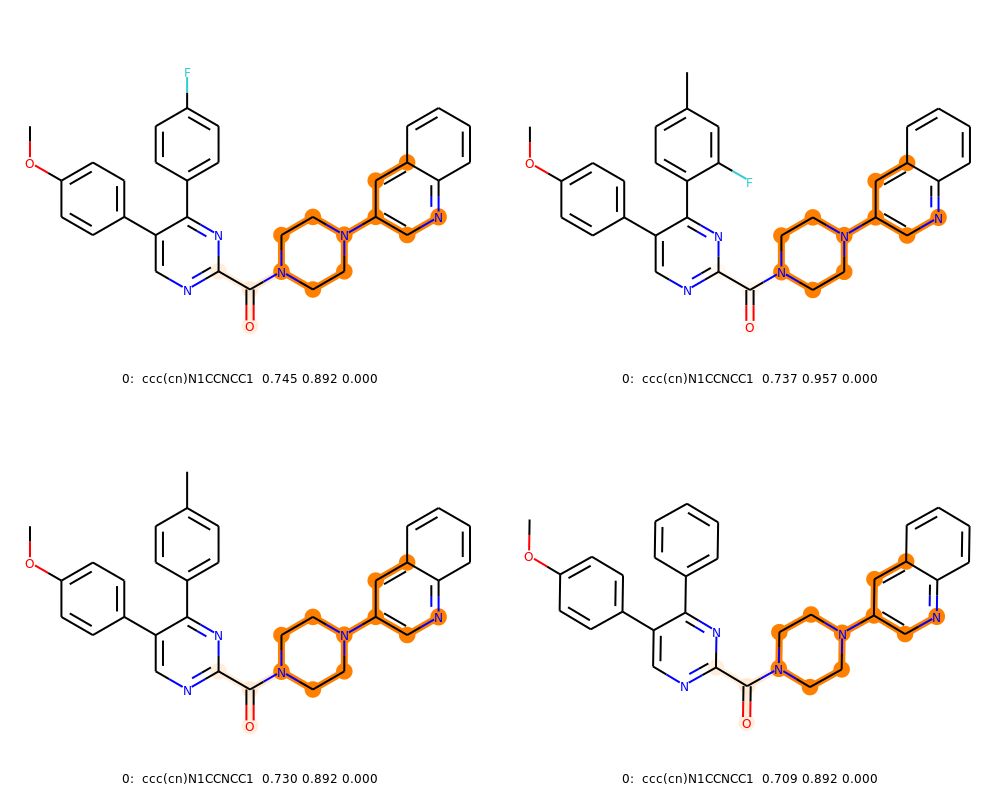

Supplement: Supplementary file 4 — Supplementary file4 (ZIP 111545 KB) [file 10822_2021_421_MOESM4_ESM.zip › 063/tp_cluster_0/tp0_mols_0.png]

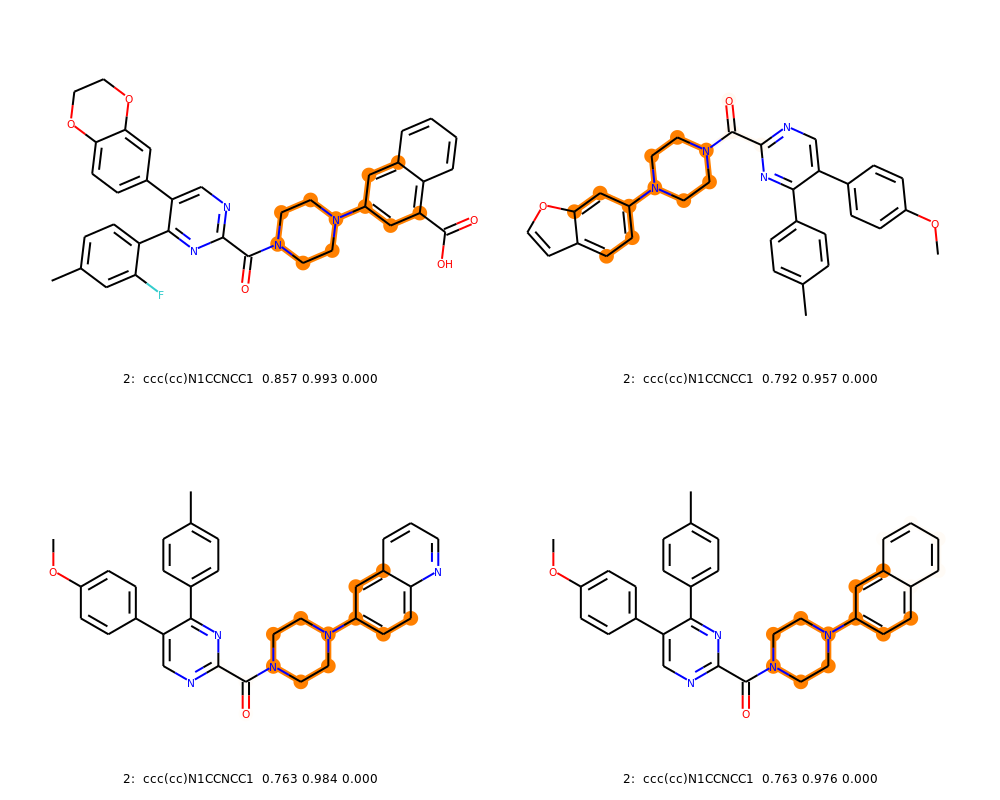

Supplement: Supplementary file 4 — Supplementary file4 (ZIP 111545 KB) [file 10822_2021_421_MOESM4_ESM.zip › 063/tp_cluster_2/tp2_mols_0.png]

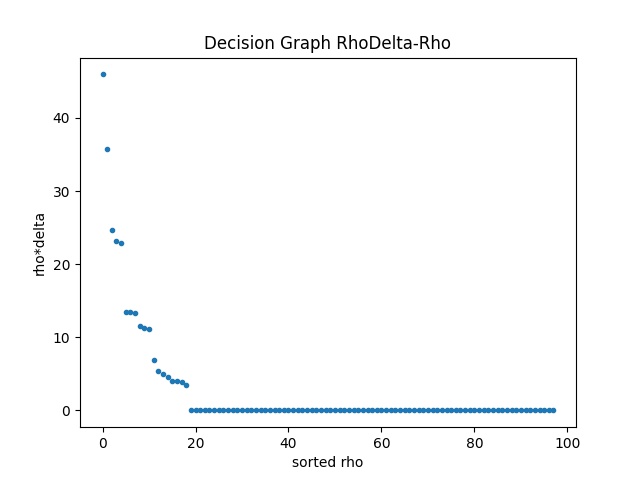

Supplement: Supplementary file 4 — Supplementary file4 (ZIP 111545 KB) [file 10822_2021_421_MOESM4_ESM.zip › 069/tp_decision_graphs/RhoDelta-Rho.jpg]

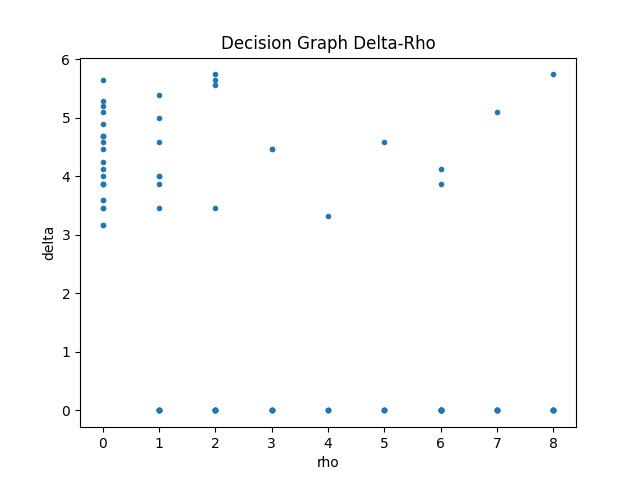

Supplement: Supplementary file 4 — Supplementary file4 (ZIP 111545 KB) [file 10822_2021_421_MOESM4_ESM.zip › 069/tp_decision_graphs/Delta-Rho.jpg]

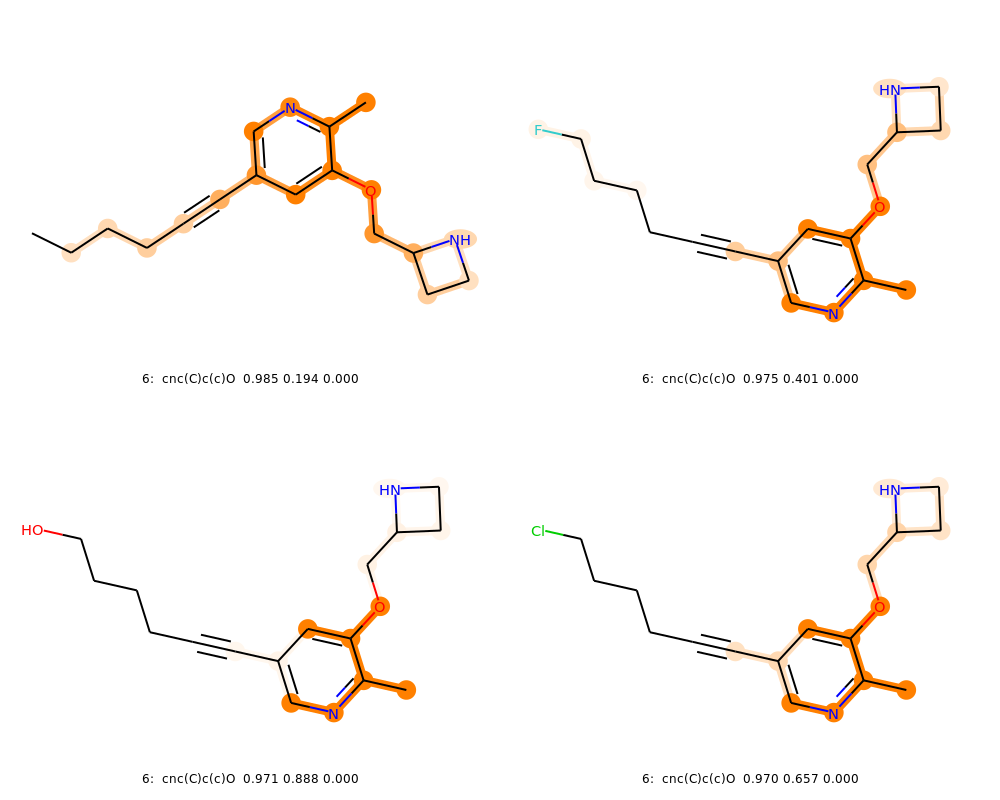

Supplement: Supplementary file 4 — Supplementary file4 (ZIP 111545 KB) [file 10822_2021_421_MOESM4_ESM.zip › 069/tp_cluster_6/tp6_mols_0.png]

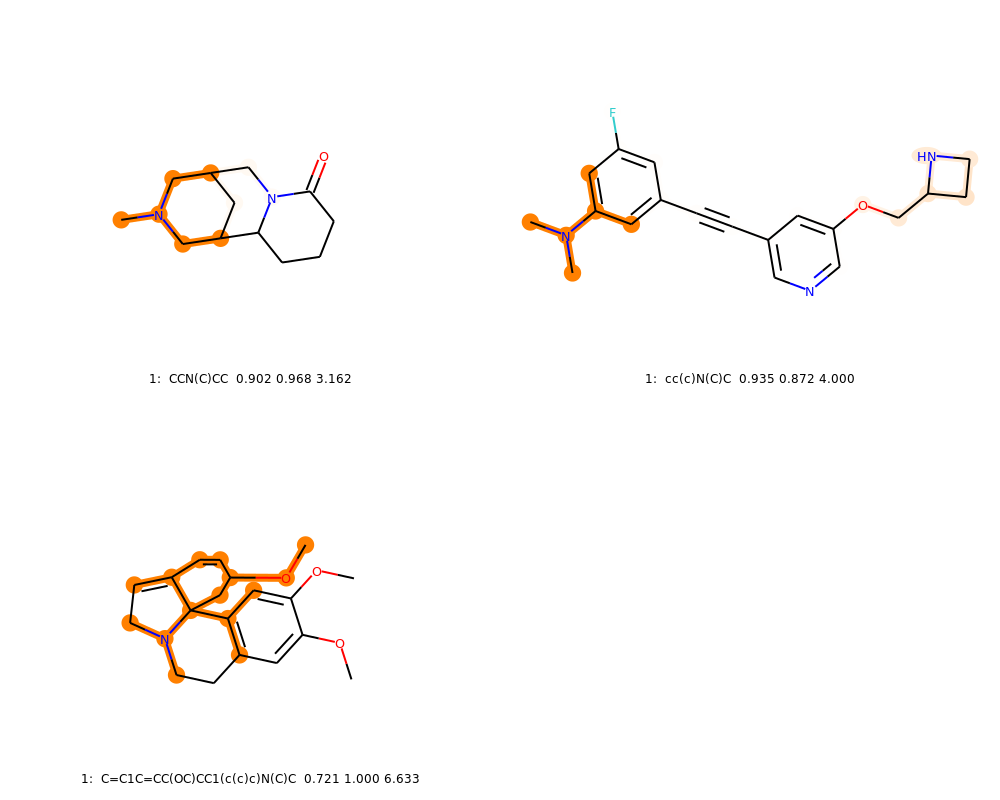

Supplement: Supplementary file 4 — Supplementary file4 (ZIP 111545 KB) [file 10822_2021_421_MOESM4_ESM.zip › 069/tp_cluster_1/tp1_mols_8.png]

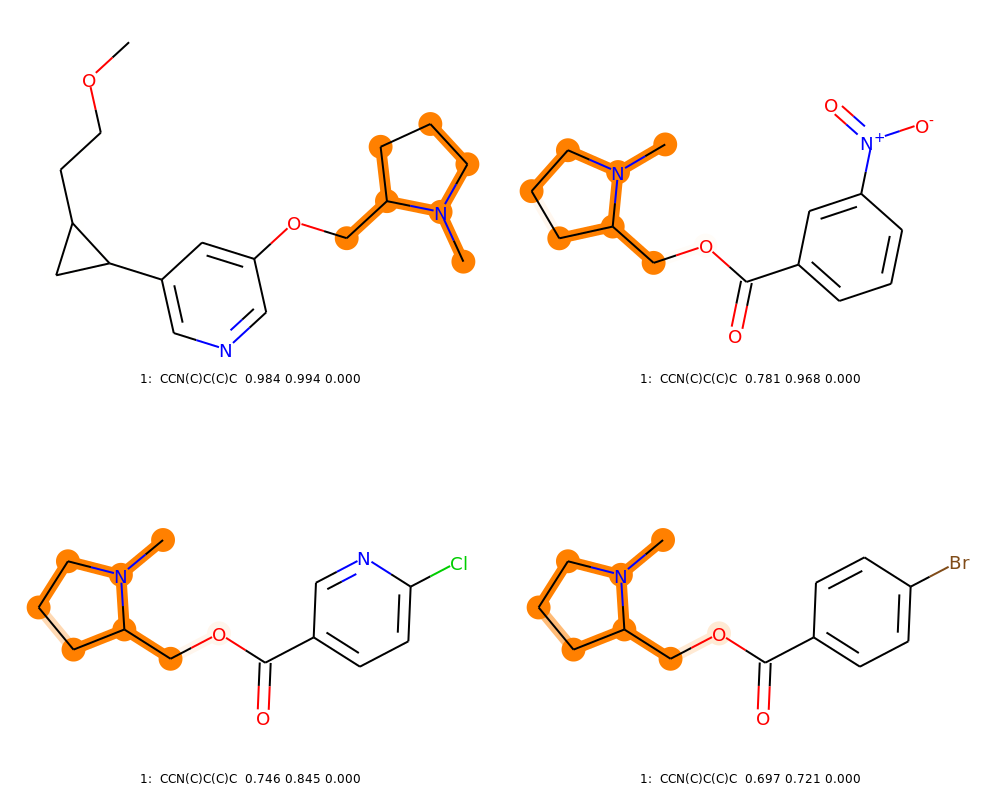

Supplement: Supplementary file 4 — Supplementary file4 (ZIP 111545 KB) [file 10822_2021_421_MOESM4_ESM.zip › 069/tp_cluster_1/tp1_mols_0.png]

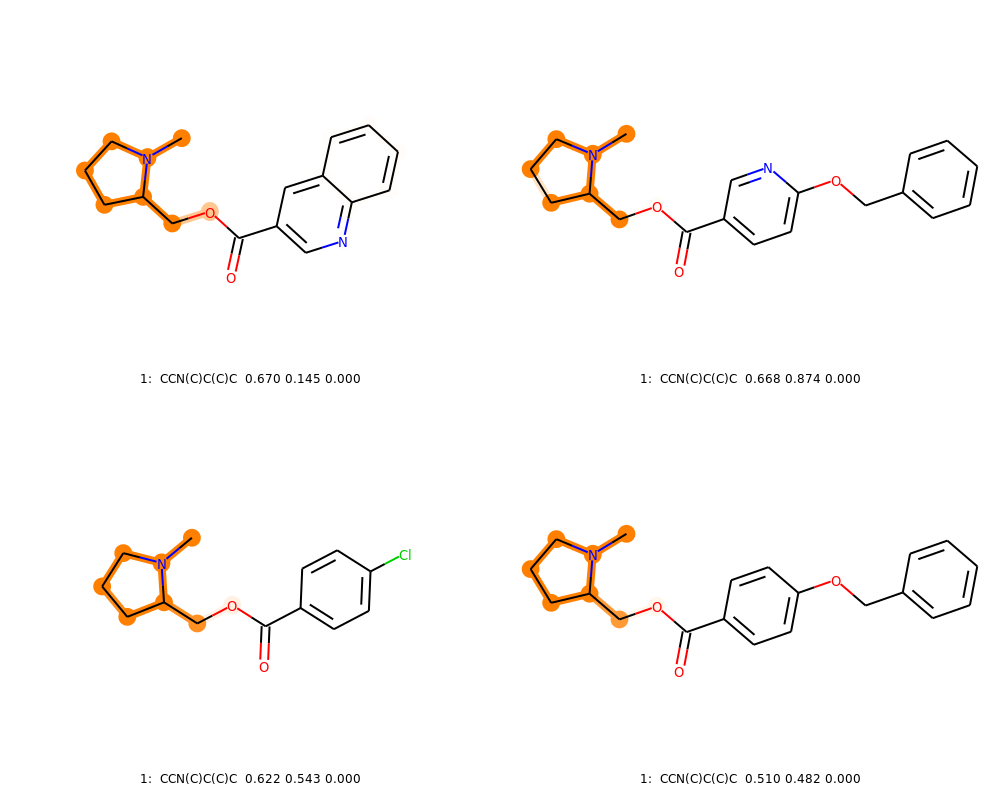

Supplement: Supplementary file 4 — Supplementary file4 (ZIP 111545 KB) [file 10822_2021_421_MOESM4_ESM.zip › 069/tp_cluster_1/tp1_mols_4.png]

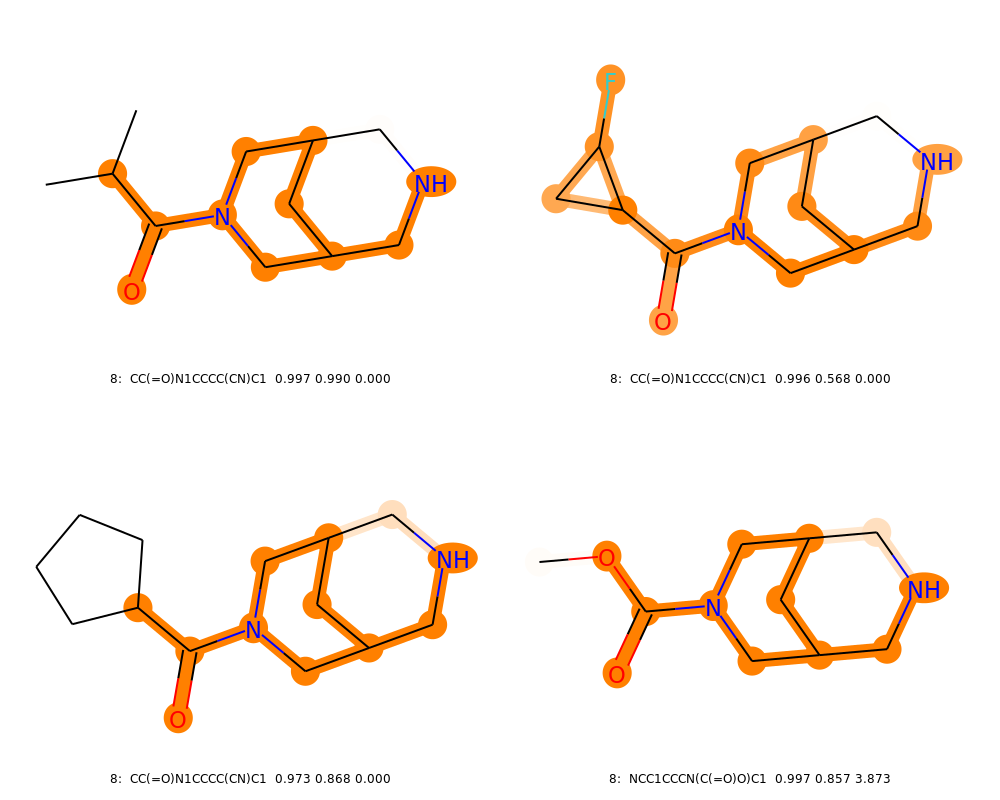

Supplement: Supplementary file 4 — Supplementary file4 (ZIP 111545 KB) [file 10822_2021_421_MOESM4_ESM.zip › 069/tp_cluster_8/tp8_mols_0.png]

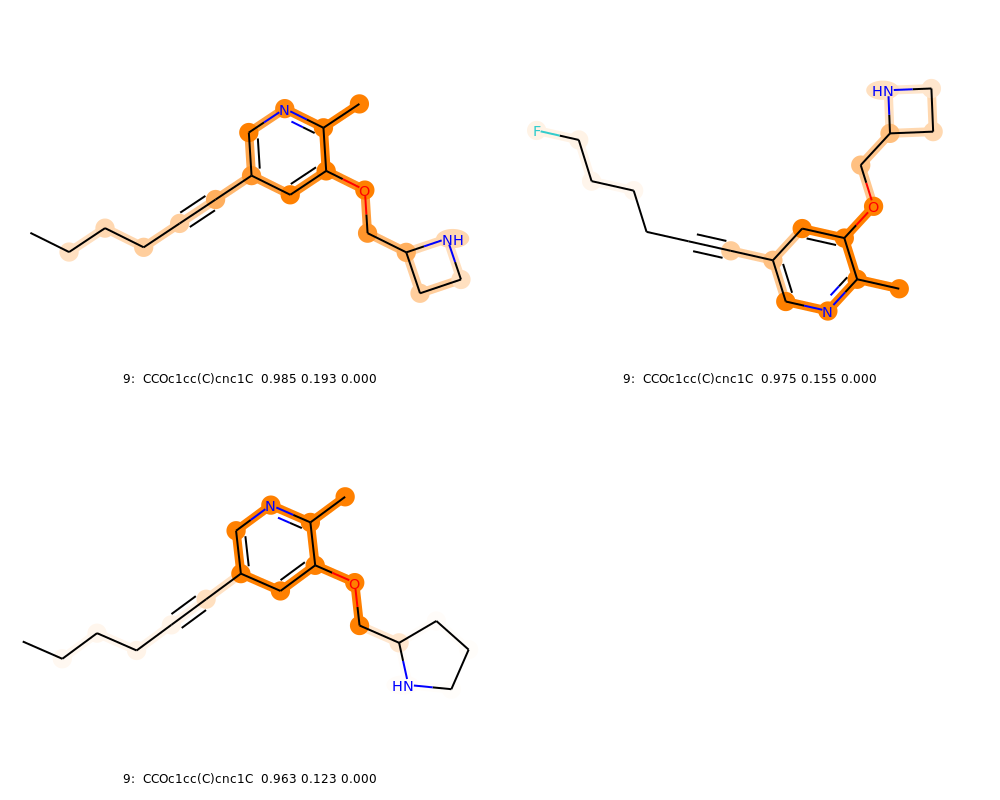

Supplement: Supplementary file 4 — Supplementary file4 (ZIP 111545 KB) [file 10822_2021_421_MOESM4_ESM.zip › 069/tp_cluster_9/tp9_mols_0.png]

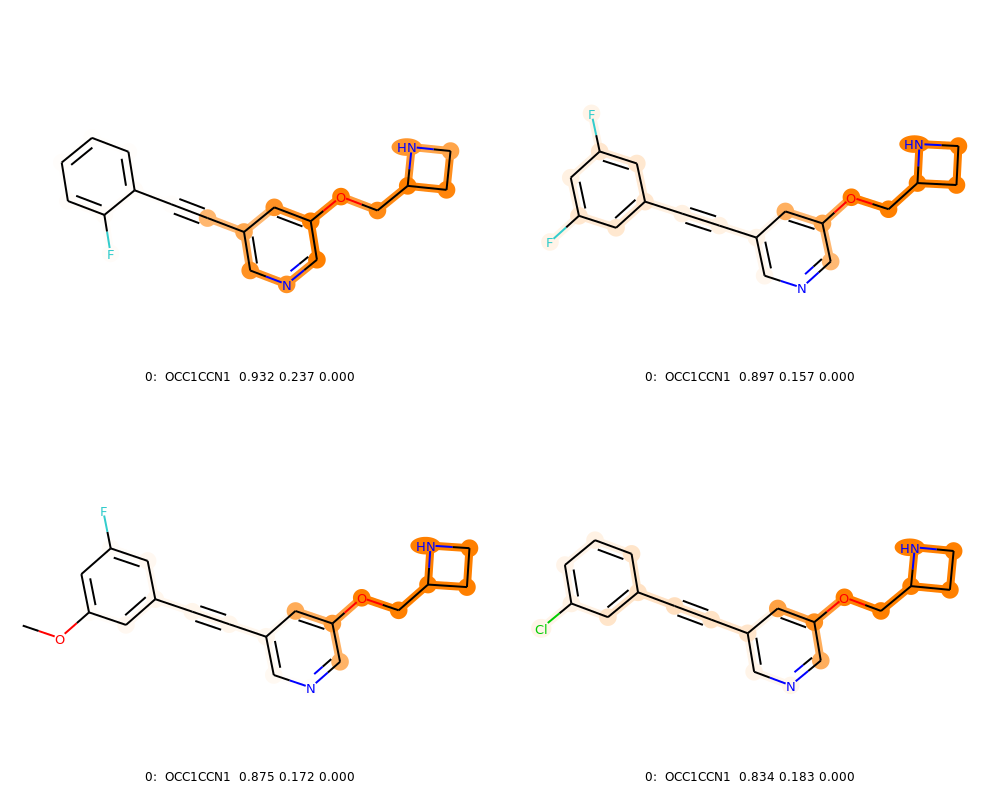

Supplement: Supplementary file 4 — Supplementary file4 (ZIP 111545 KB) [file 10822_2021_421_MOESM4_ESM.zip › 069/tp_cluster_0/tp0_mols_4.png]

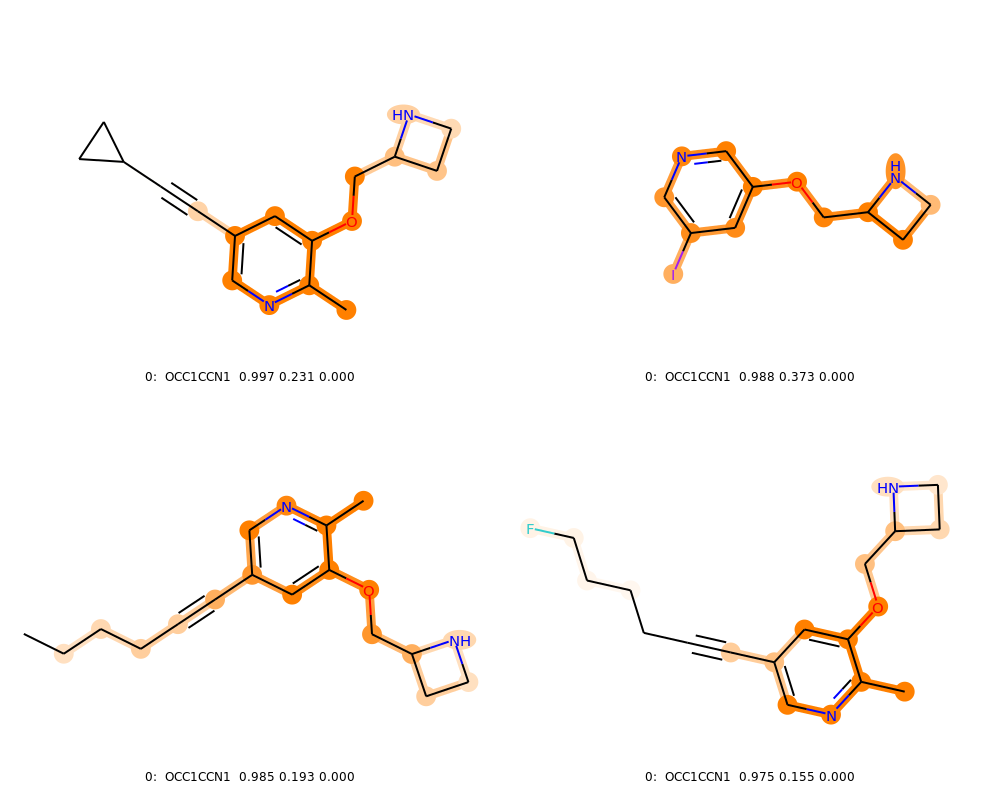

Supplement: Supplementary file 4 — Supplementary file4 (ZIP 111545 KB) [file 10822_2021_421_MOESM4_ESM.zip › 069/tp_cluster_0/tp0_mols_0.png]

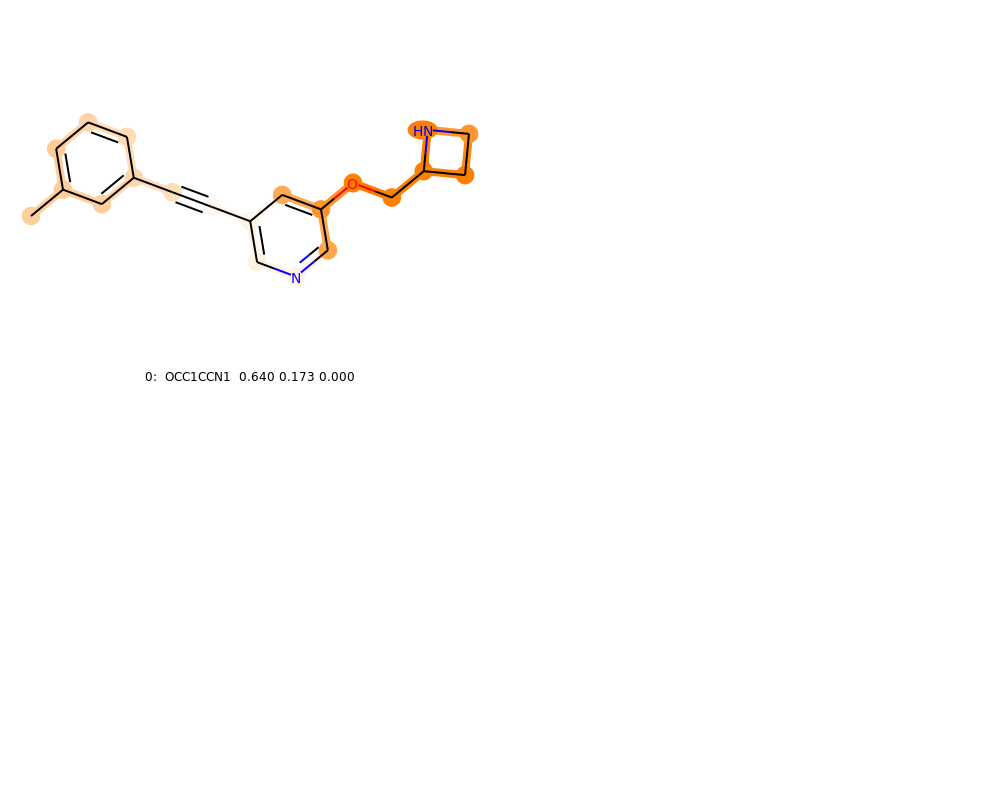

Supplement: Supplementary file 4 — Supplementary file4 (ZIP 111545 KB) [file 10822_2021_421_MOESM4_ESM.zip › 069/tp_cluster_0/tp0_mols_8.png]

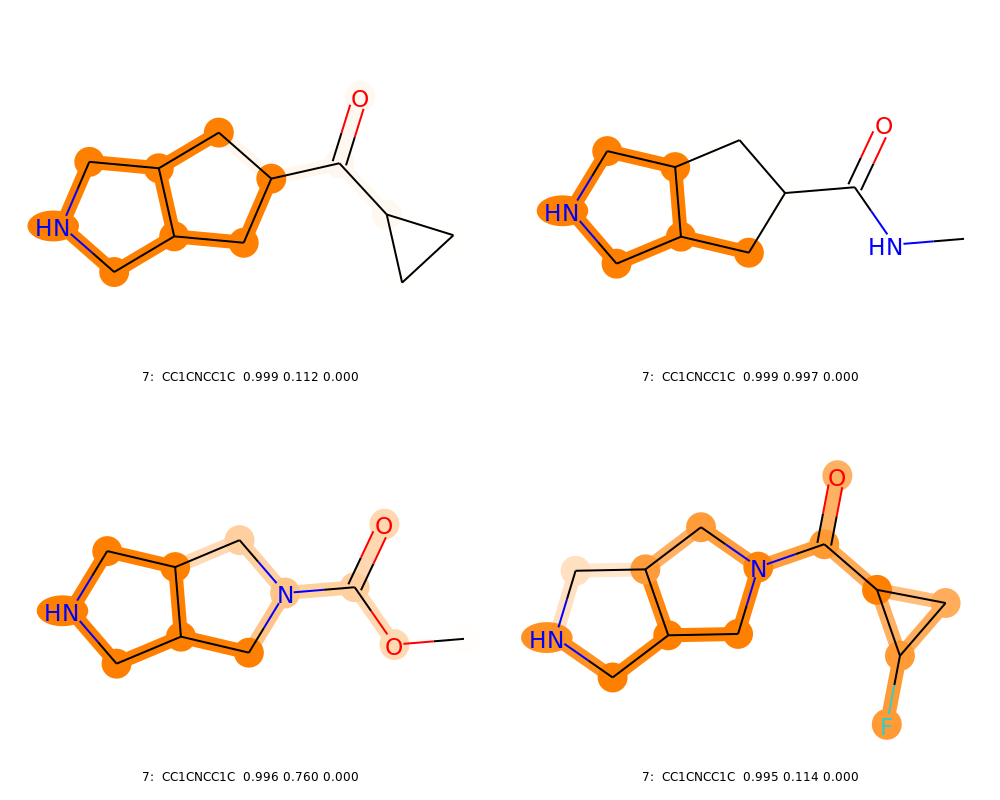

Supplement: Supplementary file 4 — Supplementary file4 (ZIP 111545 KB) [file 10822_2021_421_MOESM4_ESM.zip › 069/tp_cluster_7/tp7_mols_0.png]

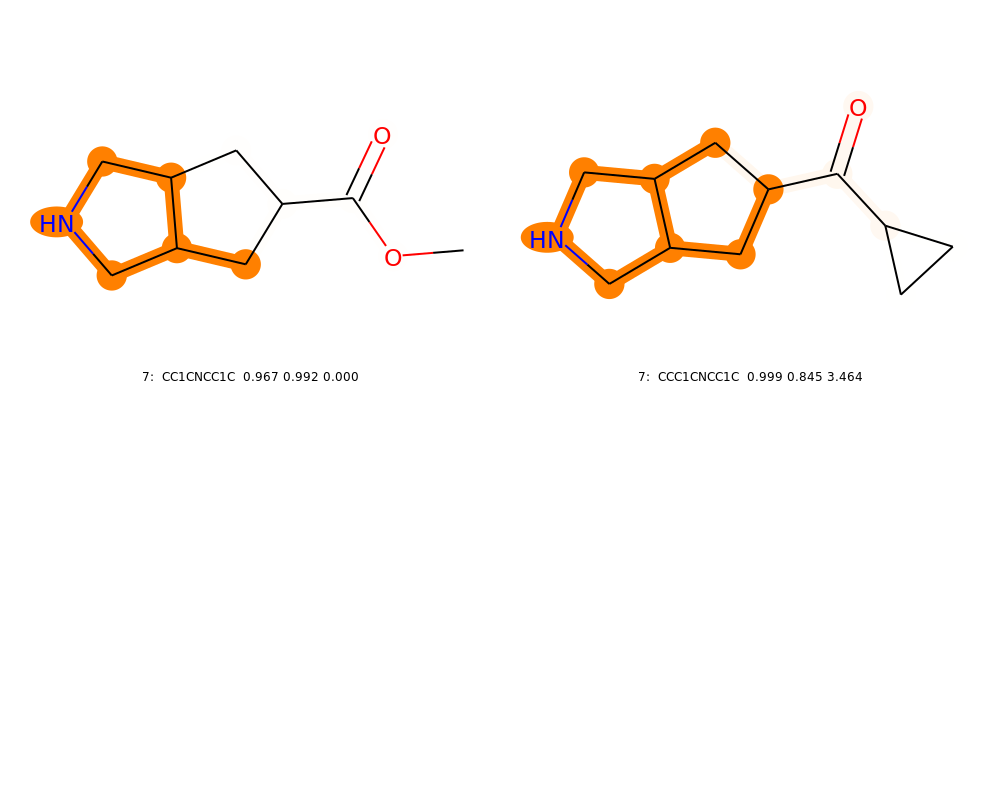

Supplement: Supplementary file 4 — Supplementary file4 (ZIP 111545 KB) [file 10822_2021_421_MOESM4_ESM.zip › 069/tp_cluster_7/tp7_mols_4.png]

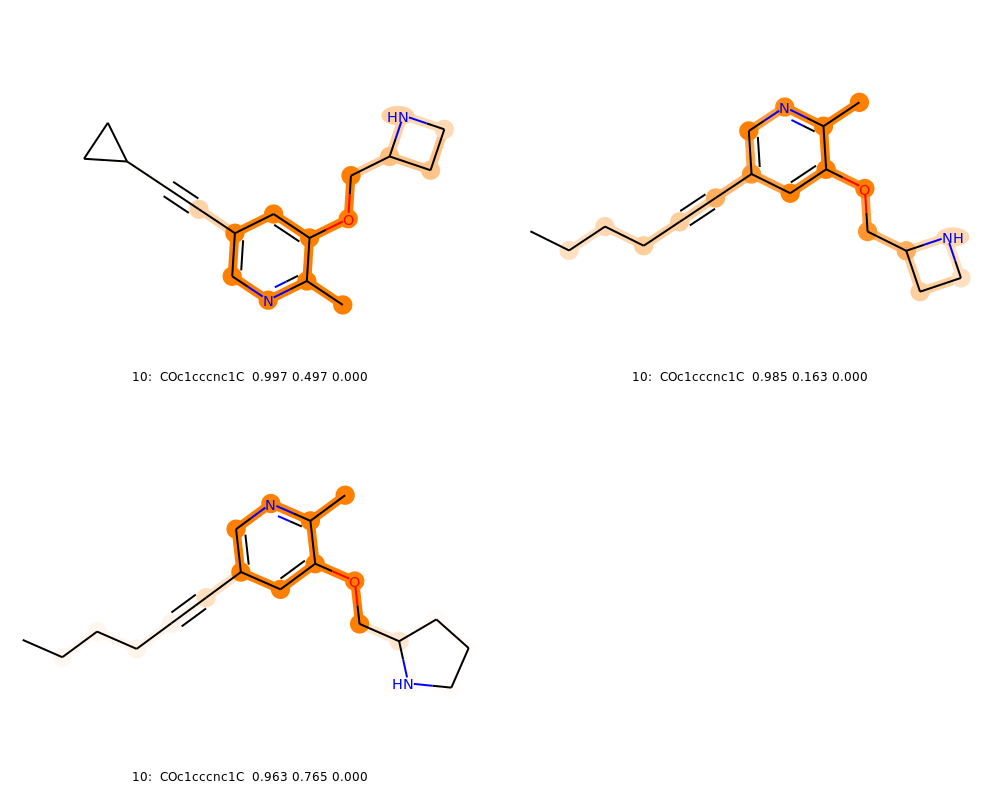

Supplement: Supplementary file 4 — Supplementary file4 (ZIP 111545 KB) [file 10822_2021_421_MOESM4_ESM.zip › 069/tp_cluster_10/tp10_mols_0.png]

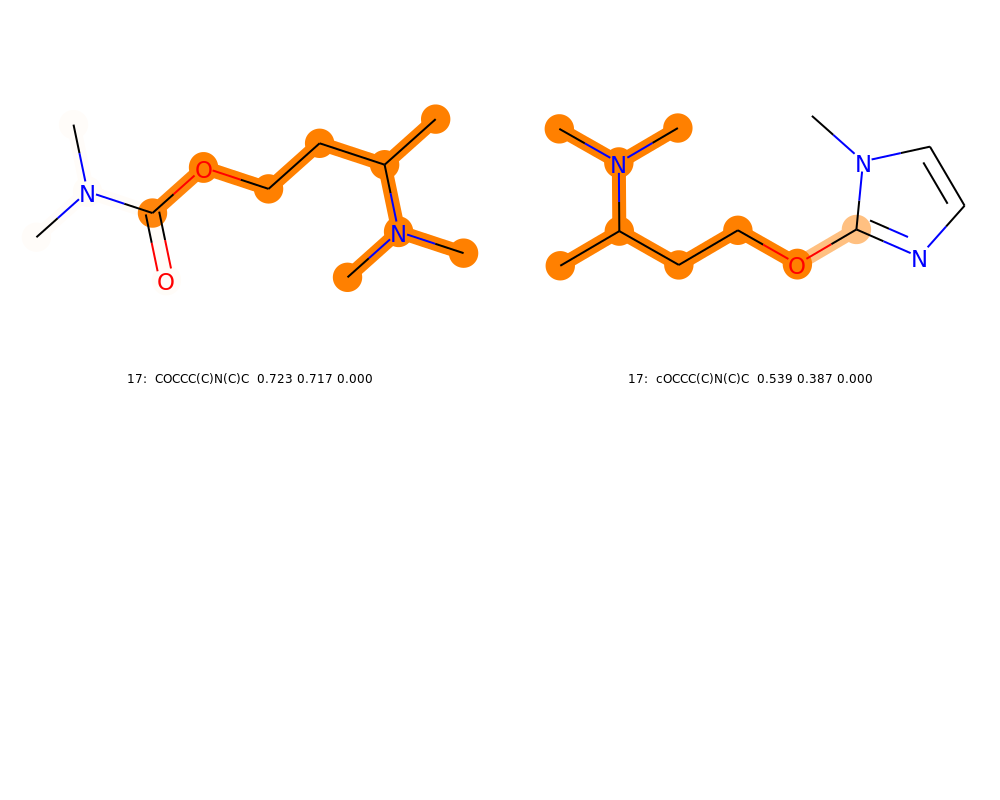

Supplement: Supplementary file 4 — Supplementary file4 (ZIP 111545 KB) [file 10822_2021_421_MOESM4_ESM.zip › 069/tp_cluster_17/tp17_mols_0.png]

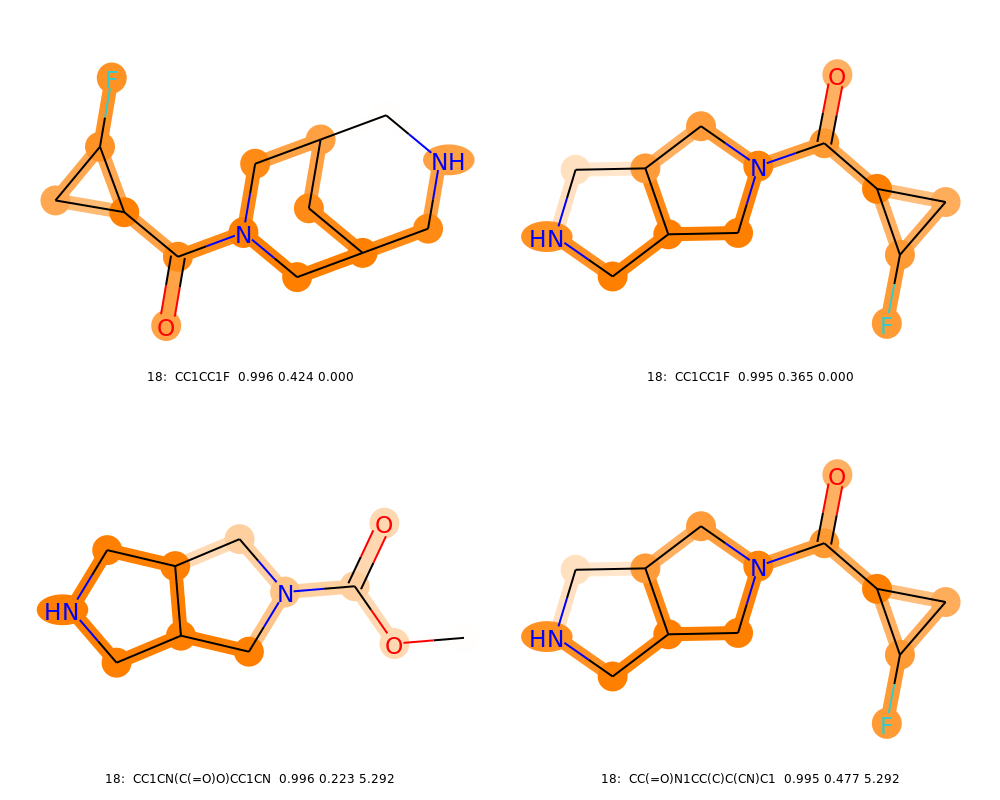

Supplement: Supplementary file 4 — Supplementary file4 (ZIP 111545 KB) [file 10822_2021_421_MOESM4_ESM.zip › 069/tp_cluster_18/tp18_mols_0.png]

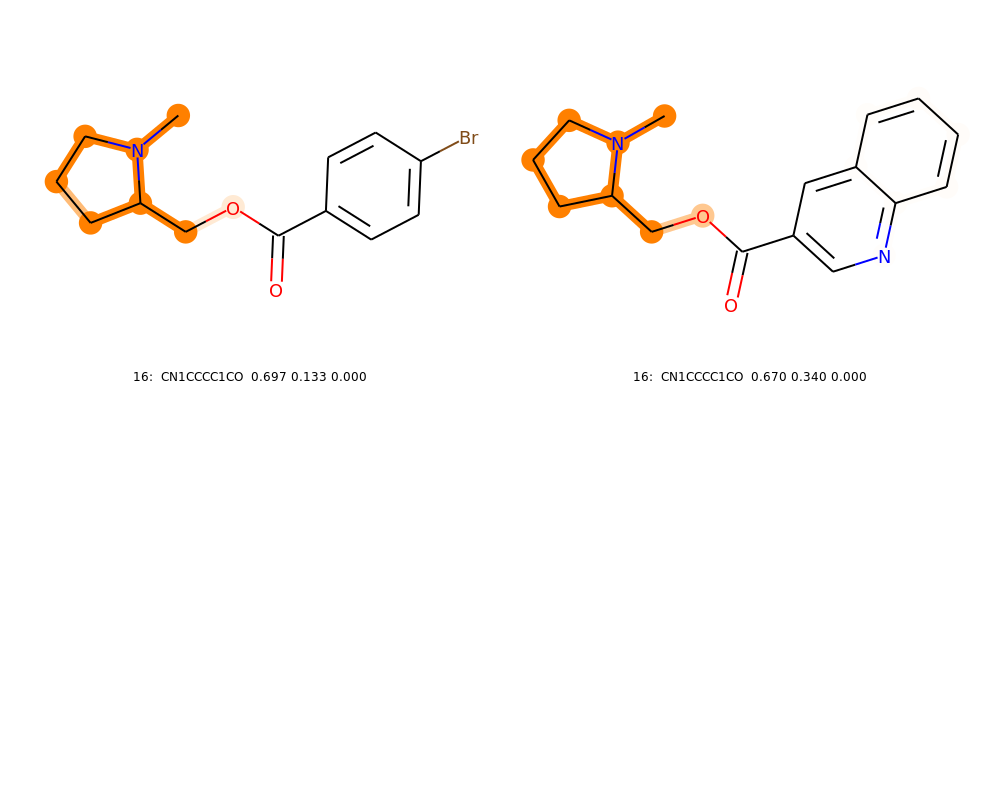

Supplement: Supplementary file 4 — Supplementary file4 (ZIP 111545 KB) [file 10822_2021_421_MOESM4_ESM.zip › 069/tp_cluster_16/tp16_mols_0.png]
